# Metagenome and metabolome study on inhaled corticosteroids in asthma patients with side effects

**DOI:** 10.1515/jib-2024-0062

**Published:** 2025-06-24

**Authors:** Igor Goryanin, Anatoly Sorokin, Meder Seitov, Berik Emilov, Muktarbek Iskakov, Irina Goryanin, Batyr Osmonov

**Affiliations:** School of Informatics, University of Edinburgh, Edinburgh, UK; Okinawa Institute Science and Technology, Okinawa, Japan; KGDiscovery LTD, Edinburgh, UK; Kyrgyz State Medical Academy Named After I.K. Akhunbaev, Bishkek, Kyrgyzstan; MMWR LTD, Edinburgh, UK

**Keywords:** microbiome, metabolome, asthma, drug side effects, novel therapeutics interventions, GSA

## Abstract

This study investigates the gut microbiome and metabolome of asthma patients treated with inhaled corticosteroids (ICS), some of whom experience adverse side effects. We analyzed stool samples from 24 participants, divided into three cohorts: asthma patients with side effects, those without, and healthy controls. Using next-generation sequencing and LC-MS/MS metabolomics, we identified significant differences in bacterial species and metabolites. Multi-Omics Factor Analysis (MOFA) and Global Sensitivity Analysis-Partial Rank Correlation Coefficient (GSA-PRCC) provided insights into key contributors to side effects, such as tryptophan depletion and altered linolenate and glucose-1-phosphate levels. The study proposes dietary or probiotic interventions to mitigate side effects. Despite the limited sample size, these findings provide a basis for personalized asthma management approaches. Further studies are required to confirm initial fundings.

## 1 Introduction

Asthma is a complex respiratory condition that affects millions globally, often managed through therapies like inhaled corticosteroids and beta-agonists, such as salmeterol. While effective, these therapies can induce adverse side effects, particularly after long-term use. Understanding the underlying causes of these side effects has become a focal point in asthma research. Recent advances emphasize the significant role of the gut microbiome in modulating systemic immunity and influencing lung health, primarily through the gut-lung axis. This bidirectional communication between the gut and lungs is now thought to be pivotal in shaping immune responses and the pathophysiology of chronic respiratory diseases such as asthma.

This relationship is highlighted in recent studies showing that gut microbiota can influence the respiratory microbiome, altering lung immunity and the effectiveness of treatments like corticosteroids [1], 2].

Recent research into the gut-lung axis has demonstrated that corticosteroid use not only affects the bronchial microbiome but also impacts the gut microbiome, potentially leading to side effects in patients [3], 4].

Given these findings, we chose to investigate the gut microbiome in this study to uncover the microbial signatures associated with the adverse side effects of inhaled corticosteroids, particularly salmeterol. By using next-generation sequencing and metabolomic profiling, we aim to delineate the key microbial taxa and metabolites involved. This will not only enhance our understanding of the gut-lung axis but also pave the way for more personalized and potentially microbiome-targeted interventions, such as dietary modifications or probiotics, to mitigate these side effects [3], 5].

As recent multi-omics studies have shown, specific gut bacteria and metabolites are increasingly implicated in modulating the body’s response to asthma therapies, making this a promising area for further exploration.

The relationship between the respiratory microbiome and pulmonary disease pathophysiology is garnering increasing recognition in the medical field. Inhaled corticosteroids (ICS), which are a mainstay in the therapeutic armamentarium for chronic respiratory ailments such as asthma and chronic obstructive pulmonary disease (COPD), have systemic ramifications that may extend to gut microbiota, influencing both drug metabolism and patient response to treatment [6], 7]. Current literature reveals a marked heterogeneity in research methodologies when investigating the impact of corticosteroids on the microbiome, leading to inconsistent and sometimes contradictory findings [8], 9]. This diversity in study outcomes, ranging from reports of increased to decreased microbial diversity following corticosteroid therapy, underscores a pressing need for a cohesive analytical framework to decipher the true impact of these drugs on microbial communities. Traditional microbiome analyses are often dependent on semi-automated processes that require substantial manual curation, potentially overlooking the nuanced intricacies present in meta-omics data, especially those about metabolomics. We used Multi-Omics Factor Analysis (MOFA) and Flux Balance Analysis Partial Rank Correlation Coefficient (FBA-PRCC) [10] platforms. We have used this approach for human [11] and environmental microbiome studies [12].

## 2 Materials and methods

### 2.1 Clinical

The clinical arm of this study involved a cohort of 24 participants (equal gender), stratified into three distinct groups: individuals diagnosed with asthma experiencing inhaled corticosteroids (ICS) related side effects (Asthma.YES), and patients who do not experience any side effects from treatment. These patients tolerate the therapy well, without any negative reactions or complications (Asthma.NO), and a control group of healthy (HEALTHY) individuals. There are eight subjects per group.

The participants in the study were diagnosed more than 1 year ago with moderate, partially controlled Non-Allergic Asthma (Not linked to allergies). They were prescribed inhaled corticosteroids, which were used for more than three weeks. The participants did not have any other allergic conditions, and they were not taking any other medications. The patients were using inhalers 25 mcg/125 mcg, each single actuation provides salmeterol xinafoate equivalent to 25 μg of salmeterol and 125 μg of fluticasone propionate, 2 puffs 2 times daily.

Inclusion Criteria:

– Adults aged 18–65 years with a confirmed diagnosis of asthma.
– Regular use of inhaled corticosteroids for a minimum of 3 months.
– Ability to provide informed consent and comply with study procedures.

Exclusion Criteria:

– Use of systemic corticosteroids within the past 3 months.
– Recent respiratory infections (within the past 4 weeks).
– Presence of other chronic respiratory diseases (e.g., COPD, bronchiectasis).
– Pregnancy or lactation.

The side effects include:

– Hoarseness of voice (dysphonia): This occurs due to the deposition of corticosteroids on the vocal cords, leading to local muscle atrophy and mucosal irritation.
– Dry mouth and throat irritation: ICS can reduce salivary secretion and cause mucosal dryness, resulting in discomfort and increased thirst.
– Tachycardia and palpitations: These are attributed to the systemic absorption of ICS and the β2-agonist components in combination inhalers, which can stimulate cardiac β2-adrenergic receptors.
– Severe cough: This may result from local irritation of the airway mucosa due to the inhaled particles.
– Rashes on the mucous membranes of the mouth: Prolonged ICS use can lead to local immunosuppression, making the oral mucosa susceptible to infections and inflammatory reactions, presenting as rashes.

Each participant in the asthma group taking ICS was examined by professional pulmonologists to control asthma symptoms, as well as for the correct technique of using inhaled medications. For control symptoms, there were clinical examinations by a pulmonologist with the collection of all complaints, and patients also used the asthma control test questionnaire [13], 14], as well as the introduction of self-monitoring of peak expiratory flows [15]. To control the correct use of ICS, patients demonstrated their skills to the pulmonologist according to the rules of inhalation using an aero chamber [16].

Alongside stool sample collection, baseline health metrics including height, body mass index (BMI), and blood pressure were systematically recorded for each participant to provide a comprehensive clinical profile. The collection protocol was meticulously followed, ensuring the uniformity and integrity of the collected samples. Post-collection, all biological materials were immediately flash-frozen at −82 °C to preserve microbial and molecular fidelity, as recommended for microbiome and metabolomic analyses [17].

The study design, including the sampling framework and participant recruitment strategy, was reviewed and received ethical approval from the Kyrgyz National Surgical Center named after Mamakeev Ethics Committee on 07 June 2022, protocol number N16. [Supplementary Material 4]. This endorsement ensured adherence to ethical standards and the safeguarding of participant welfare throughout the study. The informed consent was obtained from all subjects and/or their legal guardian(s). Further details are available in Supplementary Material 4.

### 2.2 Sample preparation

The integrity of biological samples is critical for the reliability of downstream meta-omics analyses. Consequently, all collected materials were frozen at −82 °C immediately upon collection. This stringent protocol for sample preservation and storage was integral to minimizing microbial and enzymatic activity, thereby preserving the native microbial and metabolomic profiles for subsequent analysis. Furthermore, the samples were transported under cryogenic conditions for overseas analysis, ensuring the stability and integrity of the samples were maintained throughout the logistic process.

Specifically, stool samples were collected following the standardized procedures outlined [17], 18], ensuring the preservation of microbial DNA integrity for subsequent analysis.

### 2.3 DNA. Whole genome sequencing (WGS)

The genomic library was prepared to adhere to the DNBseq technology guidelines, employing a paired end read length of 150 nucleotides (PE150).

The WGS data for the stool microbiome were initially processed using the nf-core/tax-profiler pipeline, incorporating a series of quality control steps with FastQC and adapter sequence handling with Fastp. BBDuk was utilized for filtering out low-complexity sequences and those of low quality. Raw data with adapter sequences or low-quality sequences was filtered. We went through a series of data processing to remove contamination and obtain valid data. This step was completed by SOAPnuke [19] with the following filter parameters: “ −n 0.01, −l 20, −q 0.4, −adaMis 3, −outQualSys 1, −minReadLen 150”.

Other steps include:

1. Filter adapter: if the sequencing read matches 50.0 % or more of the adapter sequence (maximum 3 base mismatches are allowed), remove the entire read.
2. Filter read length: if the length of the sequencing read is less than 150 bp, discard the entire read.
3. Remove N: if the N content in the sequencing read accounts for 1.0 % or more of the entire read, discard the entire read.
4. Filter low-quality data: if the bases with a quality value of less than 20 in the sequencing read account for 40.0 % or more of the entire read, discard the entire read.
5. Obtain clean reads: the output read quality value system is set to Phred+64, which is integral for ensuring accurate base calling in sequencing data [20], 21].
6. Subsequent processing involved the removal of adapter sequences, potential contaminants, and low-quality reads, aligning with the best practices for next-generation sequencing data analysis [21]. See Supplementary Material 1–3 for more technical details.

### 2.4 Metabolomics

The analytical setup encompassed a Low-Speed Cryogenic Universal Centrifuge (Centrifuge 5430, Eppendorf), a Vortex Mixer (QL-901, Kylin-Bell Lab Instruments Co., Ltd.), and an Ultra-pure Water Meter (Milli-Q Integral, Millipore Corporation, USA). Sample preparation was executed using a Refrigerated Vacuum Concentrator (Maxi Vacbeta, GENE COMPANY). Sample disaggregation utilized a Tissue Grinder (JXFSTPRP, ShanghaiXinNing, China). Reagents of analytical calibration, including Methanol (A454-4) and Acetonitrile (A998-4) obtained from Thermo Fisher Scientific, Ammonium Formate (17,843–250 G, Honeywell Fluka), and Formic Acid (50,144–50 ml, DIMKA) were employed in the protocol.

Metabolite extraction commenced with a gradual defrosting at 4 °C, followed by the weighing of 25 mg of sample. The sample was combined with 800 µL of a pre-chilled extraction solvent (methanol:acetonitrile:water, 2:2:1,v/v/v) and 10 µL of an internal standard within a 1.5 ml Eppendorf tube. Tissue disaggregation was achieved via grinding with steel balls (50 Hz, 5 min), succeeded by ultrasonication in a 4 °C water bath for 10 min and a subsequent 1-h stabilization at −20 °C. Centrifugation at 25,000 g for 15 min at 4 °C facilitated the collection of the supernatant, which, following vacuum concentration, was re-dissolved in a 600 µL mixture of acetonitrile:water (7:3, v/v). Post-vortex and centrifugation, the supernatant was allocated into analysis vials. A composite quality control (QC) sample was formulated by amalgamating 50 µL from each sample to ensure analytical reproducibility in the LC-MS procedure [22].

Metabolite separation and detection were facilitated by a Waters UPLC I-Class Plus system coupled with a Q Exactive high-resolution mass spectrometer (Thermo Fisher Scientific, USA). Chromatographic separation was conducted on a BEH Amide column (1.7 µm, 2.1 × 100 mm, Waters, USA) employing a dual-solvent system: Mobile phase A (95 % acetonitrile with 10 mM ammonium formate and 0.1 % formic acid) and Mobile phase B (50 % acetonitrile with 10 mM ammonium formate and 0.1 % formic acid), both pH-adjusted to 9 using ammonia. The column temperature was maintained at 30 °C, utilizing a gradient elution program. The flow rate was set at 0.35 mL/min, and the injection volume was 2 µL.

Mass spectrometric analysis was conducted using the Q Exactive system, configured for both primary and secondary mass spectrometry data acquisition. Operational parameters included a full scan range of 70–1,050 *m*/*z*, with resolution settings of 70,000 for MS and 17,500 for MS/MS. The automatic gain control (AGC) target for MS acquisitions was set to 3e6, with stepped normalized collision energy (15, 30, and 45 eV) applied for precursor fragmentation. Sheath and auxiliary gas flow rates, as well as electrospray ionization voltages, were optimized for both positive and negative ion modes, and temperatures for the capillary and auxiliary gas heater were regulated at 320 °C and 350 °C, respectively.

Offline mass spectrometry data were imported into Compound Discoverer 3.3 (Thermo Fisher Scientific, USA) for metabolite identification and quantification against established databases such as the BGI Metabolome Database (bmdb), mzCloud Database, and ChemSpider. The generated data matrix, comprising metabolite peak areas and identification results, was further processed using the Compound Discoverer 3.3 platform (https://mycompounddiscoverer.com/).

The metaX software platform was pivotal in preprocessing this data, enabling the creation of detailed compound profiles and intensity metrics required for downstream analysis. This step was critical in discriminating between human and bacterial metabolites.

The software’s algorithms employed Probabilistic Quotient Normalization (PQN) for enhancing sample comparability: for every feature the mean response is calculated across all QC samples. A reference vector is then generated. The median between the reference vector and every sample is computed obtaining a vector of coefficients related to each sample. Each sample is then divided by the median value of the vector of coefficients; this median value is different for each sample.

Quality Control-based Robust LOESS (QC-RLSC) was used for correcting batch effects. Furthermore, a cutoff was established to exclude metabolites with a Coefficient of Variation greater than 30 % in the quality control (QC) samples, ensuring data reliability [23], 24].

### 2.5 Bioinformatics

Taxonomic categorization was conducted using Kraken2, supplemented by Bracken for species-level read reassignment. The differential abundance of microbial species across the cohorts was assessed with the DESeq2 R package, providing a quantitative landscape of microbial presence, FDR cutoff *α* = 0.1 was used to adjust p-value by the Benjamini–Hochberg procedure. To further probe into the meta-omics data, Multi-Omics Factor Analysis (MOFA) and Advance Software (ASAR) were employed to perform to facilitate advanced integrative analysis [25], [26], [27], [28], [29], [30], [31]. R package MOFA2 was used to calculate MOFA factors, source code is available from the GitHub repository.

The innovative GSA FBA-PRCC [32] methodology was leveraged to dissect the influence of exo-metabolite production and consumption on bacterial growth dynamics. By integrating metagenomic and metabolomic datasets, the platform provided insights into the stability of microbial communities and the roles of specific microbial taxa and metabolites within these complex ecosystems [32].

## 3 Results

### 3.1 Taxonomical analysis

Our analysis revealed alterations in the abundance of various bacterial strains, underlining the complex influence of corticosteroids on the microbiota that extends beyond specific strains [33]. Notably, we identified an increased abundance of the following pathogens in asthma patients with and without corticosteroid side effects, designated in Table 1 as ASTHMA.YES and ASTHMA.NO respectively. The differential abundance of species was quantified using the DeSEQ2 R package, which demonstrated that specific species are overrepresented in asthma patients on corticosteroids [34].

**Table 1:** Top overabundant species in all Asthma patients compared to healthy individuals. Bonferroni multiple testing correction method was used. P-adj < 0.009.

Species	TaxID	BaseMean	logFoldChange	p-adj
*Megamonas funiformis*	437,897	46,142	8.61	2.89e-09
*Bacteroides stercoris*	46,506	112,090	4.16	0.00051
*Phascolarctobacterium faecium*	33,025	35,209	4.92	0.00131
*Escherichia coli*	562	157,018	3.15	0.0059
*Escherichia fergusonii*	564	445	2.79	0.0059
*Limosilactobacillus mucosae*	97,478	366	3.19	0.00847
*Bifidobacterium pseudocatenulatum*	28,026	27,749	3.9	0.00885

Table 1 shows bacterial species that are present in higher quantities in the gut microbiome of asthma patients when compared to healthy individuals. *Megamonas funiformis* reduces complement C3 level, glucocorticoids and aldosterone (in alveolar epithelium) as well. So, the bacteria may increase the side effects of these steroids via complement C3 [35].

*Bacteroides stercorischanges* in abundance. have been correlated with asthma [36]. *Phascolarctobacterium faecium* is noted for its SCFA production. SCFAs have been implicated in the modulation of systemic immune responses, which are relevant to asthma [37]*. Limosilactobacillus mucosae* is traditionally linked with healthy mucosal surfaces, the implications of its abundance in asthma patients are not fully understood and represent a novel area of research [37]. *Bifidobacterium pseudocatenulatum and Bifidobacterium longum* are known for their health-promoting effects, including the potential suppression of allergic reactions, their unexpected prevalence in the gut microbiome of asthma patients may indicate a nuanced interaction with the host immune system [38].

Details of microbial species (Table 2) that are found in greater abundance in healthy individuals compared to those with asthma, potentially contributing to gut homeostasis and overall health.

**Table 2:** Some species are over-represented in healthy patient samples (or under-represented in Asthma patients). P-adj < 0.009.

Species	TaxID	BaseMean	logFoldChange	p-adj
*Blohavirus americanus*	2,955,510	119	26.4	1.27E-14
*Catenibacterium sp. co_0103*	2,478,954	5080	3.8	0.00415
*Catenibacterium mitsuokai*	100,886	2,488	2.93	0.00509
*Desulfovibrio desulfuricans*	876	1,397	3.4	0.00847

Research on this specific bacteriophage like *Blohavirus americanus*: is minimal; however, the role of phages in regulating the bacterial populations and the immune response is an area of active research. Phages are increasingly being studied for their therapeutic potential and their impact on the microbiome [39]*. Catenibacterium sp. co_0103* and *Catenibacterium mitsuokai* are part of the gut microbiota [40]. Known as a sulfate-reducing bacterium, the genus *Desulfovibrio* has been implicated in various gut-related issues. However, its role in asthma and respiratory health has not been well established and is being explored [41]. As mentioned earlier, variations in *Bacteroides spp.* have been correlated with several health conditions, including asthma [42].

Table 3 highlights a range of microbial species whose numbers are notably higher in the gut microbiomes of asthma patients with no side effects in comparison to the patients with side effects. Bacteriophages may impact gut bacterial populations and influence the side effects [43]. Typically, environmental *Chryseobacterium rhizoplanae and Polaribacter sejongensis* might indicate a state of dysbiosis. Such changes in microbial composition could potentially be linked to the immunomodulatory effects of corticosteroids [44]. While *Bifidobacterium spp*. is generally recognized for their health-promoting roles, an increase may reflect a compensatory reaction to inflammation or indicate a disrupted microbiome due to corticosteroid treatment [45].

**Table 3:** Some overabundant species in Asthma patients on steroids without side effects in comparison with patients on steroids with side effects. P-adj < 6E-11.

Species	TaxID	BaseMean	logFoldChange	p-adj
*Buchavirus oralis*	2,955,554	352	26.4	2.92E-37
*Buchavirus hiberniae*	2,955,551	217	25.7	1.10E-32
*Chryseobacterium rhizoplanae*	1,609,531	27.8	22.9	1.71E-21
*Polaribacter sejongensis*	985,043	24.4	22.7	4.53E-21
*Buchavirus splanchnicus*	2,955,555	207	25.6	6.28E-19
*Buchavirus coli*	2,955,548	201	25.6	8.89E-19
*Paenibacillus sp. JNUCC-32*	2,777,984	16.9	22.2	2.10E-16
*Bifidobacterium sp. KRGSERBCFTRI*	2,985,571	553	26.9	8.80E-15
*Afonbuvirus coli*	2,955,232	318	25.8	1.63E-13
*Birpovirus hiberniae*	2,955,498	57.7	23.4	3.31E-13
*Spiroplasma phoeniceum*	47,835	11.1	21.7	5.89E-12
*Blohavirus americanus*	2,955,510	121	24.2	7.37E-12
*Streptomyces lividans*	1916	38.9	23.4	5.38E-11

### 3.2 MOFA analysis

Advancements in statistical software now permit the simultaneous analysis of diverse omics data derived from the same sample set. For this study, we employed the MOFA2 [46] package to uncover latent factors that simultaneously describe metagenomics and metabolomics data.

Three distinct analyses were conducted: the first encompassed all 23,970 features detected by LC/MS pipeline, the second analysis was limited to only the 5,081 identified metabolites, excluding the 18,889 features that lacked molecular annotation, and the third analysis was limited to as little as 1,214 metabolites that have KEGG reference [47].

Table 4 summarizes the variance explained in MOFA model by both metabolomics and metagenomics data. It is interesting that despite a decrease in the metabolomics dataset by order of magnitude the proportion of variance explained by metabolites did not change much.

**Table 4:** Total variance in Metabolomic and Metagenomic Features by MOFA, three datasets with the same 8902 metagenomics features and all 24 K metabolic features (Full data), 5 K identified metabolites (Identified metabolites) and 1200 KEGG-identified metabolites (KEGG metabolites) were analyzed.

Data	Full data variance explained (%)	Identified metabolites variance explained (%)	KEGG metabolites variance explained (%)
Metabolomics	63.5	61.2	56.9
Metagenomics	11.8	17.1	17.6

These results indicate that metabolomics data account for a larger proportion of the variance explained by the MOFA model compared to metagenomics data, suggesting that metabolites may have a more pronounced role in the observed differences between patients. However, the significant variance in metagenomics data, particularly in the identified metabolites, emphasizes the need for further research to understand the complex interactions between the microbiome and metabolome in patients experiencing side effects from asthma treatment. As anticipated, a reduction in the number of features leads to a decrease in the portion of variance explained by metabolomics and an increase in the importance of metagenomics. In both models, six latent factors were identified, as shown in Table 5 below. The number of factors was limited by the number of samples in the dataset.

**Table 5:** Per factor variance explained in metabolomic and metagenomic features by MOFA models, three datasets with the same 8902 metagenomics features and all 24 K metabolic features (Full data), 5 K identified metabolites (Identified metabolites) and 1200 KEGG-identified metabolites were analyzed.

Factor	Metabolomics full (%)	Metabolomics metabolites (%)	Metabolomics KEGG metabolites (%)	Metagenomics full (%)	Metagenomics metabolites (%)	Metagenomics KEGG metabolites (%)
Factor1	23	24.7	21.4	0.171	0.182	0.0875
Factor2	7.41	5.65	4.79	10	12.9	13
Factor3	14.4	13.8	17.4	0.267	0.27	0.18
Factor4	12.4	12.5	11.8	0.237	0.284	0.25
Factor5	3.25	2.93	1.56	0.839	2.53	2.69
Factor6	3.63	2.35	0.21	0.272	0.996	1.5

**Figure 1:**
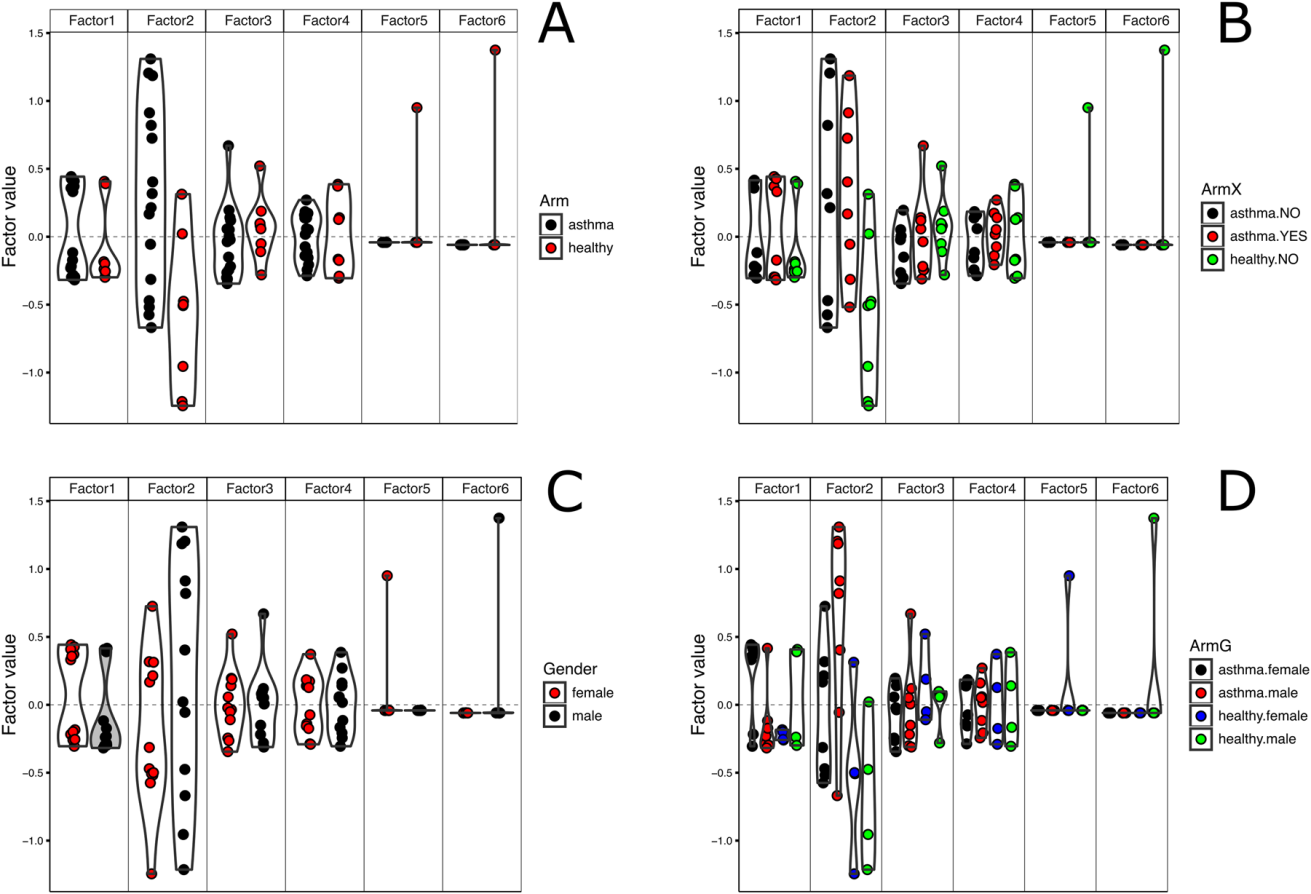
MOFA Beeswarm factor plots show the distribution of samples along their factor values. The samples are grouped and colored according to the diagnosis (A), diagnosis and side effects (B), patient gender(C) and diagnosis with patient gender (D).

We observed that Factor2 (Figure 1) is predominantly influenced by metagenomics in all three models, while the remaining factors are more representative of metabolomics data. It is also evident that Factor2 better discriminates between asthma and healthy data compared to other factors.

The analysis of the factor plot in Figure 1 shows that Factor 2, which contrary to other factors influenced more by metagenomics rather than metabolomics, better differentiates healthy and asthma patients. Moreover, that factor does not separate samples either on side effects (Figure 1B) or on patient gender (Figure 1C and D). On the other hand, Factor 1 assigns values according to a combination of diagnosis and gender (Figure 1D). It is interesting that the healthy female samples groups with asthma males, while asthma females samples groups separately. It is also clear that factors 5 and 6 are of little use because they are dominated by single healthy female and male samples correspondently. Factors 3 and 4, despite the explanation of a meaningful part of the metabolomic data variation in the model, do not show meaningful data separation along any of the annotation factors. That is why we won’t analyze factors 3 to 6 any further.

Table 6 shows how many features, which have high weight absolute value, mapped to KEGG pathways. Factor 2, despite explaining 5 % of the variability in metabolomics data, has no metabolic features with a weight above 0.1 in absolute value. However, if we select 20 features with the most negative weights two of them: N-acetylserotonin (KEGG ID C00978) Indole-3-acetaldehyde (KEGG ID C00637) will be mapped to the Trytophan pathway (KEGG ID map00380).

**Table 6:** Metabolic pathways mapped to the features with significant (0.5 value) value of the weight in MOFA model.

Factor	Pathway	KEGG ID	Sign	N features
Factor 1	Biosynthesis of unsaturated fatty acids	map01040	–	7
Factor 1	Galactose metabolism	map01100	+	3
Factor 1	ABC transporters	map02010	+	3

Factor 1 mainly deals with metabolomics data and demonstrates a good separation of samples along with diagnosis and gender annotations. Seven out of 13 metabolic features with weight below −0.5 are mapped to the Biosynthesis of unsaturated fatty acids (KEGG ID map01040): Nervonic acid (KEGG ID C08323), Arachidic acid (KEGG ID C06425), Erucic acid (KEGG ID C08316), Palmitic acid (KEGG ID C00249), Docosanoic acid (KEGG ID C08281), Eicosapentaenoate (KEGG ID C06428), Lignoceric acid (KEGG ID C08320). We discuss the implications of fatty acid biosynthesis and tryptophan metabolism together with metabolome analysis.

Like what we saw in Factor 2 for metabolomic features, we could observe in metagenomics features for Factors 1 and 3: that there are no metagenomic features with an absolute value of weight above 0.1. That corroborates the finding that Factor 2 describes the variation of metagenomics data while Factors 1, 3, and 4 describe the variation of metabolomics data with little influence across views.

### 3.3 Metabolome analysis

**Figure 2:**
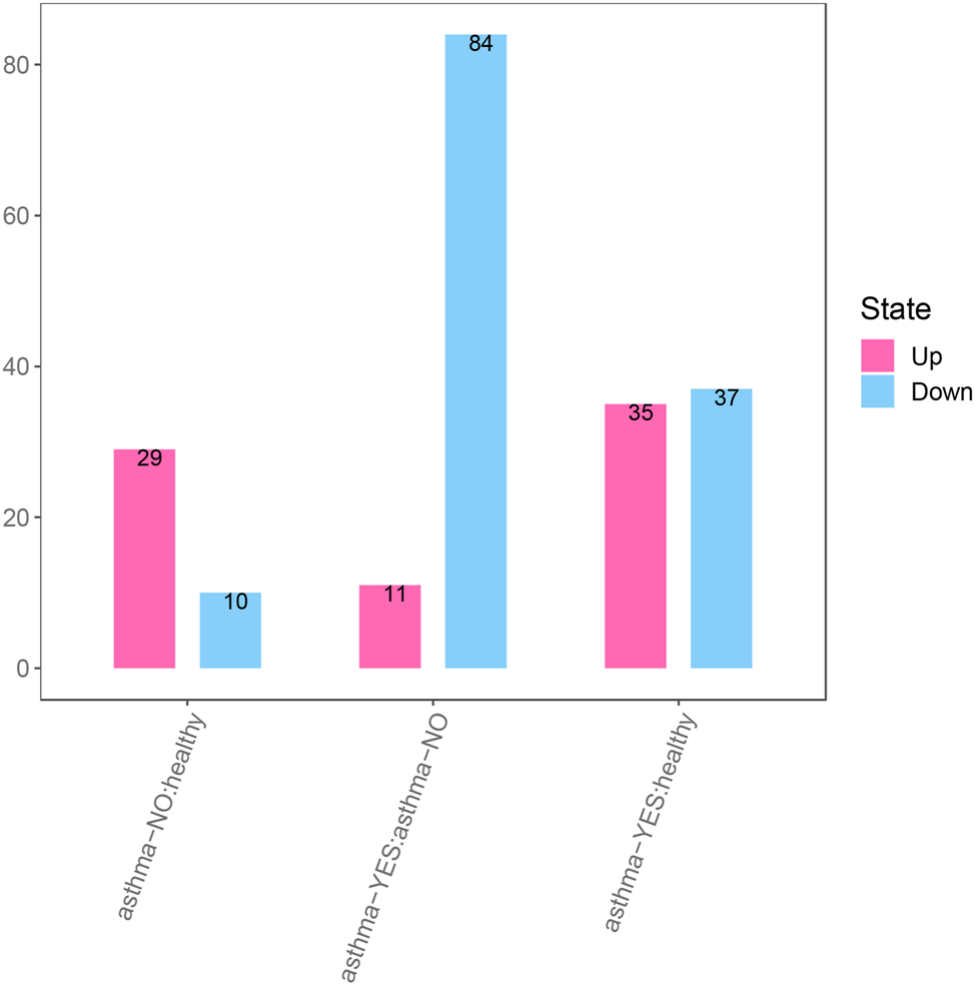
Overall changes in metabolites. Differences between three groups of patients represented: Healthy people (healthy), asthma patients with corticosteroid side effects (asthma.YES) and asthma patients without side effects (asthma.NO).

**Figure 3:**
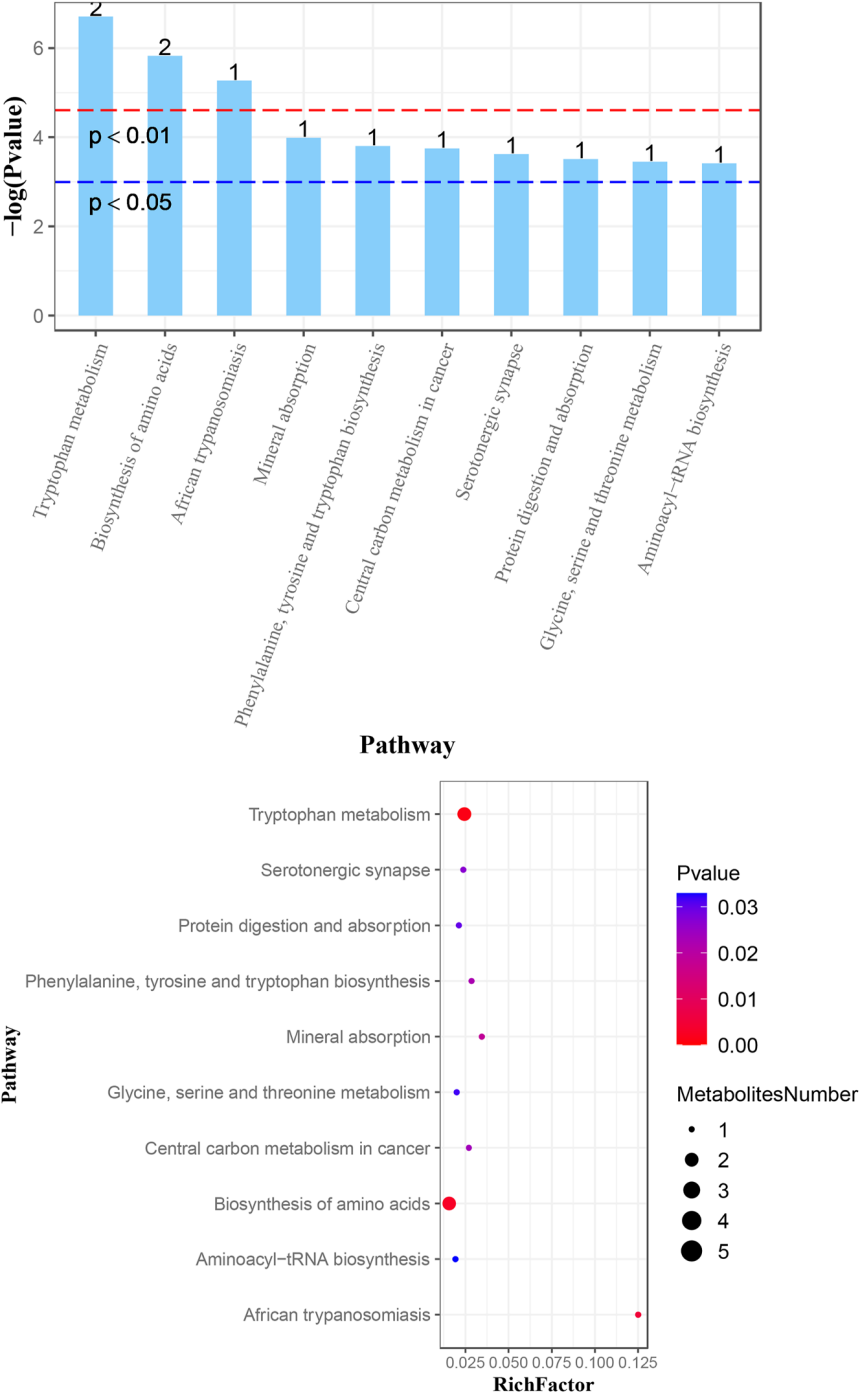
Major changes in pathways. Pairwise analysis of data from asthma patients with corticosteroid side effects vs. asthma patients without side effects.

**Figure 4:**
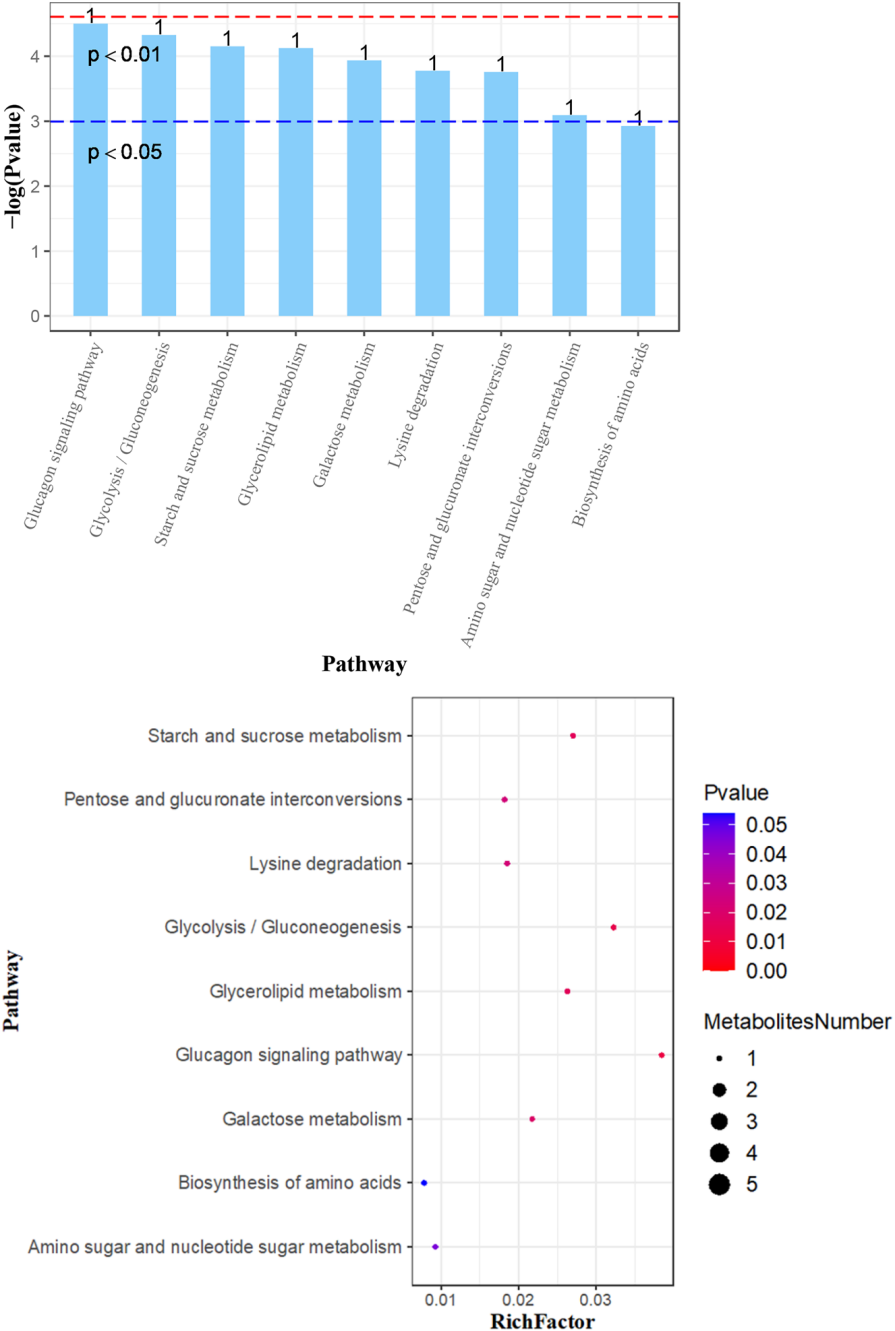
Major changes in pathways. Pairwise analysis of data from asthma patients with corticosteroid side effects vs. healthy people.

**Figure 5:**
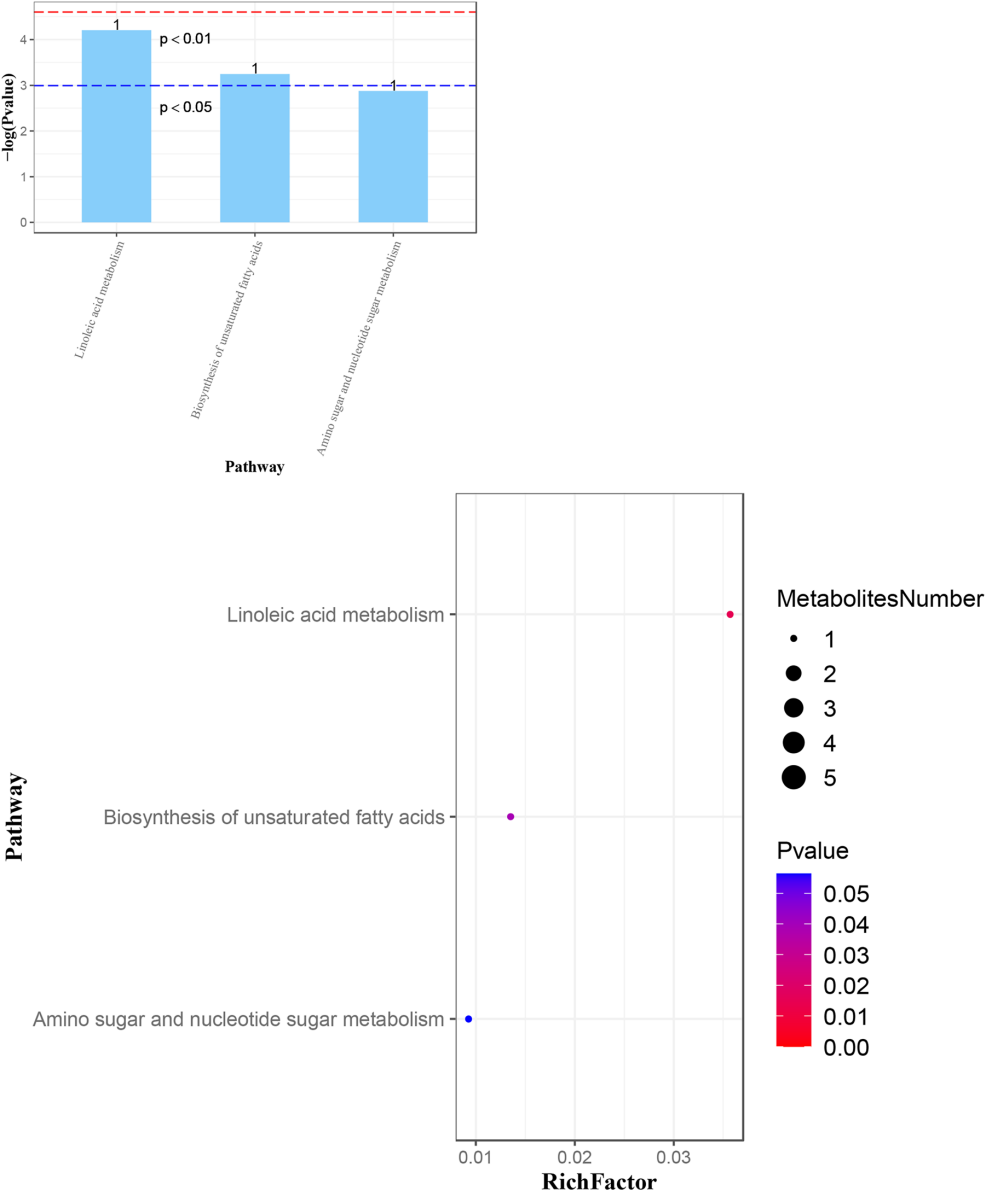
Major changes in pathways. Pairwise analysis of data from asthma patients without corticosteroid side effects vs. healthy people.

The figures below illustrate major findings in metabolomics. Figure 2 shows overall changes in metabolites between all three groups: healthy people, asthma patients with and without corticosteroid side effects side effects. Figure 3 shows which pathways are affected in comparison to data from patients with and without side effects. Figure 4 shows which pathways are affected in patients with side effects compared to healthy people. Figure 5 shows which pathways are affected in patients with no side effects compared to healthy people.

### 3.4 FBA-PRCC analysis

The structure of the microbial community is controlled by the interactions between community members and the environment. Most such interactions are mediated by small molecules called exo-metabolites, which are produced and consumed by members of the community. We applied FBA-PRCC [32] to investigate which bacteria and metabolites belong to which category. For each species in AGORA2 [48] collections of whole-genome models of human symbiotic bacteria we have calculated sensitivity coefficients linking metabolite influx/outflux and bacterial growth. By filtering out only coefficients significant at the level of 10 % we build a sensitivity graph in which 7295 bacterial strains are linked to 843 small molecules. Each edge of the graph has a weight corresponding to the partial rank-correlation coefficient between bacterial growth and metabolite availability. A negative sign of weight indicates that growth is suppressed by an increase of metabolite concentration. The direction of the edge defines either the production or the consumption of the metabolite influenced the growth.

Similar types of graphs are well studied about the protein-protein interaction (PPI) networks, where they are used to identify putative multi-subunit complexes and protein-disease relationships by property propagation from the seed set of known proteins [49]. However, PPI is usually undirected, and their edge weights are positive. Santolini and Barabasi [50] extend that type of analysis to directed graphs and signed weights in their DYNAMO algorithm. We adopted DYNAMO algorithm as it was implemented in BioNAR package [51] to evaluate the stable distribution of species and metabolite abundances in the community. The propagation algorithm simulates the mutual directional influence of species and metabolites on each other. For this we used seed abundances, for example, setting 10 most abundant species to their sample counts keep the rest as zero, and apply the propagation algorithm until convergence. The presence of negative weights could mean their extinction from the community. To avoid negative weights, we modified the propagation algorithm in a way that once a node acquires a negative weight its weight is set to zero. That modification allows the simulation of full consumption of metabolites and extinction of species.

**Figure 6:**
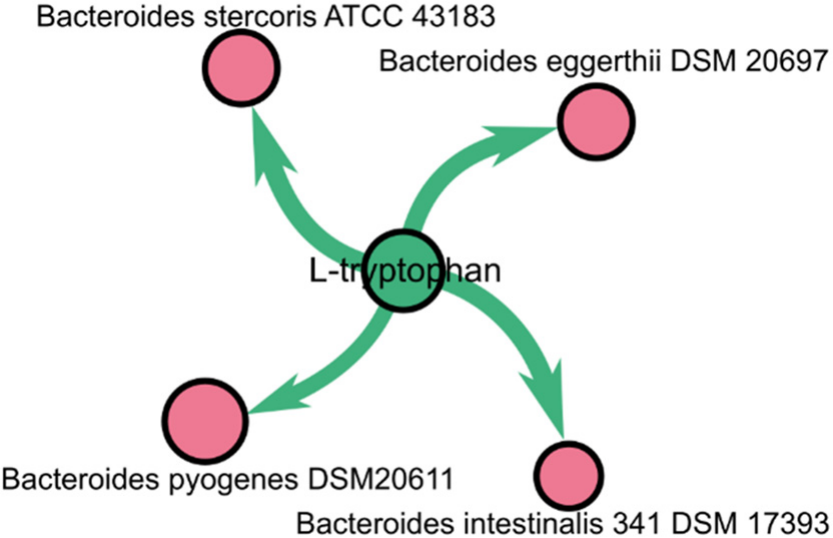
FBA-PRCC tryptophan confirmation.

We did have neither a human model in our graph nor diet information available, so we could use metabolomics data as the predefined environment and checked the structure of the steady-state distribution of the species dictated by metabolomic conditions. To compare species distribution, we used the Top-Down Concordance Coefficient (TDCC) [52] because it considers the fact that variability in high-abundance species is smaller than in low-abundant species, and so the contribution of the high-abundance species to the similarity measure should be higher. The top-down concordance coefficient (TDCC) was used to compare species weights with metagenomic abundances. Relative abundance of mapped 179 metabolites used as a “seed” set for propagation. In the FBA-PRCC graph, four Bacteroides species are significantly dependent on tryptophan. Differential abundance data shows *Bacteroides intestinalis* underabundant in asthma patients (Figure 6) supports findings. Linoleic acid is for one species *Roseburia nov* and one of the *Roseburia* genus is overabundant in asthma (Figure 6).

**Figure 7:**
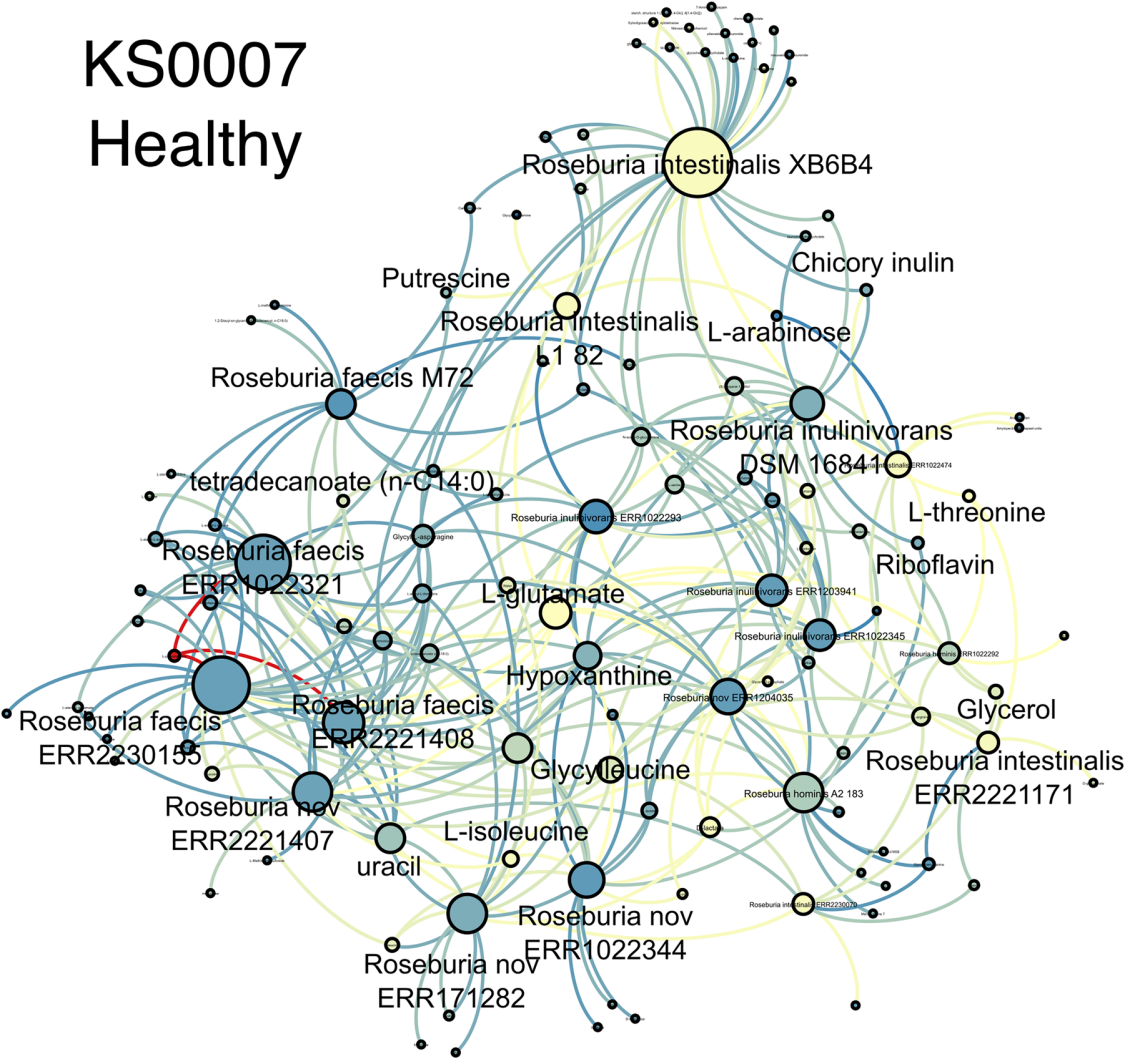
Steady-state propagation values are distributed within *Roseburia* strains. Blue is low value; yellow is medium, red is high value.

The central node, which becomes red is L-glutamate (Figure 7). Its high value is associated with Asthma.NO patients, but the number of samples is not sufficient for the rigorous statistical analysis.

## 4 Discussion

Firstly, we found that tryptophan decreased in asthma patients with side effects compared to patients without side effects. Ten metabolites from the tryptophan pathway were identified in LC/MS data, three in the negative ion registration mode and seven in the positive ion registration mode. Two of those metabolites (L-Tryptophan, Indole-3-ethanol) significantly decrease abundance in samples from asthma patients with side effects relative to samples without side effects. Metabolites from the tryptophan pathway are also found among the 20 most negatively influencing features contributing to Factor 2 of the MOFA model. That may indicate that a supplement of tryptophan could lead to a decrease in side effects manifestation. Tryptophan metabolism can be impacted by corticosteroid therapy, such as with drugs like prednisone or prednisolone, leading to decreased levels of this essential amino acid – a precursor for serotonin, melatonin, and niacin. Tryptophan is an essential amino acid, which means it cannot be synthesized by the human body and must be obtained from diet. Therefore, the observed decrease in tryptophan could be reflective of changes in dietary intake, alterations in gut microbiota, and/or the metabolic effects of the medication they are taking [53], [54], [55].

Secondly, we found that linolenate increased in asthma patients with side effects. Three metabolites from the linoleic acid pathway were identified (linoleic acid (KEGG ID C01595) Dihomo-gamma-linolenate, and 12,13-DHOME). Alpha-linolenic acid (ALA), an essential omega-3 fatty acid found in plant-based foods such as flaxseeds, chia seeds, and walnuts, has been observed. Although ALA itself is not implicated in causing these side effects, it possesses a range of health benefits, including anti-inflammatory effects that could potentially influence the inflammatory pathways affected by corticosteroid therapy [56].

Thirdly, we found that Glucose-1-phosphate (G1P) increased in asthma patients with side effects. An increase in G1P (glucose-1-phosphate) suggests alterations in glucose metabolism, which may reflect the broader metabolic effects of corticosteroid therapy. While corticosteroids are known to impact inflammation, metabolism, and immune function, G1P itself is typically involved in glycogen synthesis and breakdown within the liver and muscle tissues, playing a role in the body’s energy management [57]. The observed increase in G1P could potentially be related to corticosteroid-induced hyperglycemia, a common side effect where blood glucose levels are elevated due to the enhanced breakdown of glycogen (glycogenolysis) and increased gluconeogenesis, processes in which glucagon is also involved [58]. Furthermore, glucagon has been studied for its effects on bronchial smooth muscle relaxation, which could provide a therapeutic benefit in asthma by reducing airway hyperreactivity [59]. It has been associated with anti-inflammatory actions in models of allergic airway disease, suggesting that it may have potential therapeutic implications in reducing airway inflammation and remodeling [60].

Finally, we found that Saccharopine increase in asthma patients with side effects. An elevation of Saccharopine could indicate a disruption in amino acid metabolism, particularly within the lysine degradation pathway. Saccharopine is primarily produced by humans as part of the lysine degradation pathway, a process in the metabolism of the amino acid lysine. Saccharopine is formed during the conversion of lysine to *α*-aminoadipic acid in the mitochondria. The elevation of Saccharopine might indicate a disturbance in amino acid metabolism. Corticosteroids may indirectly affect various metabolic processes, including the biosynthesis and degradation of amino acids such as lysine, although they do not directly target these pathways [61], 62]. Specifically, Saccharopine is synthesized from lysine and *α*-ketoglutarate. Disruptions in this pathway may lead to mitochondrial stress and could potentially influence the development and exacerbation of asthma [63]. Moreover, Saccharopine has been studied as a marker in asthma [64].

The exploration of microbiomes and metabolome is shedding light on the potential side effects and effectiveness of corticosteroid therapies [65]. The gut microbiota’s role in drug metabolism is significant, particularly in how it affects the pharmacokinetics of corticosteroids, altering the outcomes of treatments like Enerzair, and other advanced therapies [66]. In the realm of chronic respiratory diseases such as asthma and COPD, ICS remain a cornerstone of treatment. Current research also include understanding ICS interaction with the gut microbiome, which may modify drug responses and side effect profiles [67].

Tryptophan, linolenate, G1P, Saccharopine play an **active role** in the manifestation of corticosteroid side effects by affecting mood, inflammation, immunity, and microbiome function. Thus, correcting tryptophan levels (e.g., through diet or probiotic interventions) could **reduce the burden of side effects**, making this a promising avenue for personalized asthma therapy.

Dietary interventions, especially those that boost tryptophan intake, can play a pivotal role in mitigating the neuropsychiatric side effects linked to corticosteroid use by enhancing the synthesis of serotonin and melatonin [68]. To increase tryptophan levels effectively, it’s also important to include carbohydrates. Carbohydrates can help make tryptophan more available to the brain [69]. Balanced tryptophan-rich foods can elevate tryptophan availability for serotonin and melatonin synthesis, potentially improving mood and sleep disrupted by corticosteroid use [70], 71]. The changing of diet could be tested in a follow up large scale clinical study.

The modest sample size of 24 participants represents a significant limitation, which may reduce the statistical power of the results and limit the generalizability of the findings. Additionally, the study focused on a single therapeutic intervention (inhaled corticosteroids) without accounting for other potential confounding factors, such as diet, environmental influences, or concurrent medications. Larger-scale studies are needed to validate these findings, confirm the observed microbiome and metabolome interactions, and fully explore the therapeutic potential of personalized interventions for asthma patients.

Further studies are needed to build on these preliminary observations by examining larger patient groups, which will help solidify the conclusions about drug-microbiome interactions. The development of models that can distinguish between the metabolic contributions of the host and those of the microbiota will be a critical step forward in this field [72]. Collaborative efforts with pharmaceutical companies could expedite the development of such models, integrating microbiome insights into drug development and potentially diminishing unwanted effects while enhancing drug efficacy [73], 74].

## Supplementary Material

Supplementary Material Details

Supplementary Material Details

Supplementary Material Details

Supplementary Material Details

## Abbreviations

ICS: inhaled corticosteroids
MOFA: Multi-Omics Factor Analysis
NGS: next generation sequencing
LC-MS/MS: liquid chromatography-tandem mass spectrometry
GSA-PRCC: Global Sensitivity Analysis-Partial Rank Correlation Coefficient;
COPD: chronic obstructive pulmonary disease
FBA-PRCC: Flux Balance Analysis Partial Rank Correlation Coefficient
ASAR: Advanced metagenomic Sequence Analysis in R
PQN: Probabilistic Quotient Normalization
QC-RLSC: Quality Control-based Robust LOESS
QC: quality control
SCFA: short-chain fatty acids
