# Supplementary material for: Metagenome and metabolome study on inhaled corticosteroids in asthma patients with side effects
Source: J Integr Bioinform. 2025 Jun 24;22(3):20240062. doi: 10.1515/jib-2024-0062 (PMC13066347; doi:10.1515/jib-2024-0062)
Supplement: Supplementary file 2 — Supplementary Material Details [file j_jib-2024-0062_suppl_002.pdf]

Kyrgyz asthma metagenomic analysis

A. Sorokin

04.03.2024

1 Full metabolic model

1.1 Read model

1.2 Variance explained

metabolomics	metagenomics
63.5	11.8

	metabolomics	metagenomics
Factor1	23	0.171
Factor2	7.41	10
Factor3	14.4	0.267
Factor4	12.4	0.237
Factor5	3.25	0.839
Factor6	3.63	0.272

1.3 Factor Plots

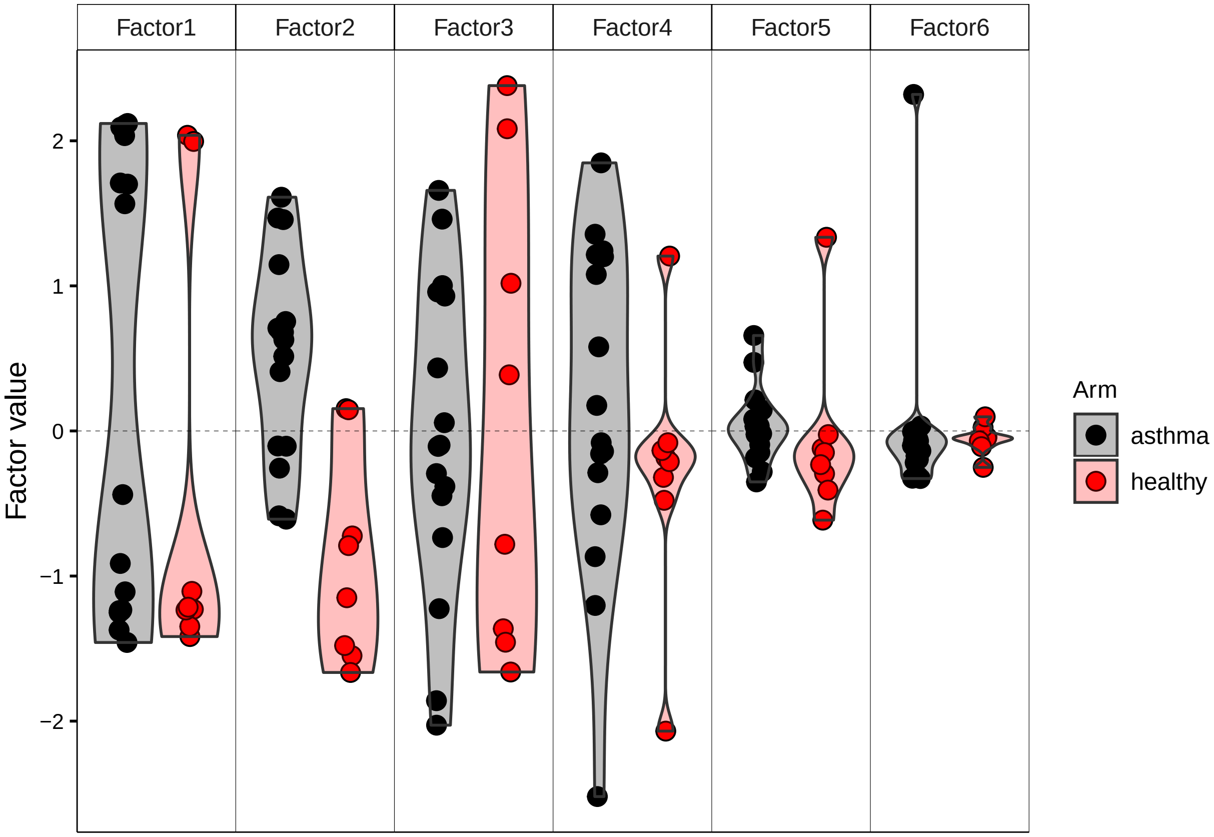

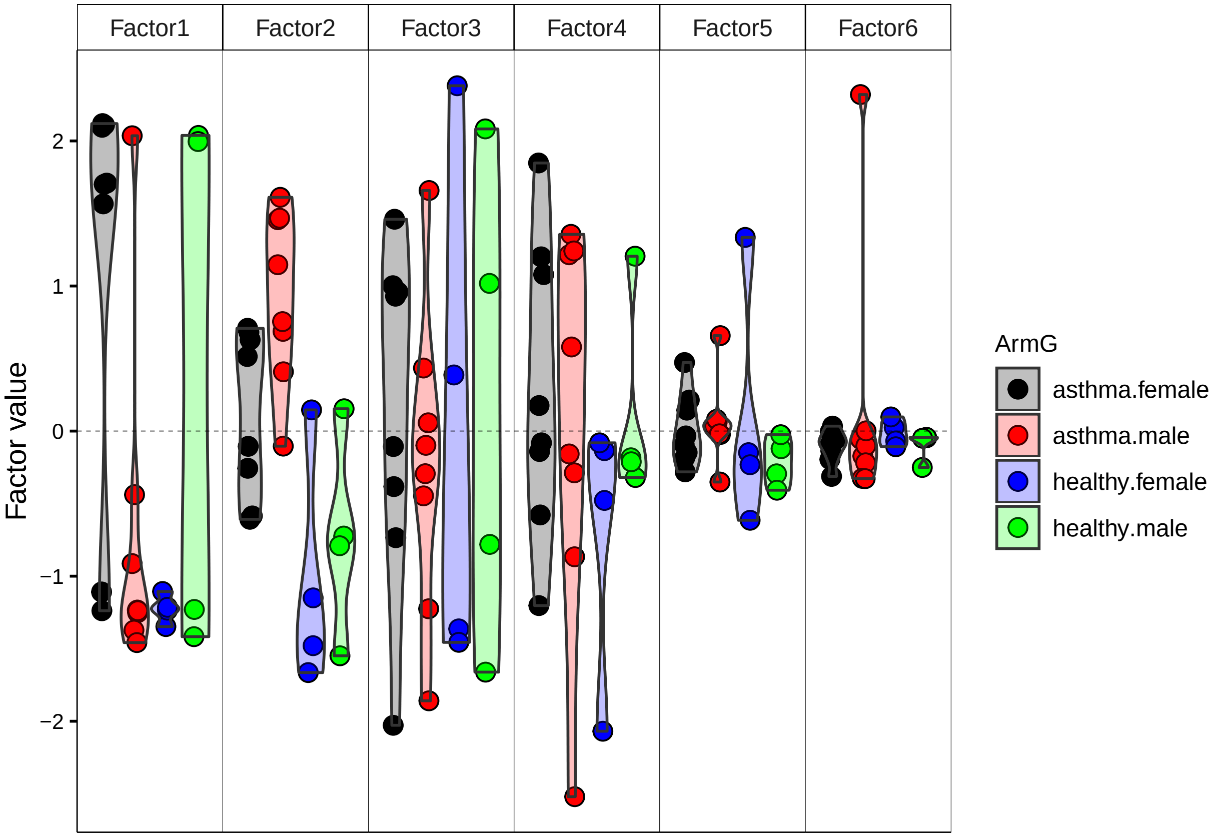

Factor1
Factor2
Factor3
Factor4
Factor5
Factor6
Factor value
2
1
0
−1
−2
ArmG
asthma.female
asthma.male
healthy.female
healthy.male

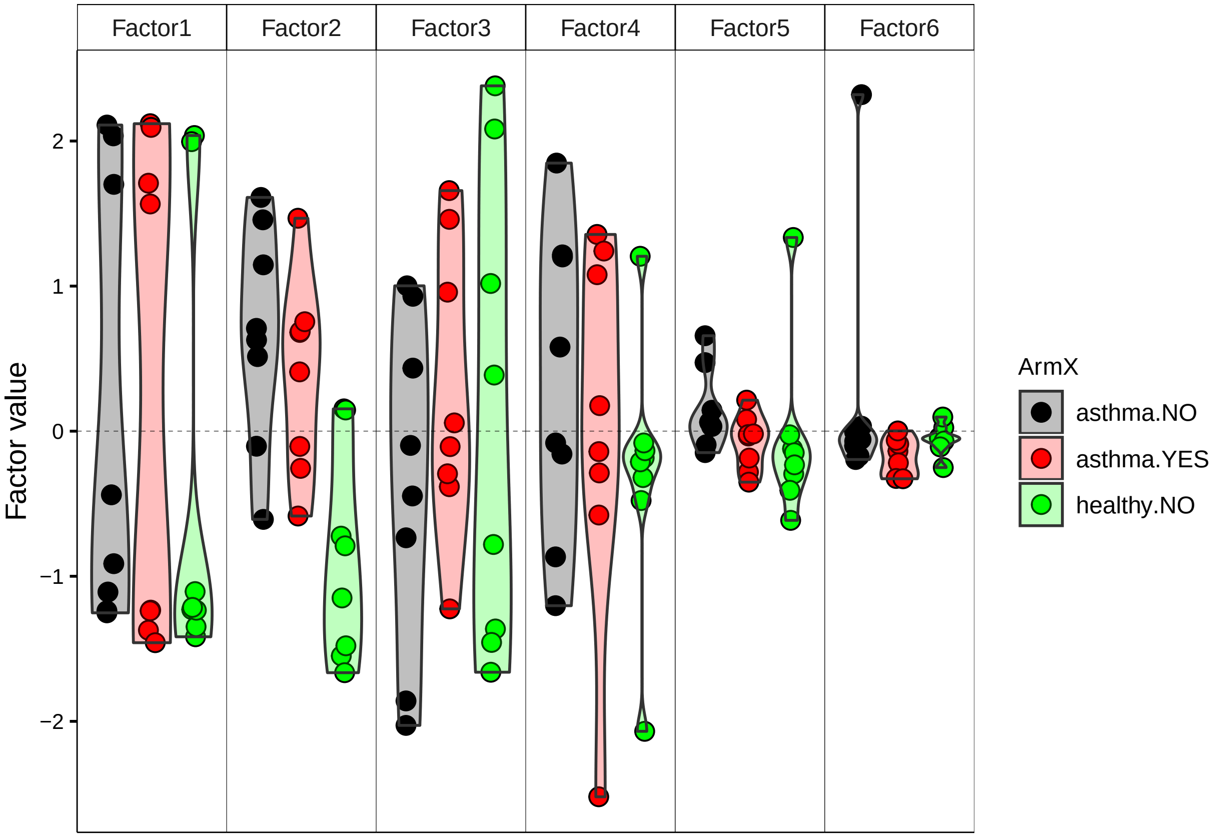

Factor1
Factor2
Factor3
Factor4
Factor5
Factor6
Factor value
2
1
0
−1
−2
ArmX
asthma.NO
asthma.YES
healthy.NO

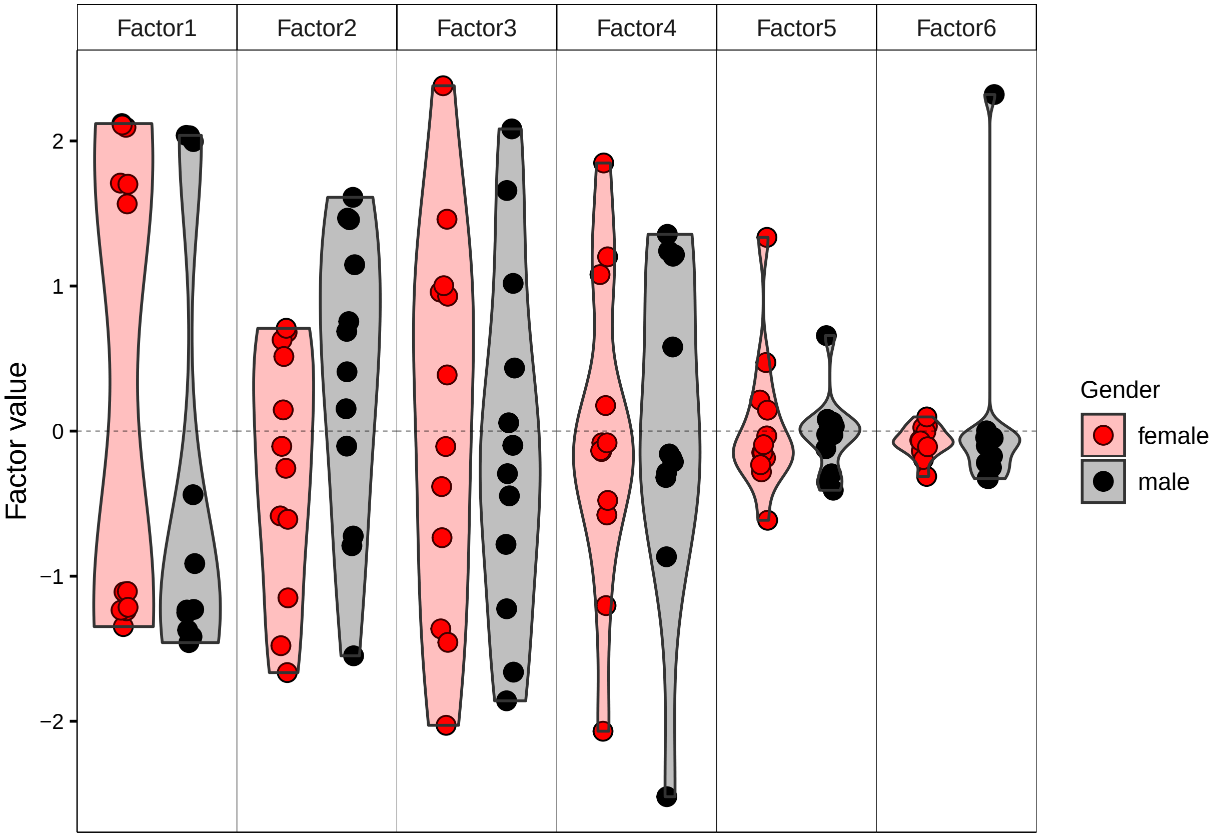

Factor1
Factor2
Factor3
Factor4
Factor5
Factor6
Factor value
2
1
0
−1
−2
Gender
female
male

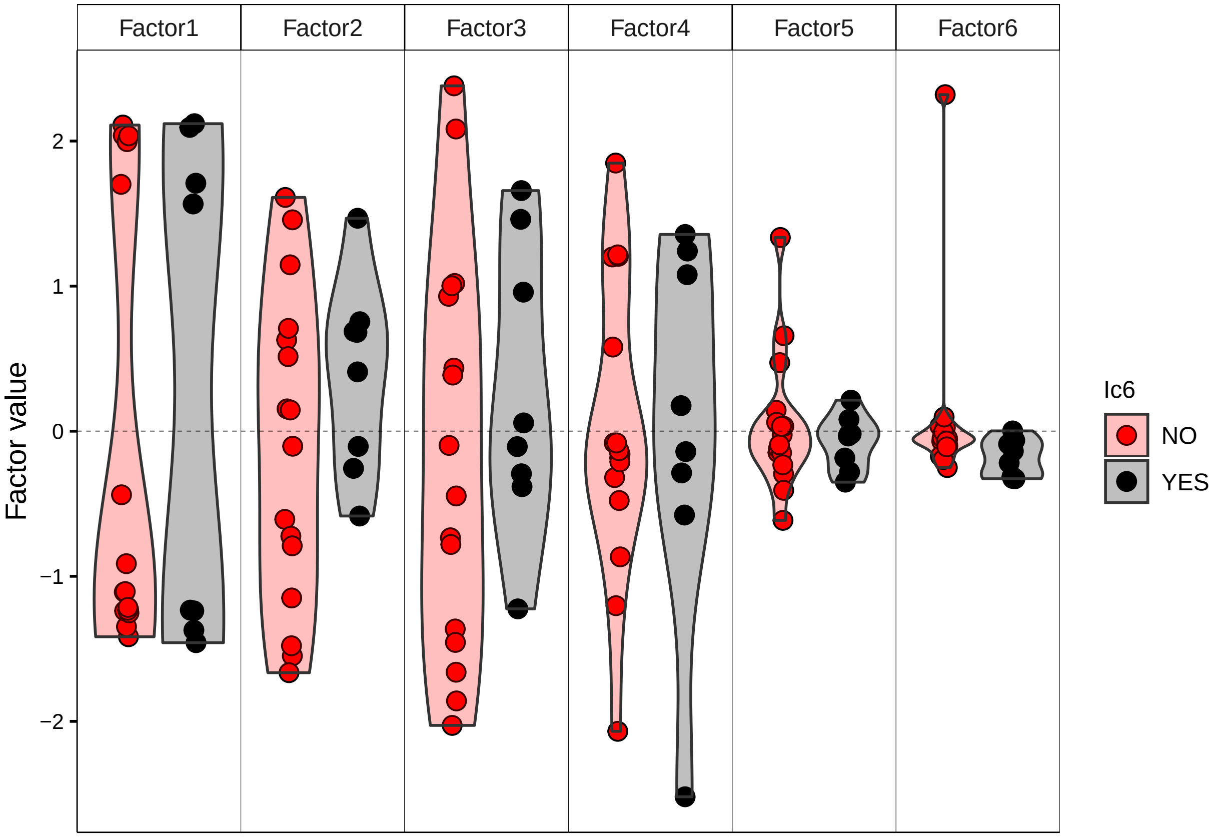

Factor1
Factor2
Factor3
Factor4
Factor5
Factor6
Factor value
2
1
0
−1
−2
lc6
NO
YES

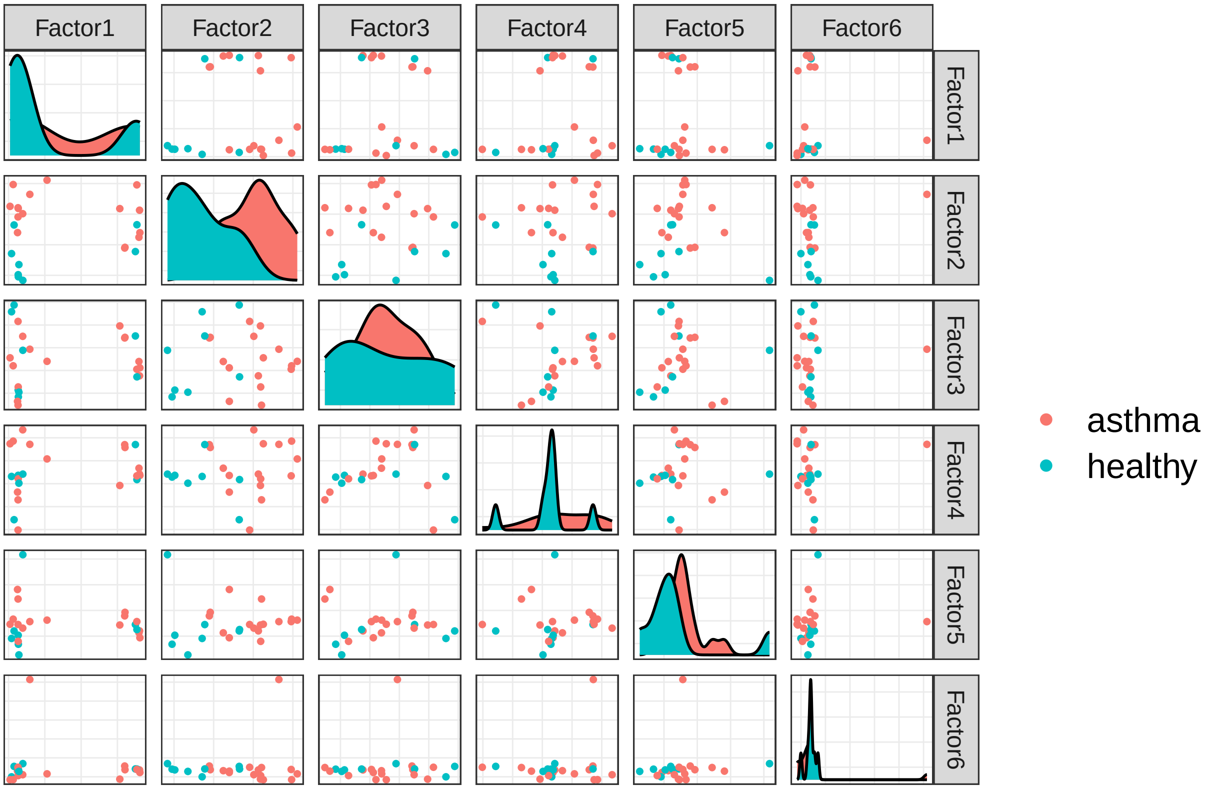

1.4 Factor matrix plot

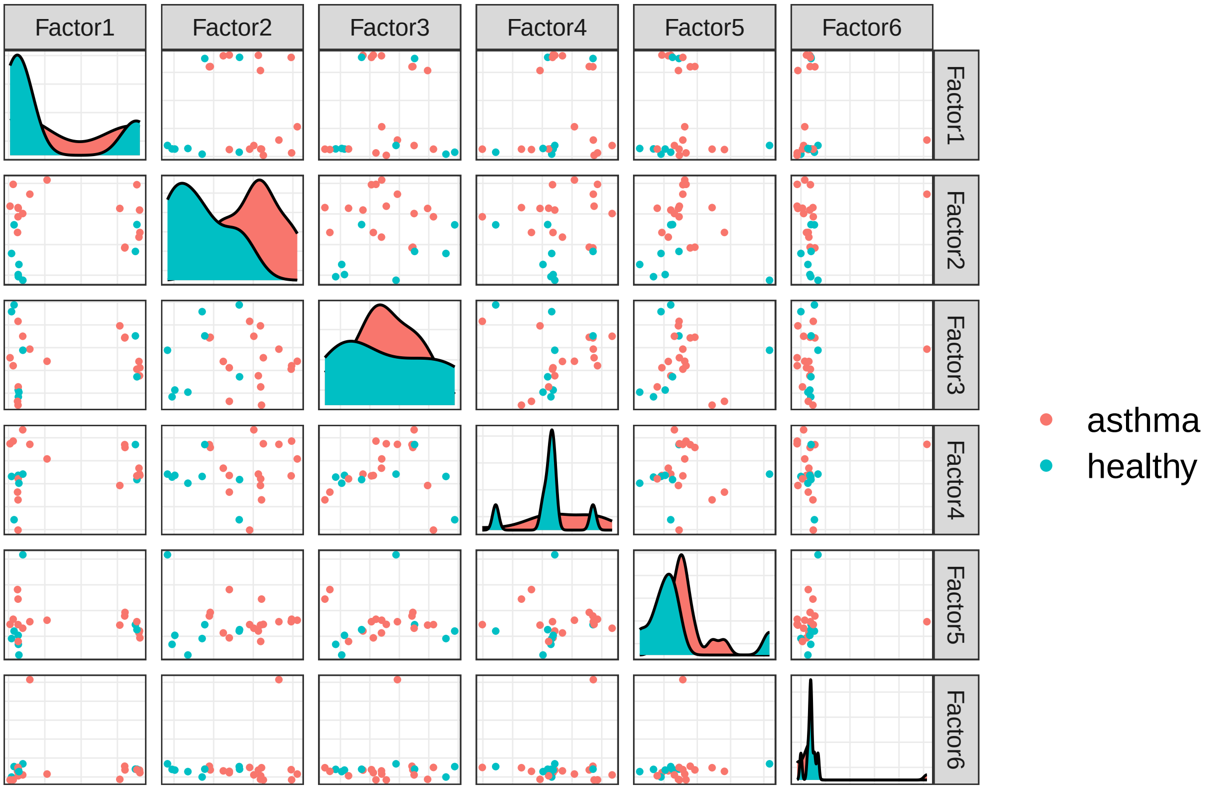

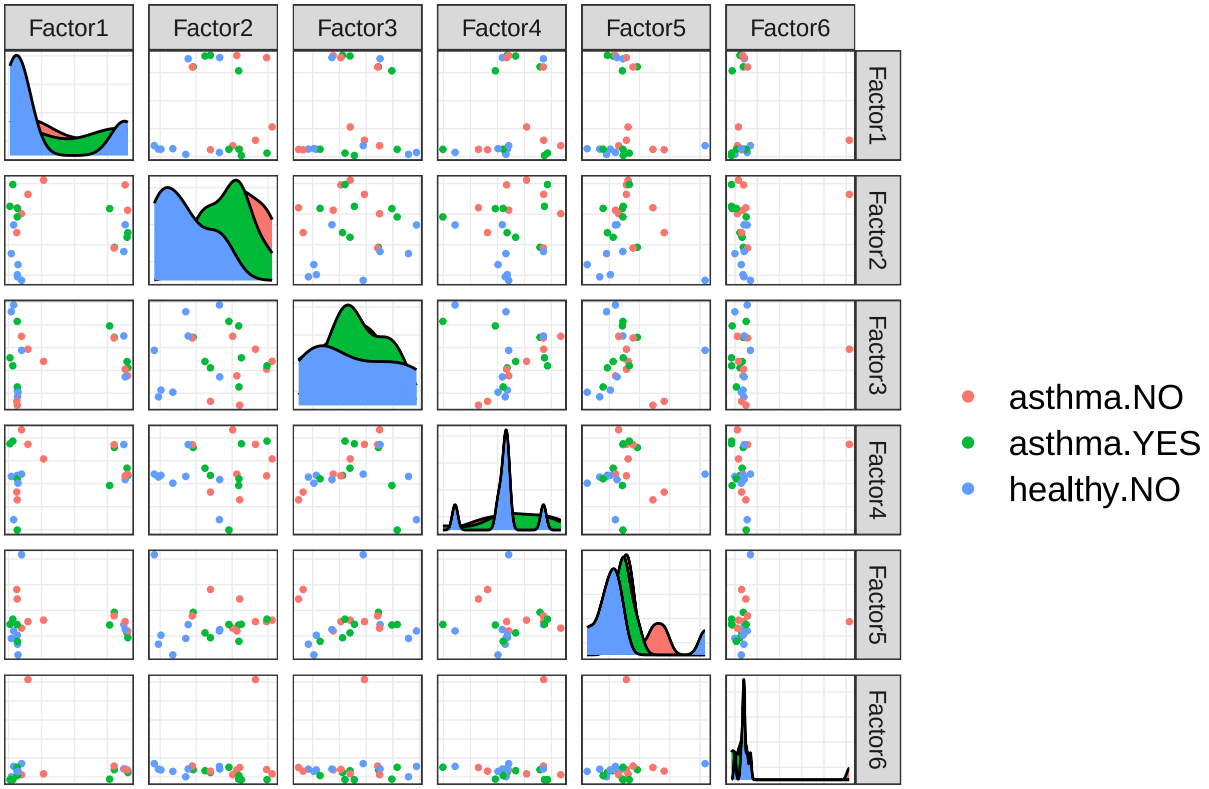
Factor1
Factor2
Factor3
Factor4
Factor5
Factor6
asthma.NO
asthma.YES
healthy.NO

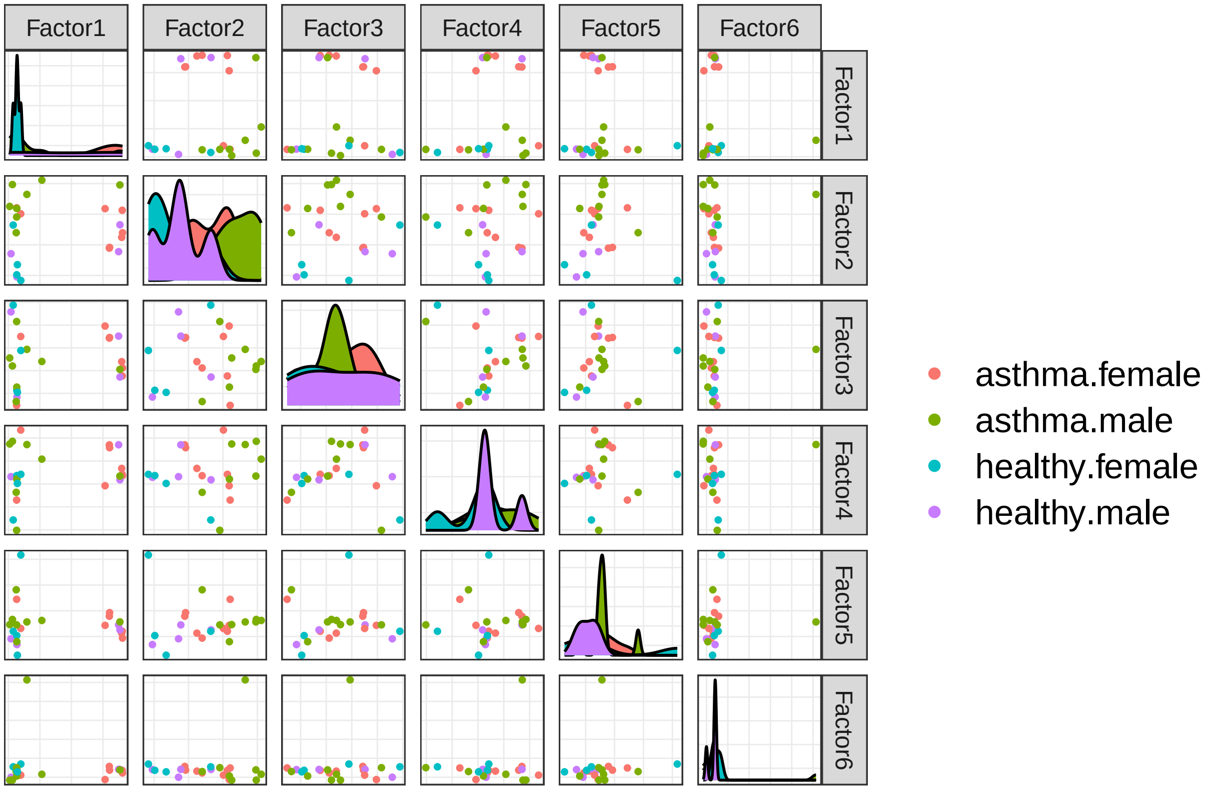
Factor1
Factor2
Factor3
Factor4
Factor5
Factor6
asthma.female
asthma.male
healthy.female
healthy.male

1.5 Plot weights

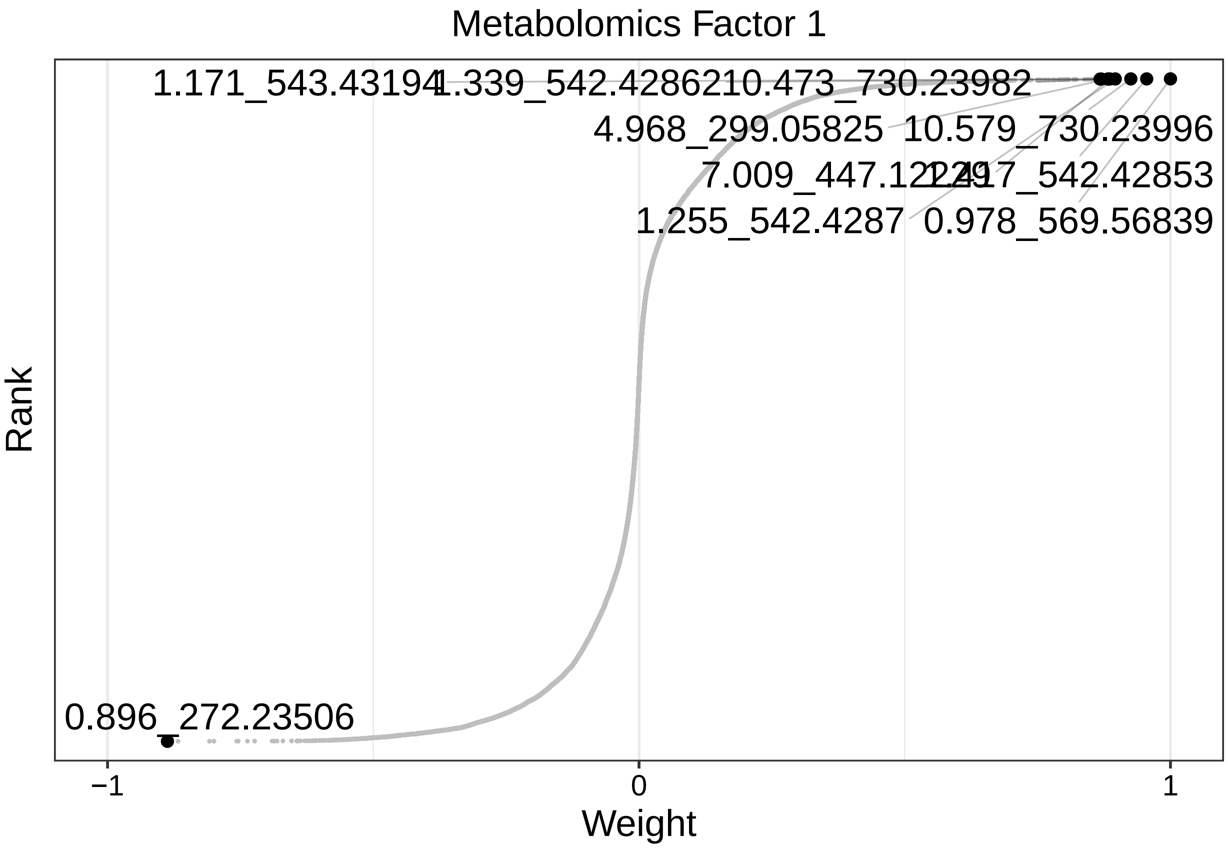

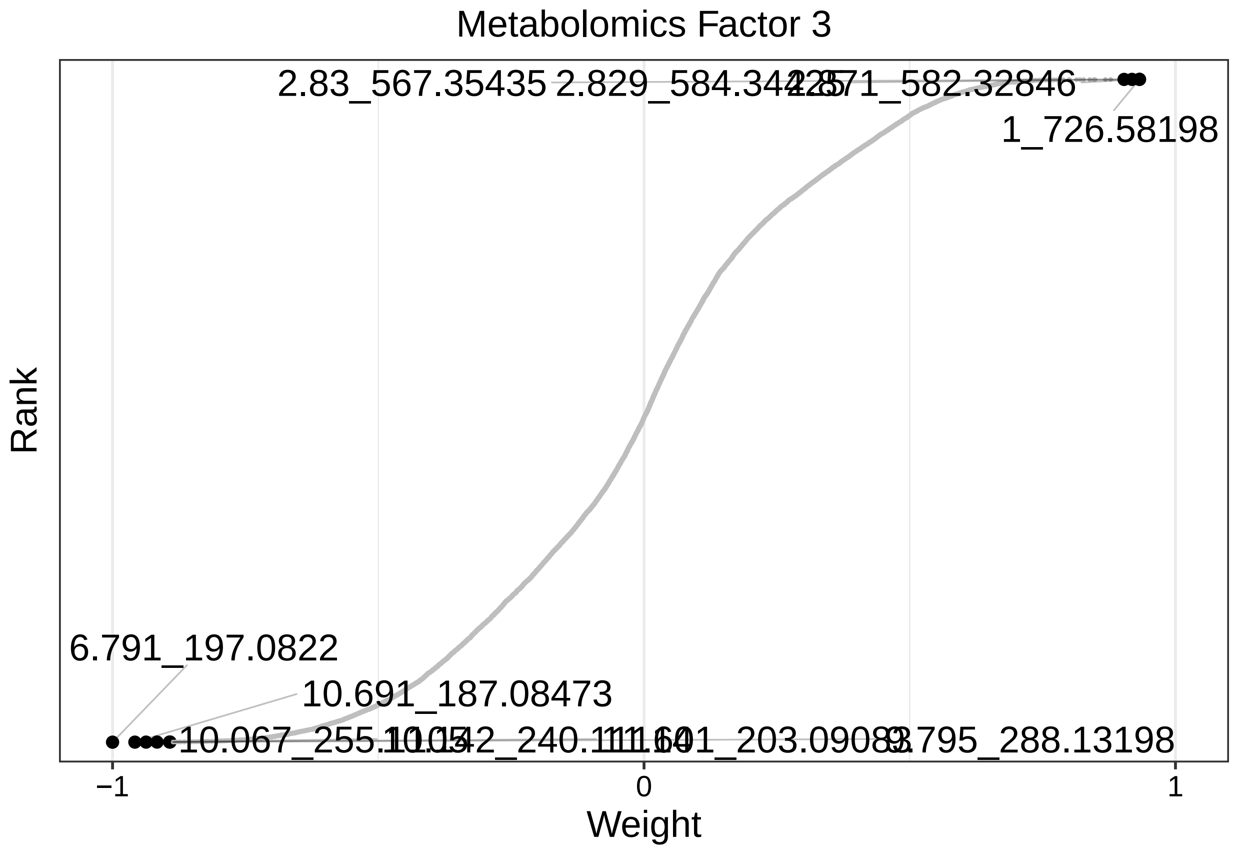
Metabolomics Factor 3
2.83_567.35435
1_726.58198
6.791_197.0822
10.691_187.08473
Rank
Weight
−1
0
1

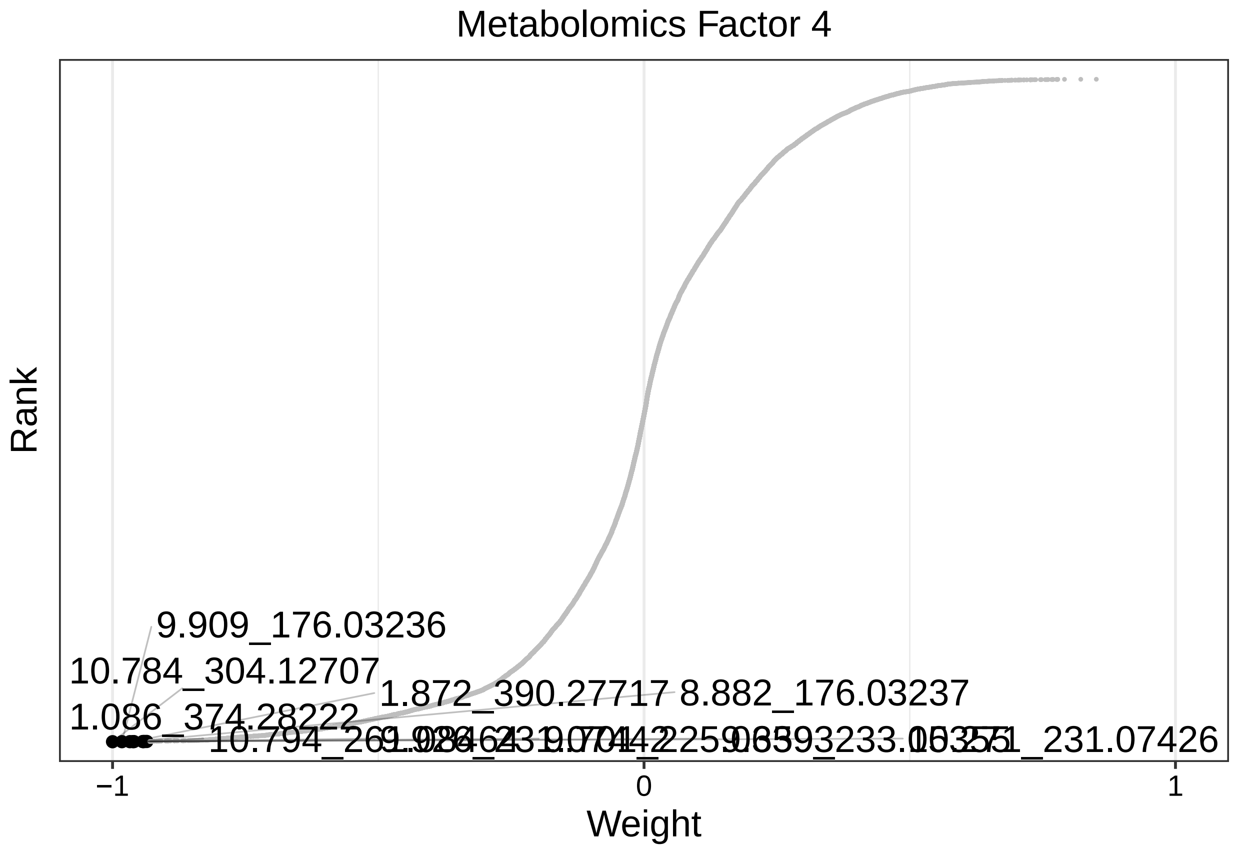
Metabolomics Factor 4
9.909_176.03236
10.784_304.12707
1.872_390.27717
8.882_176.03237
Rank
Weight
−1
0
1

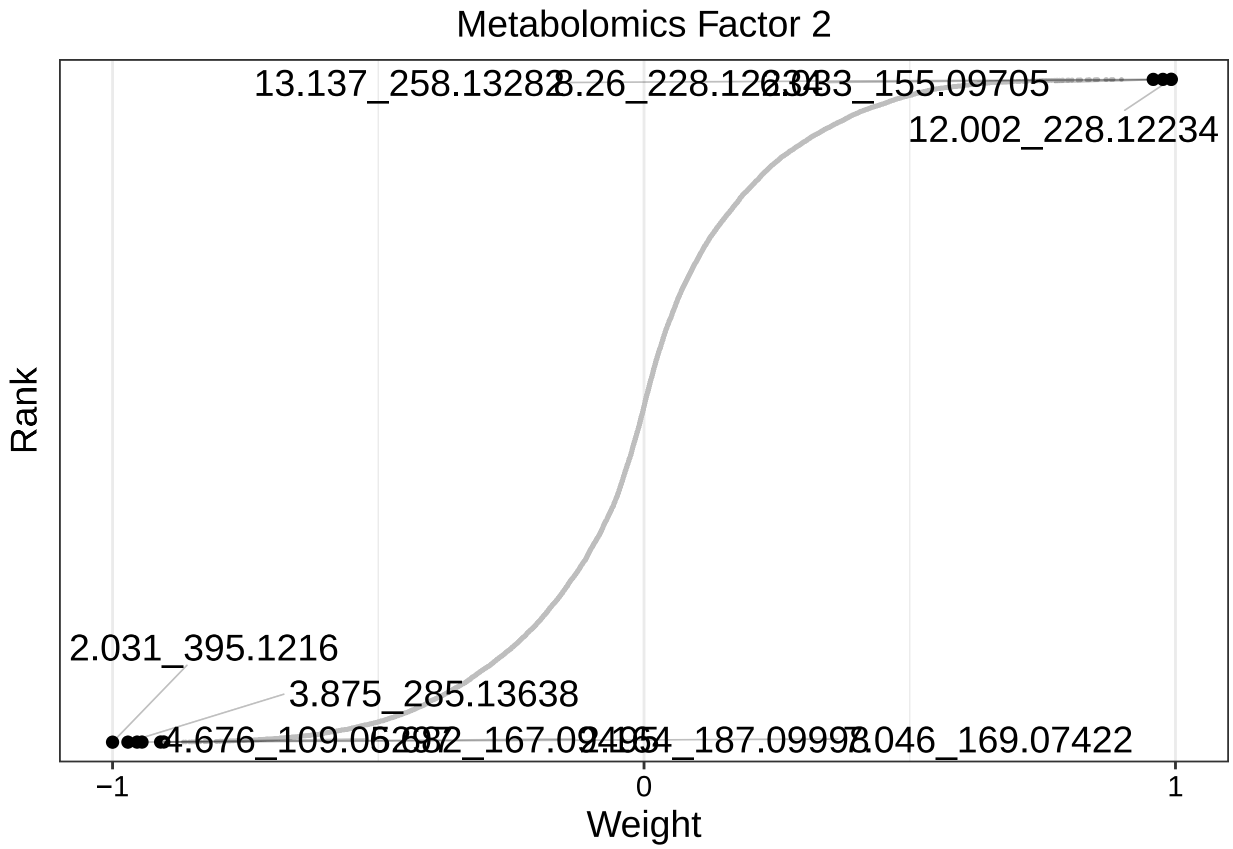
Metabolomics Factor 2
12.002_228.12234
2.031_395.1216
3.875_285.13638
Rank
−1
0
1
Weight

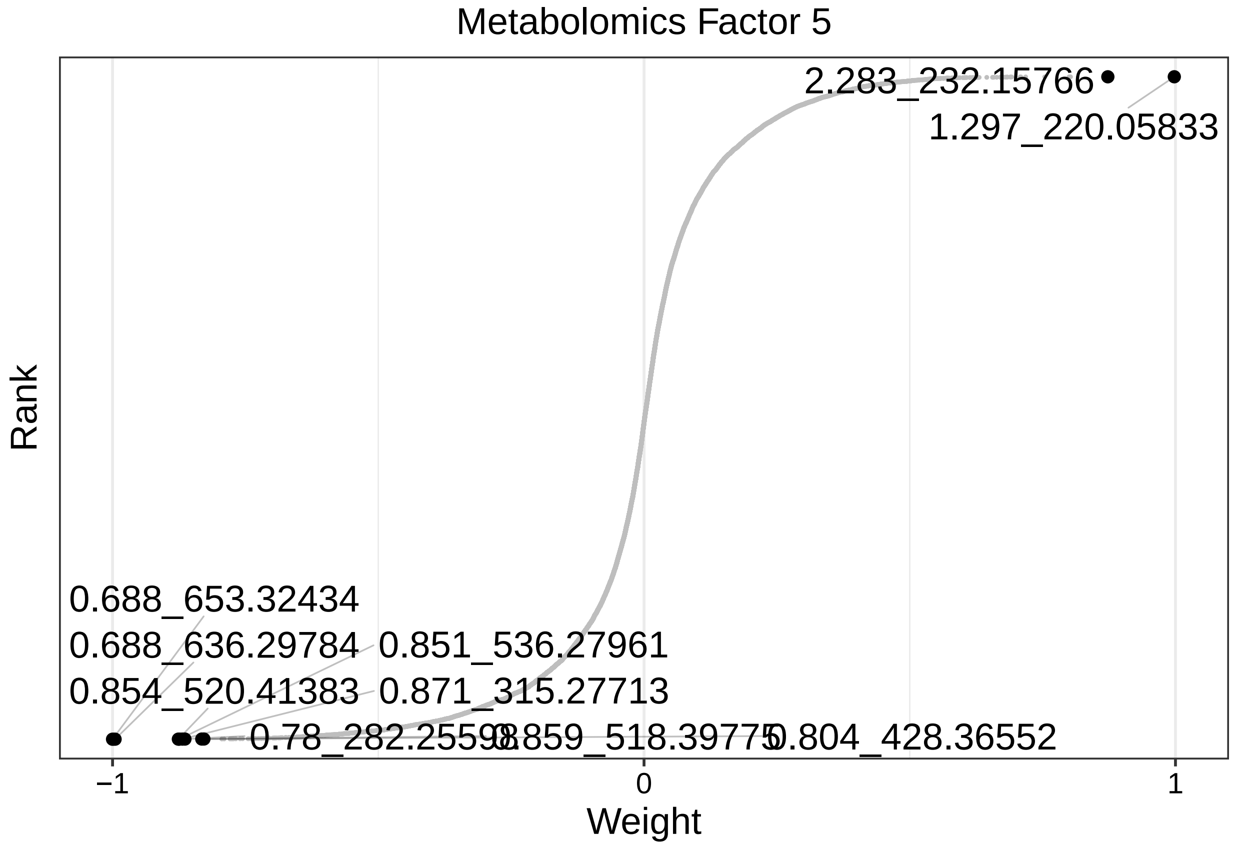
Metabolomics Factor 5
2.283_232.15766
1.297_220.05833
0.688_653.32434
0.688_636.29784
0.851_536.27961
0.854_520.41383
0.871_315.27713
0.804_428.36552
Rank
−1
0
1
Weight

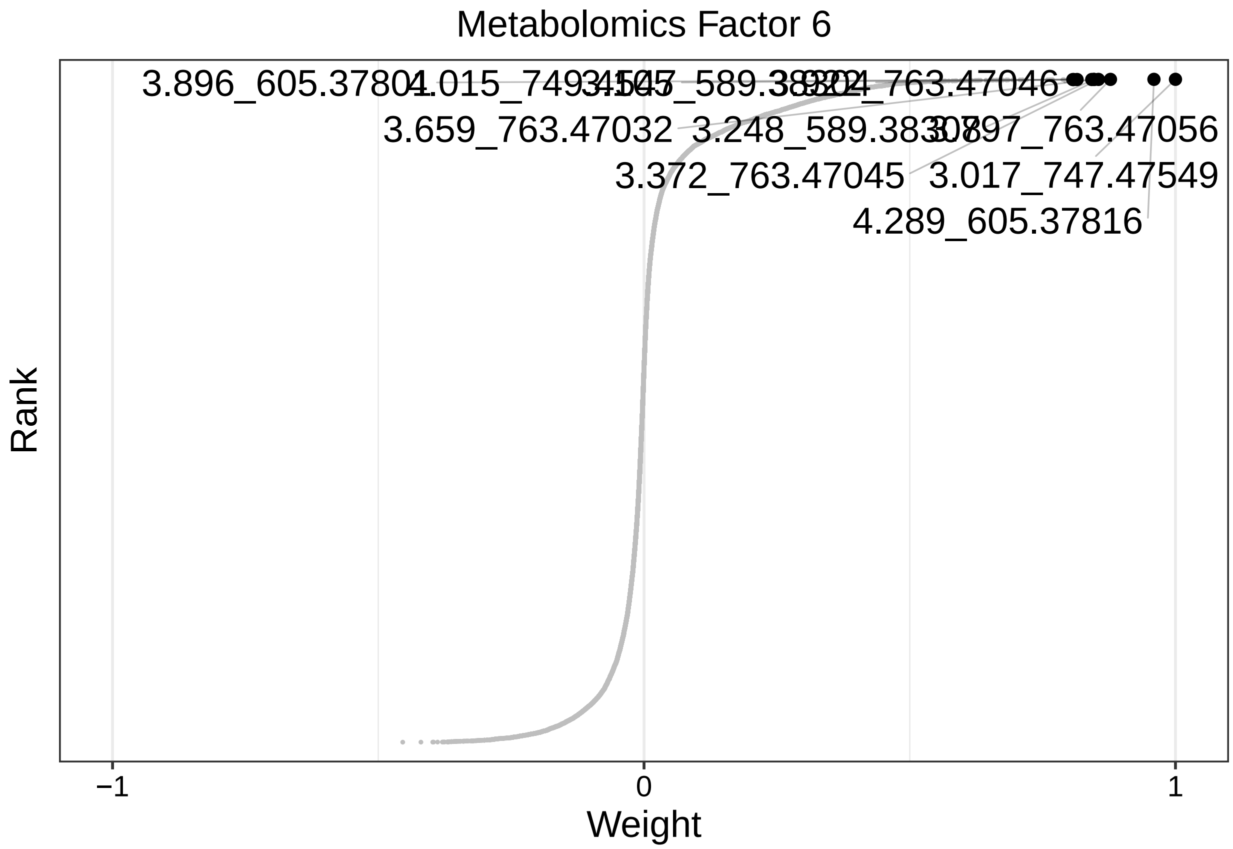
Metabolomics Factor 6
3.659_763.47032
3.372_763.47045
3.017_747.47549
4.289_605.37816
Rank
Weight
−1
0
1

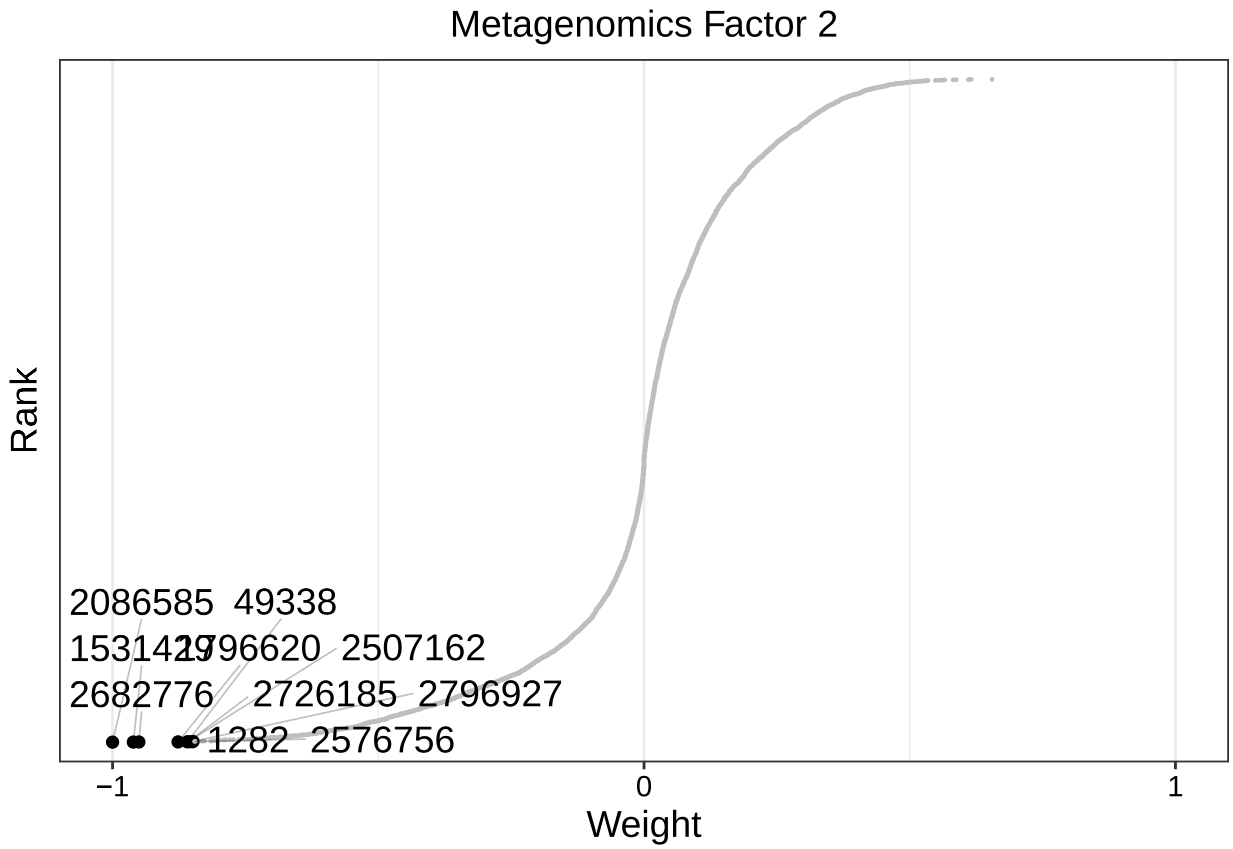
Metagenomics Factor 2
2086585
49338
2507162
2682776
2726185
2796927
2576756
Rank
Weight
−1
0
1

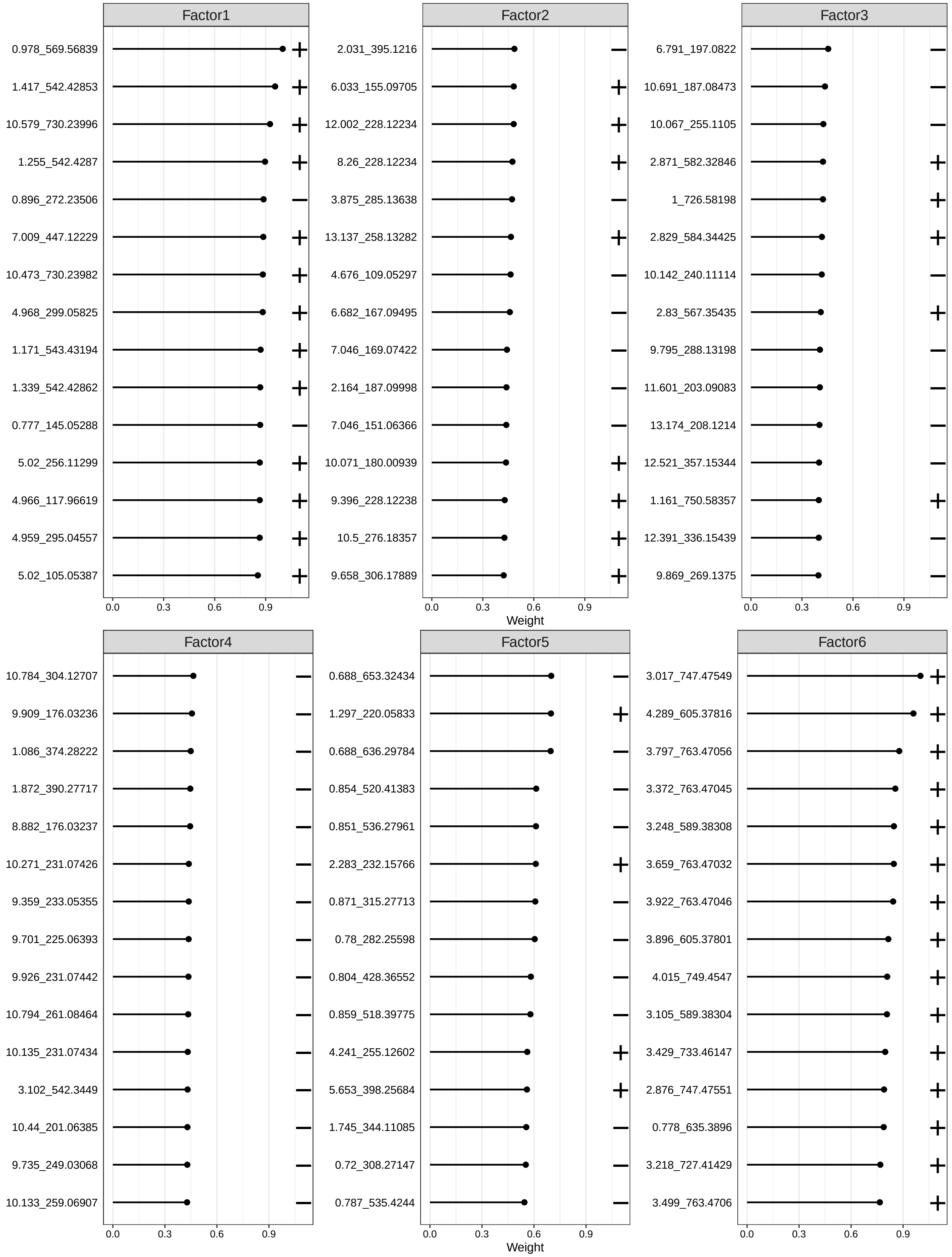
Factor1
0.978_569.56839
1.417_542.42853
10.579_730.23996
1.255_542.4287
0.896_272.23506
7.009_447.12229
10.473_730.23982
4.968_299.05825
1.171_543.43194
1.339_542.42862
0.777_145.05288
5.02_256.11299
4.966_117.96619
4.959_295.04557
5.02_105.05387
Factor2
2.031_395.1216
6.033_155.09705
12.002_228.12234
8.26_228.12234
3.875_285.13638
13.137_258.13282
4.676_109.05297
6.682_167.09495
7.046_169.07422
2.164_187.09998
7.046_151.06366
10.071_180.00939
9.396_228.12238
10.5_276.18357
9.658_306.17889
Factor3
6.791_197.0822
10.691_187.08473
10.067_255.1105
2.871_582.32846
1_726.58198
2.829_584.34425
10.142_240.11114
2.83_567.35435
9.795_288.13198
11.601_203.09083
13.174_208.1214
12.521_357.15344
1.161_750.58357
12.391_336.15439
9.869_269.1375
0.0
0.3
0.6
0.9
Weight
Factor4
10.784_304.12707
9.909_176.03236
1.086_374.28222
1.872_390.27717
8.882_176.03237
10.271_231.07426
9.359_233.05355
9.701_225.06393
9.926_231.07442
10.794_261.08464
10.135_231.07434
3.102_542.3449
10.44_201.06385
9.735_249.03068
10.133_259.06907
Factor5
0.688_653.32434
1.297_220.05833
0.688_636.29784
0.854_520.41383
0.851_536.27961
2.283_232.15766
0.871_315.27713
0.78_282.25598
0.804_428.36552
0.859_518.39775
4.241_255.12602
5.653_398.25684
1.745_344.11085
0.72_308.27147
0.787_535.4244
Factor6
3.017_747.47549
4.289_605.37816
3.797_763.47056
3.372_763.47045
3.248_589.38308
3.659_763.47032
3.922_763.47046
3.896_605.37801
4.015_749.4547
3.105_589.38304
3.429_733.46147
2.876_747.47551
0.778_635.3896
3.218_727.41429
3.499_763.4706
0.0
0.3
0.6
0.9
Weight

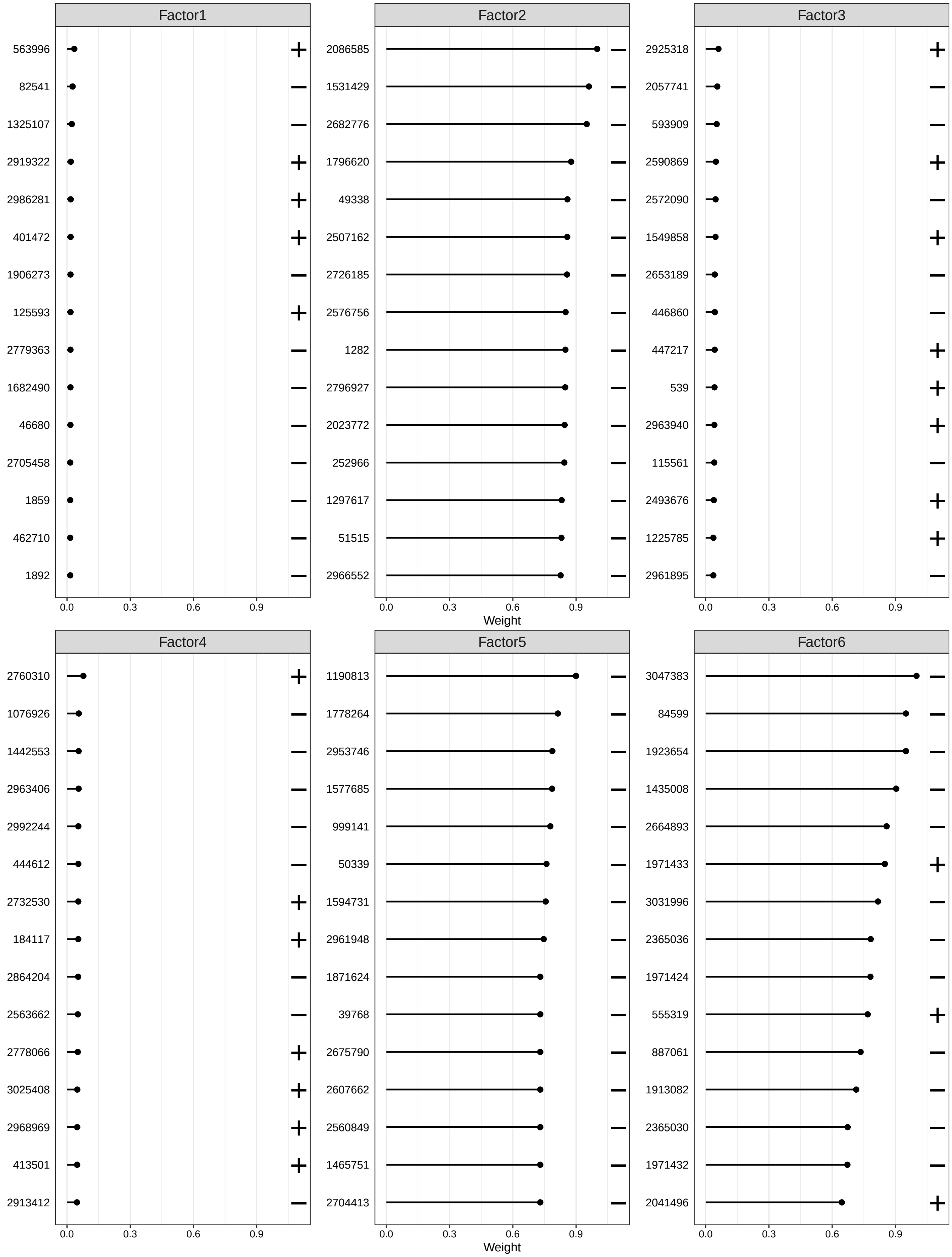
Factor1
563996
82541
1325107
2919322
2986281
401472
1906273
125593
2779363
1682490
46680
2705458
1859
462710
1892
Factor2
2086585
1531429
2682776
1796620
49338
2507162
2726185
2576756
1282
2796927
2023772
252966
1297617
51515
2966552
Factor3
2925318
2057741
593909
2590869
2572090
1549858
2653189
446860
447217
539
2963940
115561
2493676
1225785
2961895
Factor4
2760310
1076926
1442553
2963406
2992244
444612
2732530
184117
2864204
2563662
2778066
3025408
2968969
413501
2913412
Factor5
1190813
1778264
2953746
1577685
999141
50339
1594731
2961948
1871624
39768
2675790
2607662
2560849
1465751
2704413
Factor6
3047383
84599
1923654
1435008
2664893
1971433
3031996
2365036
1971424
555319
887061
1913082
2365030
1971432
2041496
0.0
0.3
0.6
0.9
Weight

1.6 UMAP projection

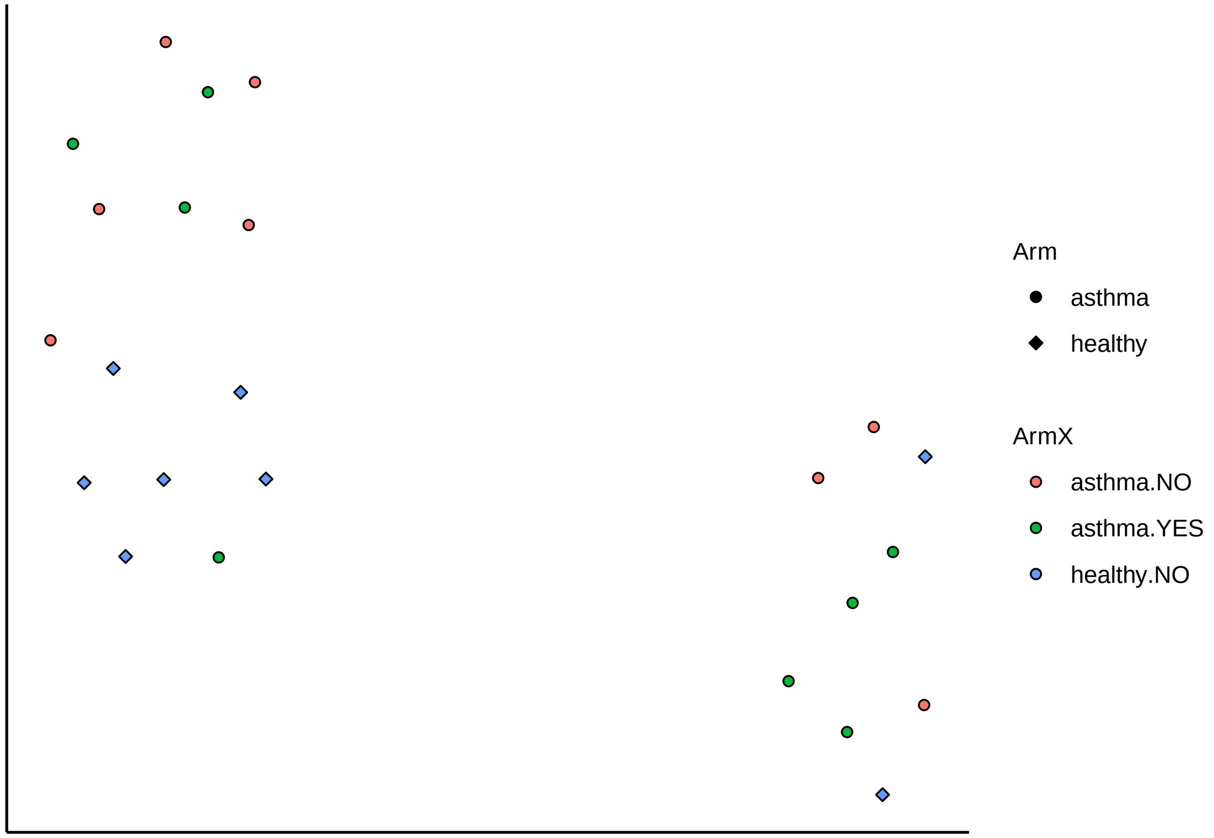

2 Identified metabolites only model

2.1 Read model

2.2 Variance explained

metabolomics	metagenomics
61.2	17.1

	metabolomics	metagenomics
Factor1	24.7	0.182
Factor2	5.65	12.9
Factor3	13.8	0.27
Factor4	12.5	0.284
Factor5	2.93	2.53
Factor6	2.35	0.996

2.3 Factor Plots

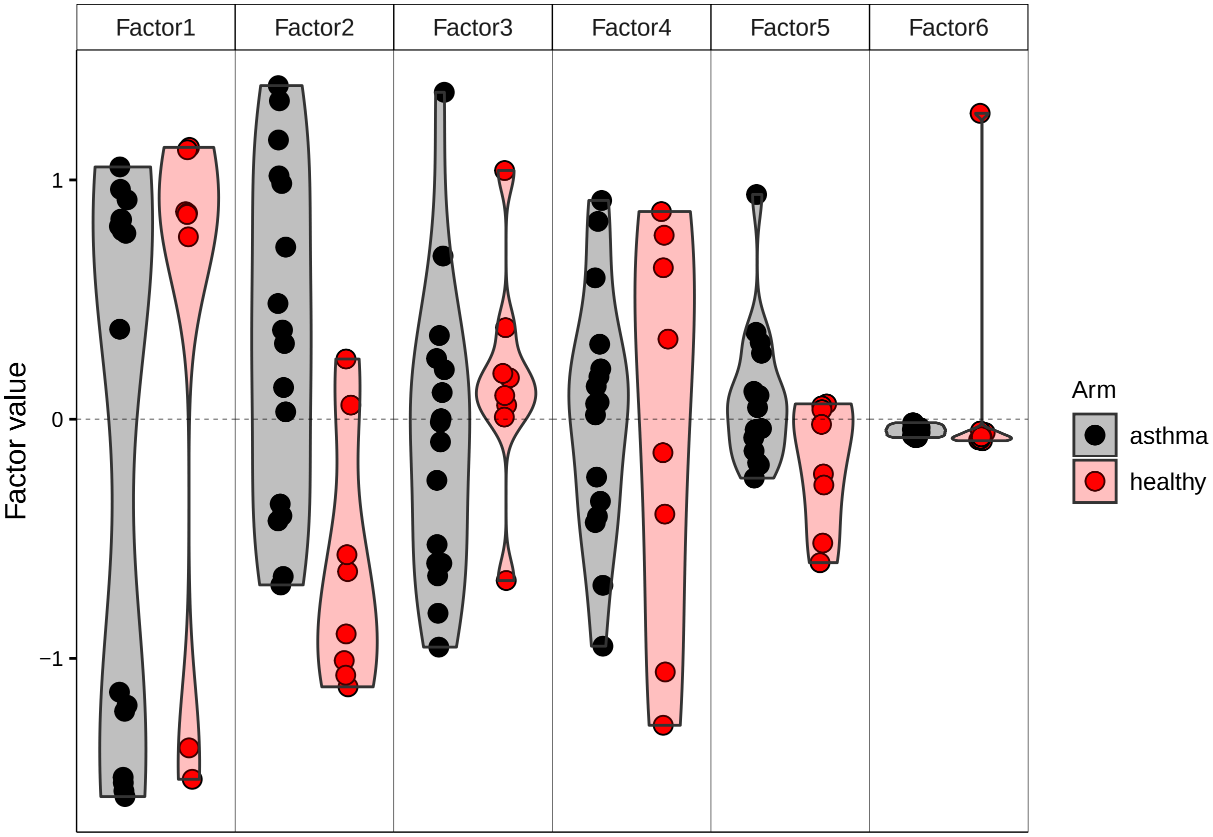

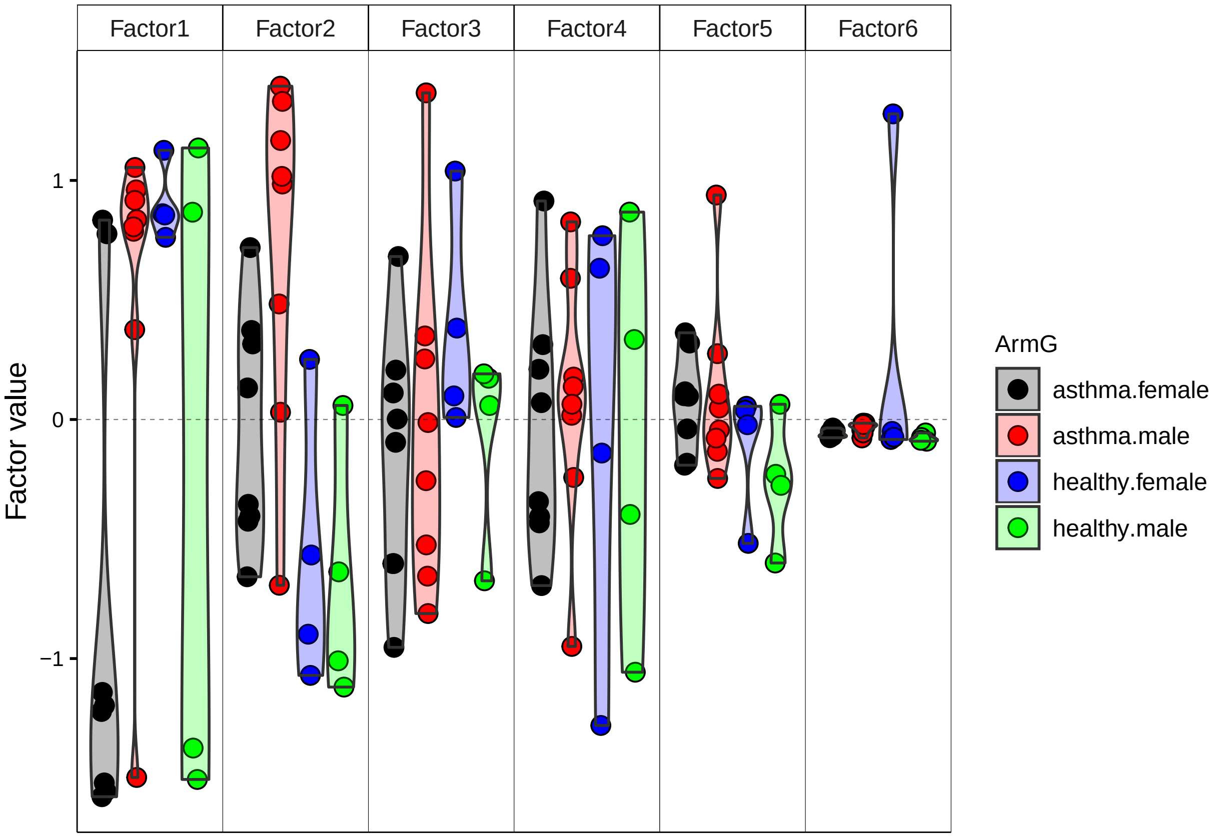
Factor1
Factor2
Factor3
Factor4
Factor5
Factor6
Factor value
1
0
−1
ArmG
asthma.female
asthma.male
healthy.female
healthy.male

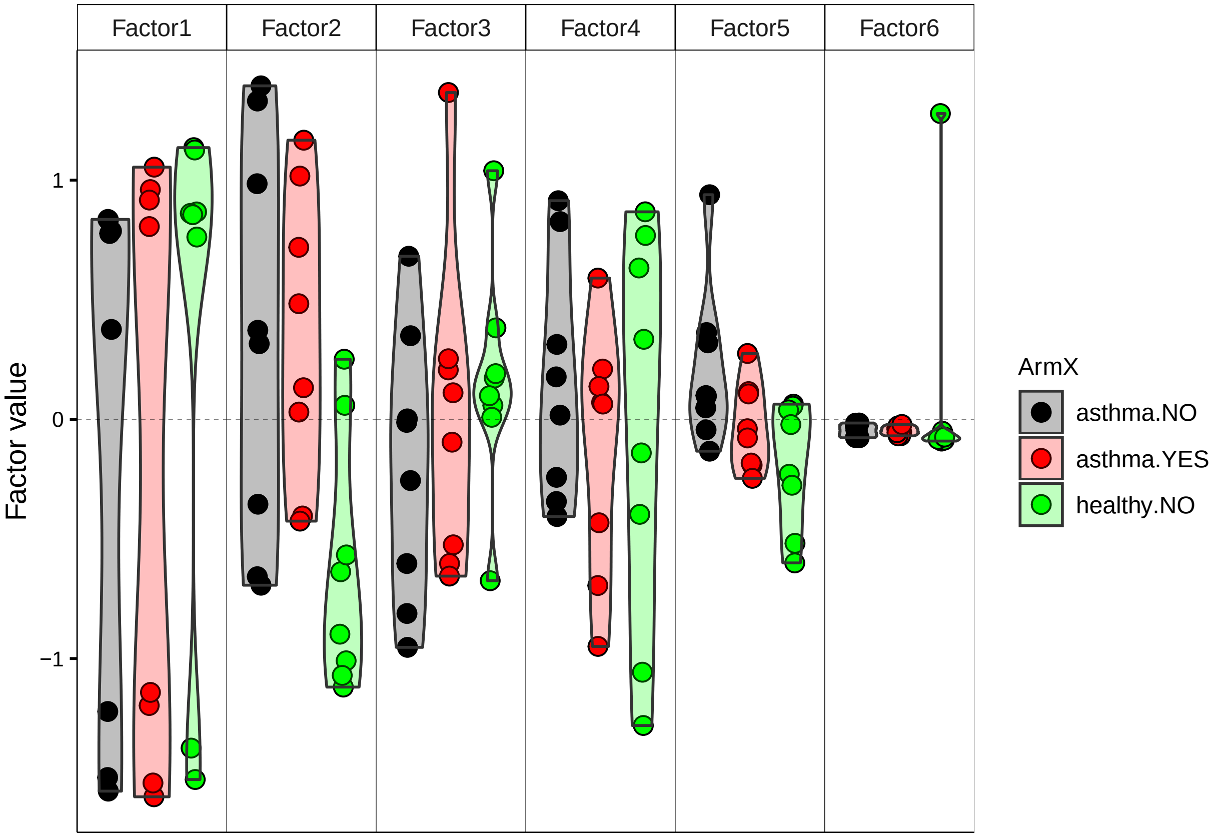
Factor1
Factor2
Factor3
Factor4
Factor5
Factor6
Factor value
1
0
−1
ArmX
asthma.NO
asthma.YES
healthy.NO

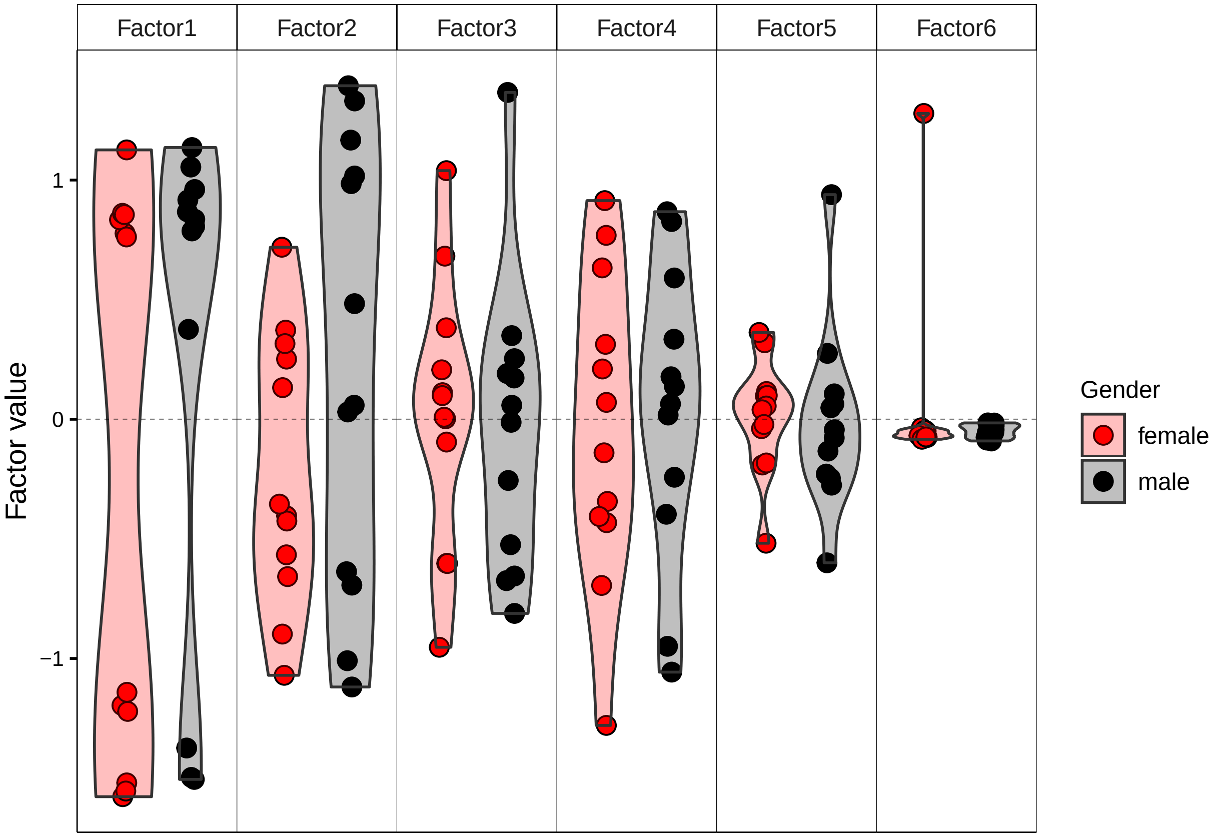
Factor1
Factor2
Factor3
Factor4
Factor5
Factor6
Factor value
1
0
−1
Gender
female
male

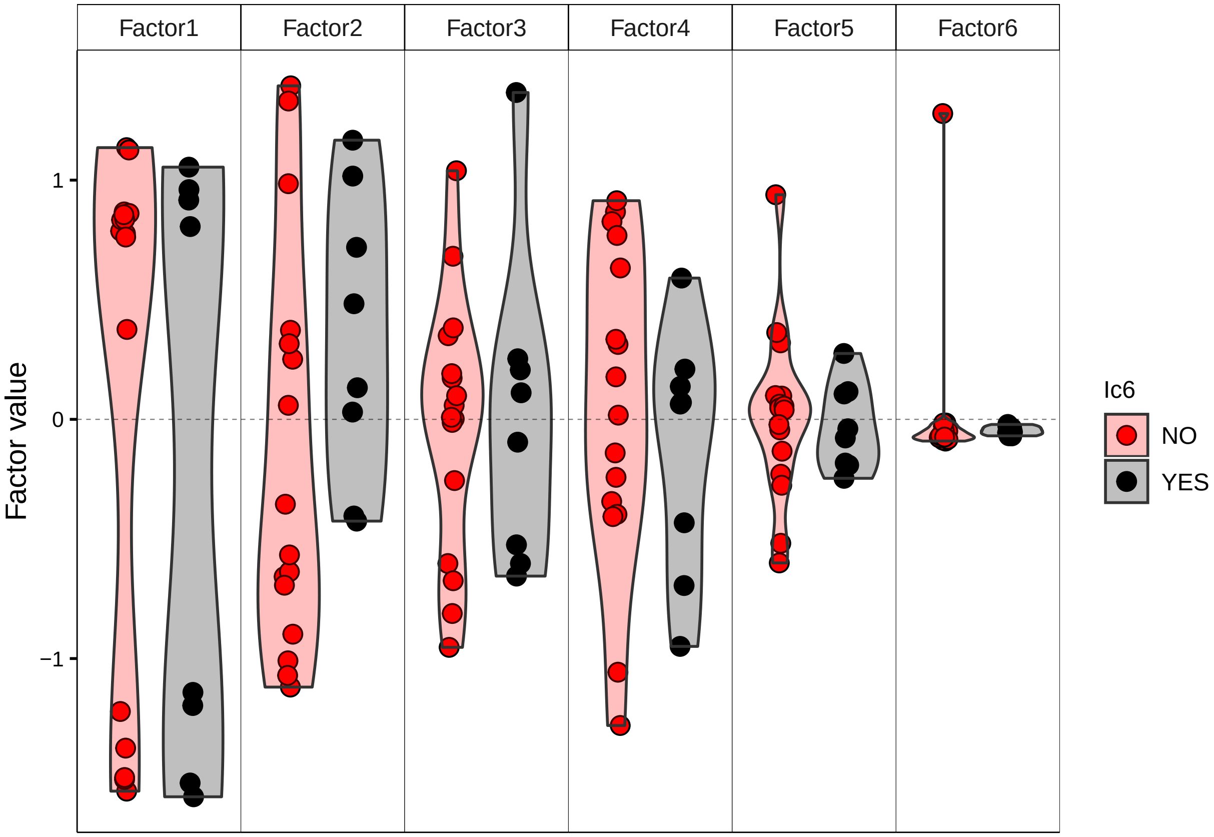
Factor1
Factor2
Factor3
Factor4
Factor5
Factor6
Factor value
1
0
−1
Ic6
NO
YES

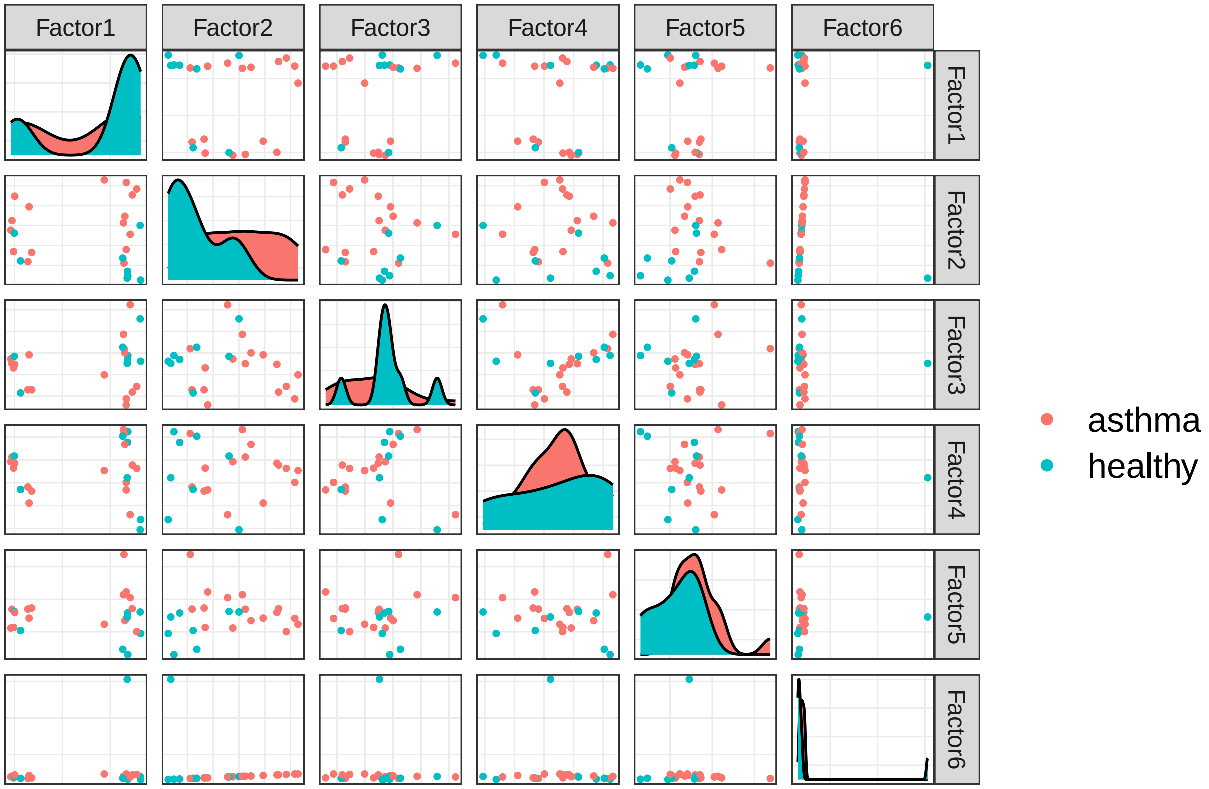

2.4 Factor matrix plot

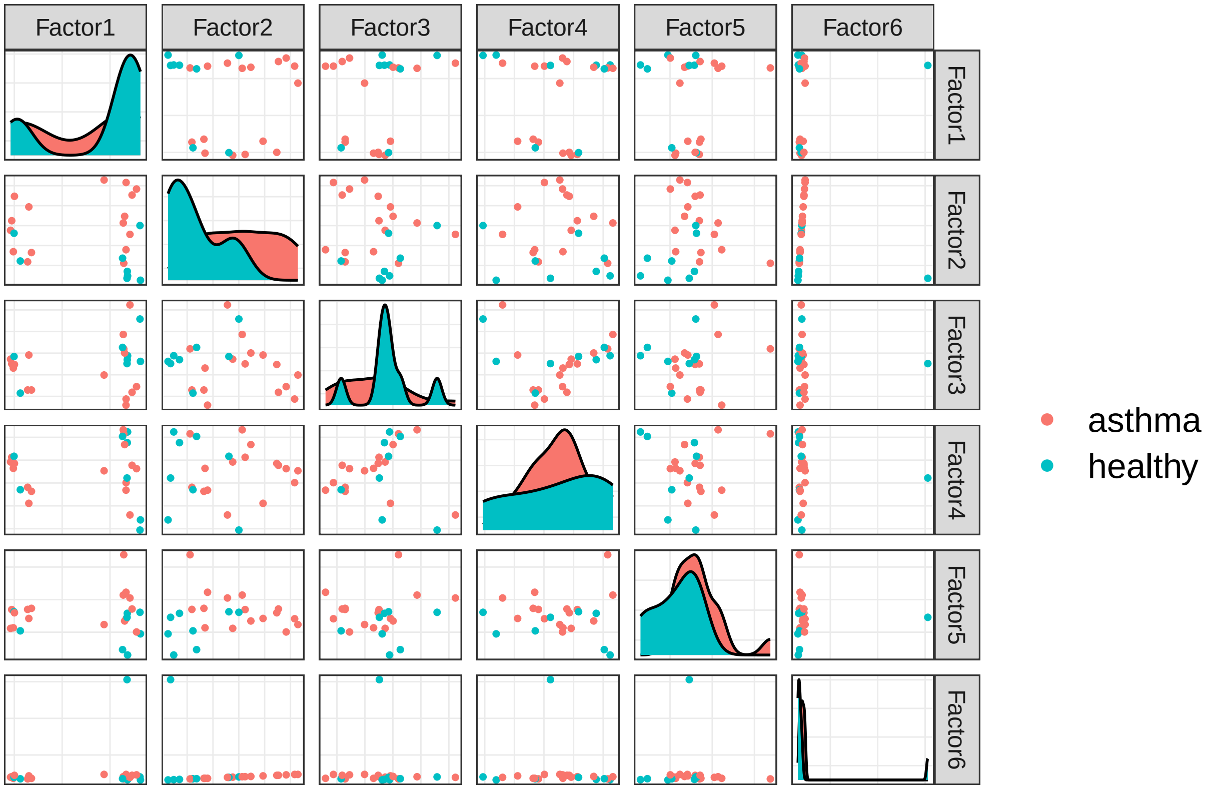

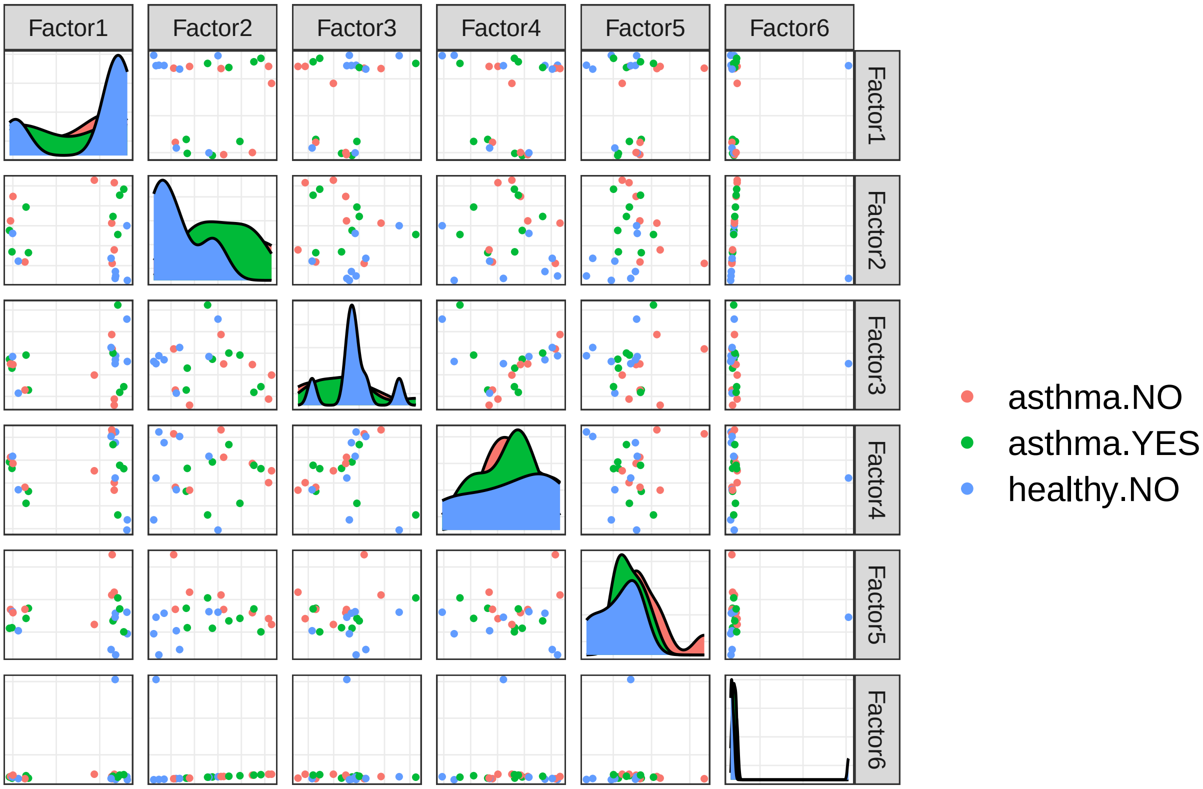
Factor1
Factor2
Factor3
Factor4
Factor5
Factor6
asthma.NO
asthma.YES
healthy.NO

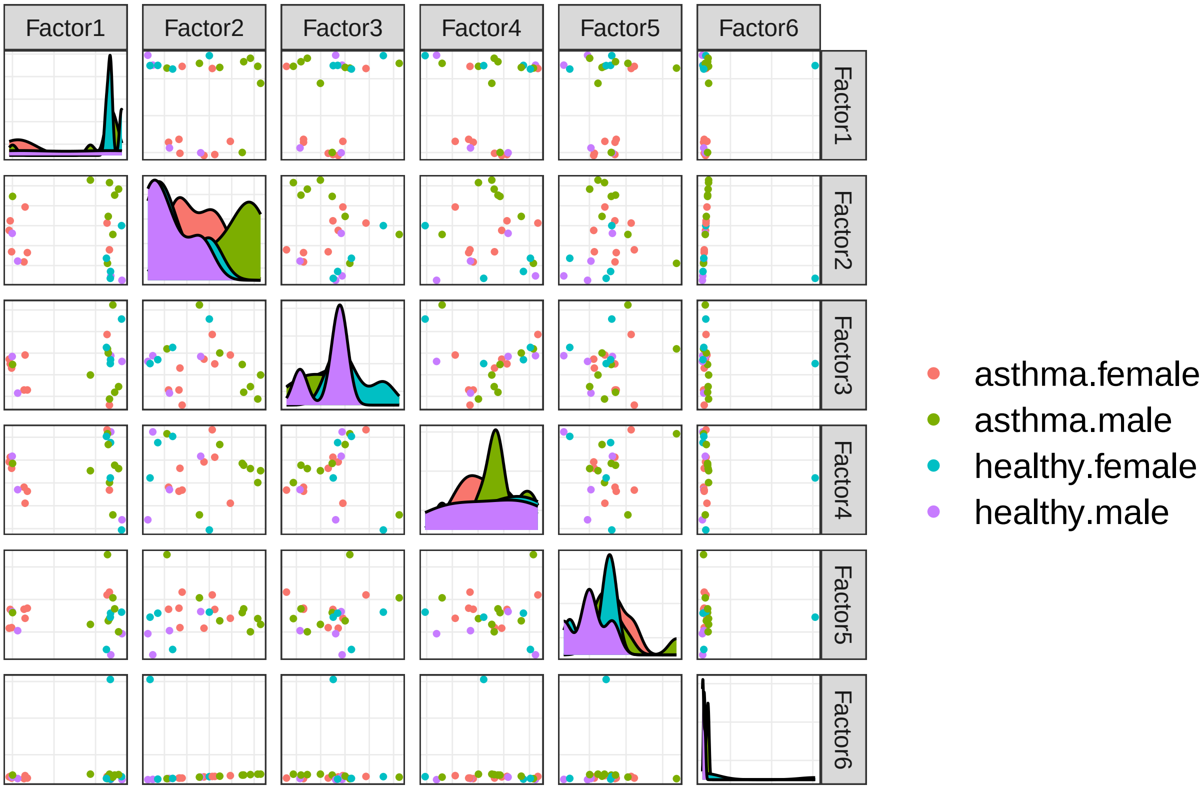
Factor1
Factor2
Factor3
Factor4
Factor5
Factor6
asthma.female
asthma.male
healthy.female
healthy.male

2.5 Plot weights

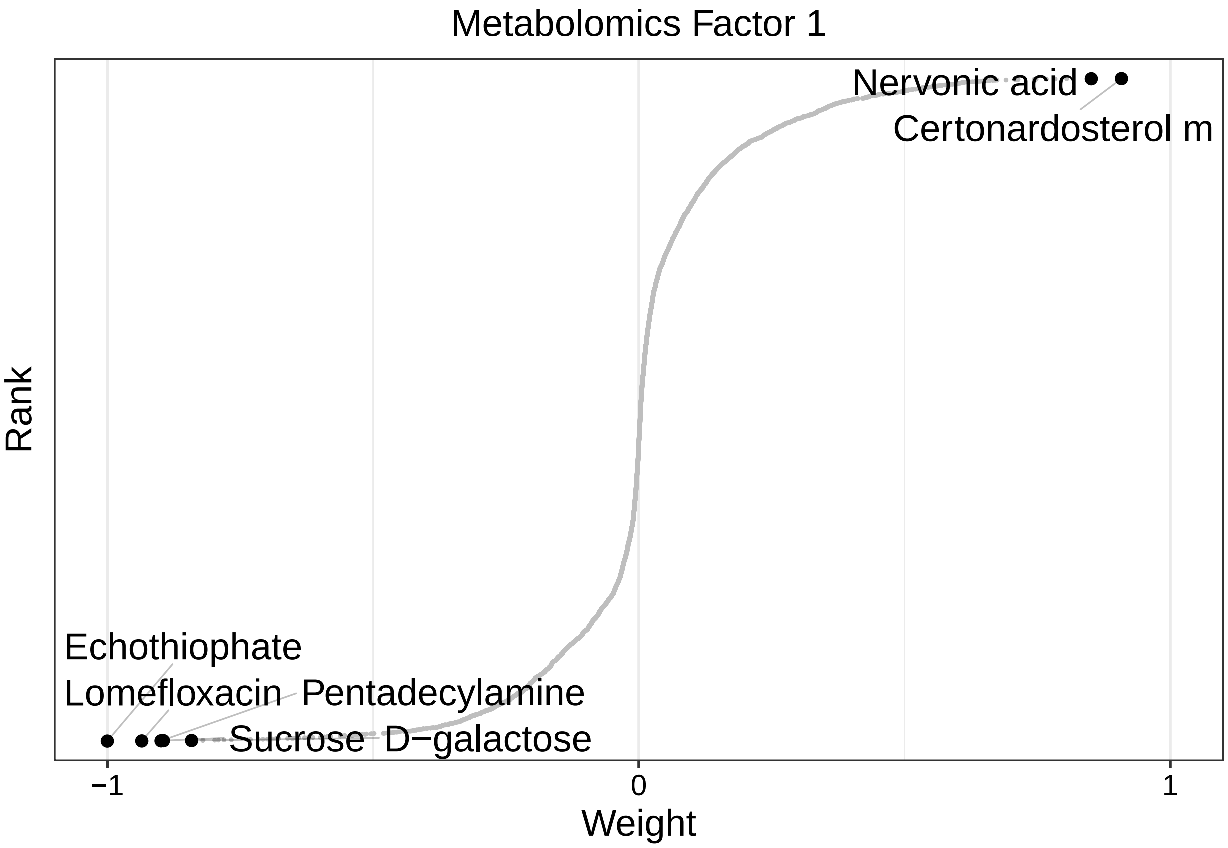

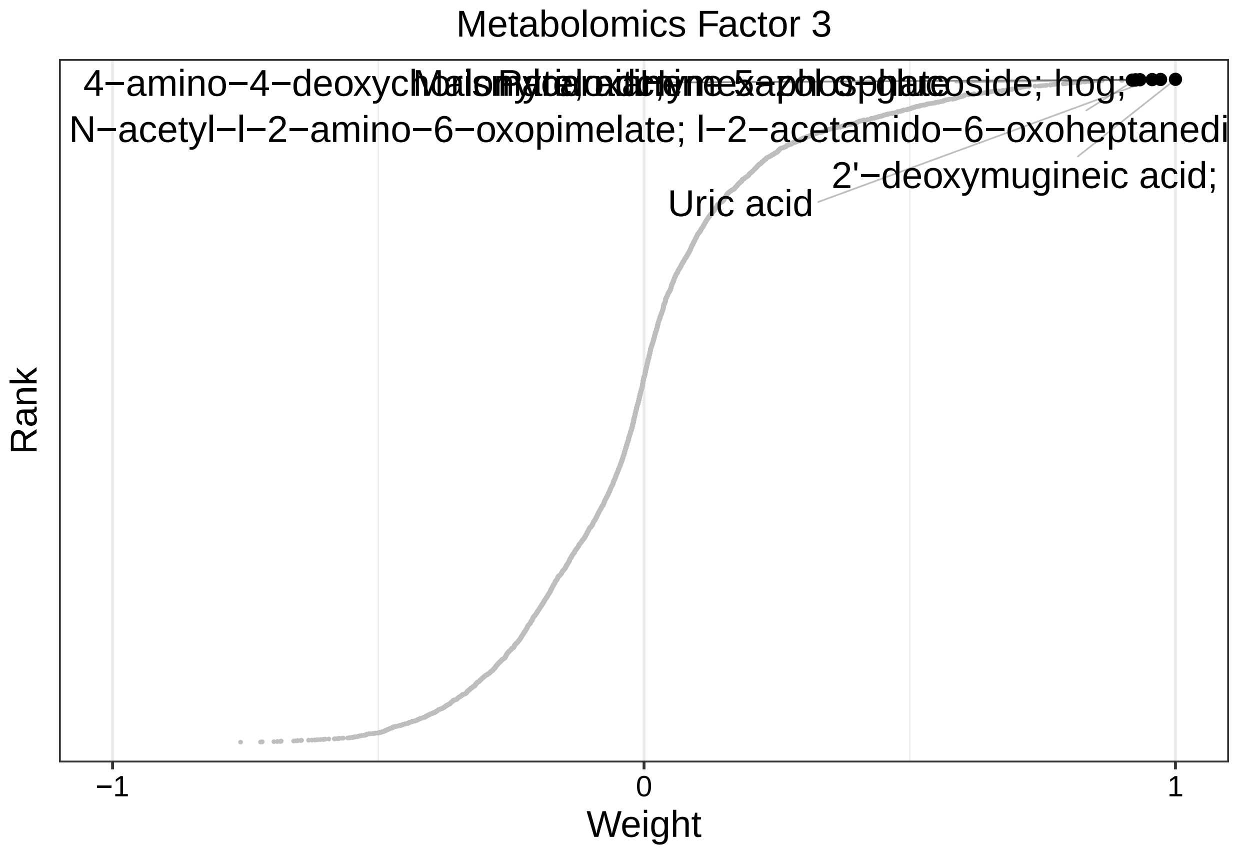
Metabolomics Factor 3
N−acetyl−l−2−amino−6−oxopimelate; l−2−acetamido−6−oxoheptanedi
2'−deoxymugineic acid;
Uric acid
Rank
−1
0
1
Weight

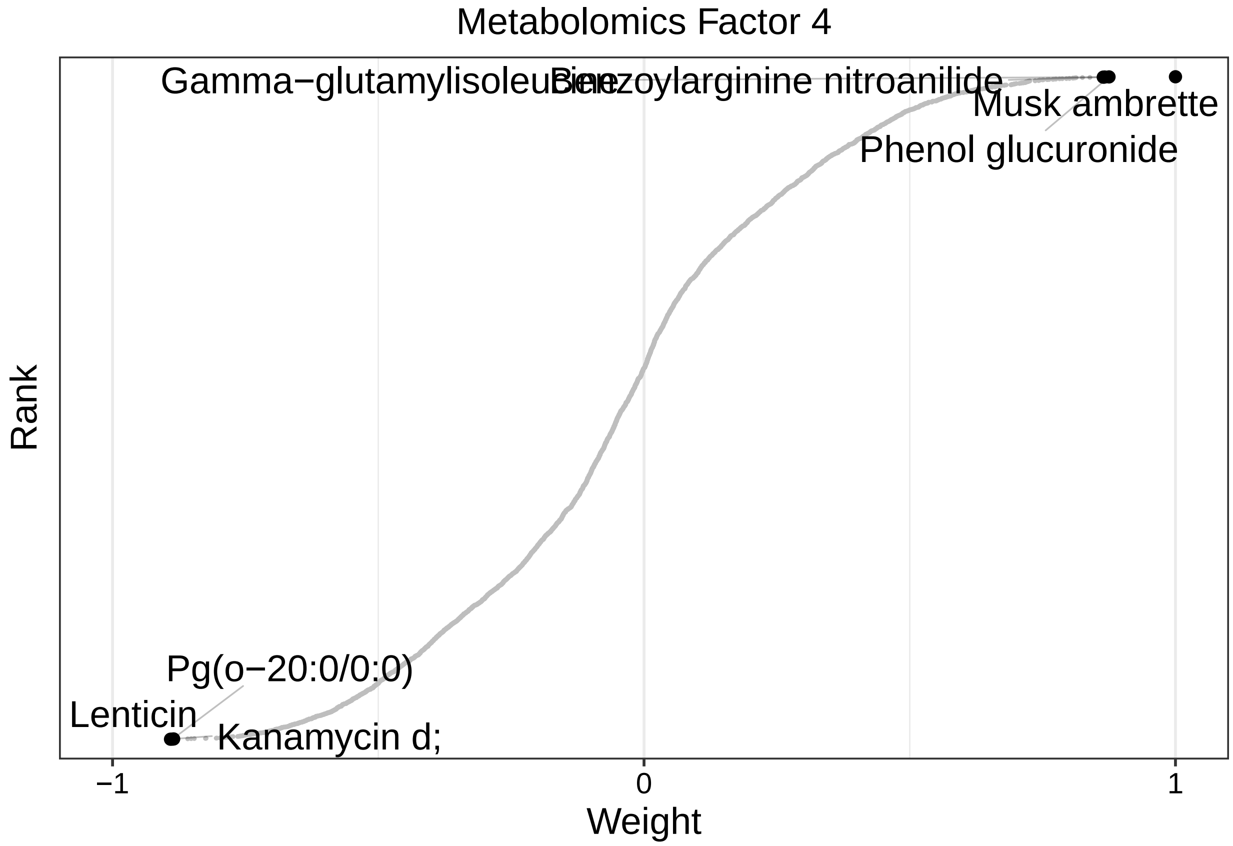
Metabolomics Factor 4
Benzoylarginine nitroanilide
Musk ambrette
Phenol glucuronide
Pg(o−20:0/0:0)
Lenticin
Kanamycin d;
Rank
−1
0
1
Weight

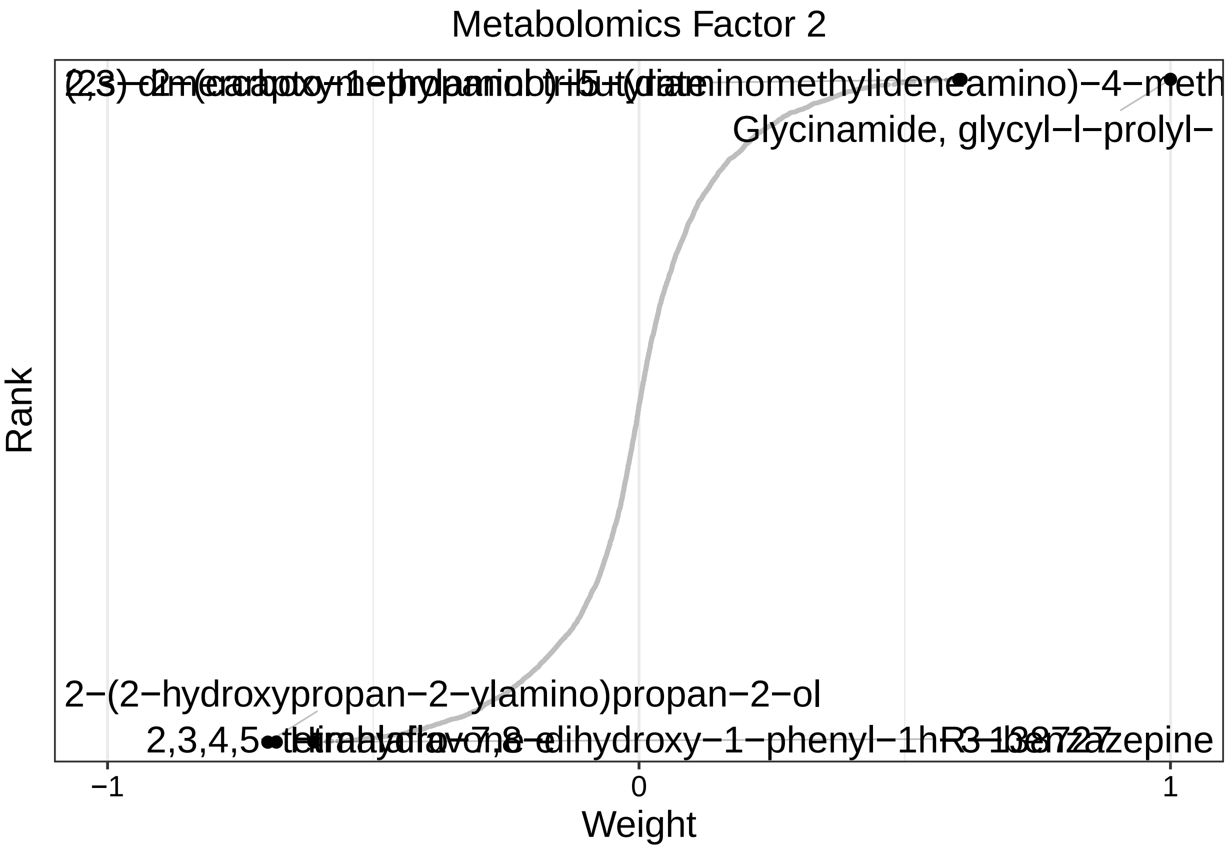

Metabolomics Factor 2
Glycinamide, glycyl−l−prolyl−
2−(2−hydroxypropan−2−ylamino)propan−2−ol
Rank
−1
0
1
Weight

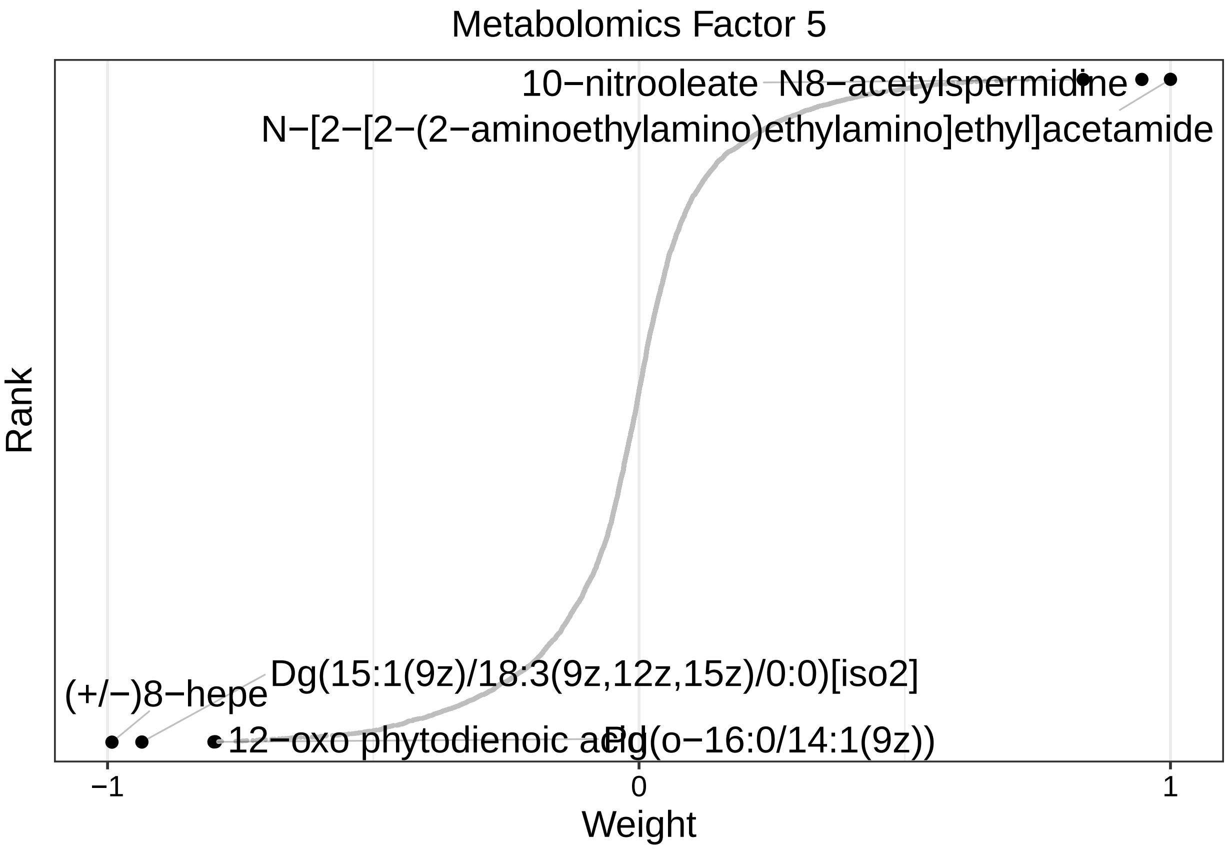

Metabolomics Factor 5
10−nitrooleate
N8−acetylspermidine
N−[2−[2−(2−aminoethylamino)ethylamino]ethyl]acetamide
Dg(15:1(9z)/18:3(9z,12z,15z)/0:0)[iso2]
(+/−)8−hepe
12−oxo phytodienoic acid
Pg(o−16:0/14:1(9z))
Rank
−1
0
1
Weight

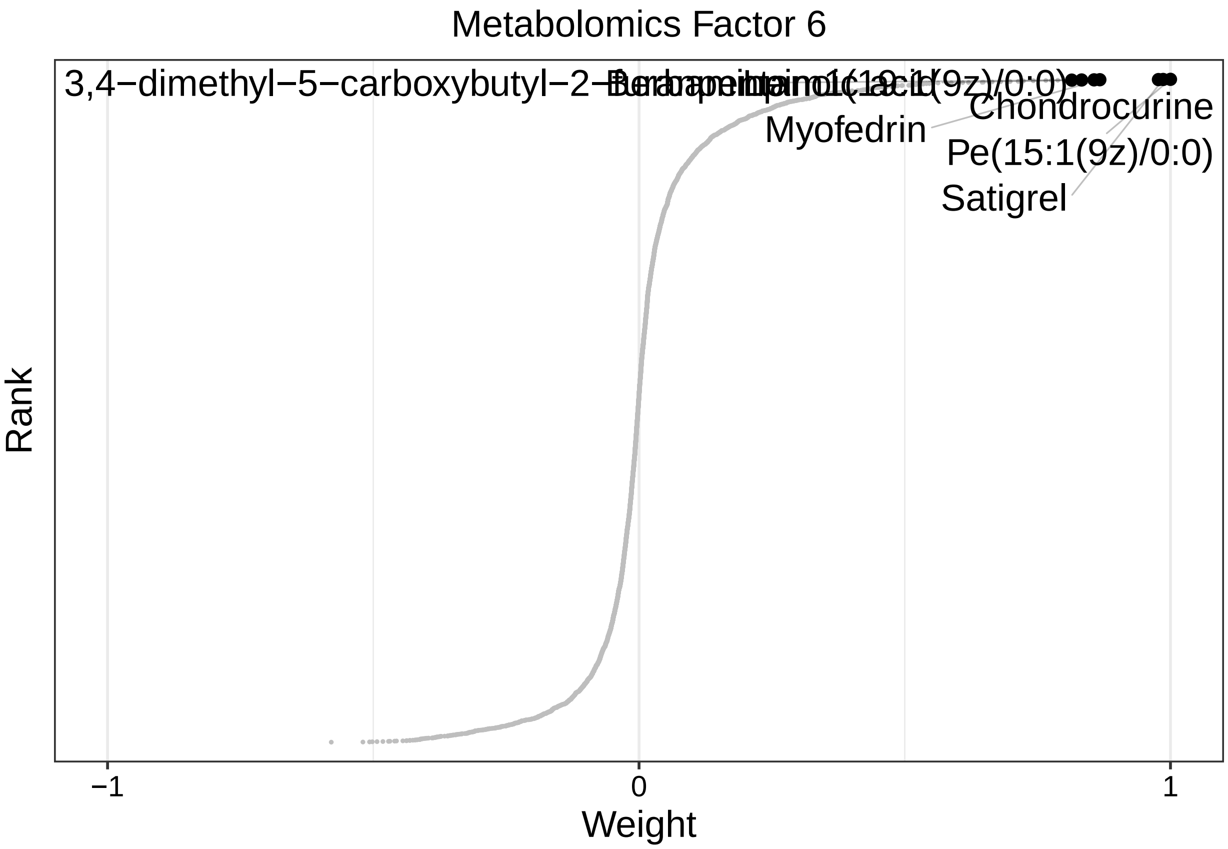
Metabolomics Factor 6
Myofedrin
Chondrocurine
Pe(15:1(9z)/0:0)
Satigrel
Rank
−1
0
1
Weight

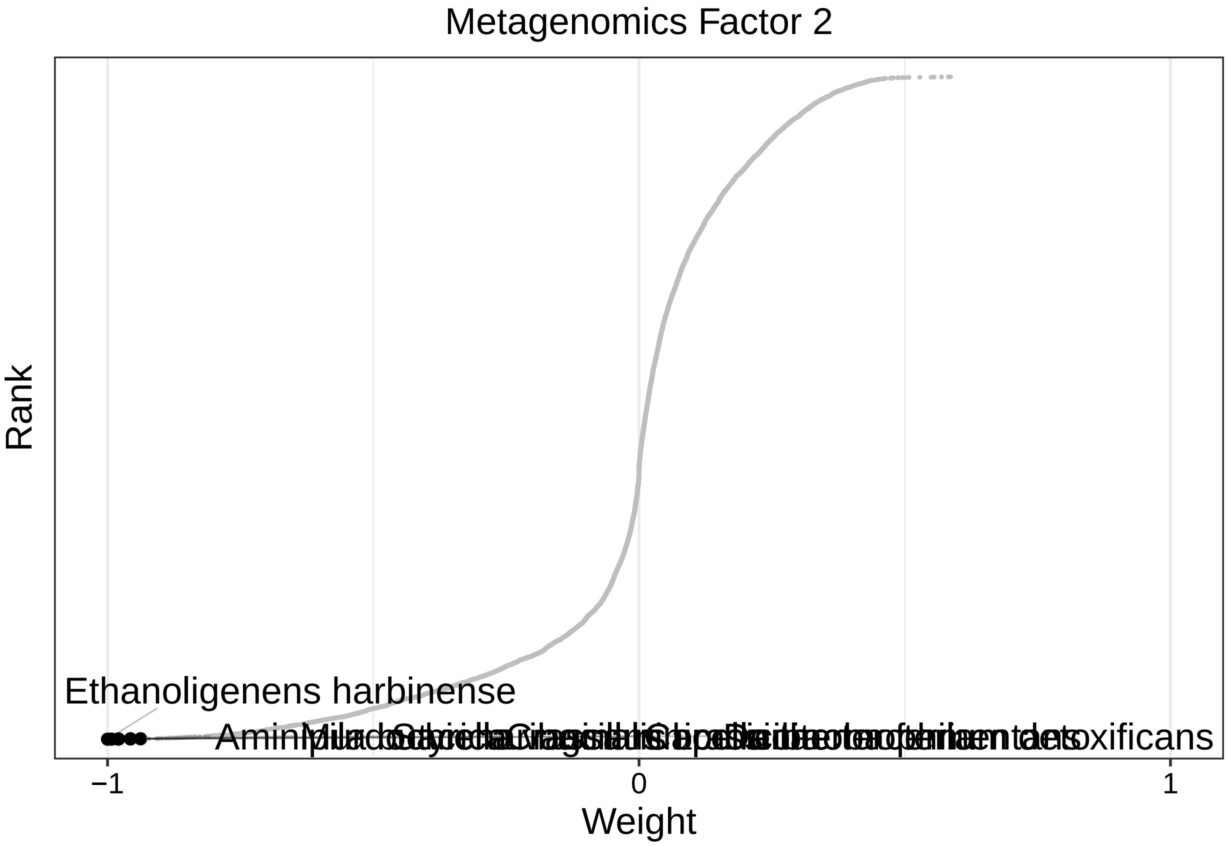
Metagenomics Factor 2
Ethanoligenens harbinense
Rank
−1
0
1
Weight

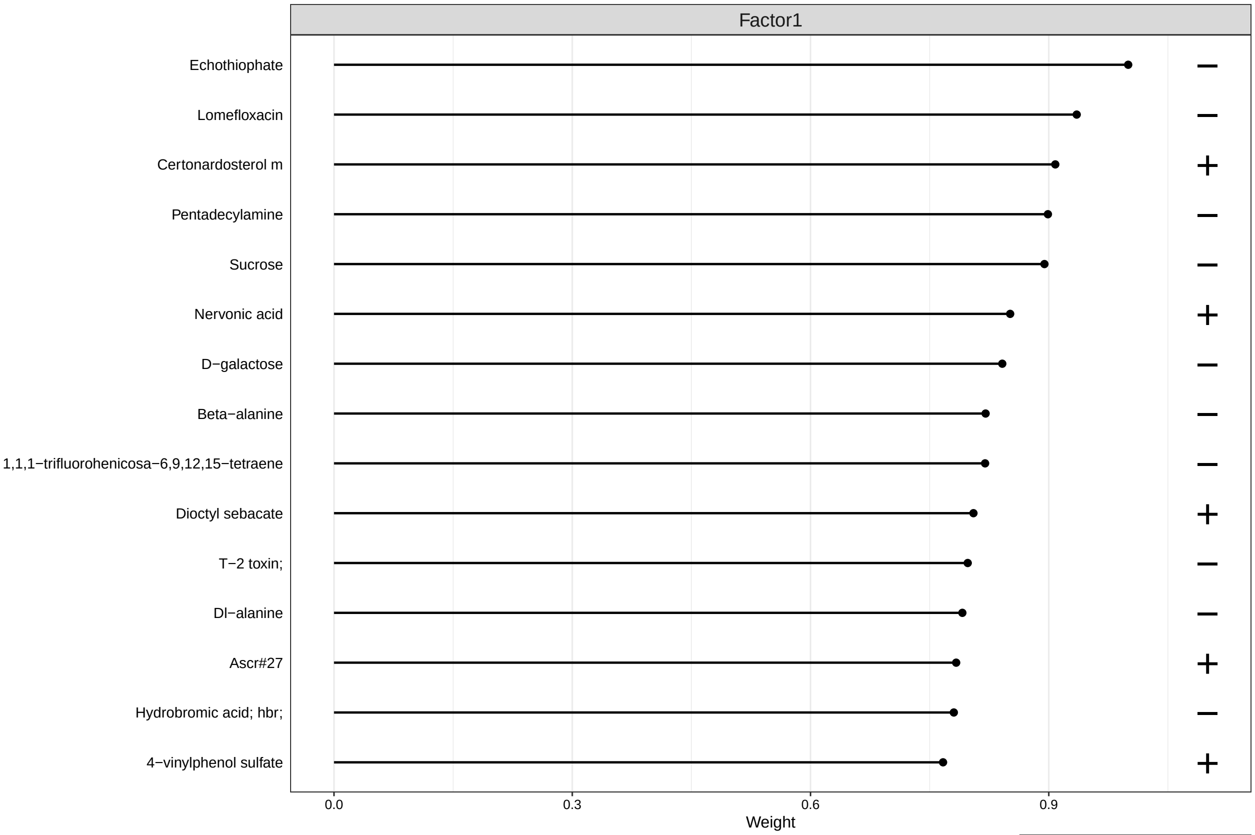

Factor1
Echothiophate
Lomefloxacin
Certonardosterol m
Pentadecylamine
Sucrose
Nervonic acid
D−galactose
Beta−alanine
1,1,1−trifluorohenicosa−6,9,12,15−tetraene
Dioctyl sebacate
T−2 toxin;
Dl−alanine
Ascr#27
Hydrobromic acid; hbr;
4−vinylphenol sulfate
0.0
0.3
0.6
0.9
Weight

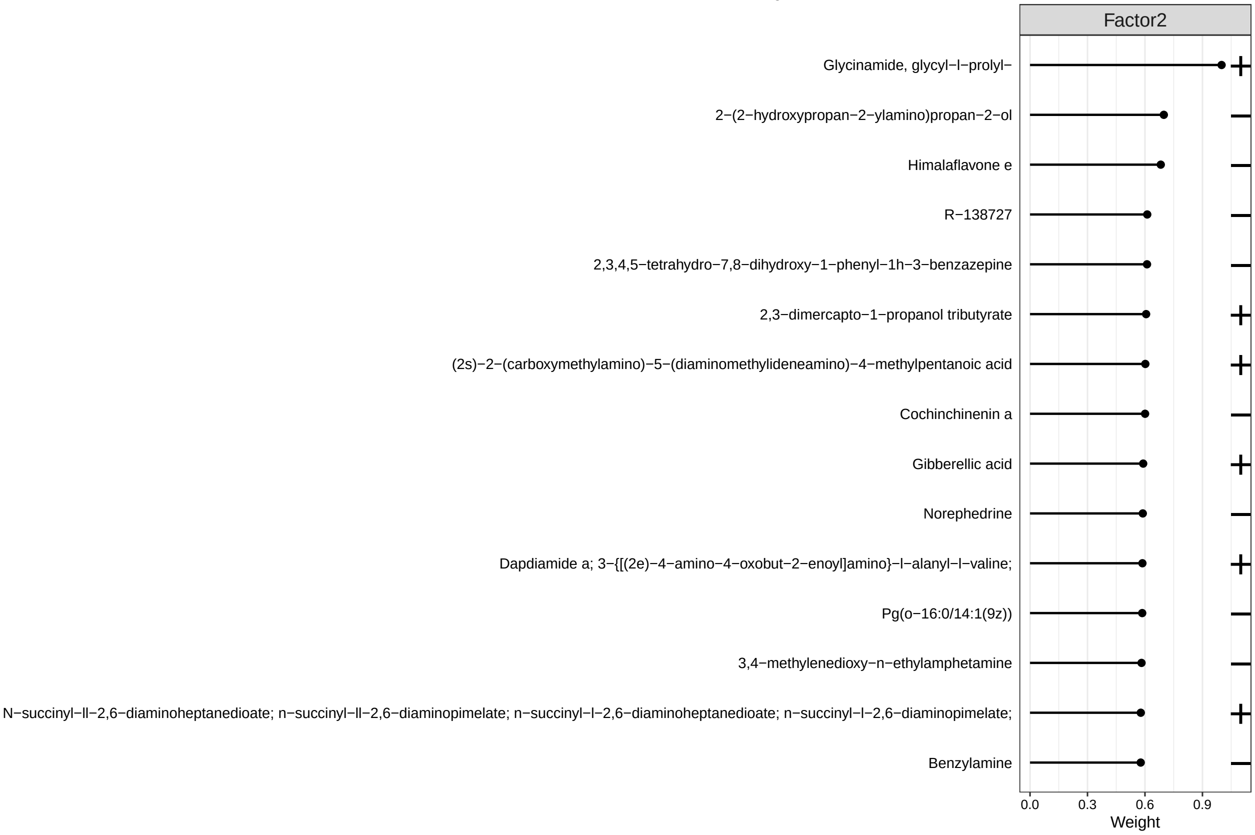

Factor2
Glycinamide, glycyl−l−prolyl−
2−(2−hydroxypropan−2−ylamino)propan−2−ol
Himalaflavone e
R−138727
2,3,4,5−tetrahydro−7,8−dihydroxy−1−phenyl−1h−3−benzazepine
2,3−dimercapto−1−propanol tributyrate
(2s)−2−(carboxymethylamino)−5−(diaminomethylideneamino)−4−methylpentanoic acid
Cochinchinenin a
Gibberellic acid
Norephedrine
Dapdiamide a; 3−{[(2e)−4−amino−4−oxobut−2−enoyl]amino}−l−alanyl−l−valine;
Pg(o−16:0/14:1(9z))
3,4−methylenedioxy−n−ethylamphetamine
N−succinyl−ll−2,6−diaminoheptanedioate; n−succinyl−ll−2,6−diaminopimelate; n−succinyl−l−2,6−diaminoheptanedioate; n−succinyl−l−2,6−diaminopimelate;
Benzylamine
0.0
0.3
0.6
0.9
Weight

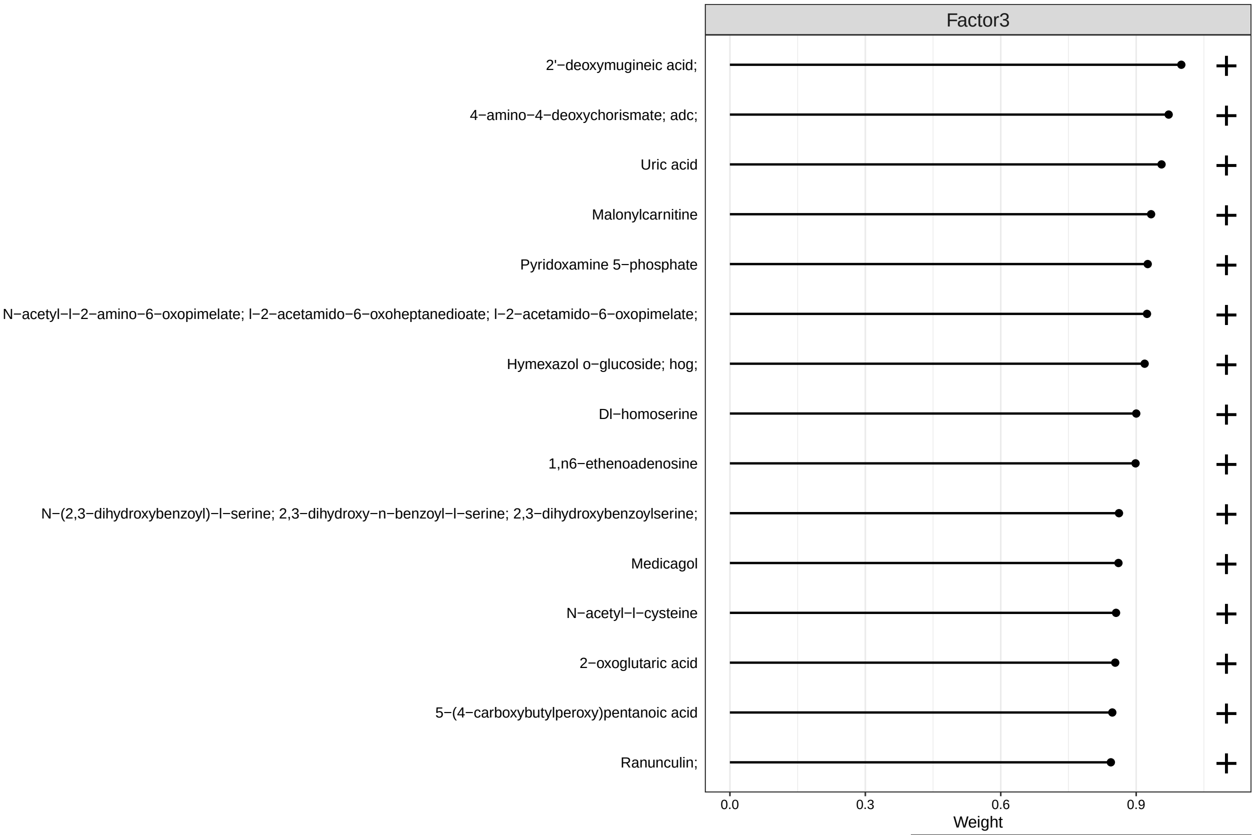
Factor3
2'−deoxymugineic acid;
4−amino−4−deoxychorismate; adc;
Uric acid
Malonylcarnitine
Pyridoxamine 5−phosphate
N−acetyl−l−2−amino−6−oxopimelate; l−2−acetamido−6−oxoheptanedioate; l−2−acetamido−6−oxopimelate;
Hymexazol o−glucoside; hog;
Dl−homoserine
1,n6−ethenoadenosine
N−(2,3−dihydroxybenzoyl)−l−serine; 2,3−dihydroxy−n−benzoyl−l−serine; 2,3−dihydroxybenzoylserine;
Medicagol
N−acetyl−l−cysteine
2−oxoglutaric acid
5−(4−carboxybutylperoxy)pentanoic acid
Ranunculin;
0.0
0.3
0.6
0.9
Weight

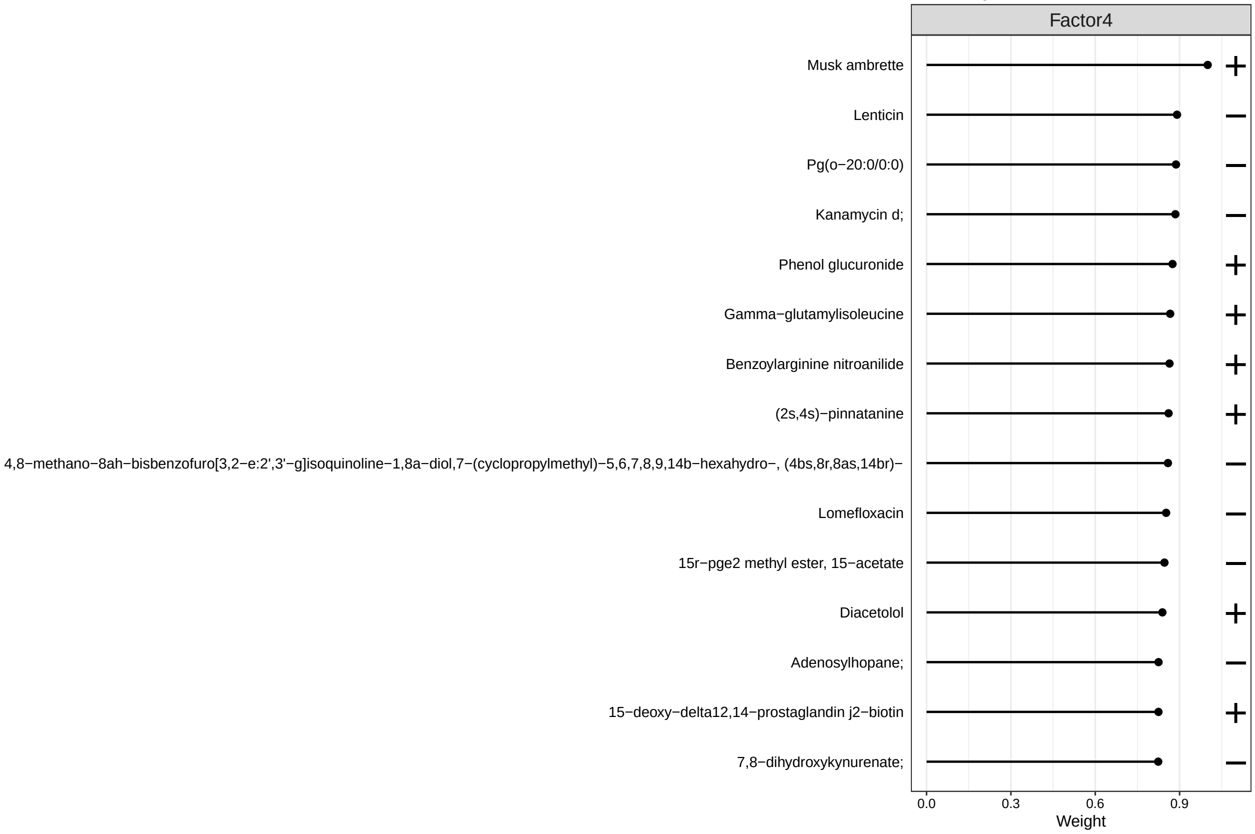
Factor4
Musk ambrette
Lenticin
Pg(o−20:0/0:0)
Kanamycin d;
Phenol glucuronide
Gamma−glutamylisoleucine
Benzoylarginine nitroanilide
(2s,4s)−pinnatanine
4,8−methano−8ah−bisbenzofuro[3,2−e:2',3'−g]isoquinoline−1,8a−diol,7−(cyclopropylmethyl)−5,6,7,8,9,14b−hexahydro−, (4bs,8r,8as,14br)−
Lomefloxacin
15r−pge2 methyl ester, 15−acetate
Diacetolol
Adenosylhopane;
15−deoxy−delta12,14−prostaglandin j2−biotin
7,8−dihydroxykynurenate;
0.0
0.3
0.6
0.9
Weight

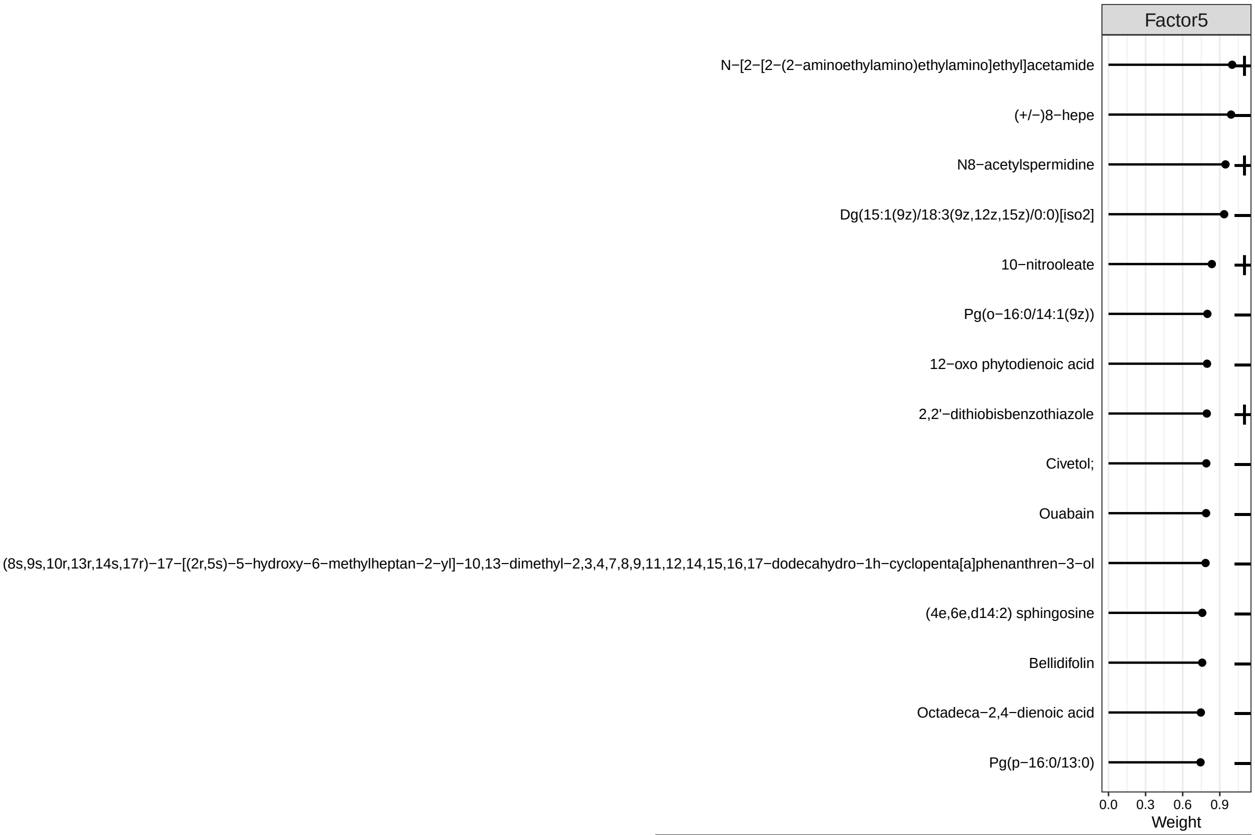
Factor5
N−[2−[2−(2−aminoethylamino)ethylamino]ethyl]acetamide
(+/−)8−hepe
N8−acetylspermidine
Dg(15:1(9z)/18:3(9z,12z,15z)/0:0)[iso2]
10−nitrooleate
Pg(o−16:0/14:1(9z))
12−oxo phytodienoic acid
2,2'−dithiobisbenzothiazole
Civetol;
Ouabain
(8s,9s,10r,13r,14s,17r)−17−[(2r,5s)−5−hydroxy−6−methylheptan−2−yl]−10,13−dimethyl−2,3,4,7,8,9,11,12,14,15,16,17−dodecahydro−1h−cyclopenta[a]phenanthren−3−ol
(4e,6e,d14:2) sphingosine
Bellidifolin
Octadeca−2,4−dienoic acid
Pg(p−16:0/13:0)
0.0
0.3
0.6
0.9
Weight

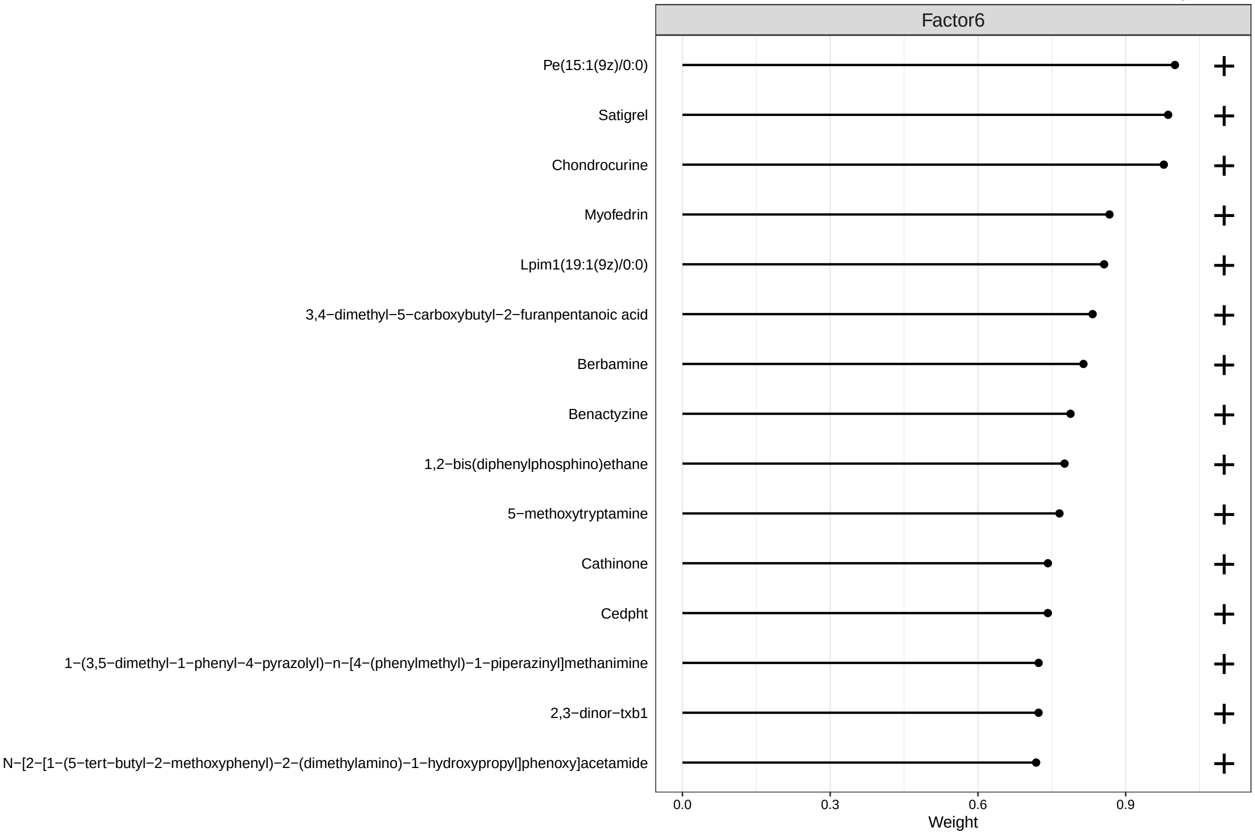
Factor6
Pe(15:1(9z)/0:0)
Satigrel
Chondrocurine
Myofedrin
Lpim1(19:1(9z)/0:0)
3,4−dimethyl−5−carboxybutyl−2−furanpentanoic acid
Berbamine
Benactyzine
1,2−bis(diphenylphosphino)ethane
5−methoxytryptamine
Cathinone
Cedpht
1−(3,5−dimethyl−1−phenyl−4−pyrazolyl)−n−[4−(phenylmethyl)−1−piperazinyl]methanimine
2,3−dinor−txb1
N−[2−[1−(5−tert−butyl−2−methoxyphenyl)−2−(dimethylamino)−1−hydroxypropyl]phenoxy]acetamide
0.0
0.3
0.6
0.9
Weight

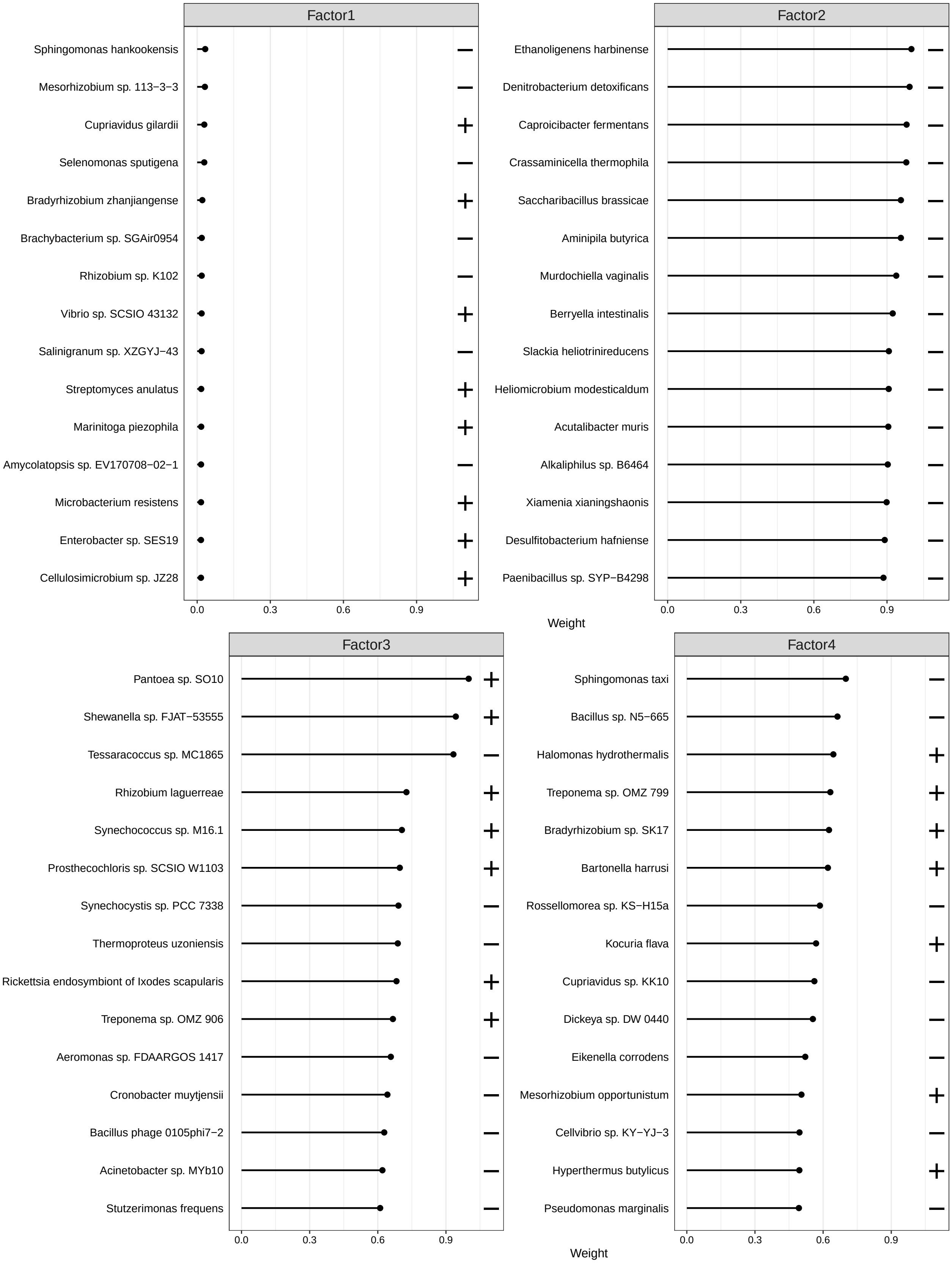
Factor1
Sphingomonas hankookensis
Mesorhizobium sp. 113−3−3
Cupriavidus gilardii
Selenomonas sputigena
Bradyrhizobium zhanjiangense
Brachybacterium sp. SGAir0954
Rhizobium sp. K102
Vibrio sp. SCSIO 43132
Salinigranum sp. XZGYJ−43
Streptomyces anulatus
Marinitoga piezophila
Amycolatopsis sp. EV170708−02−1
Microbacterium resistens
Enterobacter sp. SES19
Cellulosimicrobium sp. JZ28
Factor2
Ethanoligenens harbinense
Denitrobacterium detoxificans
Caproicibacter fermentans
Crassaminicella thermophila
Saccharibacillus brassicae
Aminipila butyrica
Murdochiella vaginalis
Berryella intestinalis
Slackia heliotrinireducens
Heliomicrobium modesticaldum
Acutalibacter muris
Alkaliphilus sp. B6464
Xiamenia xianingshaonis
Desulfitobacterium hafniense
Paenibacillus sp. SYP−B4298
0.0
0.3
0.6
0.9
Weight
Factor3
Pantoea sp. SO10
Shewanella sp. FJAT−53555
Tessaracoccus sp. MC1865
Rhizobium laguerreae
Synechococcus sp. M16.1
Prosthecochloris sp. SCSIO W1103
Synechocystis sp. PCC 7338
Thermoproteus uzoniensis
Rickettsia endosymbiont of Ixodes scapularis
Treponema sp. OMZ 906
Aeromonas sp. FDAARGOS 1417
Cronobacter muytjensii
Bacillus phage 0105phi7−2
Acinetobacter sp. MYb10
Stutzerimonas frequens
Factor4
Sphingomonas taxi
Bacillus sp. N5−665
Halomonas hydrothermalis
Treponema sp. OMZ 799
Bradyrhizobium sp. SK17
Bartonella harrusi
Rossellomorea sp. KS−H15a
Kocuria flava
Cupriavidus sp. KK10
Dickeya sp. DW 0440
Eikenella corrodens
Mesorhizobium opportunistum
Cellvibrio sp. KY−YJ−3
Hyperthermus butylicus
Pseudomonas marginalis
0.0
0.3
0.6
0.9
Weight

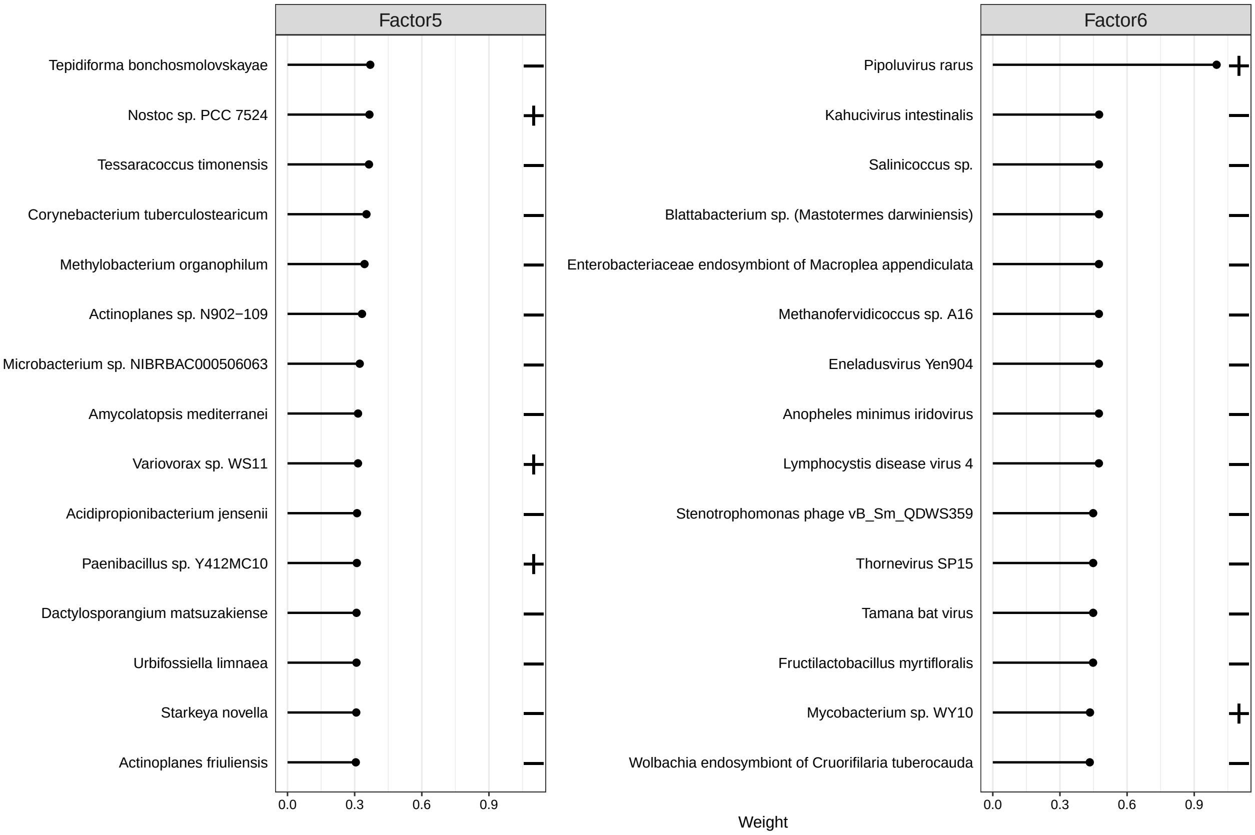
Factor5
Tepidiforma bonchosmolovskayae
Nostoc sp. PCC 7524
Tessaracoccus timonensis
Corynebacterium tuberculostearicum
Methylobacterium organophilum
Actinoplanes sp. N902−109
Microbacterium sp. NIBRBAC000506063
Amycolatopsis mediterranei
Variovorax sp. WS11
Acidipropionibacterium jensenii
Paenibacillus sp. Y412MC10
Dactylosporangium matsuzakiense
Urbifossiella limnaea
Starkeya novella
Actinoplanes friuliensis
Factor6
Pipoluvirus rarus
Kahucivirus intestinalis
Salinicoccus sp.
Blattabacterium sp. (Mastotermes darwiniensis)
Enterobacteriaceae endosymbiont of Macroplea appendiculata
Methanofervidicoccus sp. A16
Eneladusvirus Yen904
Anopheles minimus iridovirus
Lymphocystis disease virus 4
Stenotrophomonas phage vB_Sm_QDWS359
Thornevirus SP15
Tamana bat virus
Fructilactobacillus myrtifloralis
Mycobacterium sp. WY10
Wolbachia endosymbiont of Cruorifilaria tuberocauda
0.0
0.3
0.6
0.9
Weight

2.6 UMAP projection

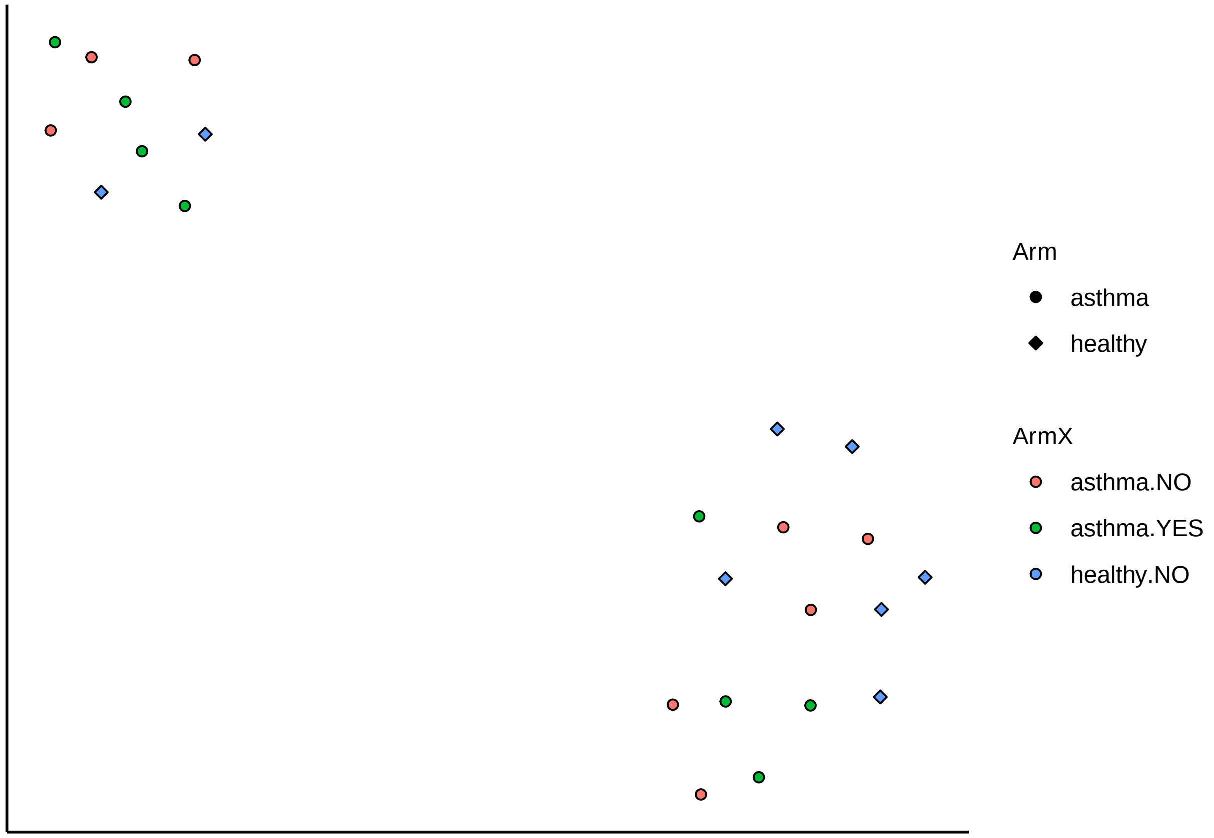

3 KEGG metabolites only model

3.1 Read model

3.2 Variance explained

metabolomics	metagenomics
56.9	17.6

	metabolomics	metagenomics
Factor1	21.4	0.0875
Factor2	4.79	13
Factor3	17.4	0.181
Factor4	11.8	0.252
Factor5	1.56	2.69
Factor6	0.21	1.5

3.3 Factor Plots

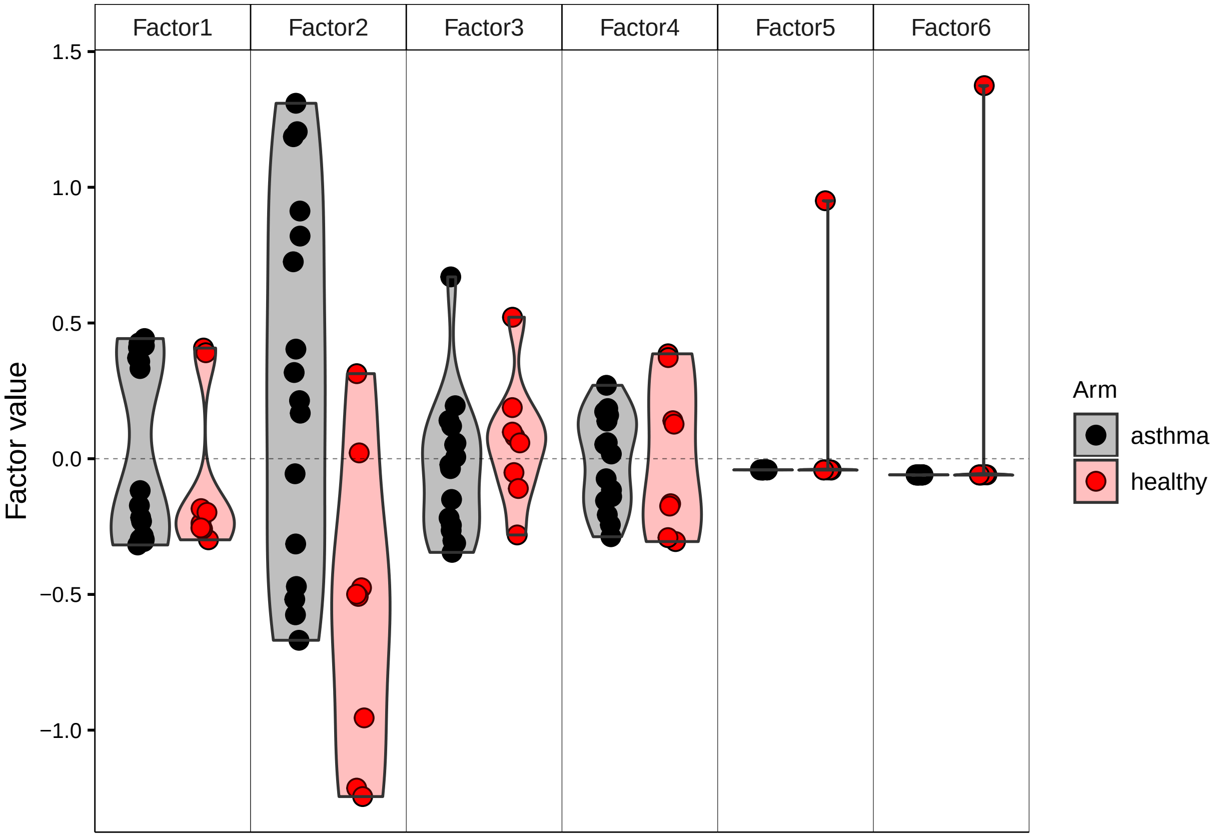

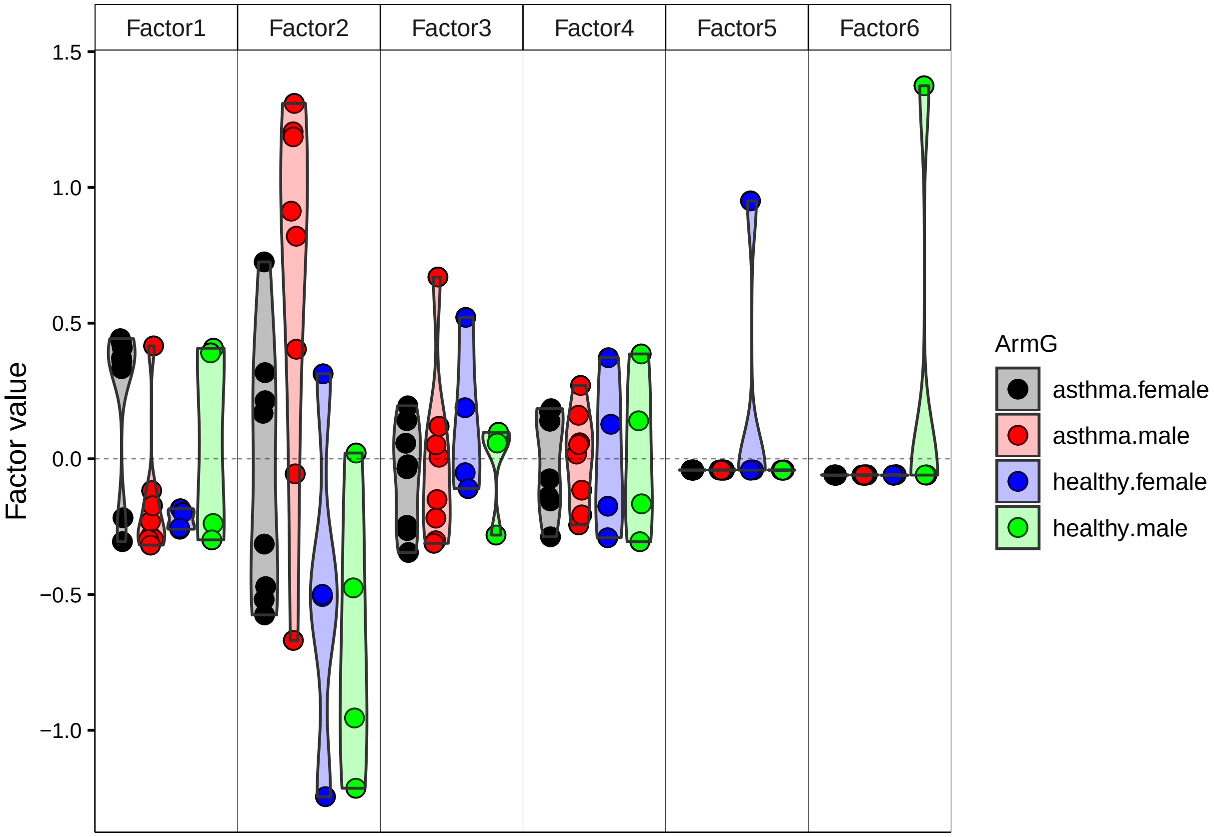
Factor1
Factor2
Factor3
Factor4
Factor5
Factor6
1.5
1.0
0.5
0.0
−0.5
−1.0
Factor value
ArmG
asthma.female
asthma.male
healthy.female
healthy.male

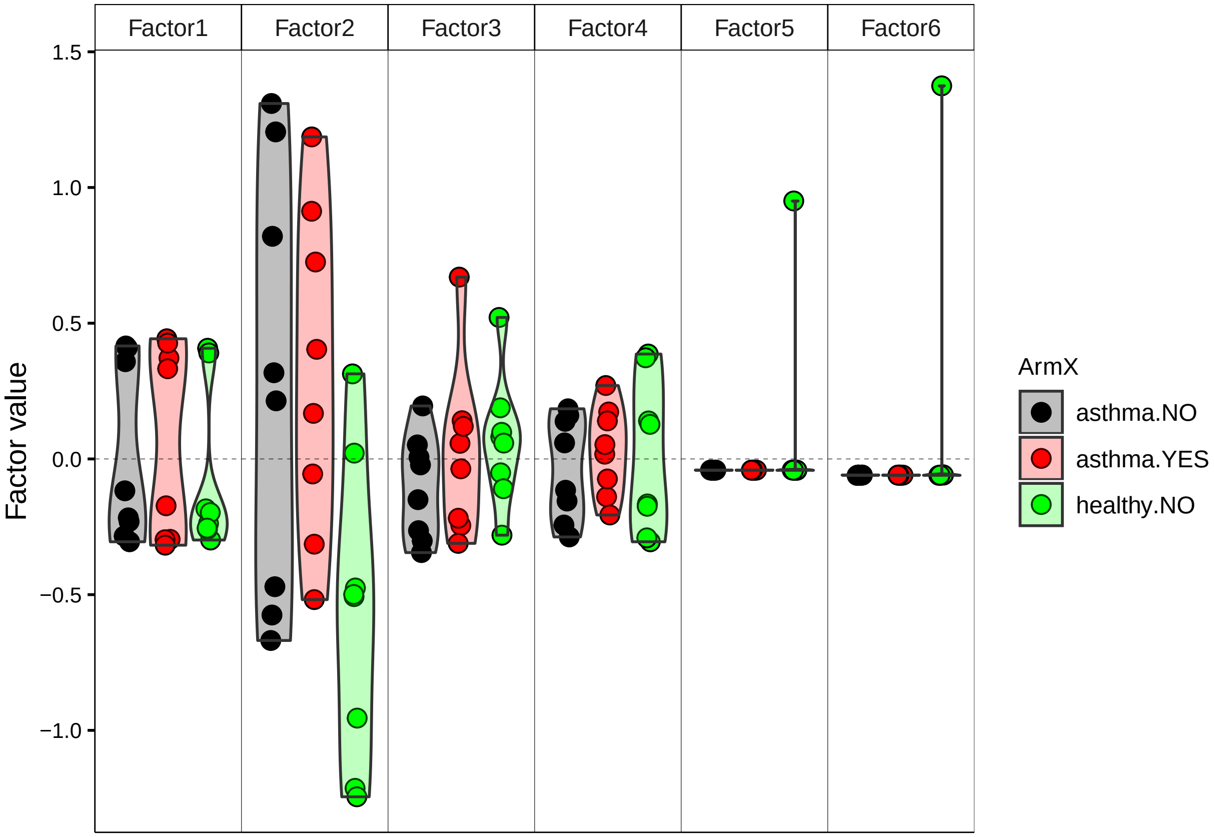
Factor1
Factor2
Factor3
Factor4
Factor5
Factor6
1.5
1.0
0.5
0.0
−0.5
−1.0
Factor value
ArmX
asthma.NO
asthma.YES
healthy.NO

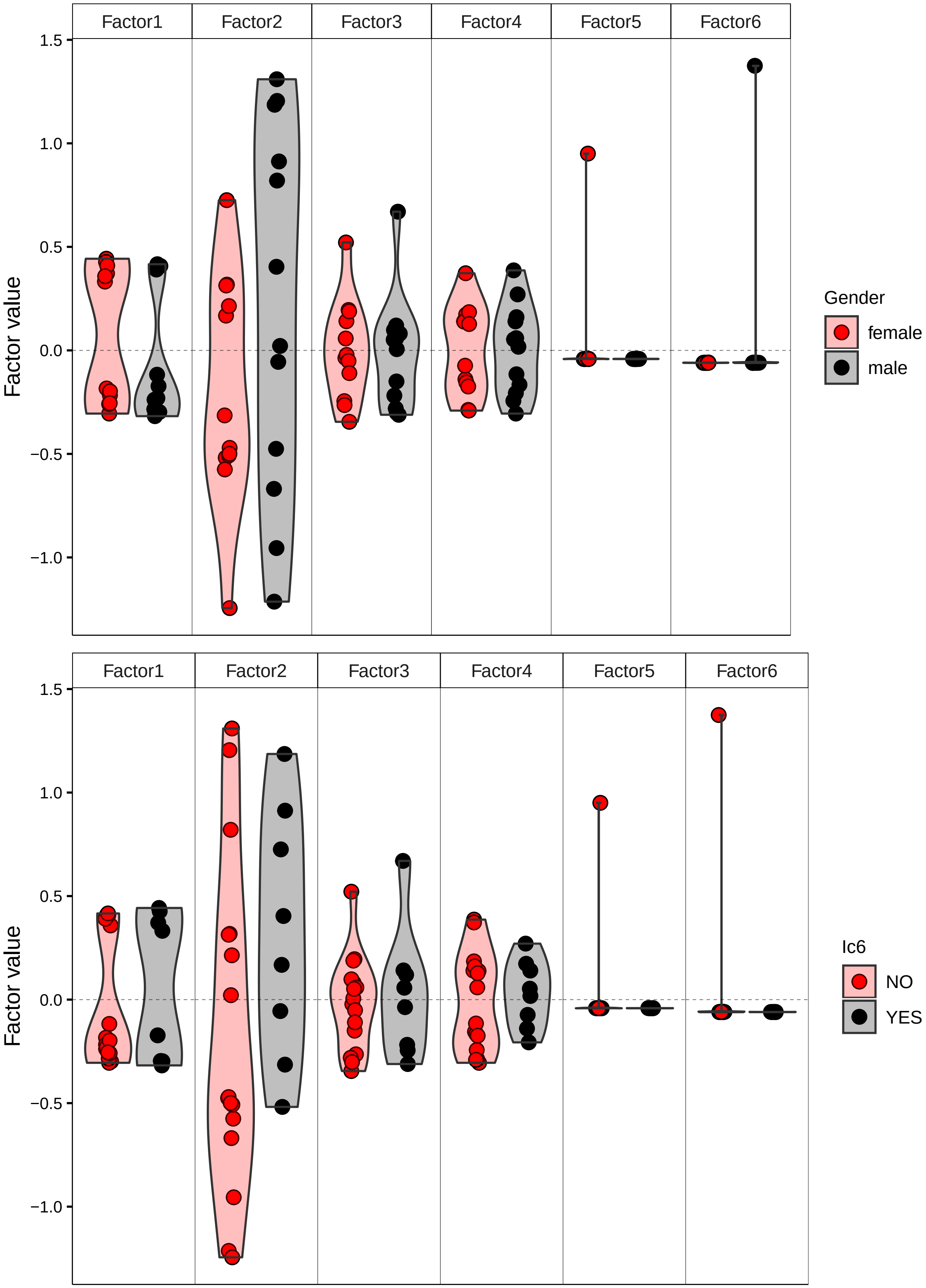
Factor1
Factor2
Factor3
Factor4
Factor5
Factor6
Factor value
1.5
1.0
0.5
0.0
−0.5
−1.0
Gender
female
male
Factor1
Factor2
Factor3
Factor4
Factor5
Factor6
Factor value
1.5
1.0
0.5
0.0
−0.5
−1.0
Ic6
NO
YES

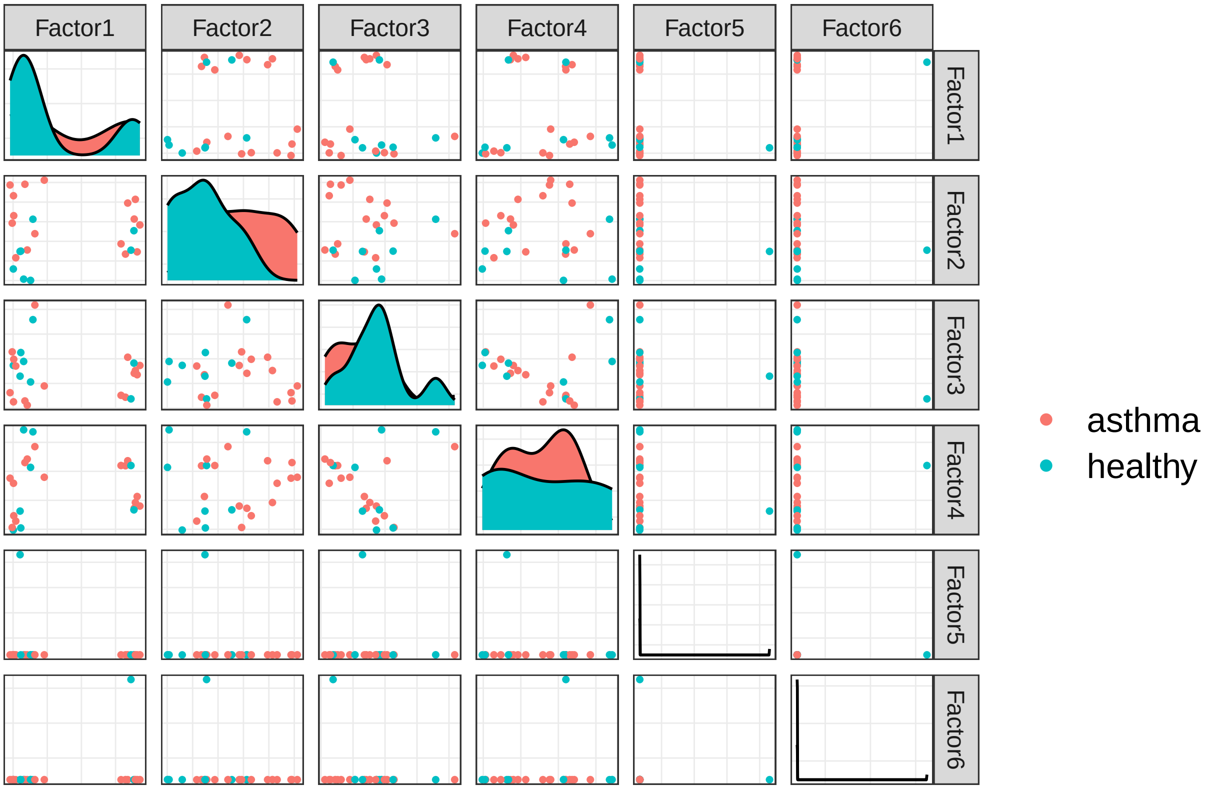

3.4 Factor matrix plot

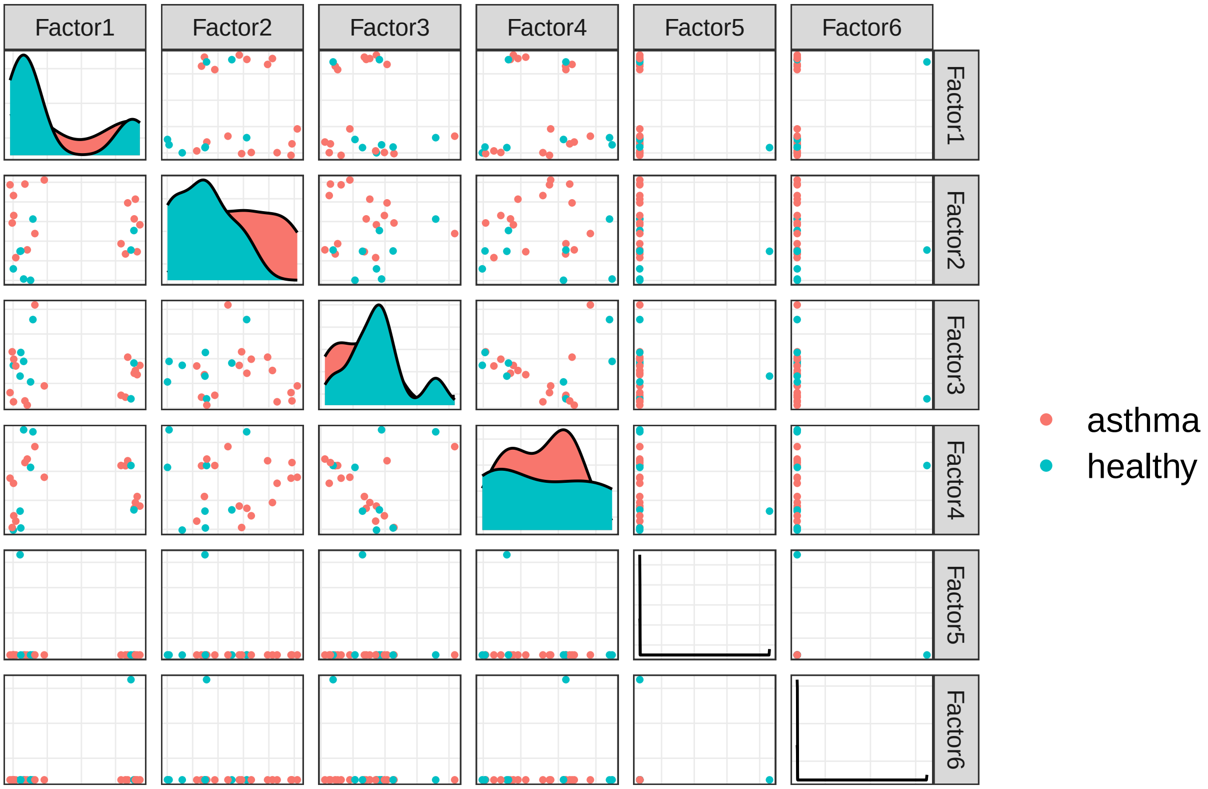

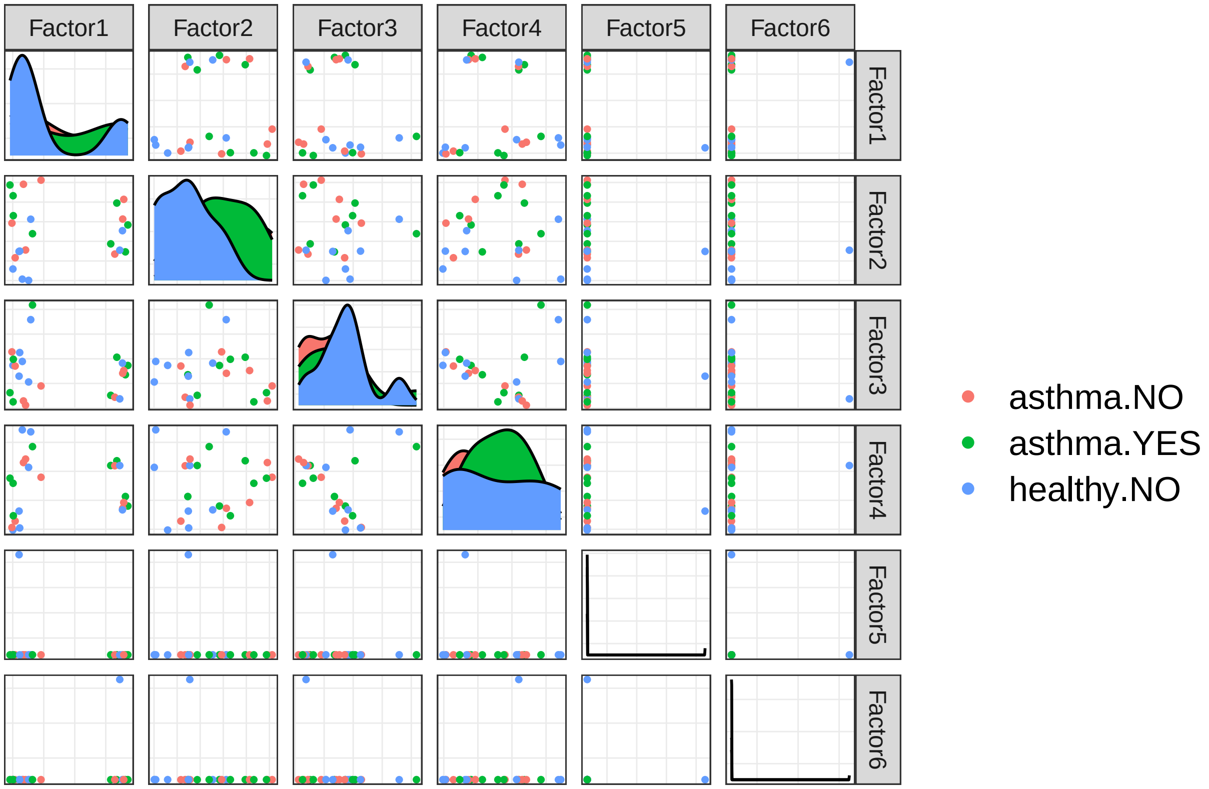
Factor1
Factor2
Factor3
Factor4
Factor5
Factor6
asthma.NO
asthma.YES
healthy.NO

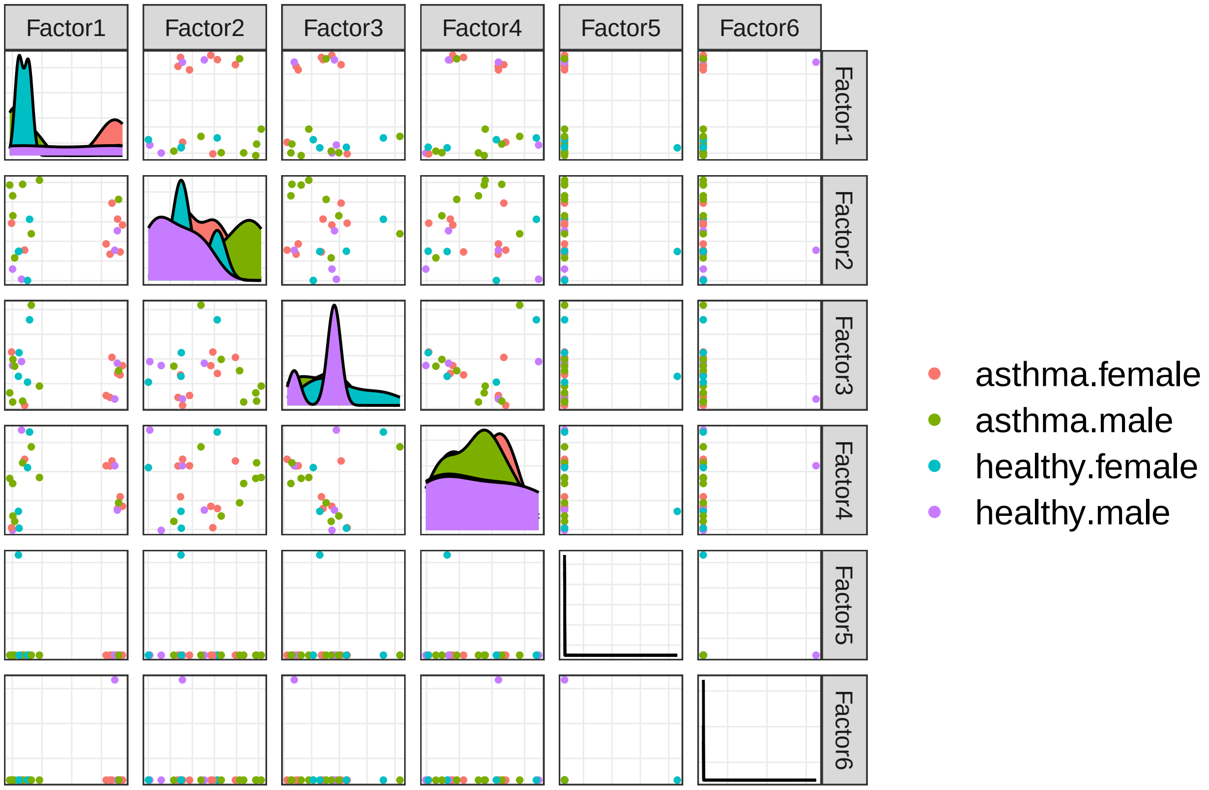
Factor1
Factor2
Factor3
Factor4
Factor5
Factor6
asthma.female
asthma.male
healthy.female
healthy.male

3.5 Plot weights

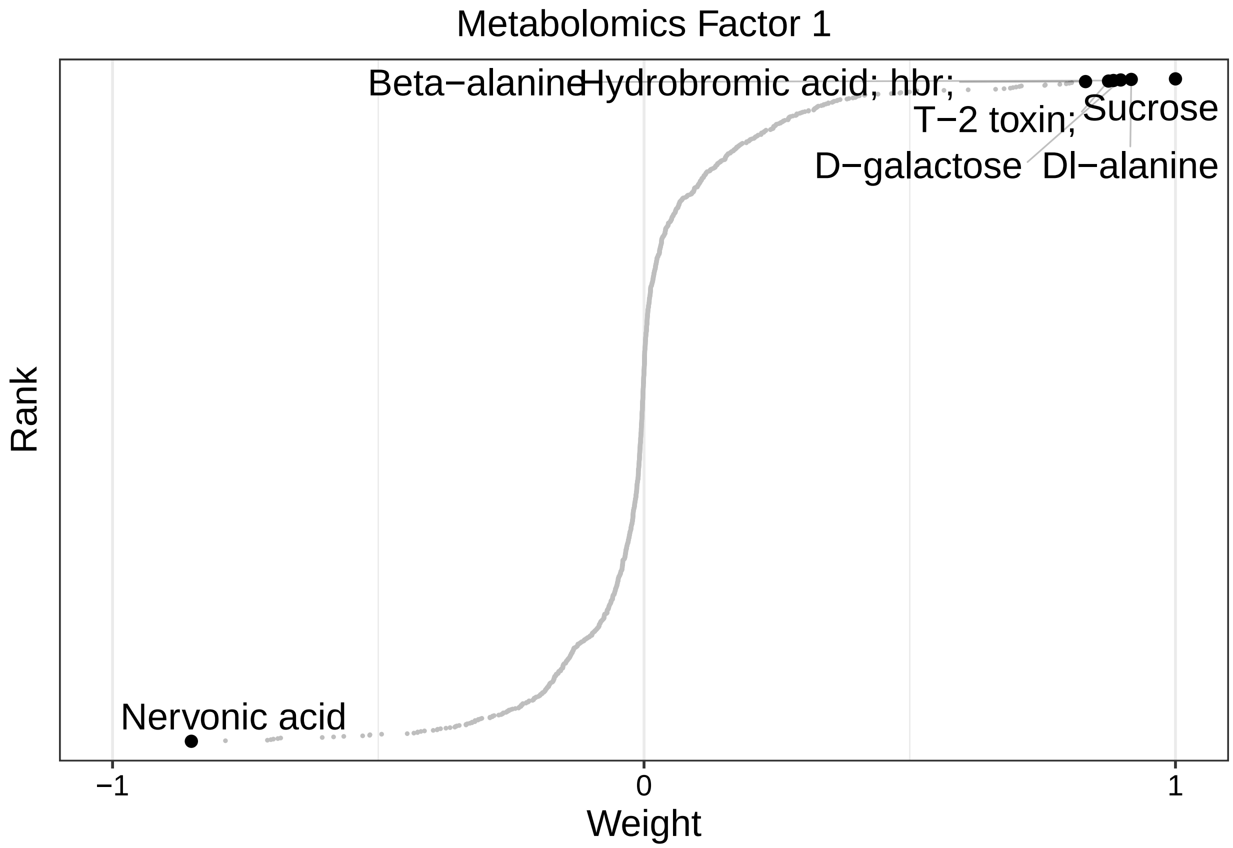

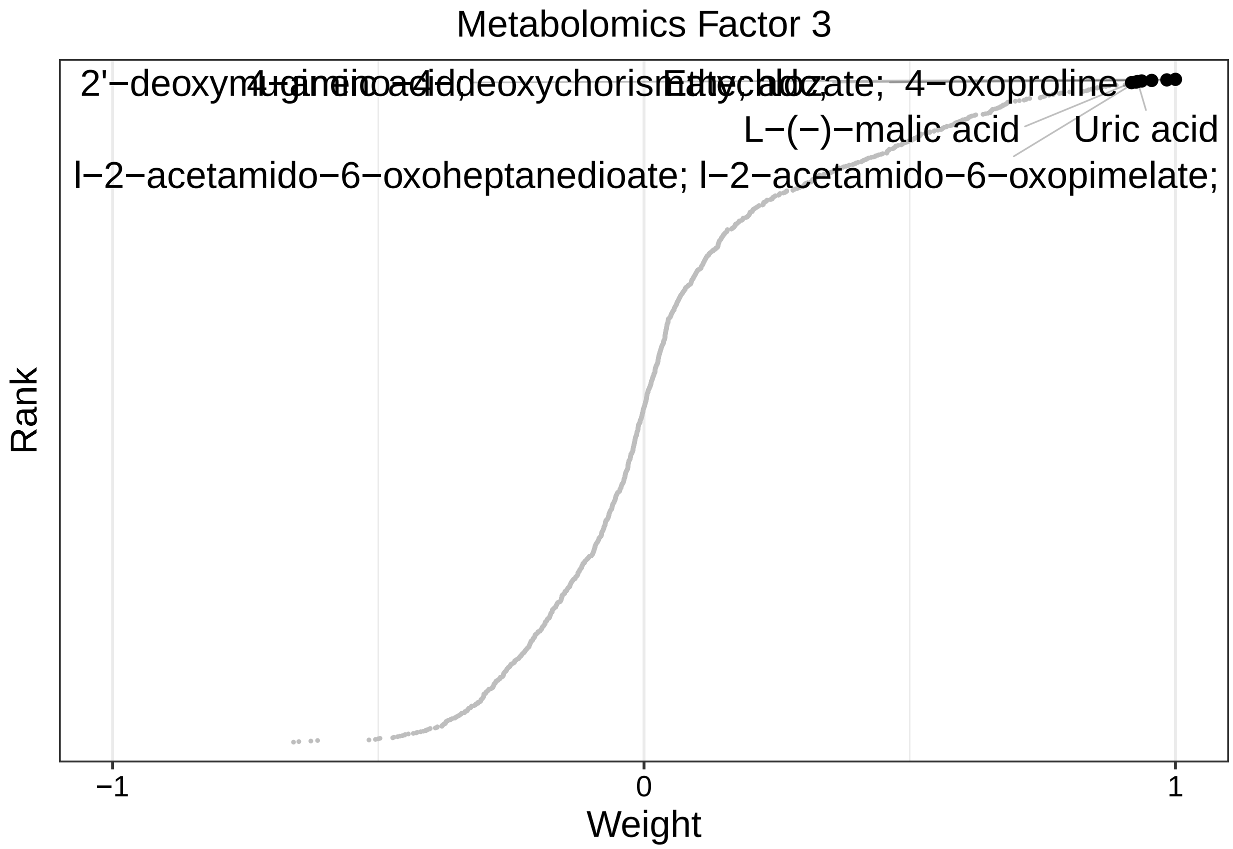
Metabolomics Factor 3
4−oxoproline
L−(−)−malic acid
Uric acid
l−2−acetamido−6−oxoheptanedioate; l−2−acetamido−6−oxopimelate;
Rank
−1
0
1
Weight

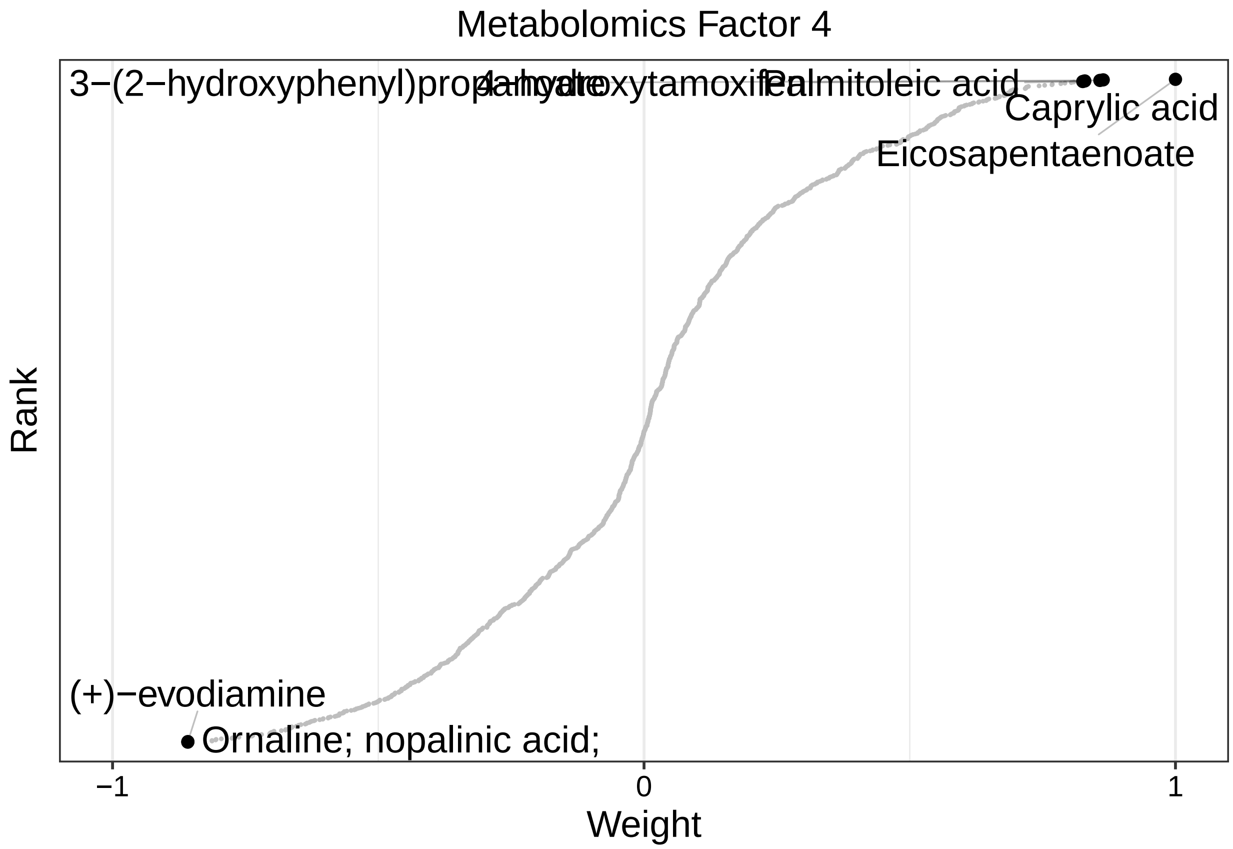
Metabolomics Factor 4
Caprylic acid
Eicosapentaenoate
(+)−evodiamine
Ornaline; nopalinic acid;
Rank
−1
0
1
Weight

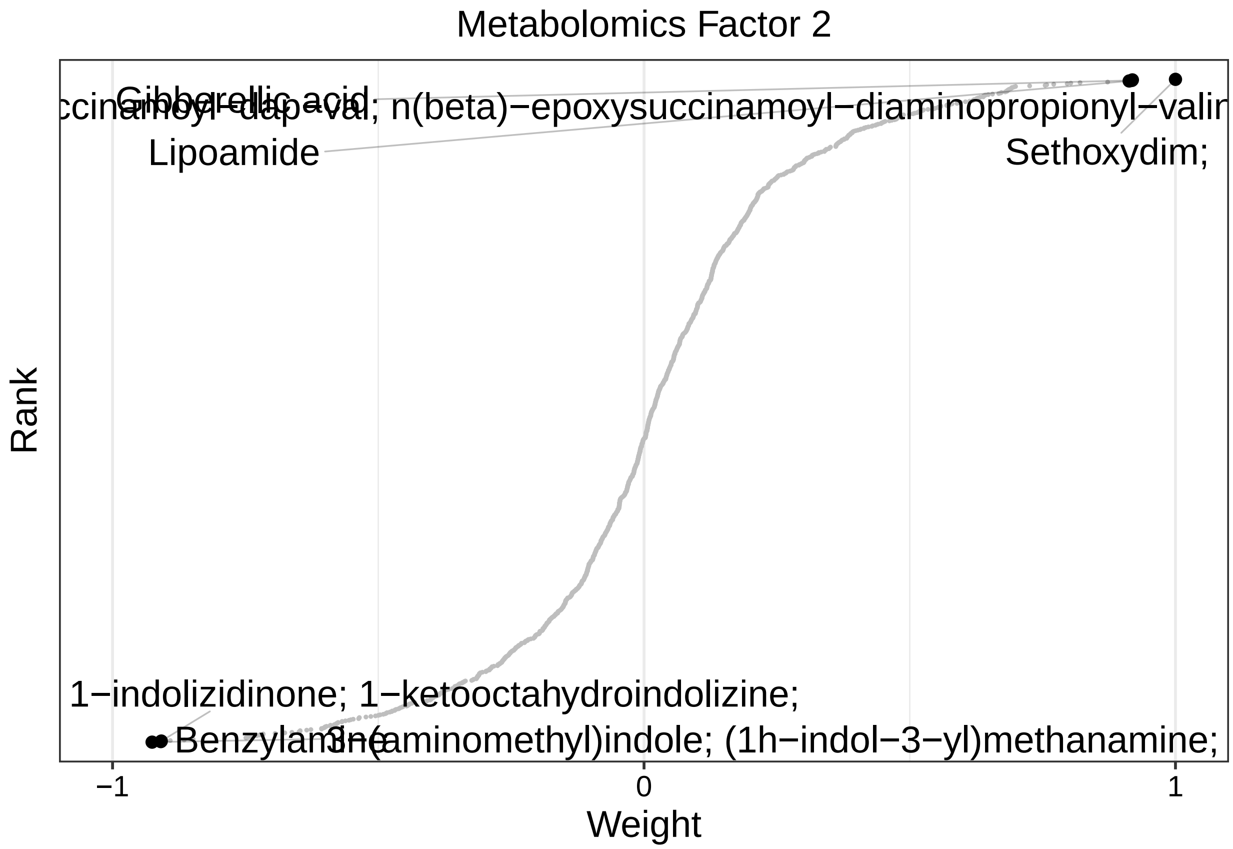
Metabolomics Factor 2
Gibberellic acid;
n(beta)−epoxysuccinamoyl−diaminopropionyl−valin
Lipoamide
Sethoxydim;
1−indolizidinone; 1−ketooctahydroindolizine;
3−(aminomethyl)indole; (1h−indol−3−yl)methanamine;
Rank
−1
0
1
Weight

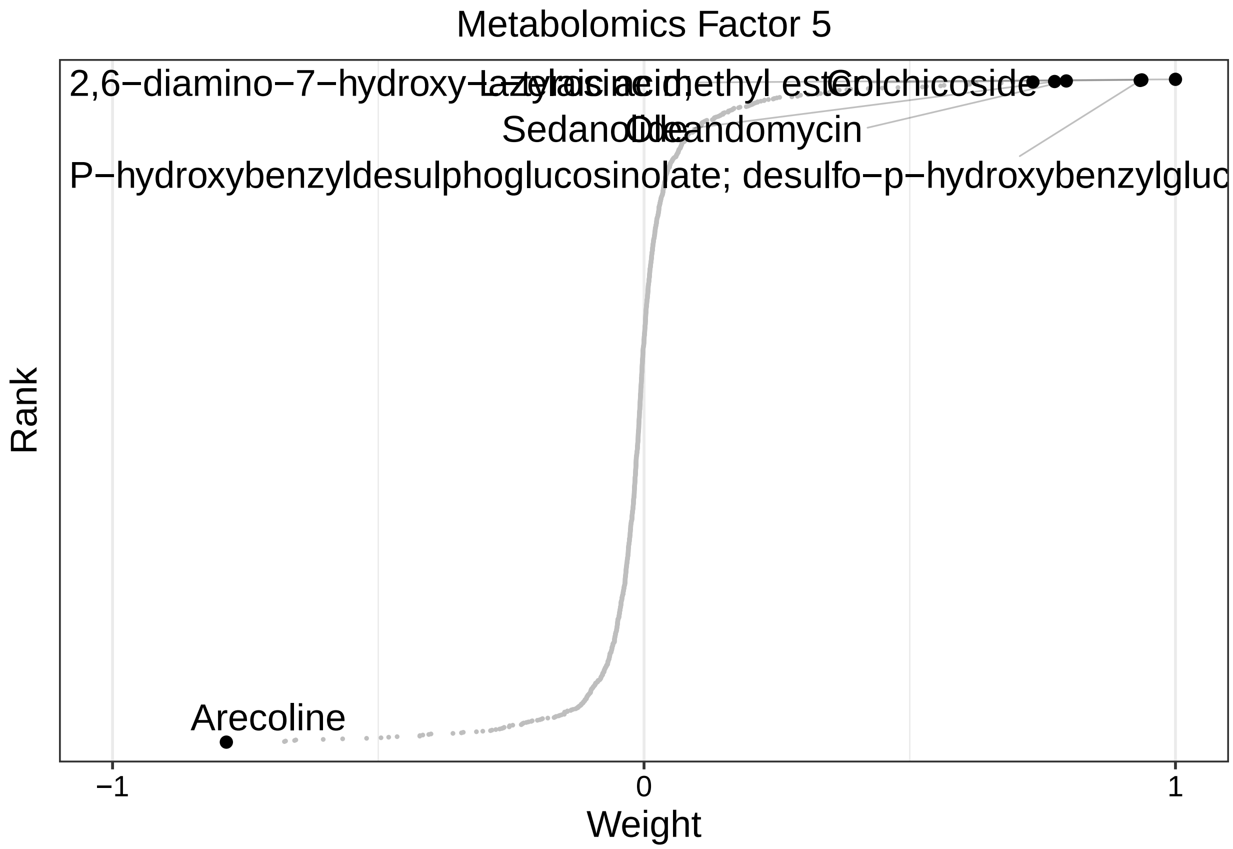
Metabolomics Factor 5
2,6−diamino−7−hydroxy−azelaic acid;
Colchicoside
Oleandomycin
P−hydroxybenzyldesulphoglucosinolate; desulfo−p−hydroxybenzylgluc
Arecoline
Rank
−1
0
1
Weight

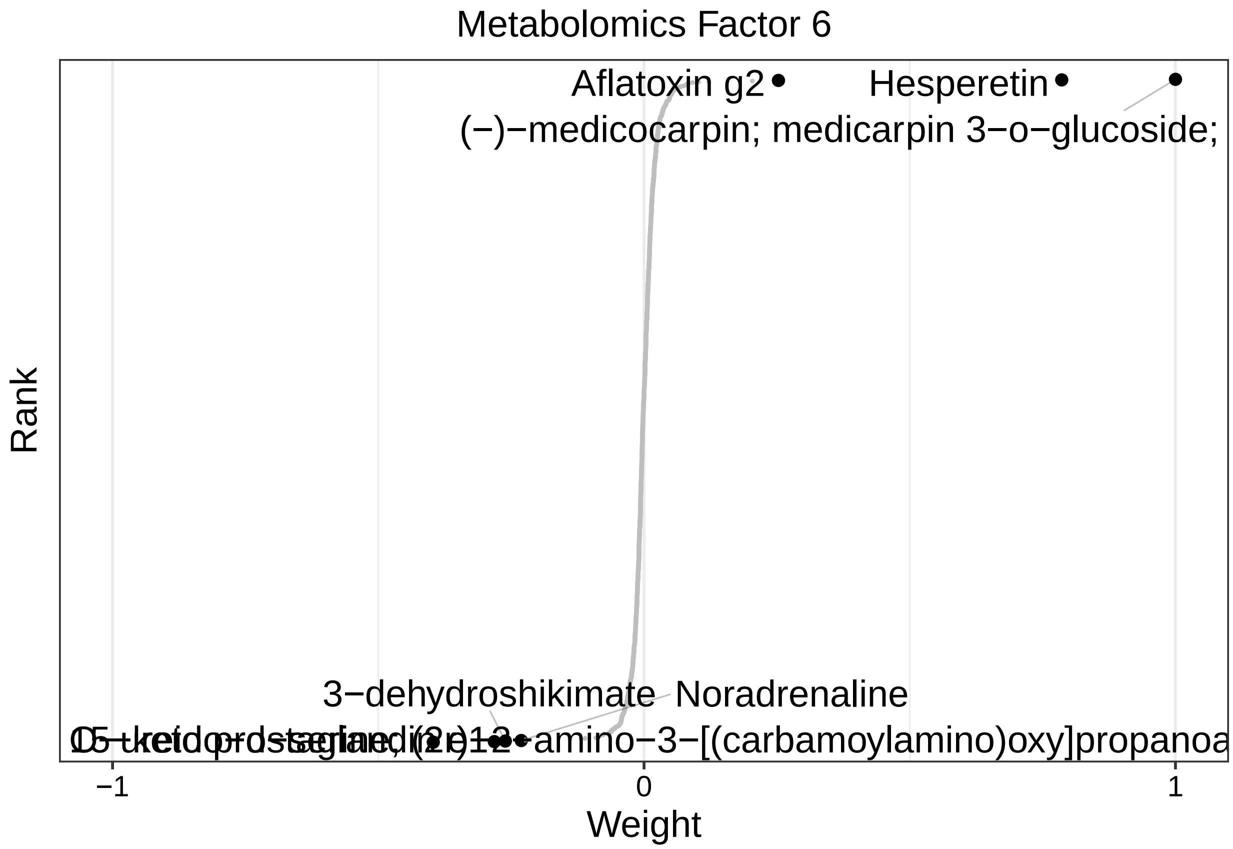
Metabolomics Factor 6
Aflatoxin g2
Hesperetin
(−)−medicocarpin; medicarpin 3−o−glucoside;
3−dehydroshikimate
Noradrenaline
amino−3−[(carbamoylamino)oxy]propanoa
Rank
−1
0
1
Weight

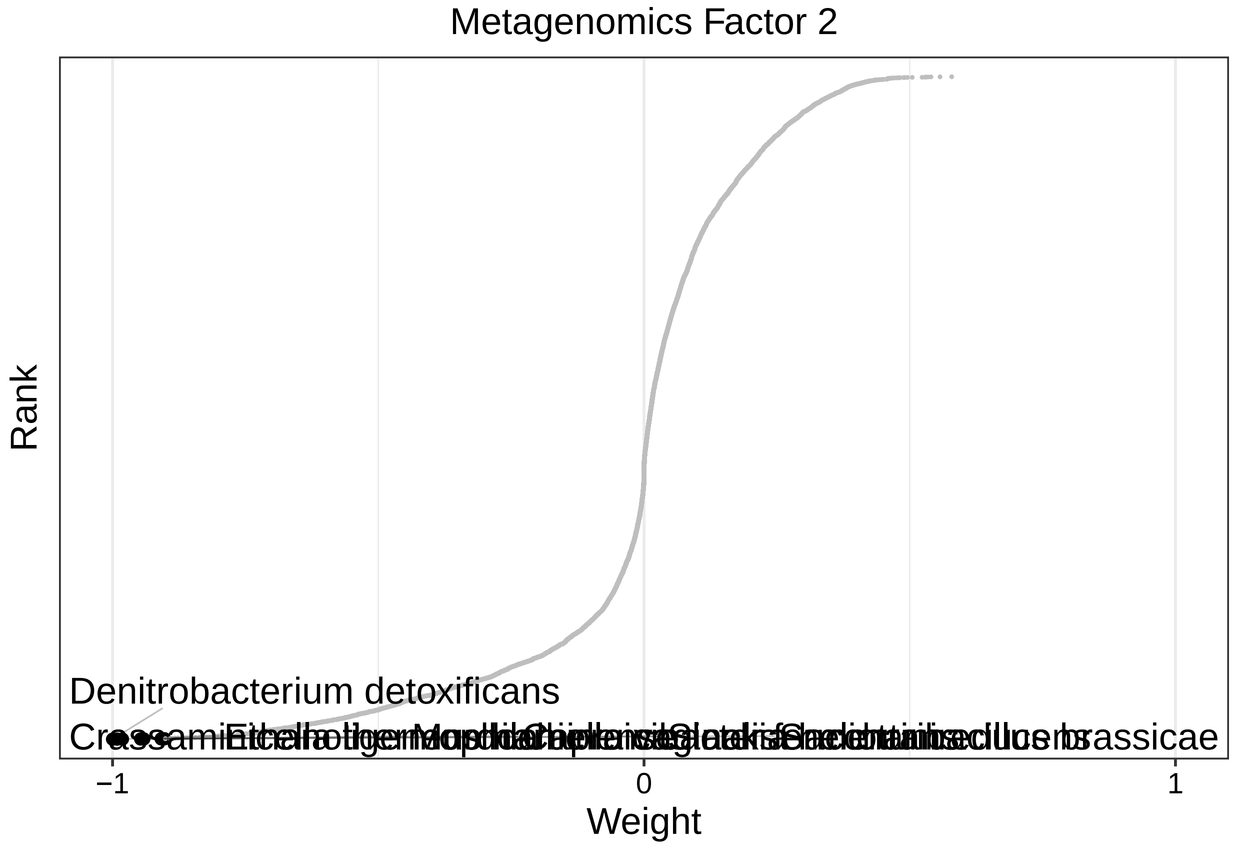
Metagenomics Factor 2
Denitrobacterium detoxificans
brassicae
Rank
−1
0
1
Weight

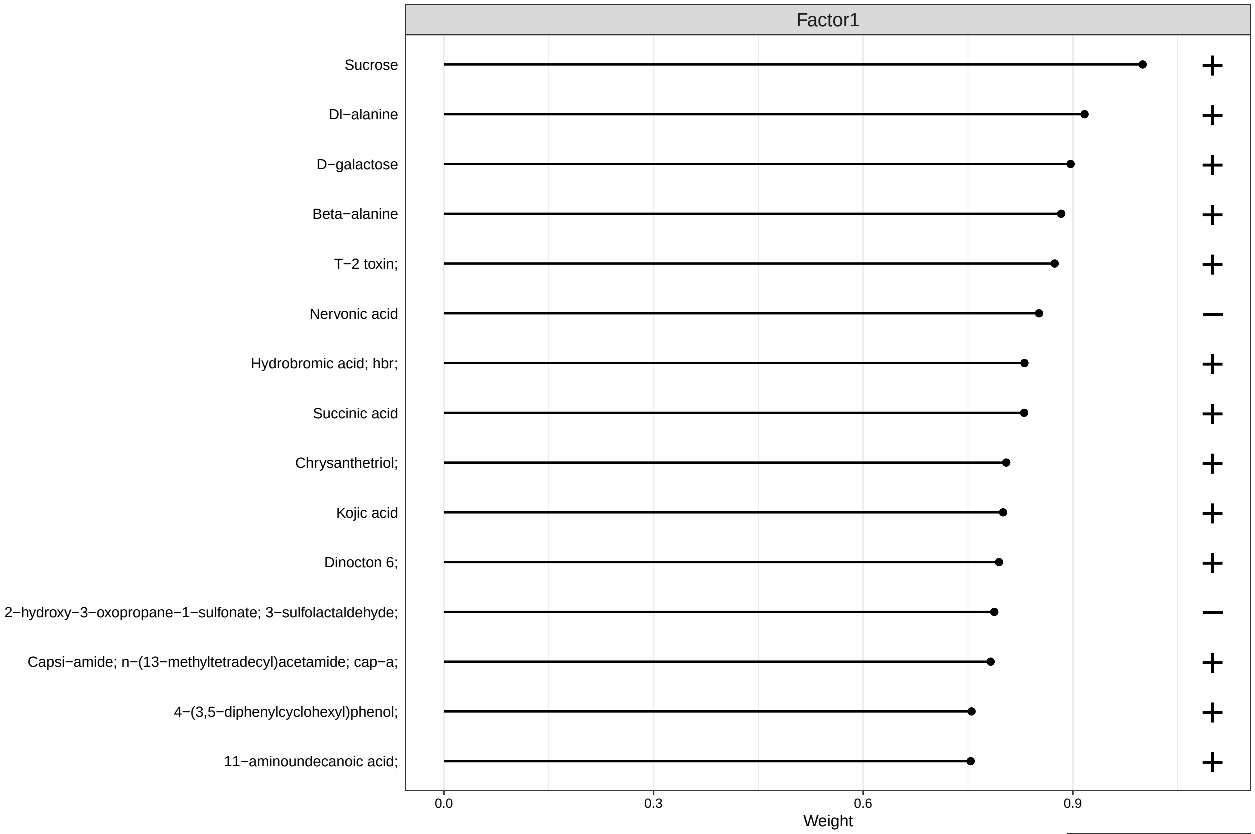
Factor1
Sucrose
Dl−alanine
D−galactose
Beta−alanine
T−2 toxin;
Nervonic acid
Hydrobromic acid; hbr;
Succinic acid
Chrysanthetriol;
Kojic acid
Dinocton 6;
2−hydroxy−3−oxopropane−1−sulfonate; 3−sulfolactaldehyde;
Capsi−amide; n−(13−methyltetradecyl)acetamide; cap−a;
4−(3,5−diphenylcyclohexyl)phenol;
11−aminoundecanoic acid;
0.0
0.3
0.6
0.9
Weight

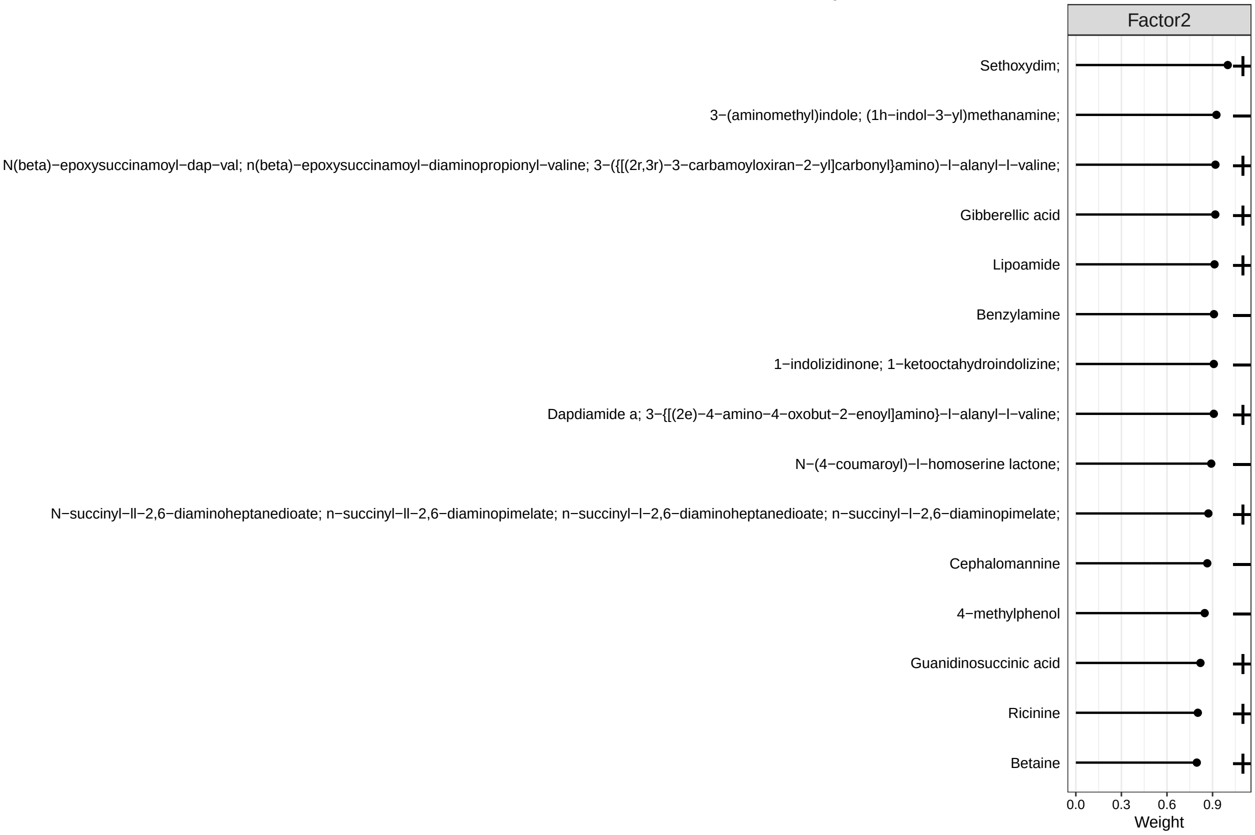
Factor2
Sethoxydim;
3−(aminomethyl)indole; (1h−indol−3−yl)methanamine;
N(beta)−epoxysuccinamoyl−dap−val; n(beta)−epoxysuccinamoyl−diaminopropionyl−valine; 3−({[(2r,3r)−3−carbamoyloxiran−2−yl]carbonyl}amino)−l−alanyl−l−valine;
Gibberellic acid
Lipoamide
Benzylamine
1−indolizidinone; 1−ketooctahydroindolizine;
Dapdiamide a; 3−{[(2e)−4−amino−4−oxobut−2−enoyl]amino}−l−alanyl−l−valine;
N−(4−coumaroyl)−l−homoserine lactone;
N−succinyl−ll−2,6−diaminoheptanedioate; n−succinyl−ll−2,6−diaminopimelate; n−succinyl−l−2,6−diaminoheptanedioate; n−succinyl−l−2,6−diaminopimelate;
Cephalomannine
4−methylphenol
Guanidinosuccinic acid
Ricinine
Betaine
0.0
0.3
0.6
0.9
Weight

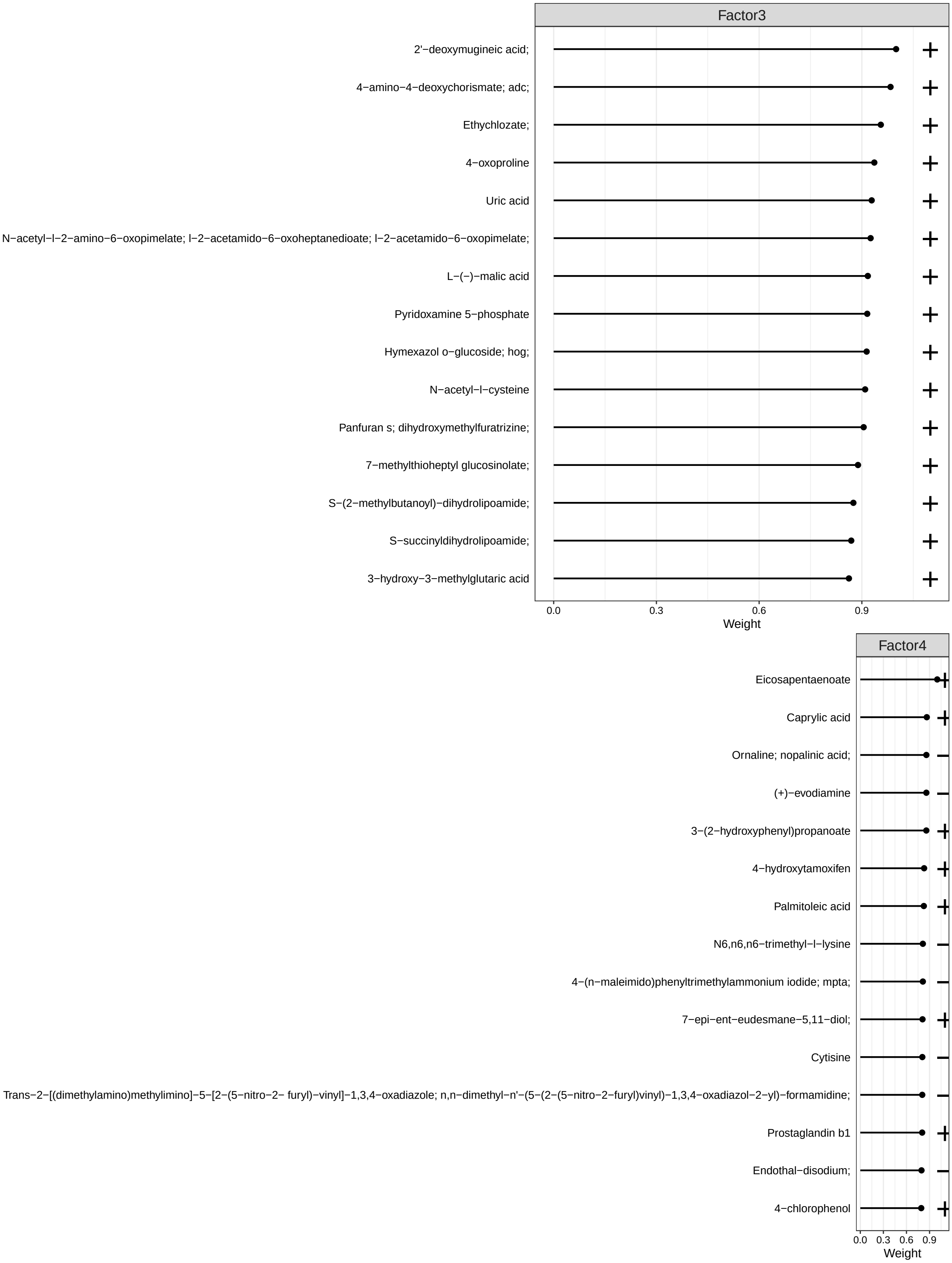
Factor3
2'−deoxymugineic acid;
4−amino−4−deoxychorismate; adc;
Ethychlozate;
4−oxoproline
Uric acid
N−acetyl−l−2−amino−6−oxopimelate; l−2−acetamido−6−oxoheptanedioate; l−2−acetamido−6−oxopimelate;
L−(−)−malic acid
Pyridoxamine 5−phosphate
Hymexazol o−glucoside; hog;
N−acetyl−l−cysteine
Panfuran s; dihydroxymethylfuratrizine;
7−methylthioheptyl glucosinolate;
S−(2−methylbutanoyl)−dihydrolipoamide;
S−succinyldihydrolipoamide;
3−hydroxy−3−methylglutaric acid
0.0
0.3
0.6
0.9
Weight
Factor4
Eicosapentaenoate
Caprylic acid
Ornaline; nopalinic acid;
(+)−evodiamine
3−(2−hydroxyphenyl)propanoate
4−hydroxytamoxifen
Palmitoleic acid
N6,n6,n6−trimethyl−l−lysine
4−(n−maleimido)phenyltrimethylammonium iodide; mpta;
7−epi−ent−eudesmane−5,11−diol;
Cytisine
Trans−2−[(dimethylamino)methylimino]−5−[2−(5−nitro−2− furyl)−vinyl]−1,3,4−oxadiazole; n,n−dimethyl−n'−(5−(2−(5−nitro−2−furyl)vinyl)−1,3,4−oxadiazol−2−yl)−formamidine;
Prostaglandin b1
Endothal−disodium;
4−chlorophenol
0.0
0.3
0.6
0.9
Weight

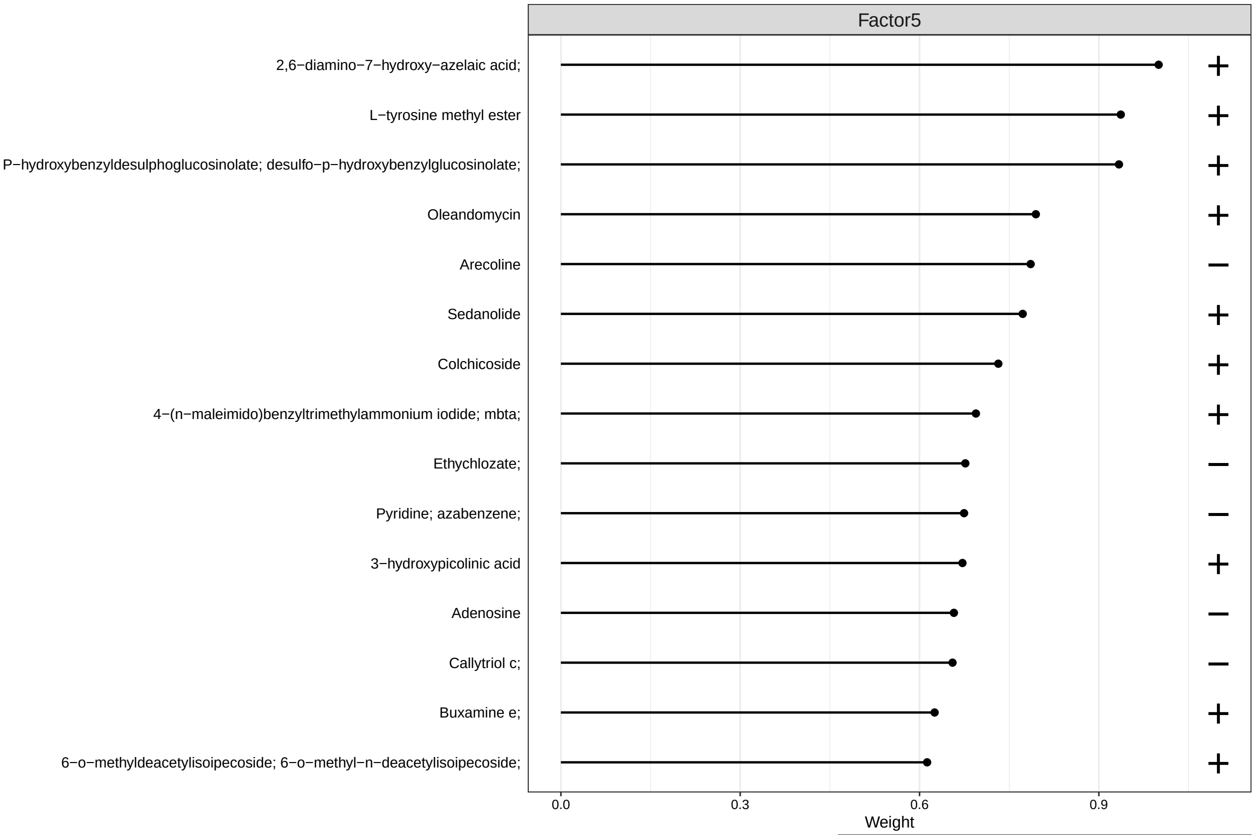

Factor5
2,6−diamino−7−hydroxy−azelaic acid;
L−tyrosine methyl ester
P−hydroxybenzyldesulphoglucosinolate; desulfo−p−hydroxybenzylglucosinolate;
Oleandomycin
Arecoline
Sedanolide
Colchicoside
4−(n−maleimido)benzyltrimethylammonium iodide; mbta;
Ethychlozate;
Pyridine; azabenzene;
3−hydroxypicolinic acid
Adenosine
Callytriol c;
Buxamine e;
6−o−methyldeacetylisoipecoside; 6−o−methyl−n−deacetylisoipecoside;
0.0
0.3
0.6
0.9
Weight

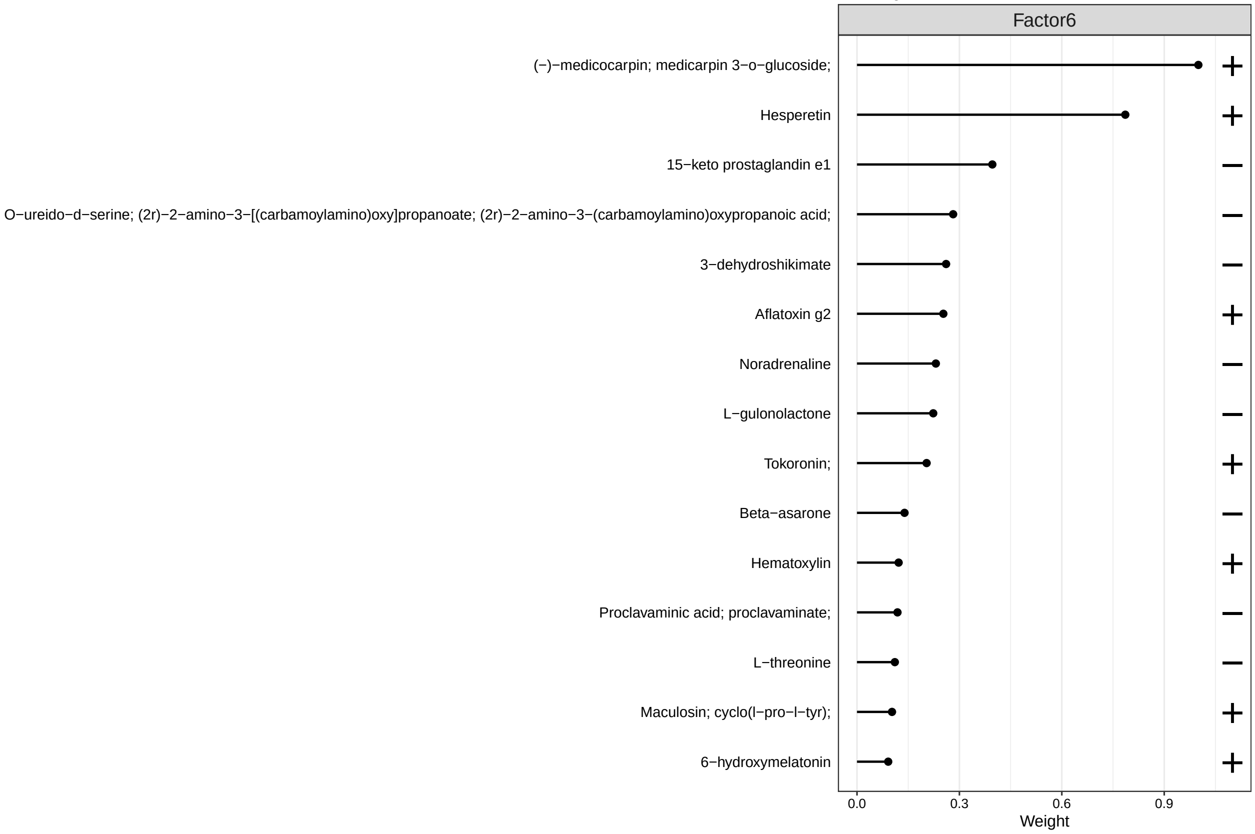

Factor6
(−)−medicocarpin; medicarpin 3−o−glucoside;
Hesperetin
15−keto prostaglandin e1
O−ureido−d−serine; (2r)−2−amino−3−[(carbamoylamino)oxy]propanoate; (2r)−2−amino−3−(carbamoylamino)oxypropanoic acid;
3−dehydroshikimate
Aflatoxin g2
Noradrenaline
L−gulonolactone
Tokoronin;
Beta−asarone
Hematoxylin
Proclavaminic acid; proclavaminate;
L−threonine
Maculosin; cyclo(l−pro−l−tyr);
6−hydroxymelatonin
0.0
0.3
0.6
0.9
Weight

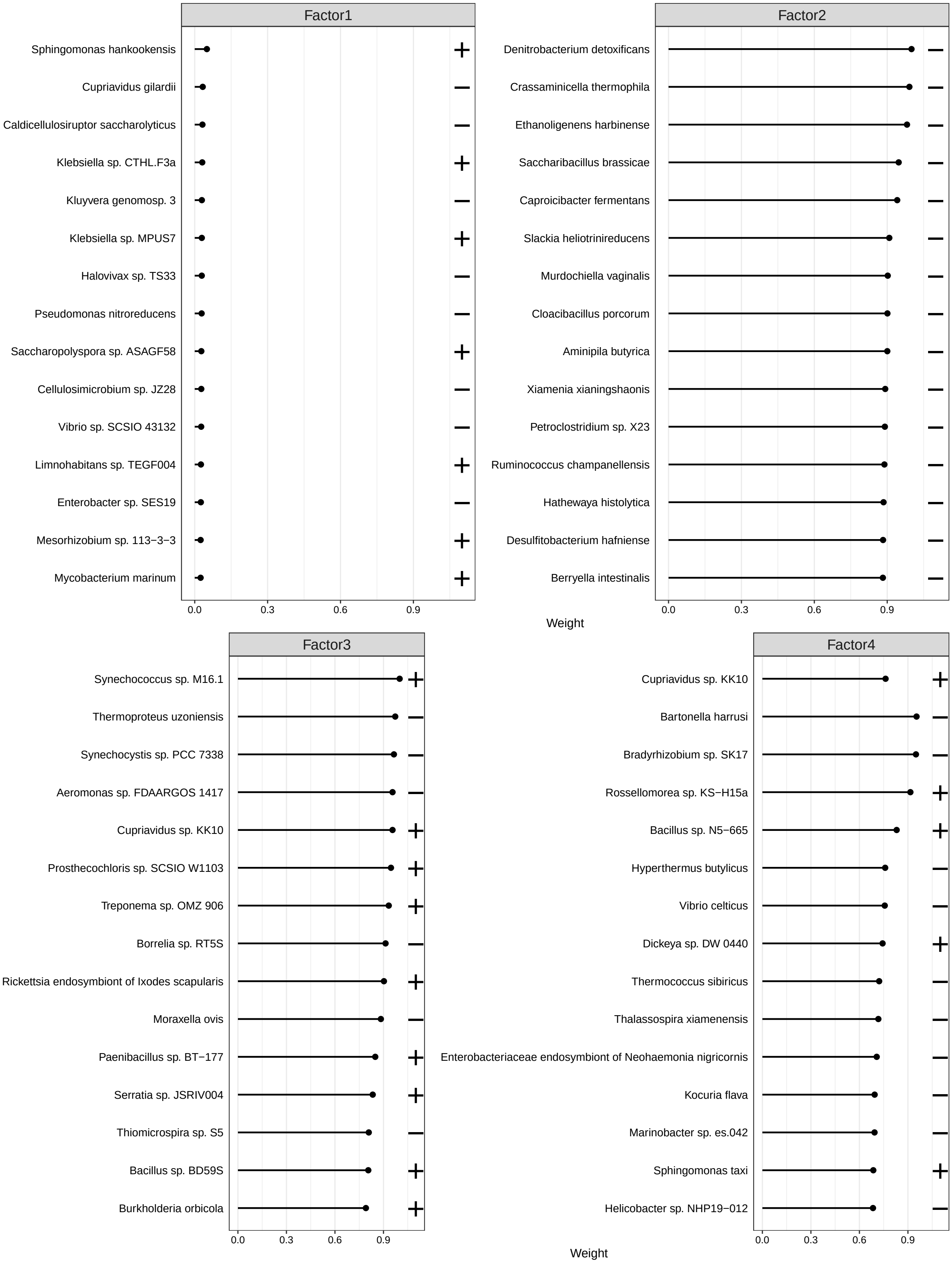
Factor1
Sphingomonas hankookensis
Cupriavidus gilardii
Caldicellulosiruptor saccharolyticus
Klebsiella sp. CTHL.F3a
Kluyvera genomosp. 3
Klebsiella sp. MPUS7
Halovivax sp. TS33
Pseudomonas nitroreducens
Saccharopolyspora sp. ASAGF58
Cellulosimicrobium sp. JZ28
Vibrio sp. SCSIO 43132
Limnohabitans sp. TEGF004
Enterobacter sp. SES19
Mesorhizobium sp. 113−3−3
Mycobacterium marinum
Factor2
Denitrobacterium detoxificans
Crassaminicella thermophila
Ethanoligenens harbinense
Saccharibacillus brassicae
Caproicibacter fermentans
Slackia heliotrinireducens
Murdochiella vaginalis
Cloacibacillus porcorum
Aminipila butyrica
Xiamenia xianingshaonis
Petroclostridium sp. X23
Ruminococcus champanellensis
Hathewaya histolytica
Desulfitobacterium hafniense
Berryella intestinalis
0.0
0.3
0.6
0.9
Weight
Factor3
Synechococcus sp. M16.1
Thermoproteus uzoniensis
Synechocystis sp. PCC 7338
Aeromonas sp. FDAARGOS 1417
Cupriavidus sp. KK10
Prosthecochloris sp. SCSIO W1103
Treponema sp. OMZ 906
Borrelia sp. RT5S
Rickettsia endosymbiont of Ixodes scapularis
Moraxella ovis
Paenibacillus sp. BT−177
Serratia sp. JSRIV004
Thiomicrospira sp. S5
Bacillus sp. BD59S
Burkholderia orbicola
Factor4
Cupriavidus sp. KK10
Bartonella harrusi
Bradyrhizobium sp. SK17
Rossellomorea sp. KS−H15a
Bacillus sp. N5−665
Hyperthermus butylicus
Vibrio celticus
Dickeya sp. DW 0440
Thermococcus sibiricus
Thalassospira xiamenensis
Enterobacteriaceae endosymbiont of Neohaemonia nigricornis
Kocuria flava
Marinobacter sp. es.042
Sphingomonas taxi
Helicobacter sp. NHP19−012
0.0
0.3
0.6
0.9
Weight

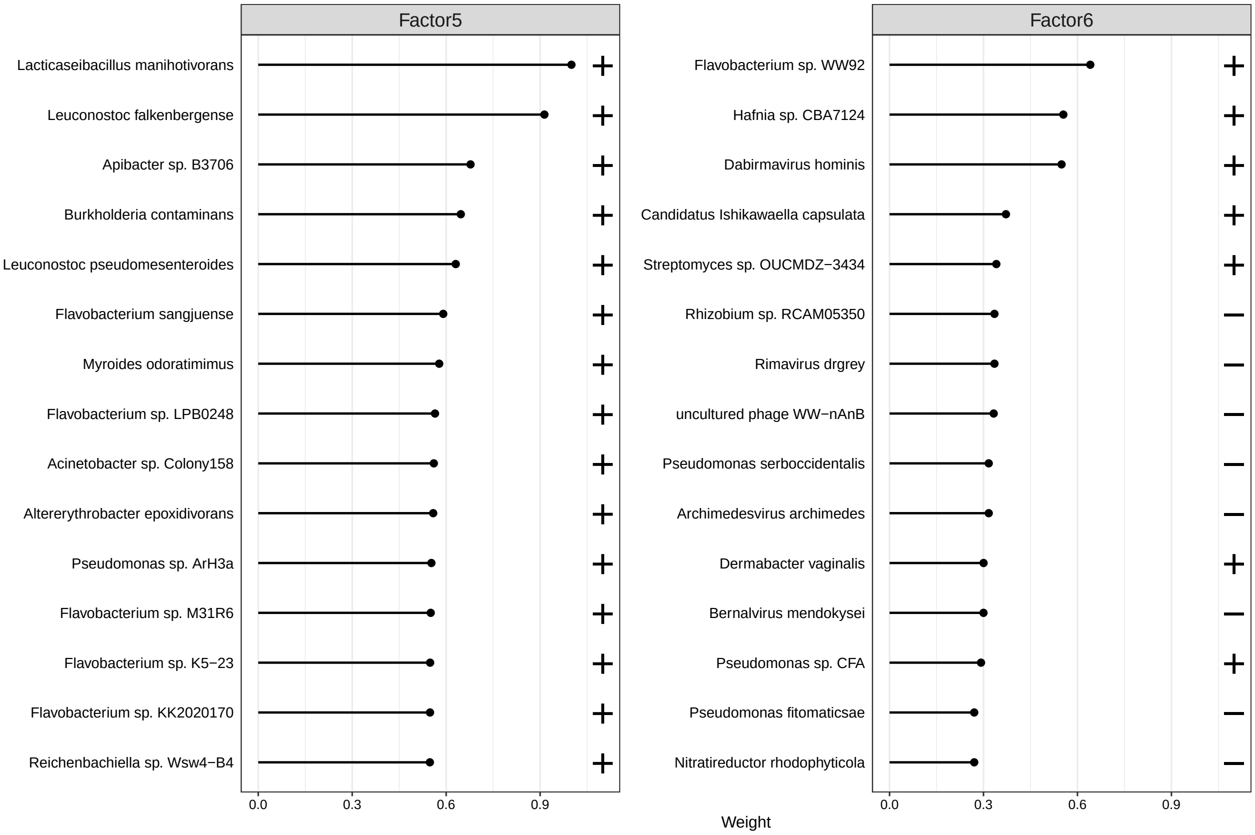
Factor5
Lacticaseibacillus manihotivorans
Leuconostoc falkenbergense
Apibacter sp. B3706
Burkholderia contaminans
Leuconostoc pseudomesenteroides
Flavobacterium sangjuense
Myroides odoratimimus
Flavobacterium sp. LPB0248
Acinetobacter sp. Colony158
Altererythrobacter epoxidivorans
Pseudomonas sp. ArH3a
Flavobacterium sp. M31R6
Flavobacterium sp. K5−23
Flavobacterium sp. KK2020170
Reichenbachiella sp. Wsw4−B4
Factor6
Flavobacterium sp. WW92
Hafnia sp. CBA7124
Dabirmavirus hominis
Candidatus Ishikawaella capsulata
Streptomyces sp. OUCMDZ−3434
Rhizobium sp. RCAM05350
Rimavirus drgrey
uncultured phage WW−nAnB
Pseudomonas serboccidentalis
Archimedesvirus archimedes
Dermabacter vaginalis
Bernalvirus mendokysei
Pseudomonas sp. CFA
Pseudomonas fitomaticsae
Nitratireductor rhodophyticola
0.0
0.3
0.6
0.9
Weight

3.6 UMAP projection

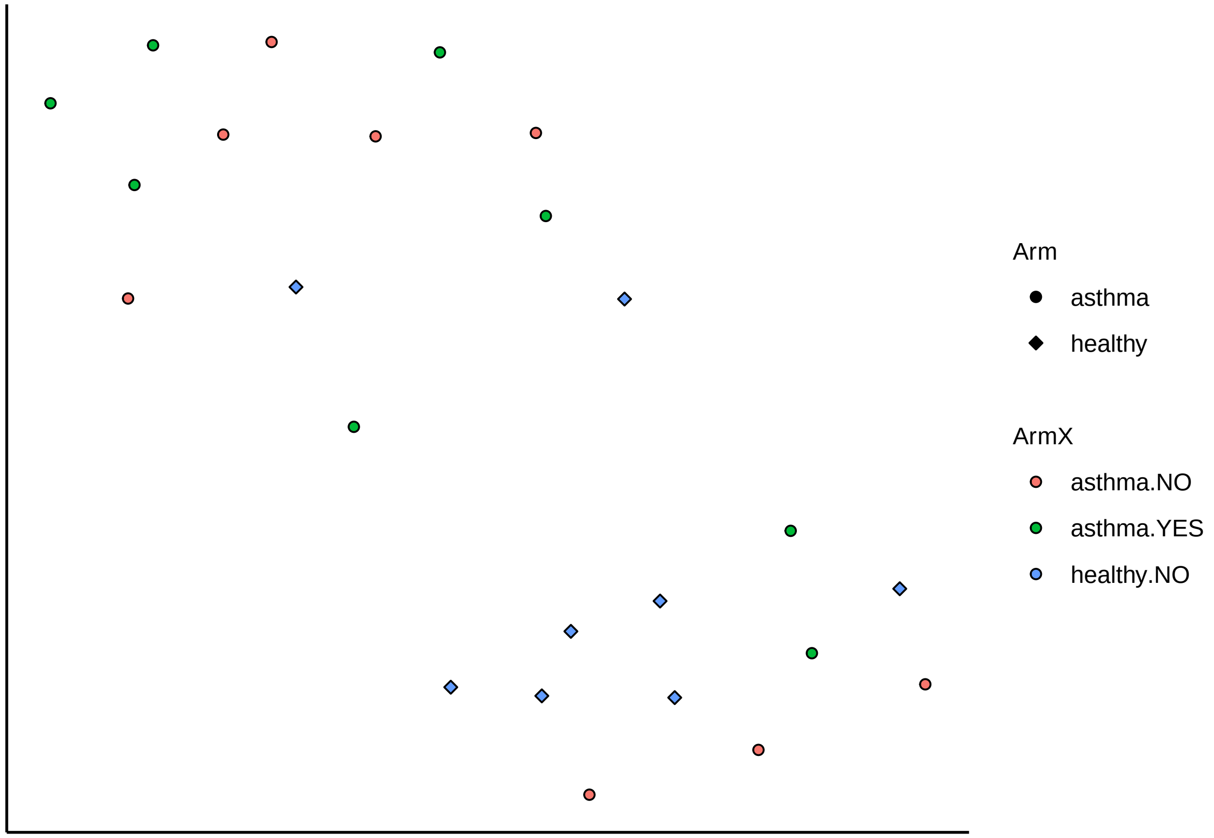

3.7 Pathway analysis

3.7.1 Factor1 positive pathways with feature weight >=0.5

[1] 24

Var1	Freq
map01100 Metabolic pathways	10
map00052 Galactose metabolism	3
map02010 ABC transporters	3
map04973 Carbohydrate digestion and absorption	3
map00360 Phenylalanine metabolism	2
map00640 Propanoate metabolism	2
map04080 Neuroactive ligand-receptor interaction	2
map00020 Citrate cycle (TCA cycle)	1
map00190 Oxidative phosphorylation	1
map00240 Pyrimidine metabolism	1
map00250 Alanine, aspartate and glutamate metabolism	1
map00260 Glycine, serine and threonine metabolism	1
map00310 Lysine degradation	1
map00330 Arginine and proline metabolism	1

Var1	Freq
map00350 Tyrosine metabolism	1
map00410 beta-Alanine metabolism	1
map00500 Starch and sucrose metabolism	1
map00520 Amino sugar and nucleotide sugar metabolism	1
map00620 Pyruvate metabolism	1
map00630 Glyoxylate and dicarboxylate metabolism	1
map00650 Butanoate metabolism	1
map00760 Nicotinate and nicotinamide metabolism	1
map00770 Pantothenate and CoA biosynthesis	1
map00920 Sulfur metabolism	1
map01200 Carbon metabolism	1
map01210 2-Oxocarboxylic acid metabolism	1
map04024 cAMP signaling pathway	1
map04714 Thermogenesis	1
map04723 Retrograde endocannabinoid signaling	1
map04727 GABAergic synapse	1
map04742 Taste transduction	1
map04750 Inflammatory mediator regulation of TRP channels	1
map04922 Glucagon signaling pathway	1
map04974 Protein digestion and absorption	1
map04976 Bile secretion	1
map05230 Central carbon metabolism in cancer	1

Таблица 8: Table, Features with positive weight >= 0.5 (24)

Name	weight	Formula	Ion.Mode	KEGG.ID	Sub.Pathway	Pathway
Sucrose	1.000	C12 H22 O11	NEG	C00089	Galactose metabolism	map00052 Galactose metabolism; map00500 Starch and sucrose metabolism; map01100 Metabolic pathways; map02010 ABC transporters; map04742 Taste transduction; map04973 Carbohydrate digestion and absorption;
Dl-alanine	0.917	C3 H7 N O2	NEG	C01401	Cyanoamino acid metabolism	map01100 Metabolic pathways;
D-galactose	0.897	C6 H12 O6	POS	C00984	Galactose metabolism	map00052 Galactose metabolism; map00520 Amino sugar and nucleotide sugar metabolism; map01100 Metabolic pathways;
Beta-alanine	0.883	C3 H7 N O2	POS	C00099	Pyrimidine metabolism	map00240 Pyrimidine metabolism; map00410 beta-Alanine metabolism; map00640 Propanoate metabolism; map00770 Pantothenate and CoA biosynthesis; map01100 Metabolic pathways; map04080 Neuroactive ligand-receptor interaction; map04974 Protein digestion and absorption;
T-2 toxin;	0.874	C24 H34 O9	NEG	C09738		
Hydrobromic acid; hbr;	0.831	H Br	NEG	C13645		

Continued on next page

Name	weight	Formula	Ion.Mode	KEGG.ID	Sub.Pathway	Pathway
Succinic acid	0.830	C4 H6 O4	NEG	C00042	Citrate cycle (TCA cycle)	map00020 Citrate cycle (TCA cycle); map00190 Oxidative phosphorylation; map00250 Alanine, aspartate and glutamate metabolism; map00310 Lysine degradation; map00350 Tyrosine metabolism; map00360 Phenylalanine metabolism; map00620 Pyruvate metabolism; map00630 Glyoxylate and dicarboxylate metabolism; map00640 Propanoate metabolism; map00650 Butanoate metabolism; map00760 Nicotinate and nicotinamide metabolism; map00920 Sulfur metabolism; map01100 Metabolic pathways; map01200 Carbon metabolism; map04024 cAMP signaling pathway; map04727 GABAergic synapse; map04922 Glucagon signaling pathway; map05230 Central carbon metabolism in cancer;
Chrysanthetriol;	0.805	C15 H26 O3	POS	C17610		
Kojic acid	0.800	C6 H6 O4	POS	C14516		
Dinocton 6;	0.794	C16 H22 N2 O7	NEG	C18922		
Capsi-amide; n-(13-methyltetradecyl)acetamide; cap-a;	0.782	C17 H35 N O	POS	C17515		

Continued on next page

Name	weight	Formula	Ion.Mode	KEGG.ID	Sub.Pathway	Pathway
4-(3,5-diphenylcyclohexyl)phenol;	0.755	C24 H24 O	POS	C14276		
11-aminoundecanoic acid;	0.754	C11 H23 N O2	POS	C19325		
Maltotriose	0.710	C18 H32 O16	POS	C01835		map02010 ABC transporters; map04973 Carbohydrate digestion and absorption;
8-hydroxyalanylclavam;	0.706	C8 H12 N2 O5	POS	C17359	Biosynthesis of antibiotics	
Cis-(homo)3-aconitate;	0.700	C9 H12 O6	POS	C20582	Methane metabolism	map01100 Metabolic pathways; map01210 2-Oxocarboxylic acid metabolism;
Adenosylhopane;	0.694	C40 H63 N5 O3	POS	C21117		
Salicylate	0.689	C7 H6 O3	NEG	C00805	Phenylalanine metabolism	map00360 Phenylalanine metabolism; map01100 Metabolic pathways; map04976 Bile secretion;
2-aminobutyric acid	0.677	C4 H9 N O2	POS	C02261		
D(-)-amygdalin	0.662	C20 H27 N O11	NEG	C08325	Cyanoamino acid metabolism	map01100 Metabolic pathways;
Anandamide (aea)	0.610	C22 H37 N O2	POS	C11695		map04080 Neuroactive ligand-receptor interaction; map04714 Thermogenesis; map04723 Retrograde endocannabinoid signaling; map04750 Inflammatory mediator regulation of TRP channels;
Guanidineacetic acid	0.564	C3 H7 N3 O2	POS	C00581	Glycine, serine and threonine metabolism	map00260 Glycine, serine and threonine metabolism; map00330 Arginine and proline metabolism; map01100 Metabolic pathways;

Continued on next page

Name	weight	Formula	Ion.Mode	KEGG.ID	Sub.Pathway	Pathway
Alpha-lactose	0.513	C12 H22 O11	POS	C00243	Galactose metabolism	map00052 Galactose metabolism; map01100 Metabolic pathways; map02010 ABC transporters; map04973 Carbohydrate digestion and absorption;
4-amino-2-hydroxylamino-6-nitrotoluene;	0.513	C7 H9 N3 O3	POS	C16403	Nitrotoluene degradation	

3.7.2 Factor1 negative pathways with feature weight <= -0.5

Var1	Freq
map01040 Biosynthesis of unsaturated fatty acids	7
map01100 Metabolic pathways	2
map00061 Fatty acid biosynthesis	1
map00062 Fatty acid elongation	1
map00071 Fatty acid degradation	1
map00380 Tryptophan metabolism	1
map01212 Fatty acid metabolism	1

Таблица 11: Table, Features with negative weight <= -0.5 (13)

Name	weight	Formula	Ion.Mode	KEGG.ID	Sub.Pathway	Pathway
Nervonic acid	-1	C24 H46 O2	NEG	C08323	Biosynthesis of unsaturated fatty acids	map01040 Biosynthesis of unsaturated fatty acids;
2-hydroxy-3-oxopropane-1-sulfonate; 3-sulfolactaldehyde;	-1	C3 H6 O5 S	NEG	C20798		
Arachidic acid	-1	C20 H40 O2	NEG	C06425	Biosynthesis of unsaturated fatty acids	map01040 Biosynthesis of unsaturated fatty acids;
Erucic acid	-1	C22 H42 O2	NEG	C08316	Biosynthesis of unsaturated fatty acids	map01040 Biosynthesis of unsaturated fatty acids;
Palmitic acid	-1	C16 H32 O2	NEG	C00249	Fatty acid biosynthesis	map00061 Fatty acid biosynthesis; map00062 Fatty acid elongation; map00071 Fatty acid degradation; map01040 Biosynthesis of unsaturated fatty acids; map01100 Metabolic pathways; map01212 Fatty acid metabolism;
Gallic acid	-1	C7 H6 O5	NEG	C01424	Aminobenzoate degradation	
Indole-3-acetic acid	-1	C10 H9 N O2	NEG	C00954	Tryptophan metabolism	map00380 Tryptophan metabolism; map01100 Metabolic pathways;
Docosanoic acid	-1	C22 H44 O2	NEG	C08281	Cutin, suberine and wax biosynthesis	map01040 Biosynthesis of unsaturated fatty acids;
Eicosapentaenoate	-1	C20 H30 O2	NEG	C06428	Biosynthesis of unsaturated fatty acids	map01040 Biosynthesis of unsaturated fatty acids;
Xanthohumol	-1	C21 H22 O5	NEG	C16417	Flavonoid biosynthesis	
Dimethirimol;	-1	C11 H19 N3 O	NEG	C18784		

Continued on next page

Name	weight	Formula	Ion.Mode	KEGG.ID	Sub.Pathway	Pathway
Lignoceric acid	-1	C24 H48 O2	NEG	C08320	Biosynthesis of unsaturated fatty acids	map01040 Biosynthesis of unsaturated fatty acids;
7-epi-ent-eudesmane-5,11-diol;	-1	C15 H28 O2	NEG	C22043		

3.7.3 Factor2 positive pathways with feature weight >=0.5

```
## [1] 9
```

Таблица 13: Table, Features with positive weight >= 0.5 (9)

order	family	genus	species	weight
Pseudomonadales	Pseudomonadaceae	Pseudomonas	Pseudomonas sp. BT-42-2	0.579
Enterobacterales	Erwiniaceae	Tatumella	Tatumella ptyseos	0.557
Mycobacteriales	Mycobacteriaceae	Mycolicibacterium	Mycolicibacterium aurum	0.540
Hyphomicrobiales	Rhizobiaceae	Agrobacterium	Agrobacterium cucumeris	0.534
Rhodobacterales	Roseobacteraceae	Roseibacterium	Roseibacterium elongatum	0.530
Pseudomonadales	Pseudomonadaceae	Pseudomonas	Pseudomonas sp. KU26590	0.529
Burkholderiales	Comamonadaceae	Rhodoferax	Rhodoferax aquaticus	0.529
Alteromonadales	Shewanellaceae	Parashewanella	Parashewanella spongiae	0.523
Oceanospirillales	Halomonadaceae	Halomonas	Halomonas sp. CKK8	0.505

3.7.4 Factor2 negative pathways with feature weight <= -0.5

Таблица 15: Table, Features with negative weight <= -0.5 (395)

order	family	genus	species	weight
Eggerthellales	Eggerthellaceae	Denitrobacterium	Denitrobacterium detoxificans	-1.000
Eubacteriales	Clostridiaceae	Crassaminicella	Crassaminicella thermophila	-0.991
Eubacteriales	Oscillospiraceae	Ethanoligenens	Ethanoligenens harbinense	-0.981
Bacillales	Paenibacillaceae	Saccharibacillus	Saccharibacillus brassicae	-0.947
Eubacteriales	Oscillospiraceae	Caproicibacter	Caproicibacter fermentans	-0.941
Eggerthellales	Eggerthellaceae	Slackia	Slackia heliotrinireducens	-0.909
Tissierellales	Peptoniphilaceae	Murdochiella	Murdochiella vaginalis	-0.902
Synergistales	Synergistaceae	Cloacibacillus	Cloacibacillus porcorum	-0.901
Eubacteriales	Eubacteriales Family XIII. Incertae Sedis	Aminipila	Aminipila butyrica	-0.900
Eggerthellales	Eggerthellaceae	Xiamenia	Xiamenia xianingshaonis	-0.892
Eubacteriales	Oscillospiraceae	Petroclostridium	Petroclostridium sp. X23	-0.890
Eubacteriales	Oscillospiraceae	Ruminococcus	Ruminococcus champanellensis	-0.888
Eubacteriales	Clostridiaceae	Hathewaya	Hathewaya histolytica	-0.885
Eubacteriales	Desulfitobacteriaceae	Desulfitobacterium	Desulfitobacterium hafniense	-0.883
Eggerthellales	Eggerthellaceae	Berryella	Berryella intestinalis	-0.882
Eubacteriales	Clostridiaceae	Alkaliphilus	Alkaliphilus sp. B6464	-0.881
Eubacteriales	Oscillospiraceae	Ruminiclostridium	Ruminiclostridium herbifermentans	-0.877
Eubacteriales	Heliobacteriaceae	Heliomicrobium	Heliomicrobium modesticaldum	-0.877
Eubacteriales	Peptococcaceae	Dehalobacterium	Dehalobacterium formicoaceticum	-0.864
Eubacteriales	Clostridiaceae	Clostridium	Clostridium kluyveri	-0.860
Eubacteriales	Oscillospiraceae		Oscillospiraceae bacterium MDTJ8	-0.857
Hyphomicrobiales	Nitrobacteraceae	Rhodopseudomonas	Rhodopseudomonas palustris	-0.853
Eubacteriales	Oscillospiraceae	Vescimonas	Vescimonas fastidiosa	-0.852
Eubacteriales	Clostridiaceae	Proteiniclasticum	Proteiniclasticum sp. QWL-01	-0.845
Eubacteriales	Eubacteriales Family XIII. Incertae Sedis	Aminipila	Aminipila terrae	-0.844
Eubacteriales	Clostridiaceae	Clostridium	Clostridium sp. JN-1	-0.837
Eubacteriales	Oscillospiraceae	Acutalibacter	Acutalibacter muris	-0.836
Bacillales	Paenibacillaceae	Cohnella	Cohnella candidum	-0.835
Bacillales	Paenibacillaceae	Paenibacillus	Paenibacillus sp. SYP-B4298	-0.825
Tissierellales	Peptoniphilaceae	Miniphocaeibacter	Miniphocaeibacter halophilus	-0.825
Bacillales	Staphylococcaceae	Staphylococcus	Staphylococcus epidermidis	-0.823
Eubacteriales	Clostridiaceae	Oceanirhabdus	Oceanirhabdus sp. W0125-5	-0.818
Peptostreptococcales	Peptostreptococcaceae	Tepidibacter	Tepidibacter hydrothermalis	-0.813
Bacillales	Paenibacillaceae	Thermobacillus	Thermobacillus composti	-0.810
Eubacteriales	Desulfallaceae	Desulfoscipio	Desulfoscipio gibsoniae	-0.810

Continued on next page

order	family	genus	species	weight
Eubacteriales	Oscillospiraceae	Caproicibacterium	Caproicibacterium sp. BJN0003	-0.806
Eubacteriales	Oscillospiraceae	Pusillibacter	Pusillibacter faecalis	-0.806
Eubacteriales	Oscillospiraceae	Mageeibacillus	Mageeibacillus indolicus	-0.804
Eubacteriales	Peptostreptococcaceae	Acetoanaerobium	Acetoanaerobium sticklandii	-0.803
Eubacteriales	Oscillospiraceae	Fastidiosipila	Fastidiosipila sanguinis	-0.803
Eubacteriales	Eubacteriaceae	Acetobacterium	Acetobacterium sp. KB-1	-0.803
Eubacteriales	Clostridiaceae	Caloramator	Caloramator sp. Dgby_cultured_2	-0.801
Eubacteriales	Oscillospiraceae	Vescimonas	Vescimonas coprocola	-0.800
Eubacteriales	Oscillospiraceae	Caproiciproducens	Caproiciproducens sp. NJN-50	-0.798
Coriobacteriales	Atopobiaceae	Olsenella	Olsenella uli	-0.797
Bacillales	Alicyclobacillaceae	Alicyclobacillus	Alicyclobacillus acidocaldarius	-0.791
Eubacteriales	Oscillospiraceae	Acetivibrio	Acetivibrio thermocellus	-0.789
Bacillales	Paenibacillaceae	Paenibacillus	Paenibacillus azoreducens	-0.787
Eubacteriales		Intestinimonas	Intestinimonas butyriciproducens	-0.782
Bacillales	Bacillaceae	Bacillus	Bacillus sp. FJAT-18017	-0.782
Tissierellales	Peptoniphilaceae	Parvimonas	Parvimonas micra	-0.781
Selenomonadales	Selenomonadaceae	Selenomonas	Selenomonas ruminantium	-0.778
Eubacteriales	Eubacteriaceae	Acetobacterium	Acetobacterium wieringae	-0.778
Burkholderiales	Alcaligenaceae	Achromobacter	Achromobacter xylosoxidans	-0.776
Eubacteriales	Clostridiaceae	Clostridium	Clostridium cochlearium	-0.776
Eubacteriales	Peptococcaceae	Acididesulfobacillus	Acididesulfobacillus acetoxydans	-0.775
Bacillales	Paenibacillaceae	Paenibacillus	Paenibacillus dendritiformis	-0.773
Bacillales	Bacillaceae	Gottfriedia	Gottfriedia acidiceleris	-0.765
Spirochaetales	Treponemataceae	Treponema	Treponema brennaborense	-0.765
Eubacteriales	Oscillospiraceae	Caproicibacterium	Caproicibacterium amylolyticum	-0.764
Spirochaetales	Sphaerochaetaceae	Sphaerochaeta	Sphaerochaeta pleomorpha	-0.764
Eubacteriales	Clostridiaceae	Clostridium	Clostridium novyi	-0.759
Tissierellales	Thermohalobacteraceae	Caloranaerobacter	Caloranaerobacter azorensis	-0.758
Eubacteriales	Clostridiaceae	Serpentinicella	Serpentinicella alkaliphila	-0.758
Eubacteriales	Clostridiaceae	Clostridium	Clostridium tyrobutyricum	-0.756
Bacillales	Paenibacillaceae	Paenibacillus	Paenibacillus sp. RUD330	-0.756
Eubacteriales	Oscillospiraceae	Monoglobus	Monoglobus pectinilyticus	-0.755
Eubacteriales	Lachnospiraceae	Coprococcus	Coprococcus eutactus	-0.754
		Ezakiella	Ezakiella coagulans	-0.754
Eubacteriales	Oscillospiraceae	Ruminococcus	Ruminococcus albus	-0.753
Eubacteriales	Clostridiaceae	Crassaminicella	Crassaminicella profunda	-0.752
Eubacteriales	Clostridiaceae	Clostridium	Clostridium estertheticum	-0.749
Koleobacterales	Koleobacteraceae	Koleobacter	Koleobacter methoxysyntrophicus	-0.749
Eubacteriales	Clostridiaceae	Crassaminicella	Crassaminicella sp. 143-21	-0.744
Eubacteriales	Oscillospiraceae	Subdoligranulum	Subdoligranulum variabile	-0.744
Eggerthellales	Eggerthellaceae	Berryella	Berryella wangjianweii	-0.744

Continued on next page

order	family	genus	species	weight
Eubacteriales	Clostridiaceae	Clostridium	Clostridium tetani	-0.743
Eubacteriales	Peptostreptococcaceae	Filifactor	Filifactor alocis	-0.742
Eubacteriales	Clostridiaceae	Caloramator	Caloramator sp. mosi_1	-0.742
Eubacteriales	Oscillospiraceae	Oscillibacter	Oscillibacter hominis	-0.742
Bacillales	Bacillaceae	Bacillus	Bacillus subtilis	-0.741
Eubacteriales	Peptostreptococcaceae	Tepidibacter	Tepidibacter sp. 8C15b	-0.739
Eubacteriales	Clostridiaceae	Clostridium	Clostridium aceticum	-0.737
Eubacteriales	Vallitaleaceae	Vallitalea	Vallitalea guaymasensis	-0.736
Eubacteriales	Clostridiaceae	Clostridium	Clostridium acetobutylicum	-0.733
Eubacteriales	Clostridiaceae	Clostridium	Clostridium gelidum	-0.733
Tissierellales	Tissierellaceae	Tissierella	Tissierella sp. Yu-01	-0.731
Erysipelotrichales	Erysipelotrichaceae	Tannockella	Tannockella kyphosi	-0.730
Eubacteriales	Oscillospiraceae	Ruminiclostridium	Ruminiclostridium papyrosolvens	-0.730
Tissierellales	Gottschalkiaceae	Gottschalkia	Gottschalkia acidurici	-0.730
Bacillales	Paenibacillaceae	Paenibacillus	Paenibacillus sp. 32O-W	-0.730
Eubacteriales	Clostridiaceae	Clostridium	Clostridium sp. BNL1100	-0.730
Eubacteriales	Clostridiaceae	Clostridium	Clostridium manihotivorum	-0.729
Eubacteriales	Clostridiaceae	Clostridium	Clostridium thermarum	-0.726
Selenomonadales	Selenomonadaceae	Selenomonas	Selenomonas timonae	-0.725
Eubacteriales	Christensenellaceae	Christensenella	Christensenella sp. Marseille-P3954	-0.724
Eubacteriales	Clostridiaceae	Clostridium	Clostridium sp. BJN0001	-0.723
Eubacteriales	Oscillospiraceae	Solibaculum	Solibaculum mannosilyticum	-0.723
Eubacteriales	Oscillospiraceae	Pseudoclostridium	Pseudoclostridium thermosuccinogenes	-0.723
Eubacteriales	Clostridiaceae	Clostridium	Clostridium argentinense	-0.723
Tissierellales	Acidilutibacteraceae	Acidilutibacter	Acidilutibacter cellobiosedens	-0.720
Eubacteriales	Clostridiaceae	Clostridium	Clostridium taeniosporum	-0.719
Synergistales	Dethiosulfovibrionaceae	Pyramidobacter	Pyramidobacter piscolens	-0.719
Thermoanaerobacterales	Thermoanaerobacteraceae	Gelria	Gelria sp. Kuro-4	-0.719
Thermoanaerobacterales	Thermoanaerobacterales Family III. Incertae Sedis	Thermoanaerobacterium	Thermoanaerobacterium thermosaccharolyticum	-0.717
Bacillales	Paenibacillaceae	Paenibacillus	Paenibacillus tianjinensis	-0.716
Bacillales	Paenibacillaceae	Paenibacillus	Paenibacillus albicereus	-0.716
Flavobacteriales	Blattabacteriaceae	Blattabacterium	Blattabacterium cuenoti	-0.714
Erysipelotrichales	Erysipelotrichaceae	Faecalibaculum	Faecalibaculum rodentium	-0.714
Eubacteriales	Eubacteriaceae	Alkalibacter	Alkalibacter rhizosphaerae	-0.713
Bacillales	Bacillaceae	Niallia	Niallia circulans	-0.712
Eubacteriales	Eubacteriales Family XIII. Incertae Sedis	Aminipila	Aminipila luticellarii	-0.712
Eubacteriales	Oscillospiraceae	Acetivibrio	Acetivibrio clariflavus	-0.711

Continued on next page

order	family	genus	species	weight
Bacillales	Alicyclobacillaceae	Tumebacillus	Tumebacillus avium	-0.711
Lactobacillales	Streptococcaceae	Streptococcus	Streptococcus dysgalactiae	-0.711
Bacillales	Bacillaceae	Peribacillus	Peribacillus asahii	-0.710
Bacillales	Bacillaceae	Salisediminibacterium	Salisediminibacterium selenitireducens	-0.709
Fusobacteriales	Fusobacteriaceae	Fusobacterium	Fusobacterium ulcerans	-0.709
Eubacteriales	Clostridiaceae	Clostridium	Clostridium sp. JN-9	-0.708
Selenomonadales	Selenomonadaceae	Selenomonas	Selenomonas sputigena	-0.708
Eubacteriales	Clostridiaceae	Clostridium	Clostridium pasteurianum	-0.705
Bacillales	Paenibacillaceae	Paenibacillus	Paenibacillus mucilaginosus	-0.705
		Sedimentibacter	Sedimentibacter sp. zth1	-0.705
Eubacteriales	Clostridiaceae	Clostridium	Clostridium cadaveris	-0.704
Eubacteriales	Oscillospiraceae	Caproicibacterium	Caproicibacterium lactatifermentans	-0.703
Bacillales	Bacillaceae	Heyndrickxia	Heyndrickxia oleronia	-0.699
Selenomonadales	Selenomonadaceae	Selenomonas	Selenomonas sp. oral taxon 126	-0.699
Spirochaetales	Treponemataceae	Treponema	Treponema denticola	-0.698
Enterobacterales	Erwiniaceae	Buchnera	Buchnera aphidicola	-0.698
Bacillales	Paenibacillaceae	Paenibacillus	Paenibacillus beijingensis	-0.698
Bacillales	Paenibacillaceae	Paenibacillus	Paenibacillus konkukensis	-0.695
Eubacteriales	Peptococcaceae	Candidatus Formimonas	Candidatus Formimonas warabiya	-0.694
Bacillales	Paenibacillaceae	Paenibacillus	Paenibacillus lycopersici	-0.694
Eubacteriales	Clostridiaceae	Clostridium	Clostridium sp. DL-VIII	-0.694
Eubacteriales	Cellulosilyticaceae	Cellulosilyticum	Cellulosilyticum sp. WCF-2	-0.691
Coriobacteriales	Atopobiaceae	Thermophilibacter	Thermophilibacter immobilis	-0.690
Bacillales	Paenibacillaceae	Paenibacillus	Paenibacillus guangzhouensis	-0.689
Coriobacteriales	Coriobacteriaceae	Coriobacterium	Coriobacterium glomerans	-0.689
Thermoanaerobacterales	Thermoanaerobacteraceae	Thermoanaerobacter	Thermoanaerobacter kivui	-0.688
Bacillales	Bacillaceae	Terribacillus	Terribacillus goriensis	-0.686
Bacillales	Paenibacillaceae	Paenibacillus	Paenibacillus donghaensis	-0.685
Eubacteriales	Eubacteriaceae	Eubacterium	Eubacterium sp. MSJ-33	-0.684
Eubacteriales	Eubacteriaceae	Eubacterium	Eubacterium limosum	-0.684
Thermoanaerobacterales	Thermoanaerobacterales Family III. Incertae Sedis	Thermoanaerobacterium	Thermoanaerobacterium sp. RBIITD	-0.681
Erysipelotrichales	Erysipelotrichaceae	Intestinibaculum	Intestinibaculum porci	-0.679
Eubacteriales	Lachnospiraceae	Tyzzerella	[Clostridium] colinum	-0.679
Eggerthellales	Eggerthellaceae	Eggerthella	Eggerthella sp. YY7918	-0.678
Bacillales	Planococcaceae	Ureibacillus	Ureibacillus thermosphaericus	-0.678
Eubacteriales	Peptococcaceae	Desulforamulus	Desulforamulus ruminis	-0.678

Continued on next page

order	family	genus	species	weight
Eubacteriales	Eubacteriales Family XIII. Incertae Sedis	Emergencia	Emergencia timonensis	-0.678
Eubacteriales	Vallitaleaceae	Petrocella	Petrocella atlantisensis	-0.678
Bacillales	Paenibacillaceae	Paenibacillus	Paenibacillus protaetiae	-0.676
Eggerthellales	Eggerthellaceae	Raoultibacter	Raoultibacter timonensis	-0.676
Tissierellales	Peptoniphilaceae	Anaerococcus	Anaerococcus prevotii	-0.674
Coriobacteriales	Coriobacteriaceae	Collinsella	Collinsella stercoris	-0.672
Thermoanaerobacterales	Thermoanaerobacteraceae	Moorella	Moorella sp. Hama-1	-0.672
Bacillales	Staphylococcaceae	Mammaliicoccus	Mammaliicoccus lentus	-0.668
Eubacteriales	Desulfitobacteriaceae	Syntrophobotulus	Syntrophobotulus glycolicus	-0.668
Eubacteriales	Clostridiaceae	Clostridium	Clostridium drakei	-0.668
Tissierellales	Peptoniphilaceae	Peptoniphilus	Peptoniphilus harei	-0.666
Eubacteriales	Clostridiaceae	Clostridium	Clostridium sp. MD294	-0.665
Bacillales	Paenibacillaceae	Paenibacillus	Paenibacillus stellifer	-0.665
Bacillales	Paenibacillaceae	Cohnella	Cohnella sp. LGH	-0.664
Eubacteriales	Oscillospiraceae	Thermoclostridium	Thermoclostridium stercorarium	-0.663
Eubacteriales	Clostridiaceae	Clostridium	Clostridium intestinale	-0.663
Lactobacillales	Enterococcaceae	Tetragenococcus	Tetragenococcus halophilus	-0.661
Bacillales	Paenibacillaceae	Paenibacillus	Paenibacillus algicola	-0.660
Bacillales	Paenibacillaceae	Paenibacillus	Paenibacillus elgii	-0.660
Tissierellales	Peptoniphilaceae	Finegoldia	Finegoldia magna	-0.659
Coriobacteriales	Atopobiaceae	Parafannyhessea	Parafannyhessea umbonata	-0.658
Erysipelotrichales	Erysipelotrichaceae	Faecalitalea	Faecalitalea cylindroides	-0.658
Lactobacillales	Carnobacteriaceae	Carnobacterium	Carnobacterium maltaromaticum	-0.657
Eubacteriales	Clostridiaceae	Clostridium	Clostridium fermenticellae	-0.657
Eubacteriales	Clostridiaceae	Clostridium	Clostridium bornimense	-0.657
Thermosediminibacterales	Tepidanaerobacteraceae	Biomaibacter	Biomaibacter acetigenes	-0.656
Thermoanaerobacterales	Thermoanaerobacterales Family IV. Incertae Sedis	Mahella	Mahella australiensis	-0.655
Bacillales	Paenibacillaceae	Paenibacillus	Paenibacillus sp. PHS-Z3	-0.654
Bacillales	Paenibacillaceae	Paenibacillus	Paenibacillus yonginensis	-0.653
Fusobacteriales	Fusobacteriaceae	Fusobacterium	Fusobacterium gonidiaformans	-0.650
Lactobacillales	Streptococcaceae	Streptococcus	Streptococcus suis	-0.649
Eubacteriales	Clostridiaceae	Clostridium	Clostridium carboxidivorans	-0.649
Eubacteriales	Clostridiaceae	Clostridium	Clostridium saccharobutylicum	-0.649
Bacillales	Bacillaceae	Bacillus	Bacillus glycinifermentans	-0.649
Eubacteriales	Clostridiaceae	Sarcina	Sarcina sp. JB2	-0.648
Eubacteriales	Peptostreptococcaceae	Peptostreptococcus	Peptostreptococcus sp. CBA3647	-0.647
Bacillales	Bacillaceae	Virgibacillus	Virgibacillus phasianinus	-0.646
Thermosediminibacterales	Tepidanaerobacteraceae	Tepidanaerobacter	Tepidanaerobacter acetatoxydans	-0.645

Continued on next page

order	family	genus	species	weight
Eubacteriales	Desulfitobacteriaceae	Desulfosporosinus	Desulfosporosinus acidiphilus	-0.644
Eubacteriales	Lachnospiraceae		Lachnospiraceae bacterium MP1D12	-0.644
Eubacteriales	Clostridiaceae	Clostridium	Clostridium sporogenes	-0.643
Eubacteriales	Symbiobacteriaceae	Symbiobacterium	Symbiobacterium thermophilum	-0.643
Bacillales	Paenibacillaceae	Paenibacillus	Paenibacillus amylolyticus	-0.642
Eggerthellales	Eggerthellaceae	Gordonibacter	Gordonibacter pamelaeae	-0.642
Coriobacteriales	Atopobiaceae	Lancefieldella	Lancefieldella sp. Marseille-Q7238	-0.641
Lactobacillales	Enterococcaceae	Enterococcus	Enterococcus hirae	-0.641
Bacillales	Listeriaceae	Listeria	Listeria innocua	-0.641
Bacillales	Bacillaceae	Peribacillus	Peribacillus simplex	-0.641
Lactobacillales	Streptococcaceae	Streptococcus	Streptococcus equi	-0.638
Eubacteriales	Clostridiaceae	Clostridium	Clostridium isatidis	-0.637
Eubacteriales	Oscillospiraceae	Anaerotruncus	Anaerotruncus colihominis	-0.634
Xanthomonadales	Xanthomonadaceae	Stenotrophomonas	Stenotrophomonas maltophilia	-0.634
Eubacteriales	Clostridiaceae	Clostridium	Clostridium cellulovorans	-0.633
Eubacteriales	Clostridiaceae	Alkaliphilus	Alkaliphilus metalliredigens	-0.633
Erysipelotrichales	Erysipelotrichaceae	Allobaculum	Allobaculum sp. Allo2	-0.631
Eubacteriales	Clostridiales Family XVI. Incertae Sedis	Carboxydocella	Carboxydocella thermautotrophica	-0.630
Bacillales	Paenibacillaceae	Brevibacillus	Brevibacillus sp. WF146	-0.628
Eubacteriales	Desulfitobacteriaceae	Desulfosporosinus	Desulfosporosinus orientis	-0.627
Bacillales	Paenibacillaceae	Paenibacillus	Paenibacillus sp. B01	-0.626
Eubacteriales	Lachnospiraceae	Qiania	Qiania dongpingensis	-0.625
Tissierellales	Peptoniphilaceae	Peptoniphilus	Peptoniphilus sp. SAHP1	-0.625
Thermoanaerobacterales	Thermoanaerobacterales Family III. Incertae Sedis	Caldicellulosiruptor	Caldicellulosiruptor diazotrophicus	-0.623
Bacillales	Bacillaceae	Aeribacillus	Aeribacillus pallidus	-0.621
Enterobacterales	Morganellaceae	Proteus	Proteus vulgaris	-0.620
Bacillales	Paenibacillaceae	Paenibacillus	Paenibacillus sp. FSL H7-0357	-0.620
Eubacteriales	Clostridiaceae	Clostridium	Clostridium botulinum	-0.618
Burkholderiales	Burkholderiaceae	Burkholderia	Burkholderia cenocepacia	-0.617
Eubacteriales	Vallitaleaceae	Vallitalea	Vallitalea pronyensis	-0.617
Xanthomonadales	Xanthomonadaceae	Lysobacter	Lysobacter enzymogenes	-0.616
Bacillales	Bacillaceae	Priestia	Priestia megaterium	-0.616
Bacillales	Paenibacillaceae	Paenibacillus	Paenibacillus sp. YPG26	-0.616
Eubacteriales	Lachnospiraceae	Anaerotignum	Anaerotignum propionicum	-0.615
Bacillales	Paenibacillaceae	Paenibacillus	Paenibacillus sp. FSL R7-0273	-0.613
Bacillales	Bacillaceae	Neobacillus	Neobacillus sp. SuZ13	-0.613
Bacillales	Paenibacillaceae	Paenibacillus	Paenibacillus cellulosilyticus	-0.613

Continued on next page

order	family	genus	species	weight
Hyphomicrobiales	Rhizobiaceae	Ensifer	Ensifer adhaerens	-0.612
Brachyspirales	Brachyspiraceae	Brachyspira	Brachyspira murdochii	-0.611
Hyphomicrobiales	Rhizobiaceae	Sinorhizobium	Sinorhizobium fredii	-0.610
Eubacteriales		Fenollaria	Fenollaria massiliensis	-0.609
Eubacteriales	Lachnospiraceae	Anaeropeptidivorans	Anaeropeptidivorans aminofermentans	-0.609
Kitasatosporales	Streptomycetaceae	Streptomyces	Streptomyces venezuelae	-0.608
Eubacteriales	Clostridiaceae	Clostridium	Clostridium saccharoperbutylacetonicum	-0.608
Selenomonadales	Sporomusaceae	Methylomusa	Methylomusa anaerophila	-0.606
Eubacteriales	Lachnospiraceae	Herbinix	Herbinix luporum	-0.605
Eubacteriales	Lachnospiraceae	Coprococcus	Coprococcus catus	-0.604
Eubacteriales	Clostridiaceae	Alkaliphilus	Alkaliphilus oremlandii	-0.604
Eubacteriales	Clostridiaceae	Geosporobacter	Geosporobacter ferrireducens	-0.604
Bacillales	Bacillaceae	Weizmannia	Weizmannia coagulans	-0.604
Burkholderiales	Comamonadaceae	Variovorax	Variovorax paradoxus	-0.602
Synergistales	Aminobacteriaceae	Fretibacterium	Fretibacterium fastidiosum	-0.600
Spirochaetales	Treponemataceae	Treponema	Treponema succinifaciens	-0.598
Eubacteriales	Peptostreptococcaceae	Peptoclostridium	Peptoclostridium acidaminophilum	-0.597
Bacillales	Bacillaceae	Fictibacillus	Fictibacillus enclensis	-0.597
Eubacteriales	Clostridiaceae	Clostridium	Clostridium gasigenes	-0.596
Coriobacteriales	Atopobiaceae	Olsenella	Olsenella timonensis	-0.596
Eubacteriales	Lachnospiraceae	Butyrivibrio	Butyrivibrio proteoclasticus	-0.595
Eubacteriales		Flintibacter	Flintibacter sp. KGMB00164	-0.594
Bacillales	Bacillaceae	Metabacillus	Metabacillus sp. cB07	-0.594
Lactobacillales	Streptococcaceae	Lactococcus	Lactococcus raffinolactis	-0.593
Eggerthellales	Eggerthellaceae	Arabiibacter	Arabiibacter massiliensis	-0.593
Eubacteriales	Lachnospiraceae	Lachnoclostridium	Lachnoclostridium phytofermentans	-0.593
Bacillales	Paenibacillaceae	Paenibacillus	Paenibacillus rhizovicinus	-0.590
Eubacteriales	Lachnospiraceae	Coprococcus	Coprococcus sp. ART55/1	-0.589
Bacillales	Bacillaceae	Neobacillus	Neobacillus mesonae	-0.588
Bacillales	Paenibacillaceae	Paenibacillus	Paenibacillus macerans	-0.587
Spirochaetales	Treponemataceae	Treponema	Treponema parvum	-0.587
Spirochaetales	Breznakiellaceae	Breznakiella	Breznakiella homolactica	-0.586
Moraxellales	Moraxellaceae	Acinetobacter	Acinetobacter baumannii	-0.586
Eggerthellales	Eggerthellaceae	Adlercreutzia	Adlercreutzia hattorii	-0.586
Eubacteriales	Oscillospiraceae	Ruminiclostridium	Ruminiclostridium cellulolyticum	-0.584
Bacillales	Paenibacillaceae	Brevibacillus	Brevibacillus brevis	-0.584
Coriobacteriales	Atopobiaceae	Parolsenella	Parolsenella massiliensis	-0.584
Eubacteriales	Desulfitobacteriaceae	Desulfosporosinus	Desulfosporosinus youngiae	-0.583

Continued on next page

order	family	genus	species	weight
Bacteroidales	Odoribacteraceae	Butyricimonas	Butyricimonas faecalis	-0.583
Eubacteriales	Lachnospiraceae	Butyrivibrio	Butyrivibrio fibrisolvens	-0.579
Myxococcales	Sandaracinaceae	Sandaracinus	Sandaracinus amylolyticus	-0.579
Bacillales	Paenibacillaceae	Paenibacillus	Paenibacillus sp. URB8-2	-0.578
Pseudomonadales	Pseudomonadaceae	Pseudomonas	Pseudomonas fluorescens	-0.577
Bacillales	Bacillaceae	Priestia	Priestia filamentosa	-0.577
Bacillales	Paenibacillaceae	Paenibacillus	Paenibacillus sp. YPD9-1	-0.577
Enterobacterales	Morganellaceae	Providencia	Providencia rettgeri	-0.576
Spirochaetales	Sphaerochaetaceae	Sphaerochaeta	Sphaerochaeta globosa	-0.576
Bacillales	Paenibacillaceae	Paenibacillus	Paenibacillus agaridevorans	-0.576
Selenomonadales	Selenomonadaceae	Selenomonas	Selenomonas sp. oral taxon 920	-0.574
Burkholderiales	Burkholderiaceae	Cupriavidus	Cupriavidus taiwanensis	-0.574
		Ndongobacter	Ndongobacter massiliensis	-0.573
Lactobacillales	Lactobacillaceae	Limosilactobacillus	Limosilactobacillus reuteri	-0.573
Bacillales	Bacillaceae	Bacillus	Bacillus velezensis	-0.571
Bryobacterales	Bryobacteraceae	Paludibaculum	Paludibaculum fermentans	-0.570
Enterobacterales	Yersiniaceae	Serratia	Serratia marcescens	-0.569
Bacillales	Paenibacillaceae	Paenibacillus	Paenibacillus woosongensis	-0.569
Lactobacillales	Lactobacillaceae	Liquorilactobacillus	Liquorilactobacillus hordei	-0.568
Actinomycetales	Actinomycetaceae	Fannyhessea	Fannyhessea vaginae	-0.566
Spirochaetales	Treponemataceae	Treponema	Treponema primitia	-0.566
Bacillales	Paenibacillaceae	Paenibacillus	Paenibacillus peoriae	-0.565
Selenomonadales	Selenomonadaceae	Selenomonas	Selenomonas sp. oral taxon 478	-0.565
Tissierellales	Peptoniphilaceae	Helcococcus	Helcococcus kunzii	-0.564
Bacillales	Paenibacillaceae	Paenibacillus	Paenibacillus sp. JDR-2	-0.564
Bacillales	Paenibacillaceae	Paenibacillus	Paenibacillus physcomitrellae	-0.563
Bacillales	Bacillaceae	Oceanobacillus	Oceanobacillus oncorhynchi	-0.563
Bacillales	Paenibacillaceae	Paenibacillus	Paenibacillus odorifer	-0.562
Bacillales	Bacillaceae	Sutcliffiella	Sutcliffiella horikoshii	-0.562
Bacillales	Bacillaceae	Neobacillus	Neobacillus sp. DY30	-0.562
Bifidobacteriales	Bifidobacteriaceae	Bifidobacterium	Bifidobacterium thermophilum	-0.561
Acholeplasmatales	Acholeplasmataceae	Haploplasma	Haploplasma axanthum	-0.561
Eubacteriales	Christensenellaceae	Christensenella	Christensenella minuta	-0.559
Myxococcales	Kofleriaceae	Haliangium	Haliangium ochraceum	-0.558
Bacillales	Bacillaceae	Mesobacillus	Mesobacillus sp. S13	-0.558
Acholeplasmatales	Acholeplasmataceae	Acholeplasma	Acholeplasma laidlawii	-0.557
Eubacteriales	Eubacteriales Family XIII. Incertae Sedis	Mogibacterium	Mogibacterium diversum	-0.556
Desulfovibrionales	Desulfovibrionaceae	Oceanidesulfovibrio	Oceanidesulfovibrio marinus	-0.555
Lactobacillales	Lactobacillaceae	Lactobacillus	Lactobacillus jensenii	-0.555
Eubacteriales	Lachnospiraceae	Coprococcus	Coprococcus comes	-0.555

Continued on next page

order	family	genus	species	weight
Lactobacillales	Streptococcaceae	Lactococcus	Lactococcus garvieae	-0.554
Bacillales	Bacillaceae	Niallia	Niallia sp. Man26	-0.554
Burkholderiales	Burkholderiaceae	Ralstonia	Ralstonia solanacearum	-0.554
Bacillales	Staphylococcaceae	Macrococcus	Macrococcus caseolyticus	-0.552
Spirochaetales	Breznakiellaceae	Leadbettera	Leadbettera azotonutricia	-0.552
Bacillales	Paenibacillaceae	Paenibacillus	Paenibacillus durus	-0.551
Aeromonadales	Succinivibrionaceae	Succinivibrio	Succinivibrio dextrinosolvens	-0.551
Bacillales	Bacillaceae	Psychrobacillus	Psychrobacillus sp. INOP01	-0.550
Eubacteriales	Clostridiaceae	Clostridium	Clostridium felsineum	-0.550
Fusobacteriales	Fusobacteriaceae	Fusobacterium	Fusobacterium nucleatum	-0.550
Eubacteriales	Oscillospiraceae	Ruthenibacterium	Ruthenibacterium lactatiformans	-0.550
Lactobacillales	Streptococcaceae	Streptococcus	Streptococcus ratti	-0.549
Eubacteriales	Clostridiaceae	Clostridium	Clostridium beijerinckii	-0.548
Burkholderiales	Burkholderiaceae	Cupriavidus	Cupriavidus pauculus	-0.547
Nostocales	Rivulariaceae	Rivularia	Rivularia sp. PCC 7116	-0.547
Bacillales	Bacillaceae	Bacillus	Bacillus methanolicus	-0.547
Eubacteriales	Clostridiaceae	Clostridium	Clostridium sp. SY8519	-0.546
Legionellales	Legionellaceae	Legionella	Legionella pneumophila	-0.546
Bacillales	Alicyclobacillaceae	Tumebacillus	Tumebacillus algifaecis	-0.545
Eubacteriales	Oscillospiraceae	Acetivibrio	Acetivibrio saccincola	-0.544
Erysipelotrichales	Erysipelotrichaceae	Allobaculum	Allobaculum mucilyticum	-0.543
Lactobacillales	Streptococcaceae	Lactococcus	Lactococcus lactis	-0.542
Bacillales	Paenibacillaceae	Paenibacillus	Paenibacillus sp. 19GGS1-52	-0.540
Bacillales	Bacillaceae	Neobacillus	Neobacillus novalis	-0.540
Bacillales	Paenibacillaceae	Paenibacillus	Paenibacillus bovis	-0.540
Eubacteriales	Clostridiaceae	Clostridium	Clostridium sp. 'deep sea'	-0.540
Aeromonadales	Aeromonadaceae	Aeromonas	Aeromonas hydrophila	-0.539
Bacillales	Bacillaceae	Metabacillus	Metabacillus sediminilitoris	-0.539
Mycobacteriales	Corynebacteriaceae	Corynebacterium	Corynebacterium jeikeium	-0.539
Coriobacteriales	Atopobiaceae	Olsenella	Olsenella sp. oral taxon 807	-0.539
Eubacteriales	Clostridiaceae	Clostridium	Clostridium sp. AWRP	-0.539
Eubacteriales	Clostridiaceae	Clostridium	Clostridium neonatale	-0.538
Pseudomonadales	Pseudomonadaceae	Pseudomonas	Pseudomonas brassicacearum	-0.538
Bacillales	Bacillaceae	Oceanobacillus	Oceanobacillus zhaokaii	-0.538
Bacillales	Bacillaceae	Cytobacillus	Cytobacillus firmus	-0.538
Bacillales	Paenibacillaceae	Paenibacillus	Paenibacillus sp. FSL R7-0331	-0.537
Bacillales	Paenibacillaceae	Paenibacillus	Paenibacillus urinalis	-0.537
Lactobacillales	Lactobacillaceae	Latilactobacillus	Latilactobacillus curvatus	-0.536
Lactobacillales	Lactobacillaceae	Lactobacillus	Lactobacillus iners	-0.534
Bacillales	Bacillaceae	Priestia	Priestia koreensis	-0.534

Continued on next page

order	family	genus	species	weight
Thermoanaerobacterales	Thermoanaerobacterales Family III. Incertae Sedis	Caldicellulosiruptor	Caldicellulosiruptor morganii	-0.532
Eubacteriales	Eubacteriaceae	Eubacterium	Eubacterium callanderi	-0.532
Lactobacillales	Streptococcaceae	Streptococcus	Streptococcus lutetiensis	-0.532
Eubacteriales	Clostridiaceae	Clostridium	Clostridium chauvoei	-0.531
Eubacteriales	Lachnospiraceae	Pseudobutyrivibrio	Pseudobutyrivibrio xylanivorans	-0.531
Bacillales	Alicyclobacillaceae	Alicyclobacillus	Alicyclobacillus fastidiosus	-0.530
Bacillales	Paenibacillaceae	Paenibacillus	Paenibacillus lentus	-0.528
Desulfuromonadales	Geobacteraceae	Trichlorobacter	Trichlorobacter lovleyi	-0.528
Bacillales	Bacillaceae	Neobacillus	Neobacillus sp. YX16	-0.526
Bacillales	Bacillaceae	Evansella	Evansella cellulosilytica	-0.526
Eubacteriales	Lachnospiraceae	Lachnoanaerobaculum	Lachnoanaerobaculum umeaense	-0.525
Lactobacillales	Streptococcaceae	Streptococcus	Streptococcus pyogenes	-0.525
Eubacteriales	Lachnospiraceae	Butyrivibrio	Butyrivibrio hungatei	-0.524
Bacillales	Bacillaceae	Anoxybacillus	Anoxybacillus sediminis	-0.524
Eubacteriales	Lachnospiraceae	Anaeromicropila	Anaeromicropila herbilytica	-0.523
Fusobacteriales	Leptotrichiaceae	Streptobacillus	Streptobacillus moniliformis	-0.522
Bacillales	Paenibacillaceae	Paenibacillus	Paenibacillus antarcticus	-0.520
Lactobacillales	Lactobacillaceae	Lactiplantibacillus	Lactiplantibacillus pentosus	-0.520
Eubacteriales	Eubacteriales Family XIII. Incertae Sedis	Mogibacterium	Mogibacterium pumilum	-0.520
Eubacteriales	Lachnospiraceae	Anaerocolumna	Anaerocolumna chitinilytica	-0.520
Lactobacillales	Streptococcaceae	Streptococcus	Streptococcus equinus	-0.520
Enterobacterales	Enterobacteriaceae	Cedecea	Cedecea neteri	-0.519
Bacillales	Bacillaceae	Priestia	Priestia flexa	-0.519
Spirochaetales	Spirochaetaceae	Sediminispirochaeta	Sediminispirochaeta smaragdinae	-0.518
Eubacteriales	Desulfitobacteriaceae	Dehalobacter	Dehalobacter restrictus	-0.518
Lactobacillales	Lactobacillaceae	Ligilactobacillus	Ligilactobacillus murinus	-0.518
Bacillales	Paenibacillaceae	Paenibacillus	Paenibacillus psychroresistens	-0.517
Burkholderiales	Comamonadaceae	Comamonas	Comamonas testosteroni	-0.517
Hyphomicrobiales	Rhizobiaceae	Rhizobium	Rhizobium leguminosarum	-0.517
Bacillales	Paenibacillaceae	Paenibacillus	Paenibacillus sp. sptzw28	-0.516
Bacillales	Paenibacillaceae	Paenibacillus	Paenibacillus chitinolyticus	-0.515
Eubacteriales	Oscillospiraceae	Faecalibacterium	Faecalibacterium sp. HTF-F	-0.514
Eubacteriales	Cellulosilyticaceae	Cellulosilyticum	Cellulosilyticum lentocellum	-0.512
Pseudomonadales	Pseudomonadaceae	Pseudomonas	Pseudomonas putida	-0.511
Bacillales		Gemella	Gemella haemolysans	-0.510
Lachnospirales	Lachnospiraceae	Anaerocolumna	Anaerocolumna sp. AGMB13025	-0.510
Marinilabiliales	Marinilabiliaceae	Alkaliflexus	Alkaliflexus sp. Ai-910	-0.510
Eubacteriales	Clostridiaceae	Caloramator	Caloramator sp. E03	-0.509

Continued on next page

order	family	genus	species	weight
Bifidobacteriales	Bifidobacteriaceae	Bifidobacterium	Bifidobacterium asteroides	-0.508
Eubacteriales	Clostridiaceae	Paraclostridium	Paraclostridium bifermentans	-0.507
Eubacteriales	Peptococcaceae	Desulforamulus	Desulforamulus reducens	-0.507
Xanthomonadales	Xanthomonadaceae	Stenotrophomonas	Stenotrophomonas rhizophila	-0.506
Bacillales	Bacillaceae	Bacillus	Bacillus amyloliquefaciens	-0.506
Bacillales	Bacillaceae	Cytobacillus	Cytobacillus oceanisediminis	-0.506
Eubacteriales	Clostridiaceae	Clostridium	Clostridium formicaceticum	-0.506
Eubacteriales	Peptococcaceae	Desulfofarcimen	Desulfofarcimen acetoxidans	-0.505
Bifidobacteriales	Bifidobacteriaceae	Bifidobacterium	Bifidobacterium choerinum	-0.505
Bacillales	Paenibacillaceae	Cohnella	Cohnella herbarum	-0.504
Bacillales	Alicyclobacillaceae	Alicyclobacillus	Alicyclobacillus cycloheptanicus	-0.503
Eubacteriales	Clostridiaceae	Clostridium	Clostridium scatologenes	-0.501
Selenomonadales	Sporomusaceae	Pelosinus	Pelosinus sp. UFO1	-0.501
Bacillales	Paenibacillaceae	Brevibacillus	Brevibacillus agri	-0.500
Fusobacteriales	Fusobacteriaceae	Ilyobacter	Ilyobacter polytropus	-0.500

3.7.5 Factor3 positive pathways with feature weight >=0.5

[1] 39

Var1	Freq
map01100 Metabolic pathways	19
map01210 2-Oxocarboxylic acid metabolism	4
map01230 Biosynthesis of amino acids	4
map00630 Glyoxylate and dicarboxylate metabolism	3
map01200 Carbon metabolism	3
map04922 Glucagon signaling pathway	3
map04976 Bile secretion	3
map05230 Central carbon metabolism in cancer	3
map00020 Citrate cycle (TCA cycle)	2
map00051 Fructose and mannose metabolism	2
map00130 Ubiquinone and other terpenoid-quinone biosynthesis	2
map00220 Arginine biosynthesis	2
map00250 Alanine, aspartate and glutamate metabolism	2
map00330 Arginine and proline metabolism	2
map00620 Pyruvate metabolism	2
map04066 HIF-1 signaling pathway	2
map04964 Proximal tubule bicarbonate reclamation	2
map04977 Vitamin digestion and absorption	2
map00010 Glycolysis / Gluconeogenesis	1
map00040 Pentose and glucuronate interconversions	1
map00053 Ascorbate and aldarate metabolism	1
map00230 Purine metabolism	1
map00260 Glycine, serine and threonine metabolism	1
map00270 Cysteine and methionine metabolism	1
map00290 Valine, leucine and isoleucine biosynthesis	1
map00310 Lysine degradation	1
map00340 Histidine metabolism	1
map00360 Phenylalanine metabolism	1
map00400 Phenylalanine, tyrosine and tryptophan biosynthesis	1
map00430 Taurine and hypotaurine metabolism	1
map00471 D-Glutamine and D-glutamate metabolism	1
map00640 Propanoate metabolism	1
map00650 Butanoate metabolism	1
map00740 Riboflavin metabolism	1
map00750 Vitamin B6 metabolism	1
map00785 Lipoic acid metabolism	1
map00790 Folate biosynthesis	1
map00900 Terpenoid backbone biosynthesis	1
map00920 Sulfur metabolism	1
map02010 ABC transporters	1

Var1	Freq
map04024 cAMP signaling pathway	1
map04727 GABAergic synapse	1
map04742 Taste transduction	1
map05200 Pathways in cancer	1
map05211 Renal cell carcinoma	1

Таблица 18: Table, Features with positive weight >= 0.5 (39)

Name	weight	Formula	Ion.Mode	KEGG.ID	Sub.Pathway	Pathway
2’-deoxymugineic acid;	0.704	C12 H20 N2 O7	NEG	C15485		
4-amino-4-deoxychorismate; adc;	0.693	C10 H11 N O5	NEG	C11355	Folate biosynthesis	map00790 Folate biosynthesis;
Ethychlozate;	0.673	C11 H11 Cl N2 O2	NEG	C18532		
4-oxoproline	0.660	C5 H7 N O3	NEG	C01877	Arginine and proline metabolism	map00330 Arginine and proline metabolism; map01100 Metabolic pathways;
Uric acid	0.654	C5 H4 N4 O3	NEG	C00366	Purine metabolism	map00230 Purine metabolism; map01100 Metabolic pathways; map04976 Bile secretion;
N-acetyl-l-2-amino-6-oxopimelate; l-2-acetamido-6-oxoheptanedioate; l-2-acetamido-6-oxopimelate;	0.652	C9 H13 N O6	NEG	C05539	Monobactam biosynthesis	map01100 Metabolic pathways; map01230 Biosynthesis of amino acids;

Continued on next page

Name	weight	Formula	Ion.Mode	KEGG.ID	Sub.Pathway	Pathway
L-(-)-malic acid	0.646	C4 H6 O5	NEG	C00149	Citrate cycle (TCA cycle)	map00020 Citrate cycle (TCA cycle); map00620 Pyruvate metabolism; map00630 Glyoxylate and dicarboxylate metabolism; map01100 Metabolic pathways; map01200 Carbon metabolism; map04742 Taste transduction; map04922 Glucagon signaling pathway; map04964 Proximal tubule bicarbonate reclamation; map05200 Pathways in cancer; map05211 Renal cell carcinoma; map05230 Central carbon metabolism in cancer;
Pyridoxamine 5-phosphate	0.645	C8 H13 N2 O5 P	NEG	C00647	Vitamin B6 metabolism	map00750 Vitamin B6 metabolism; map01100 Metabolic pathways; map04977 Vitamin digestion and absorption;
Hymexazol o-glucoside; hog;	0.644	C10 H15 N O7	NEG	C11102		
N-acetyl-l-cysteine	0.641	C5 H9 N O3 S	NEG	C06809		
Panfuran s; dihydroxymethylfuratrizine;	0.638	C11 H11 N5 O5	NEG	C19289		
7-methylthioheptyl glucosinolate;	0.626	C15 H29 N O9 S3	NEG	C17252	Glucosinolate biosynthesis	map01210 2-Oxocarboxylic acid metabolism;
S-(2-methylbutanoyl)-dihydrolipoamide;	0.616	C13 H25 N O2 S2	NEG	C05118		
S-succinyldihydrolipoamide;	0.612	C12 H21 N O4 S2	NEG	C01169		
3-hydroxy-3-methylglutaric acid	0.607	C6 H10 O5	NEG	C03761		

Continued on next page

Name	weight	Formula	Ion.Mode	KEGG.ID	Sub.Pathway	Pathway
Dl-homoserine	0.601	C4 H9 N O3	NEG	C00263	Glycine, serine and threonine metabolism	map00260 Glycine, serine and threonine metabolism; map00270 Cysteine and methionine metabolism; map00920 Sulfur metabolism; map01100 Metabolic pathways; map01230 Biosynthesis of amino acids;
Ranunculin;	0.601	C11 H16 O8	NEG	C08512		
(s)-acpa; (s)-2-amino-3-(3-carboxy-5-methyl-4-isoxazolyl)porionic acid;	0.595	C8 H10 N2 O5	NEG	C13673		
4-o-(beta-l-arabinofuranosyl)-(2s,4s)-4-hydroxyproline;	0.593	C10 H17 N O7	NEG	C20706		
N-(2,3-dihydroxybenzoyl)-l-serine; 2,3-dihydroxy-n-benzoyl-l-serine; 2,3-dihydroxybenzoylserine;	0.590	C10 H11 N O6	NEG	C04204		

Continued on next page

Name	weight	Formula	Ion.Mode	KEGG.ID	Sub.Pathway	Pathway
2-oxoglutaric acid	0.588	C5 H6 O5	NEG	C00026	Citrate cycle (TCA cycle)	map00020 Citrate cycle (TCA cycle); map00040 Pentose and glucuronate interconversions; map00053 Ascorbate and aldarate metabolism; map00220 Arginine biosynthesis; map00250 Alanine, aspartate and glutamate metabolism; map00310 Lysine degradation; map00340 Histidine metabolism; map00430 Taurine and hypotaurine metabolism; map00471 D-Glutamine and D-glutamate metabolism; map00630 Glyoxylate and dicarboxylate metabolism; map00650 Butanoate metabolism; map01100 Metabolic pathways; map01200 Carbon metabolism; map01210 2-Oxocarboxylic acid metabolism; map01230 Biosynthesis of amino acids; map04066 HIF-1 signaling pathway; map04727 GABAergic synapse; map04922 Glucagon signaling pathway; map04964 Proximal tubule bicarbonate reclamation; map04976 Bile secretion; map05230 Central carbon metabolism in cancer;

Continued on next page

Name	weight	Formula	Ion.Mode	KEGG.ID	Sub.Pathway	Pathway
Clotrimazole;	0.586	C22 H17 Cl N2	NEG	C06922		
Tetracycline	0.582	C22 H24 N2 O8	NEG	C06570	Tetracycline biosynthesis	map01100 Metabolic pathways; map04976 Bile secretion;
Gamma-l-glutamyl-d-alanine; l-gamma-glutamyl-d-alanine;	0.564	C8 H14 N2 O5	NEG	C03738		
N-acetyl-l-glutamine	0.557	C7 H12 N2 O4	NEG	D07063		
2,3-diaminopropionic acid	0.553	C3 H8 N2 O2	NEG	C06393		
N-acetylaspartic acid	0.551	C6 H9 N O5	NEG	C01042	Alanine, aspartate and glutamate metabolism	map00250 Alanine, aspartate and glutamate metabolism; map01100 Metabolic pathways;
Gibberellin a4	0.542	C19 H24 O5	NEG	C11864	Diterpenoid biosynthesis	
N-succinyl-l-glutamate; (2s)-2-(3-carboxypropanoylamino)pentanedioic acid;	0.542	C9 H13 N O7	NEG	C05931	Arginine and proline metabolism	map00330 Arginine and proline metabolism; map01100 Metabolic pathways;
D-fructose 1-phosphate	0.537	C6 H13 O9 P	NEG	C01094	Fructose and mannose metabolism	map00051 Fructose and mannose metabolism; map00400 Phenylalanine, tyrosine and tryptophan biosynthesis; map01100 Metabolic pathways;
Trans-cinnamic acid	0.532	C9 H8 O2	NEG	C00423	Ubiquinone and other terpenoid-quinone biosynthesis	map00130 Ubiquinone and other terpenoid-quinone biosynthesis; map00360 Phenylalanine metabolism; map01100 Metabolic pathways;
N-acetyl-dl-glutamic acid	0.531	C7 H11 N O5	NEG	C00624	Arginine biosynthesis	map00220 Arginine biosynthesis; map01100 Metabolic pathways; map01210 2-Oxocarboxylic acid metabolism; map01230 Biosynthesis of amino acids;

Continued on next page

Name	weight	Formula	Ion.Mode	KEGG.ID	Sub.Pathway	Pathway
Methitural;	0.529	C12 H20 N2 O2 S2	NEG	C07558		
L-(+)-lactic acid	0.526	C3 H6 O3	NEG	C00186	Glycolysis / Gluconeogenesis	map00010 Glycolysis / Gluconeogenesis; map00051 Fructose and mannose metabolism; map00620 Pyruvate metabolism; map00640 Propanoate metabolism; map01100 Metabolic pathways; map04024 cAMP signaling pathway; map04066 HIF-1 signaling pathway; map04922 Glucagon signaling pathway; map05230 Central carbon metabolism in cancer;
Glycolic acid	0.525	C2 H4 O3	NEG	C00160	Chlorocyclohexane and chlorobenzene degradation	map00630 Glyoxylate and dicarboxylate metabolism; map01100 Metabolic pathways; map01200 Carbon metabolism;
Geranyl pyrophosphate	0.511	C10 H20 O7 P2	NEG	C00341	Ubiquinone and other terpenoid-quinone biosynthesis	map00130 Ubiquinone and other terpenoid-quinone biosynthesis; map00900 Terpenoid backbone biosynthesis; map01100 Metabolic pathways;
Lipoic acid	0.507	C8 H14 O2 S2	NEG	C16241	Lipoic acid metabolism	map00785 Lipoic acid metabolism; map01100 Metabolic pathways;

Continued on next page

Name	weight	Formula	Ion.Mode	KEGG.ID	Sub.Pathway	Pathway
2-methylmaleate	0.506	C5 H6 O4	NEG	C02226	Valine, leucine and isoleucine biosynthesis	map00290 Valine, leucine and isoleucine biosynthesis; map01100 Metabolic pathways; map01210 2-Oxocarboxylic acid metabolism;
Riboflavin	0.504	C17 H20 N4 O6	NEG	C00255	Riboflavin metabolism	map00740 Riboflavin metabolism; map01100 Metabolic pathways; map02010 ABC transporters; map04977 Vitamin digestion and absorption;

3.7.6 Factor3 negative pathways with feature weight <= -0.5

4 Appendix

4.1 Functions

4.2 Setup R

4.3 Versions

4.3.1 Document version

4.3.2 Session Info

Platform

- version: R version 4.3.0 (2023-04-21)
- os: macOS Monterey 12.5.1
- system: aarch64, darwin20
- ui: X11
- language: (EN)
- collate: en_US.UTF-8
- ctype: en_US.UTF-8
- tz: Asia/Tokyo
- date: 2024-03-04
- pandoc: 2.19.2 @ /Applications/RStudio.app/Contents/Resources/app/quarto/bin/tools/ (via rmarkdown)

Packages

	ondiskversion	loadedversion	date	source
abind	1.4.5	1.4-5	2016-07-21	CRAN (R 4.3.0)
basilisk	1.14.3	1.14.3	2024-01-30	Bioconductor 3.18 (R 4.3.2)
basilisk.utils	1.14.1	1.14.1	2023-11-21	Bioconductor 3.18 (R 4.3.2)
BiocGenerics	0.48.1	0.48.1	2023-11-02	Bioconductor
bit	4.0.5	4.0.5	2022-11-15	CRAN (R 4.3.0)
bit64	4.0.5	4.0.5	2020-08-30	CRAN (R 4.3.0)
blob	1.2.4	1.2.4	2023-03-17	CRAN (R 4.3.0)
cachem	1.0.8	1.0.8	2023-05-01	CRAN (R 4.3.0)
cli	3.6.2	3.6.2	2023-12-11	CRAN (R 4.3.1)
codetools	0.2.19	0.2-19	2023-02-01	CRAN (R 4.3.0)
colorspace	2.1.0	2.1-0	2023-01-23	CRAN (R 4.3.0)
corrplot	0.92	0.92	2021-11-18	CRAN (R 4.3.0)
cowplot	1.1.3	1.1.3	2024-01-22	CRAN (R 4.3.0)
crayon	1.5.2	1.5.2	2022-09-29	CRAN (R 4.3.0)
curl	5.2.0	5.2.0	2023-12-08	CRAN (R 4.3.1)
data.table	1.15.0	1.15.0	2024-01-30	CRAN (R 4.3.1)
DBI	1.2.1	1.2.1	2024-01-12	CRAN (R 4.3.0)
dbplyr	2.4.0	2.4.0	2023-10-26	CRAN (R 4.3.1)
DelayedArray	0.28.0	0.28.0	2023-10-24	Bioconductor
devtools	2.4.5	2.4.5	2022-10-11	CRAN (R 4.3.0)
digest	0.6.34	0.6.34	2024-01-11	CRAN (R 4.3.1)
dir.expiry	1.10.0	1.10.0	2023-10-26	Bioconductor
dplyr	1.1.4	1.1.4	2023-11-17	CRAN (R 4.3.1)
ellipsis	0.3.2	0.3.2	2021-04-29	CRAN (R 4.3.0)
evaluate	0.23	0.23	2023-11-01	CRAN (R 4.3.1)

	ondiskversion	loadedversion	date	source
fansi	1.0.6	1.0.6	2023-12-08	CRAN (R 4.3.1)
farver	2.1.1	2.1.1	2022-07-06	CRAN (R 4.3.0)
fastmap	1.1.1	1.1.1	2023-02-24	CRAN (R 4.3.0)
filelock	1.0.3	1.0.3	2023-12-11	CRAN (R 4.3.1)
forcats	1.0.0	1.0.0	2023-01-29	CRAN (R 4.3.0)
fs	1.6.3	1.6.3	2023-07-20	CRAN (R 4.3.0)
generics	0.1.3	0.1.3	2022-07-05	CRAN (R 4.3.0)
GGally	2.2.0	2.2.0	2023-11-22	CRAN (R 4.3.1)
ggplot2	3.4.4	3.4.4	2023-10-12	CRAN (R 4.3.1)
ggrepel	0.9.5	0.9.5	2024-01-10	CRAN (R 4.3.1)
ggstats	0.5.1	0.5.1	2023-11-21	CRAN (R 4.3.1)
glue	1.7.0	1.7.0	2024-01-09	CRAN (R 4.3.1)
gtable	0.3.4	0.3.4	2023-08-21	CRAN (R 4.3.0)
HDF5Array	1.30.0	1.30.0	2023-11-06	Bioconductor
highr	0.10	0.10	2022-12-22	CRAN (R 4.3.0)
hoardr	0.5.4	0.5.4	2024-01-23	CRAN (R 4.3.1)
htmltools	0.5.7	0.5.7	2023-11-03	CRAN (R 4.3.1)
htmlwidgets	1.6.4	1.6.4	2023-12-06	CRAN (R 4.3.1)
httpuv	1.6.14	1.6.14	2024-01-26	CRAN (R 4.3.1)
IRanges	2.36.0	2.36.0	2023-10-26	Bioconductor
irlba	2.3.5.1	2.3.5.1	2022-10-03	CRAN (R 4.3.0)
jsonlite	1.8.8	1.8.8	2023-12-04	CRAN (R 4.3.1)
knitr	1.45	1.45	2023-10-30	CRAN (R 4.3.1)
labeling	0.4.3	0.4.3	2023-08-29	CRAN (R 4.3.0)
later	1.3.2	1.3.2	2023-12-06	CRAN (R 4.3.1)
lattice	0.22.5	0.22-5	2023-10-24	CRAN (R 4.3.1)
lifecycle	1.0.4	1.0.4	2023-11-07	CRAN (R 4.3.1)
lubridate	1.9.3	1.9.3	2023-09-27	CRAN (R 4.3.1)
magrittr	2.0.3	2.0.3	2022-03-30	CRAN (R 4.3.0)
Matrix	1.6.5	1.6-5	2024-01-11	CRAN (R 4.3.0)
MatrixGenerics	1.14.0	1.14.0	2023-10-26	Bioconductor
matrixStats	1.2.0	1.2.0	2023-12-11	CRAN (R 4.3.1)
memoise	2.0.1	2.0.1	2021-11-26	CRAN (R 4.3.0)
mime	0.12	0.12	2021-09-28	CRAN (R 4.3.0)
miniUI	0.1.1.1	0.1.1.1	2018-05-18	CRAN (R 4.3.0)
MOFA2	1.12.1	1.12.1	2024-01-13	Bioconductor 3.18 (R 4.3.2)
munsell	0.5.0	0.5.0	2018-06-12	CRAN (R 4.3.0)
openxlsx	4.2.5.2	4.2.5.2	2023-02-06	CRAN (R 4.3.0)
pander	0.6.5	0.6.5	2022-03-18	CRAN (R 4.3.0)
pheatmap	1.0.12	1.0.12	2019-01-04	CRAN (R 4.3.0)
pillar	1.9.0	1.9.0	2023-03-22	CRAN (R 4.3.0)
pkgbuild	1.4.3	1.4.3	2023-12-10	CRAN (R 4.3.1)
pkgconfig	2.0.3	2.0.3	2019-09-22	CRAN (R 4.3.0)
pkgload	1.3.4	1.3.4	2024-01-16	CRAN (R 4.3.1)
plyr	1.8.9	1.8.9	2023-10-02	CRAN (R 4.3.1)
png	0.1.8	0.1-8	2022-11-29	CRAN (R 4.3.0)
profvis	0.3.8	0.3.8	2023-05-02	CRAN (R 4.3.0)
promises	1.2.1	1.2.1	2023-08-10	CRAN (R 4.3.0)
purrr	1.0.2	1.0.2	2023-08-10	CRAN (R 4.3.0)
R6	2.5.1	2.5.1	2021-08-19	CRAN (R 4.3.0)
rappdirs	0.3.3	0.3.3	2021-01-31	CRAN (R 4.3.0)
RColorBrewer	1.1.3	1.1-3	2022-04-03	CRAN (R 4.3.0)

	ondiskversion	loadedversion	date	source
Rcpp	1.0.12	1.0.12	2024-01-09	CRAN (R 4.3.1)
RcppAnnoy	0.0.22	0.0.22	2024-01-23	CRAN (R 4.3.1)
remotes	2.4.2.1	2.4.2.1	2023-07-18	CRAN (R 4.3.0)
reshape2	1.4.4	1.4.4	2020-04-09	CRAN (R 4.3.0)
reticulate	1.35.0	1.35.0	2024-01-31	CRAN (R 4.3.1)
rhdf5	2.46.1	2.46.1	2023-12-02	Bioconductor 3.18 (R 4.3.2)
rhdf5filters	1.14.1	1.14.1	2023-12-16	Bioconductor 3.18 (R 4.3.2)
Rhdf5lib	1.24.1	1.24.1	2023-12-12	Bioconductor 3.18 (R 4.3.2)
rlang	1.1.3	1.1.3	2024-01-10	CRAN (R 4.3.1)
rmarkdown	2.25	2.25	2023-09-18	CRAN (R 4.3.1)
RSQLite	2.3.5	2.3.5	2024-01-21	CRAN (R 4.3.1)
rstudioapi	0.15.0	0.15.0	2023-07-07	CRAN (R 4.3.0)
Rtsne	0.17	0.17	2023-12-07	CRAN (R 4.3.1)
S4Arrays	1.2.0	1.2.0	2023-10-26	Bioconductor
S4Vectors	0.40.2	0.40.2	2023-11-25	Bioconductor 3.18 (R 4.3.2)
scales	1.3.0	1.3.0	2023-11-28	CRAN (R 4.3.1)
sessioninfo	1.2.2	1.2.2	2021-12-06	CRAN (R 4.3.0)
shiny	1.8.0	1.8.0	2023-11-17	CRAN (R 4.3.1)
SparseArray	1.2.3	1.2.3	2023-12-26	Bioconductor 3.18 (R 4.3.2)
stringi	1.8.3	1.8.3	2023-12-11	CRAN (R 4.3.1)
stringr	1.5.1	1.5.1	2023-11-14	CRAN (R 4.3.1)
taxizedb	0.3.1	0.3.1	2023-04-03	CRAN (R 4.3.0)
tibble	3.2.1	3.2.1	2023-03-20	CRAN (R 4.3.0)
tidyr	1.3.1	1.3.1	2024-01-24	CRAN (R 4.3.1)
tidyselect	1.2.0	1.2.0	2022-10-10	CRAN (R 4.3.0)
timechange	0.3.0	0.3.0	2024-01-18	CRAN (R 4.3.1)
urlchecker	1.0.1	1.0.1	2021-11-30	CRAN (R 4.3.0)
usethis	2.2.2	2.2.2	2023-07-06	CRAN (R 4.3.0)
utf8	1.2.4	1.2.4	2023-10-22	CRAN (R 4.3.1)
uwot	0.1.16	0.1.16	2023-06-29	CRAN (R 4.3.0)
vctrs	0.6.5	0.6.5	2023-12-01	CRAN (R 4.3.1)
withr	3.0.0	3.0.0	2024-01-16	CRAN (R 4.3.1)
xfun	0.42	0.42	2024-02-08	CRAN (R 4.3.1)
xtable	1.8.4	1.8-4	2019-04-21	CRAN (R 4.3.0)
XVector	0.42.0	0.42.0	2023-10-26	Bioconductor
yaml	2.3.8	2.3.8	2023-12-11	CRAN (R 4.3.1)
zip	2.3.1	2.3.1	2024-01-27	CRAN (R 4.3.1)
zlibbioc	1.48.0	1.48.0	2023-10-26	Bioconductor
