# Supplementary material for: Metagenome and metabolome study on inhaled corticosteroids in asthma patients with side effects
Source: J Integr Bioinform. 2025 Jun 24;22(3):20240062. doi: 10.1515/jib-2024-0062 (PMC13066347; doi:10.1515/jib-2024-0062)
Supplement: Supplementary file 3 — Supplementary Material Details [file j_jib-2024-0062_suppl_003.pdf]

Kyrgyz asthma metagenomic analysis

A. Sorokin

31.10.2023

1 Read data

```
## [1] TRUE
```

```
## [1] TRUE
```

```
## class: TreeSummarizedExperiment
## dim: 8902 24
## metadata(0):
## assays(1): counts
## rownames(8902): Prevotella copri 165179 Prevotella intermedia 28131 ...
##   Citrus endogenous pararetrovirus 1435008 Alphabaculovirus
##   aucalifornicae 3047383
## rowData names(7): kingdom phylum ... genus species
## colnames(24): KS0001 KS0002 ... KS0023 KS0024
## colData names(48): name ID.participant ... GenderDiag GenderSideDiag
## reducedDimNames(0):
## mainExpName: NULL
## altExpNames(0):
## rowLinks: NULL
## rowTree: NULL
## colLinks: NULL
## colTree: NULL
```

```
## class: TreeSummarizedExperiment
## dim: 8902 24
## metadata(0):
## assays(3): counts relabundance clr
## rownames(8902): species:Prevotella copri species:Prevotella intermedia
##   ... species:Citrus endogenous pararetrovirus species:Alphabaculovirus
##   aucalifornicae
## rowData names(7): kingdom phylum ... genus species
## colnames(24): KS0001 KS0002 ... KS0023 KS0024
## colData names(48): name ID.participant ... GenderDiag GenderSideDiag
## reducedDimNames(0):
## mainExpName: NULL
## altExpNames(0):
## rowLinks: a LinkDataFrame (8902 rows)
## rowTree: 1 phylo tree(s) (8898 leaves)
## colLinks: NULL
## colTree: NULL
```

2 Abundance plot

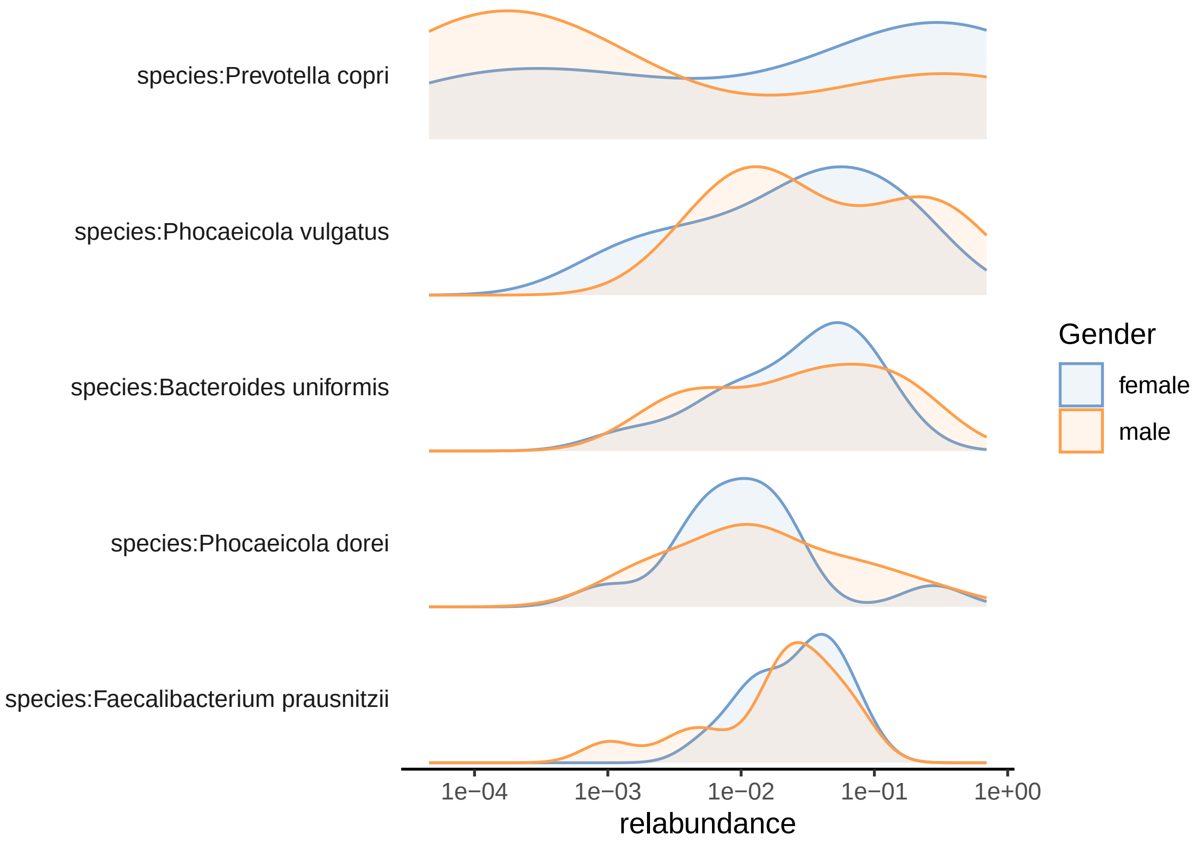

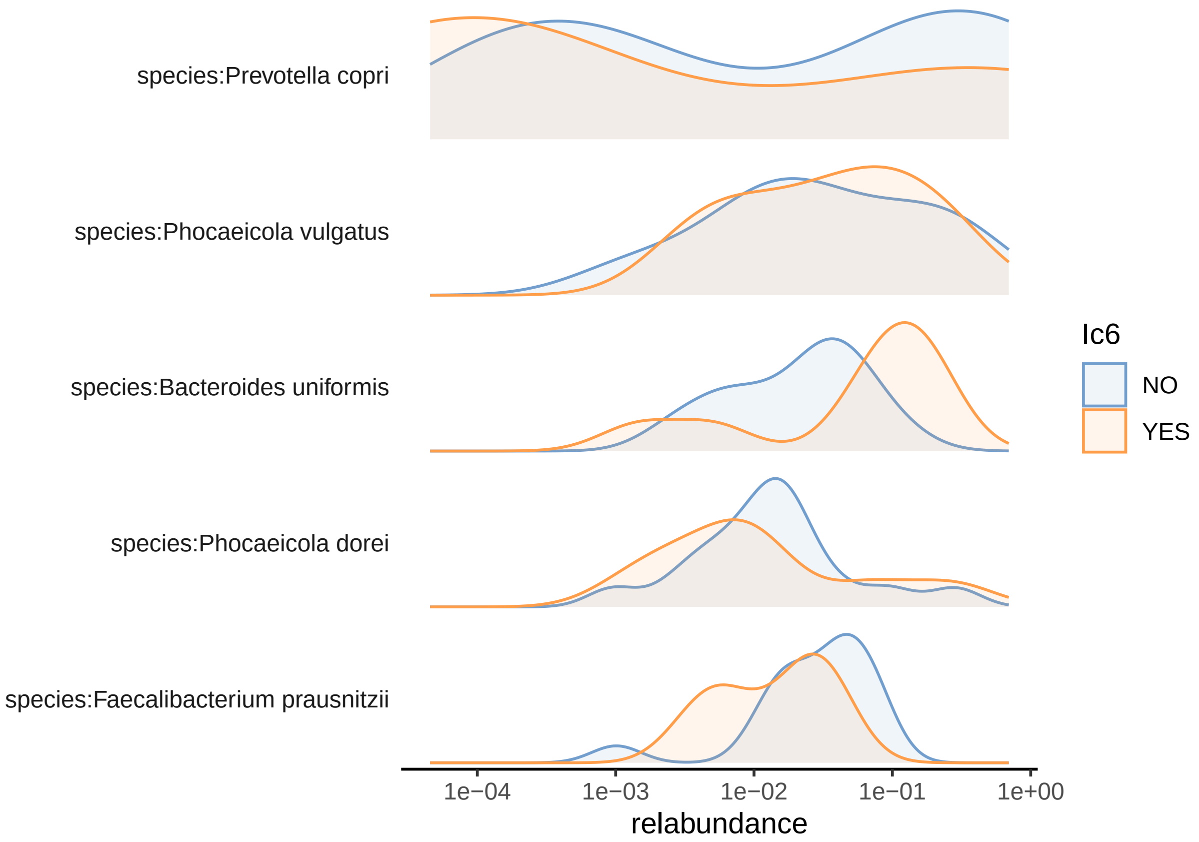

3 Prevalence

3.1 Species

```
## species:Faecalibacterium prausnitzii          species:Phocaeicola vulgatus
##                             0.8333333                             0.7500000
##            species:Bacteroides ovatus          species:Bacteroides uniformis
##                             0.7083333                             0.7083333
##          species:Bacteroides fragilis    species:Parabacteroides distasonis
##                             0.6666667                             0.6250000
## species:Bacteroides thetaiotaomicron       species:Bacteroides xylanisolvens
##                             0.6250000                             0.5833333
##             species:Phocaeicola dorei              species:Prevotella copri
##                             0.5416667                             0.4583333
##              species:Blautia wexlerae        species:Roseburia intestinalis
##                             0.4166667                             0.3750000
##        species:Parabacteroides merdae          species:Alistipes onderdonkii
##                             0.3750000                             0.3750000
##                 species:Simiaoa sunii              species:Alistipes shahii
##                             0.3333333                             0.3333333
##      species:Odoribacter splanchnicus              species:Dorea longicatena
##                             0.3333333                             0.2916667
##            species:Bacteroides caccae          species:Bacteroides stercoris
##                             0.2916667                             0.2916667
```

3.2 Genus

```
##        Bacteroides  Faecalibacterium       Phocaeicola   Parabacteroides
##          1.0000000         0.9166667         0.9166667         0.8750000
##          Alistipes         Roseburia           Blautia        Prevotella
##          0.7500000         0.6666667         0.6250000         0.4583333
##    Bifidobacterium       Coprococcus           Simiaoa       Odoribacter
##          0.3333333         0.3333333         0.3333333         0.3333333
##              Dorea Mediterraneibacter      Ruminococcus   Duodenibacillus
##          0.2916667         0.2916667         0.2500000         0.2083333
##        Escherichia             Wujia     Butyricimonas              Homo
##          0.2083333         0.2083333         0.2083333         0.1666667
```

3.3 Family

```
##     Lachnospiraceae      Bacteroidaceae    Oscillospiraceae      Tannerellaceae
##          1.00000000          1.00000000          0.95833333          0.87500000
##      Rikenellaceae    Odoribacteraceae      Prevotellaceae      Sutterellaceae
##          0.75000000          0.54166667          0.54166667          0.37500000
## Bifidobacteriaceae  Enterobacteriaceae           Hominidae    Selenomonadaceae
##          0.33333333          0.29166667          0.16666667          0.16666667
##    Akkermansiaceae     Veillonellaceae      Intestiviridae        Suoliviridae
##          0.12500000          0.12500000          0.08333333          0.08333333
##     Clostridiaceae        Steigviridae Methanobacteriaceae      Comamonadaceae
##          0.08333333          0.04166667          0.04166667          0.04166667
```

3.4 Order

```
##       Eubacteriales      Bacteroidales    Burkholderiales  Bifidobacteriales
##          1.00000000         1.00000000         0.41666667         0.33333333
##    Enterobacterales        Crassvirales     Lactobacillales           Primates
##          0.29166667         0.20833333         0.20833333         0.16666667
##    Selenomonadales Verrucomicrobiales Erysipelotrichales      Veillonellales
##          0.16666667         0.12500000         0.12500000         0.12500000
## Methanobacteriales     Pseudomonadales   Coriobacteriales          Bacillales
##          0.04166667         0.04166667         0.04166667         0.04166667
##  Acidaminococcales   Flavobacteriales        Ortervirales        Piccovirales
##          0.04166667         0.04166667         0.00000000         0.00000000
```

3.5 Class

```
##          Clostridia         Bacteroidia Gammaproteobacteria       Actinomycetes
##          1.00000000          1.00000000          0.58333333          0.45833333
##             Bacilli  Betaproteobacteria       Negativicutes      Caudoviricetes
##          0.45833333          0.41666667          0.41666667          0.25000000
##            Mammalia   Verrucomicrobiae   Erysipelotrichia       Coriobacteriia
##          0.16666667          0.12500000          0.12500000          0.08333333
##    Methanobacteria      Flavobacteriia     Revtraviricetes     Quintoviricetes
##          0.04166667          0.04166667          0.00000000          0.00000000
##      Faserviricetes        Methanopyri      Flasuviricetes   Conexivisphaeria
##          0.00000000          0.00000000          0.00000000          0.00000000
```

3.6 Phylum

```
##                 Bacillota                Bacteroidota
```

```
##                   1.00000000                1.00000000
##               Pseudomonadota             Actinomycetota
##                   0.91666667                0.70833333
##                 Uroviricota                   Chordata
##                   0.25000000                0.16666667
##           Verrucomicrobiota              Euryarchaeota
##                   0.12500000                0.04166667
##              Artverviricota               Cossaviricota
##                   0.00000000                0.00000000
##              Hofneiviricota             Kitrinoviricota
##                   0.00000000                0.00000000
##                 Pisuviricota          Preplasmiviricota
##                   0.00000000                0.00000000
## Candidatus Nanohaloarchaeota      Coprothermobacterota
##                   0.00000000                0.00000000
##               Taleaviricota              Nanoarchaeota
##                   0.00000000                0.00000000
##             Cressdnaviricota                Caldiserica
##                   0.00000000                0.00000000
```

4 Diversity

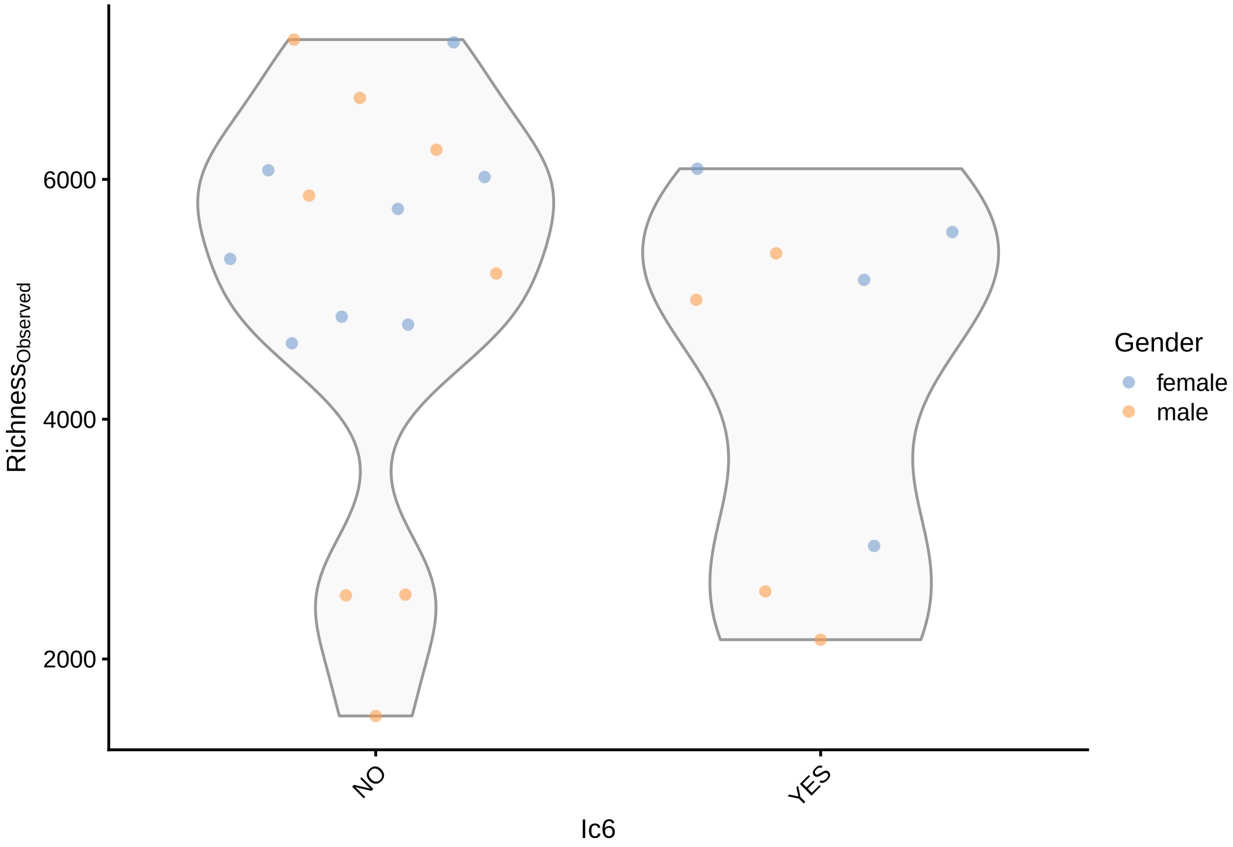

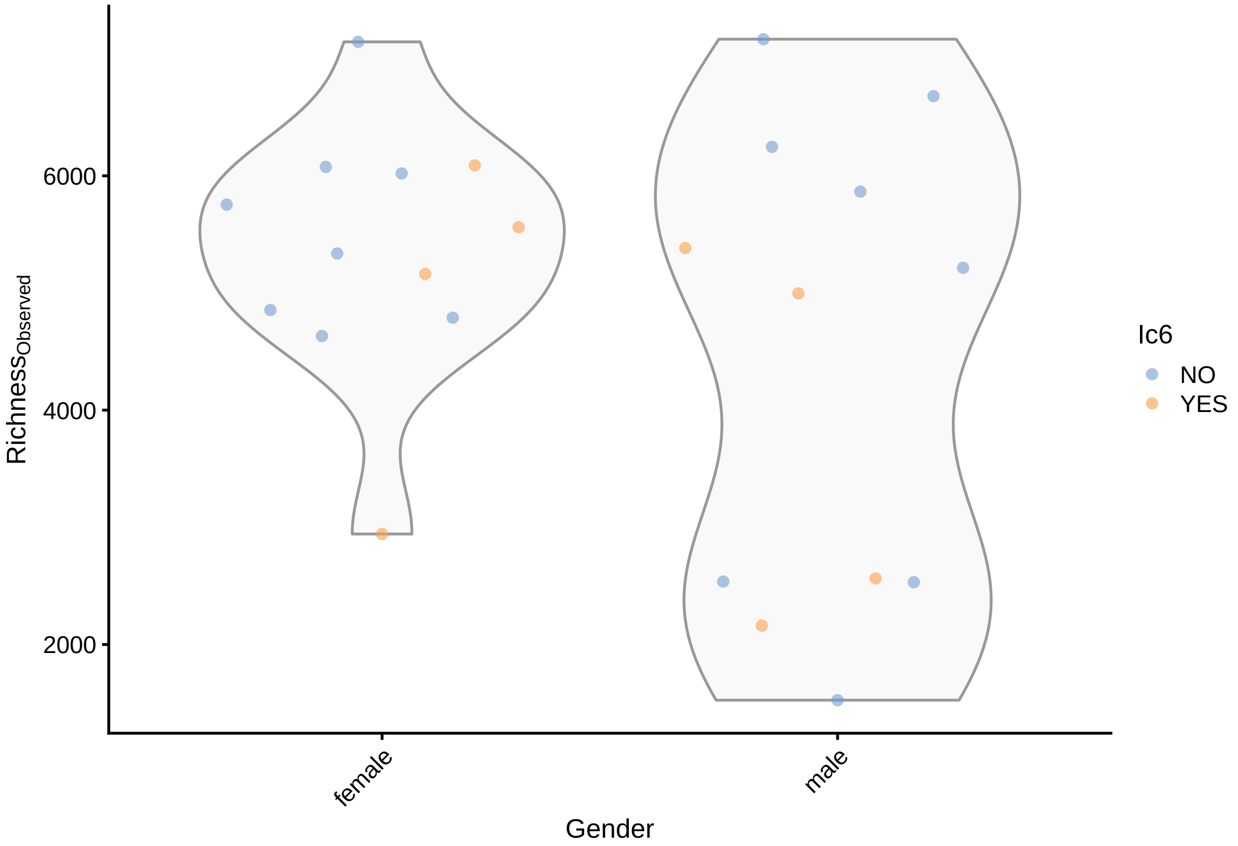

RichnessObserved
6000
4000
2000
female
male
Gender
Ic6
NO
YES

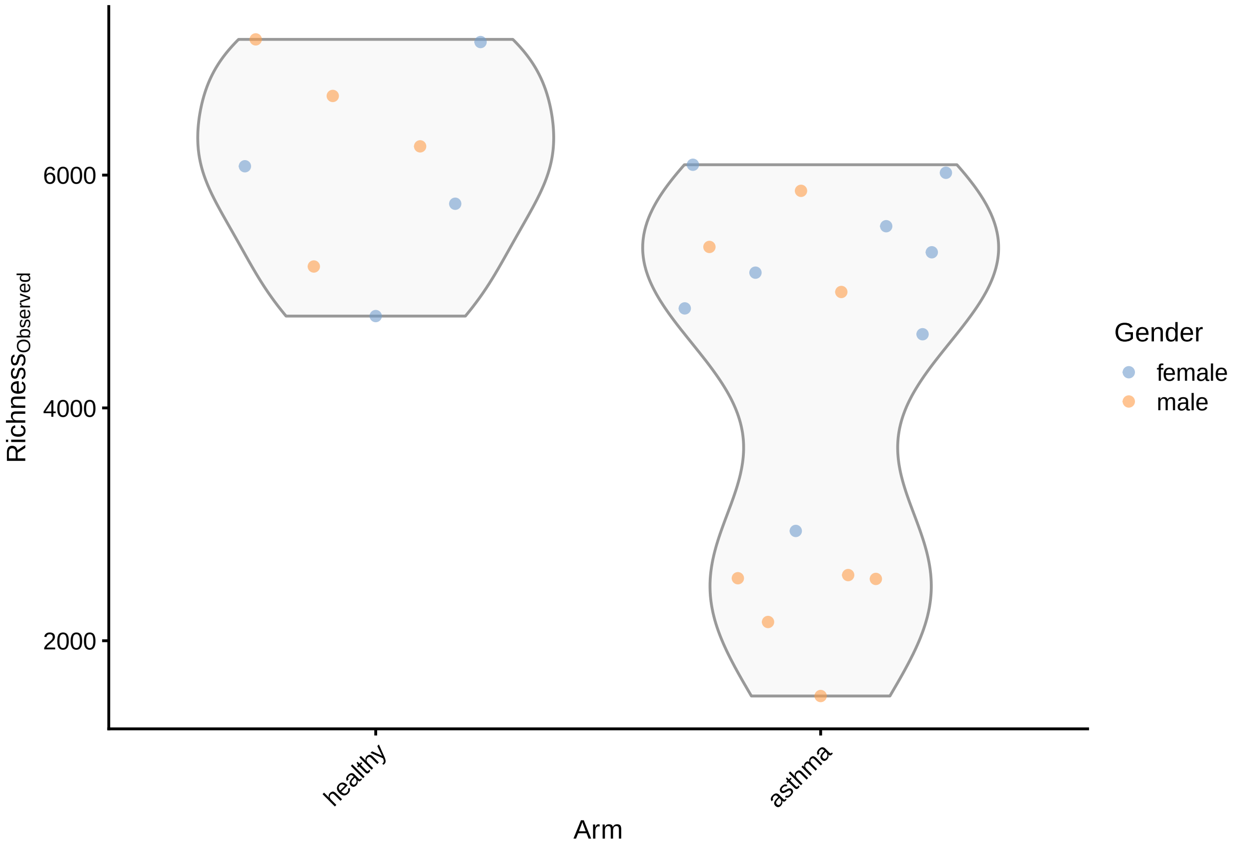

RichnessObserved
6000
4000
2000
healthy
asthma
Arm
Gender
female
male

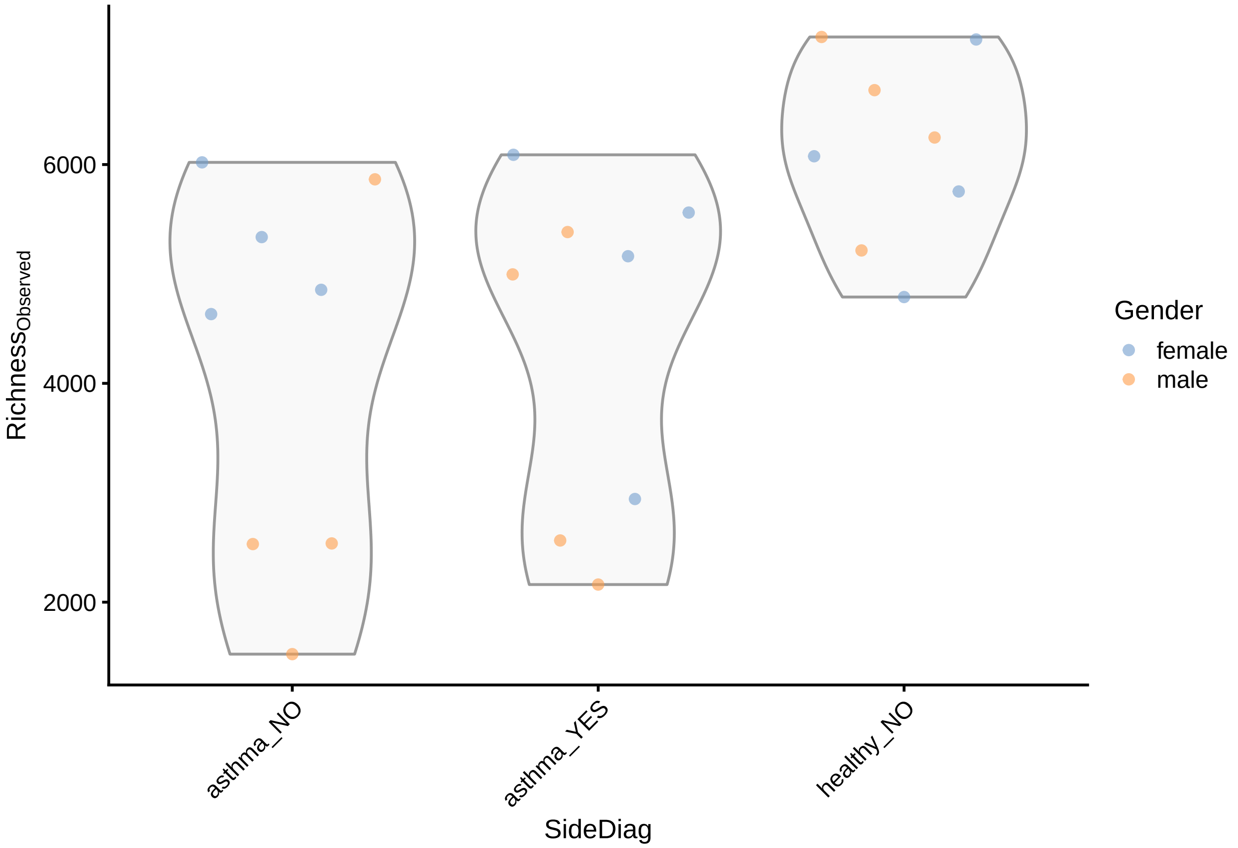

6000
4000
2000
Richness_Observed
asthma_NO
asthma_YES
healthy_NO
SideDiag
Gender
female
male

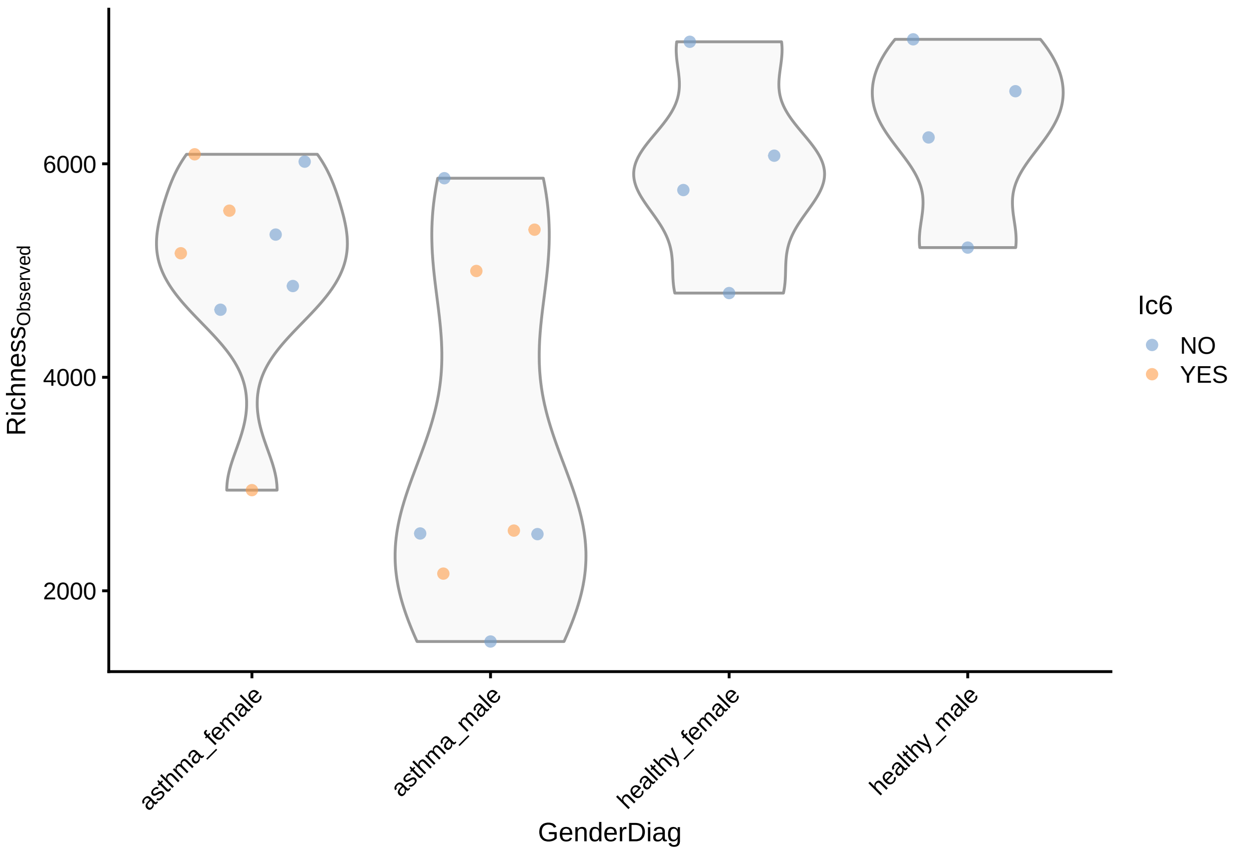

6000
4000
2000
Richness_Observed
asthma_female
asthma_male
healthy_female
healthy_male
GenderDiag
Ic6
NO
YES

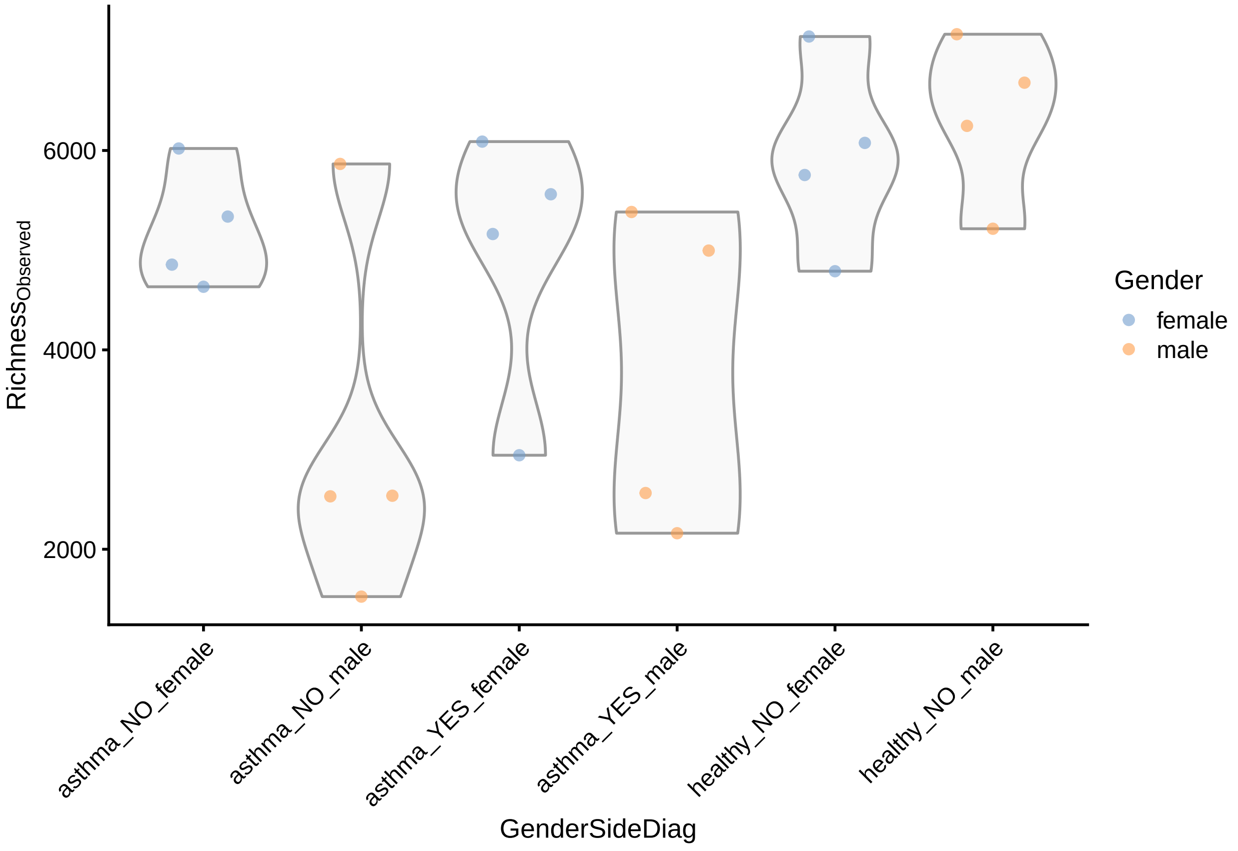
6000
4000
2000
Richness$_{Observed}$
asthma_NO_female
asthma_NO_male
asthma_YES_female
asthma_YES_male
healthy_NO_female
healthy_NO_male
GenderSideDiag
Gender
female
male

5 Shannon diversity

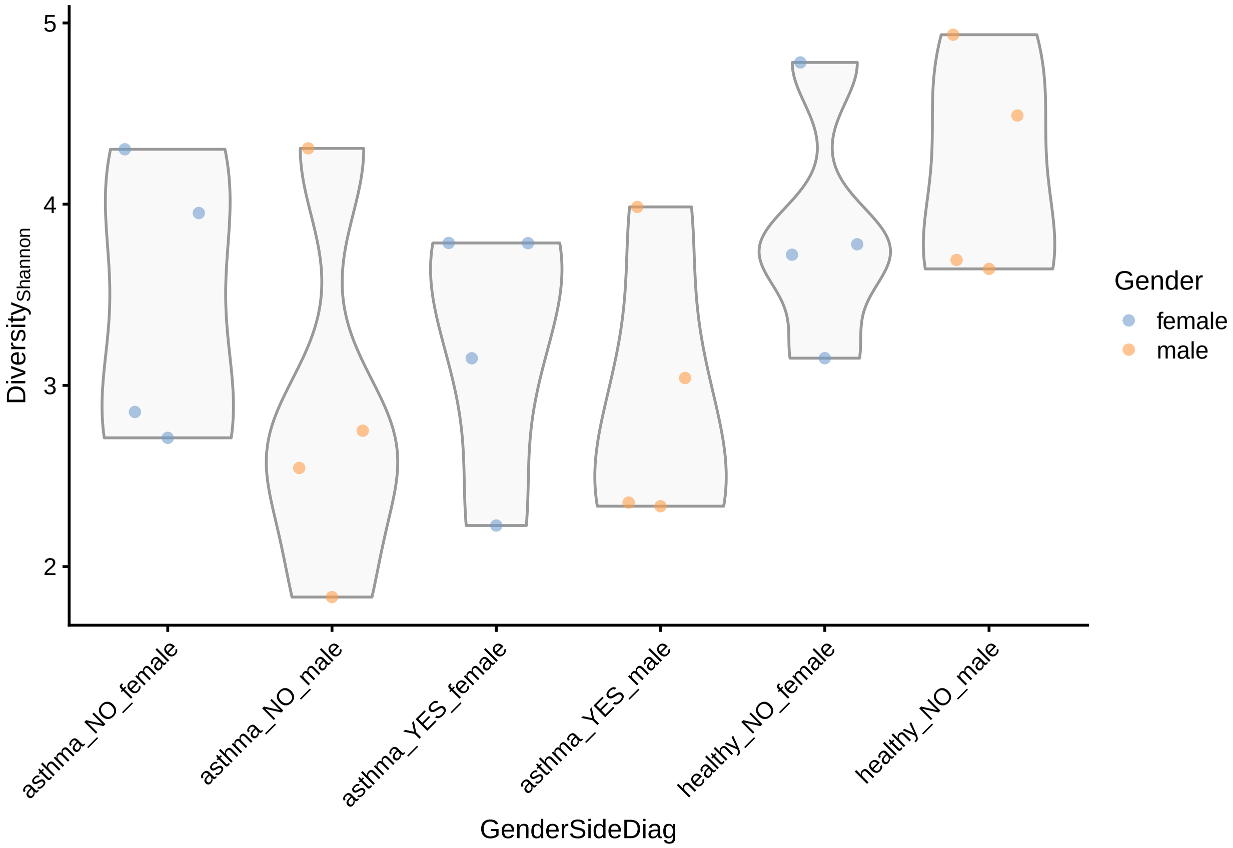

6 MDS

```
## initial  value 26.082940
## iter   5 value 14.805747
## iter  10 value 13.927737
## iter  15 value 13.607606
## iter  20 value 13.372692
## iter  20 value 13.372278
## iter  20 value 13.364132
## final  value 13.364132
## converged
```

```
## initial  value 20.748731
## iter   5 value 13.932961
## iter  10 value 10.668398
## iter  15 value 10.376008
## final  value 10.240957
## converged
```

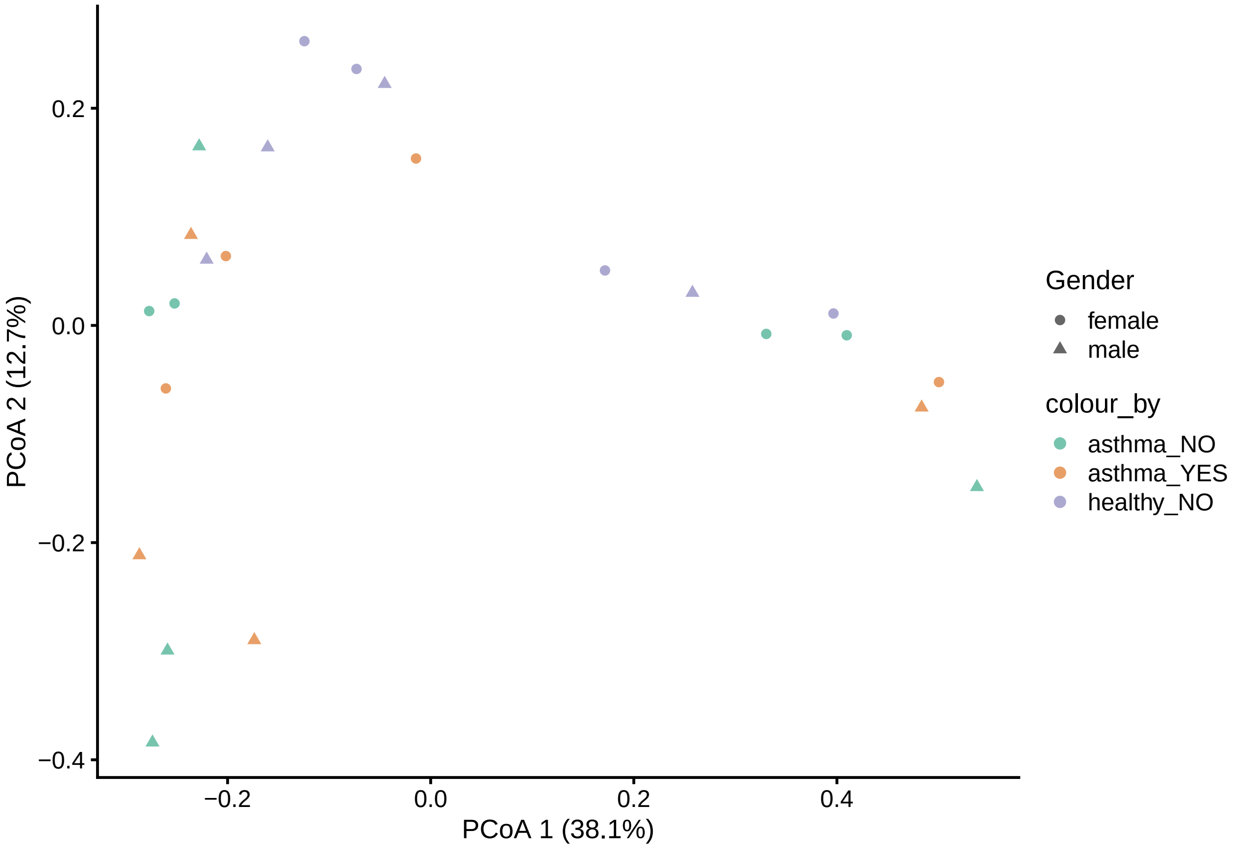
PCoA 2 (12.7%)
0.2
0.0
−0.2
−0.4
−0.2
0.0
0.2
0.4
PCoA 1 (38.1%)
Gender
female
male
colour_by
asthma_NO
asthma_YES
healthy_NO

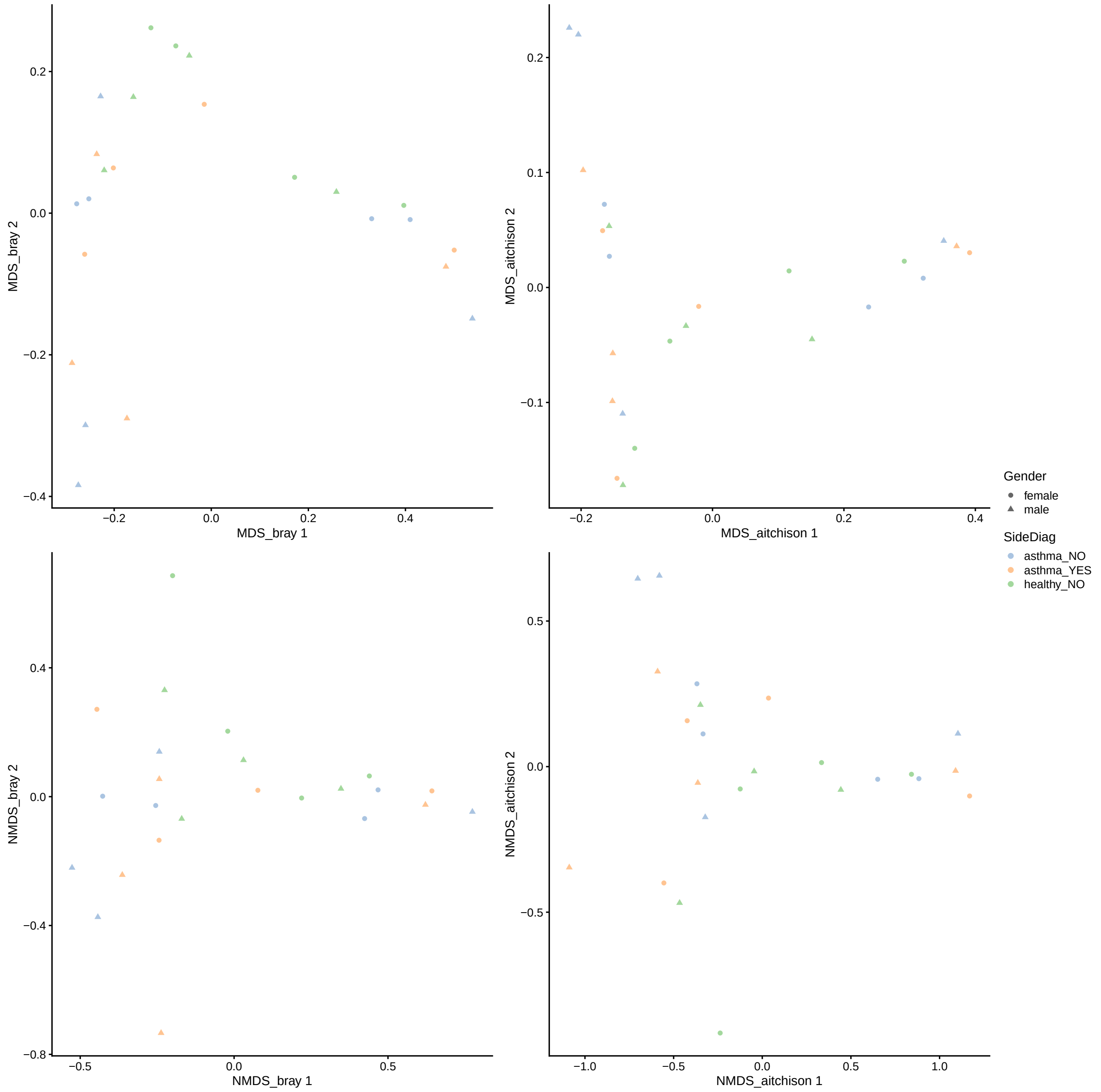
MDS_bray 1
MDS_bray 2
MDS_aitchison 1
MDS_aitchison 2
NMDS_bray 1
NMDS_bray 2
NMDS_aitchison 1
NMDS_aitchison 2
Gender
female
male
SideDiag
asthma_NO
asthma_YES
healthy_NO

7 Relative abundance

7.1 Genus

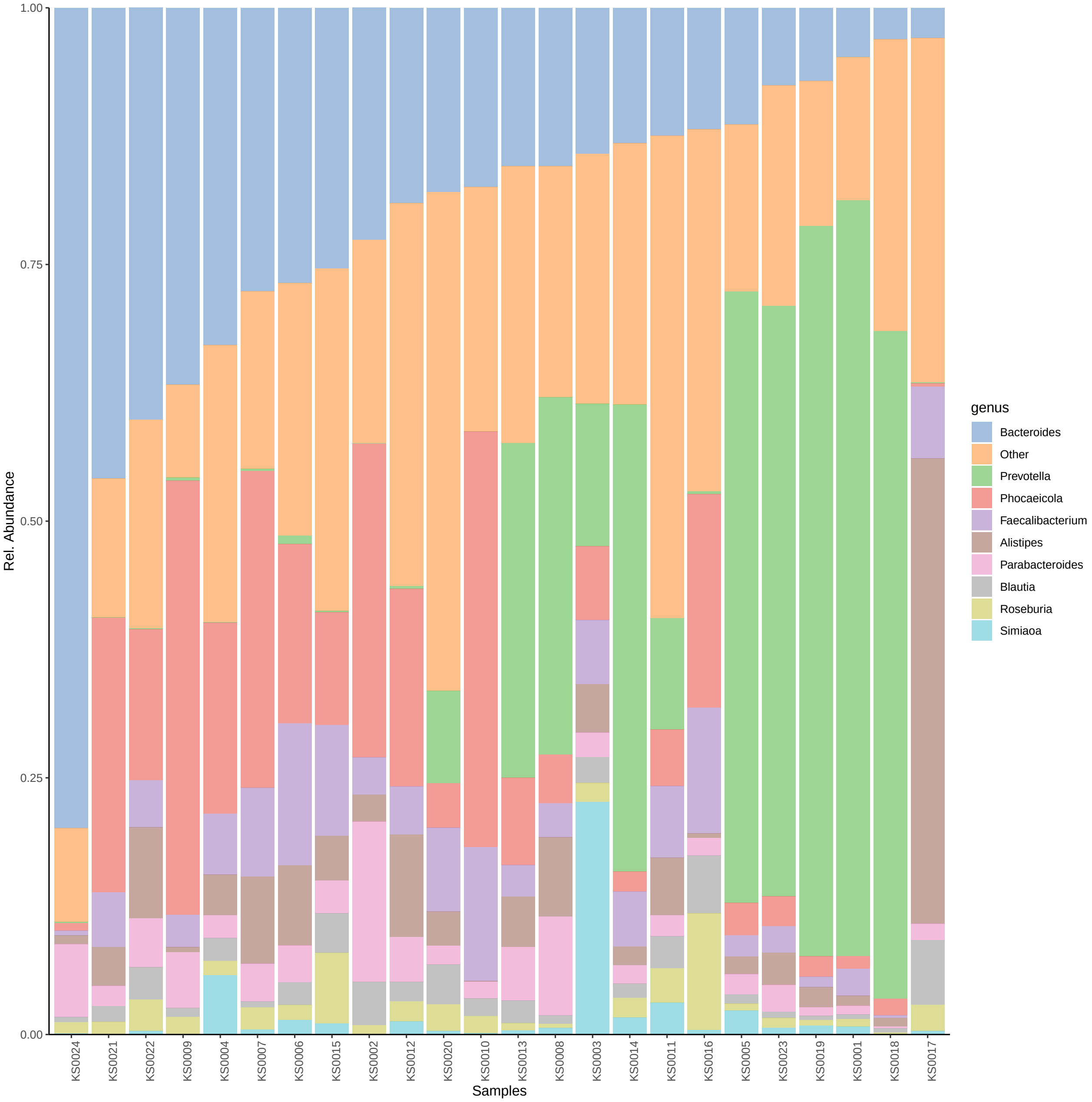

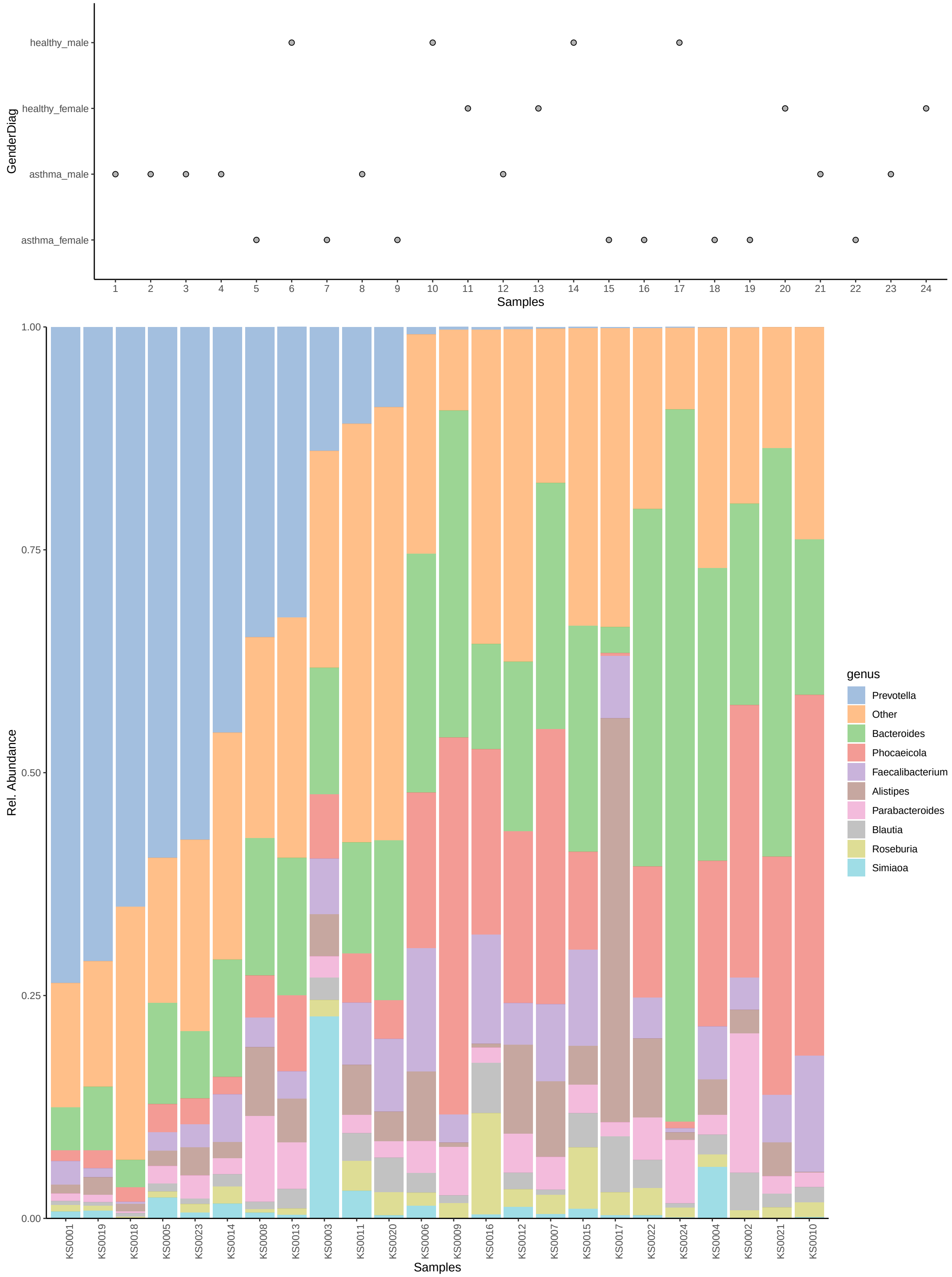

GenderDiag
healthy_male
healthy_female
asthma_male
asthma_female
1 2 3 4 5 6 7 8 9 10 11 12 13 14 15 16 17 18 19 20 21 22 23 24
Samples
Rel. Abundance
0.00
0.25
0.50
0.75
1.00
KS0001 KS0019 KS0018 KS0005 KS0023 KS0014 KS0008 KS0013 KS0003 KS0011 KS0020 KS0006 KS0009 KS0016 KS0012 KS0007 KS0015 KS0017 KS0022 KS0024 KS0004 KS0002 KS0021 KS0010
Samples
genus
Prevotella
Other
Bacteroides
Phocaeicola
Faecalibacterium
Alistipes
Parabacteroides
Blautia
Roseburia
Simiaoa

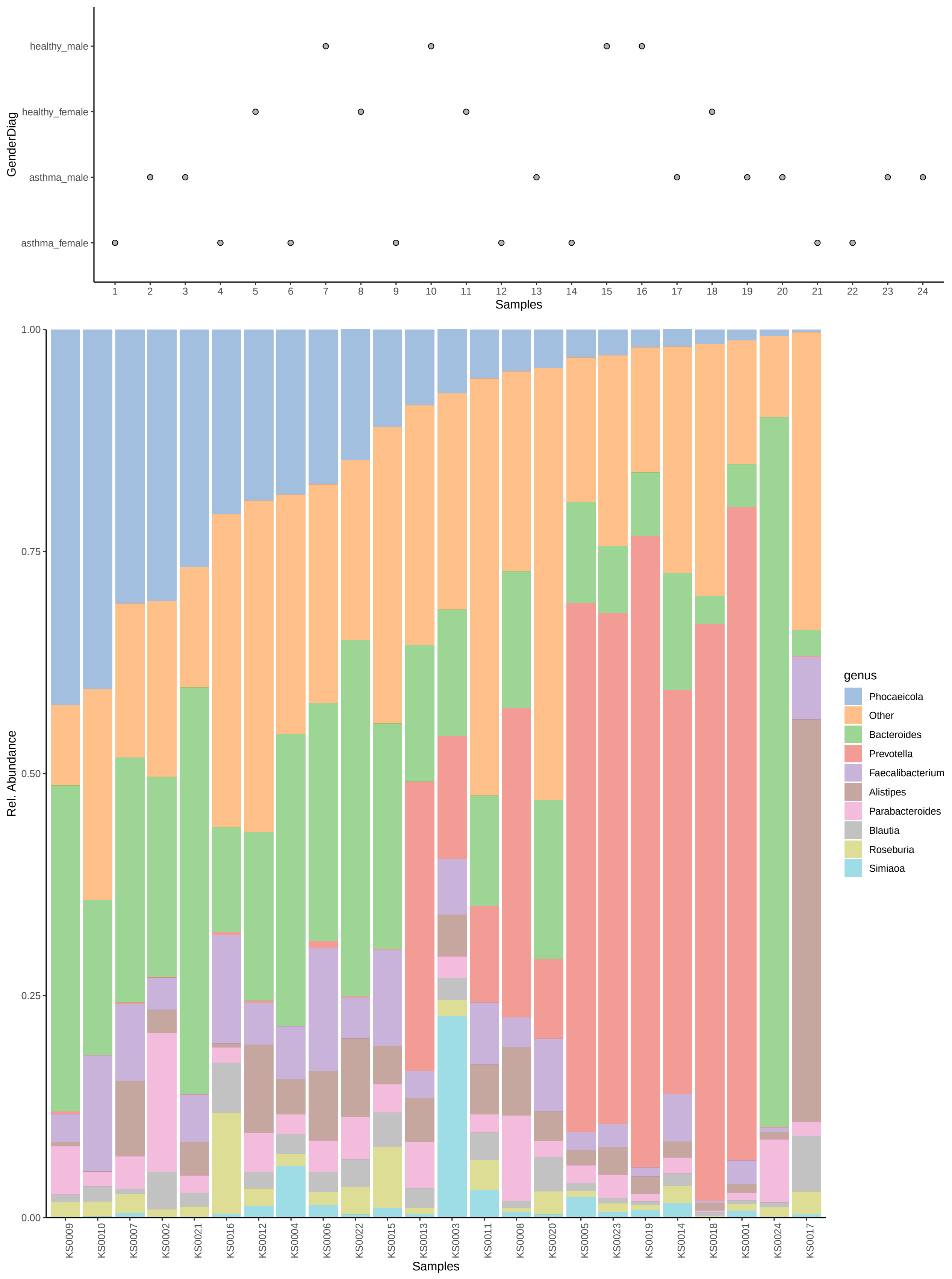
healthy_male
healthy_female
asthma_male
asthma_female
GenderDiag
1 2 3 4 5 6 7 8 9 10 11 12 13 14 15 16 17 18 19 20 21 22 23 24
Samples
Rel. Abundance
1.00
0.75
0.50
0.25
0.00
KS0009 KS0010 KS0007 KS0002 KS0021 KS0016 KS0012 KS0004 KS0006 KS0022 KS0015 KS0013 KS0003 KS0011 KS0008 KS0020 KS0005 KS0023 KS0019 KS0014 KS0018 KS0001 KS0024 KS0017
Samples
genus
Phocaeicola
Other
Bacteroides
Prevotella
Faecalibacterium
Alistipes
Parabacteroides
Blautia
Roseburia
Simiaoa

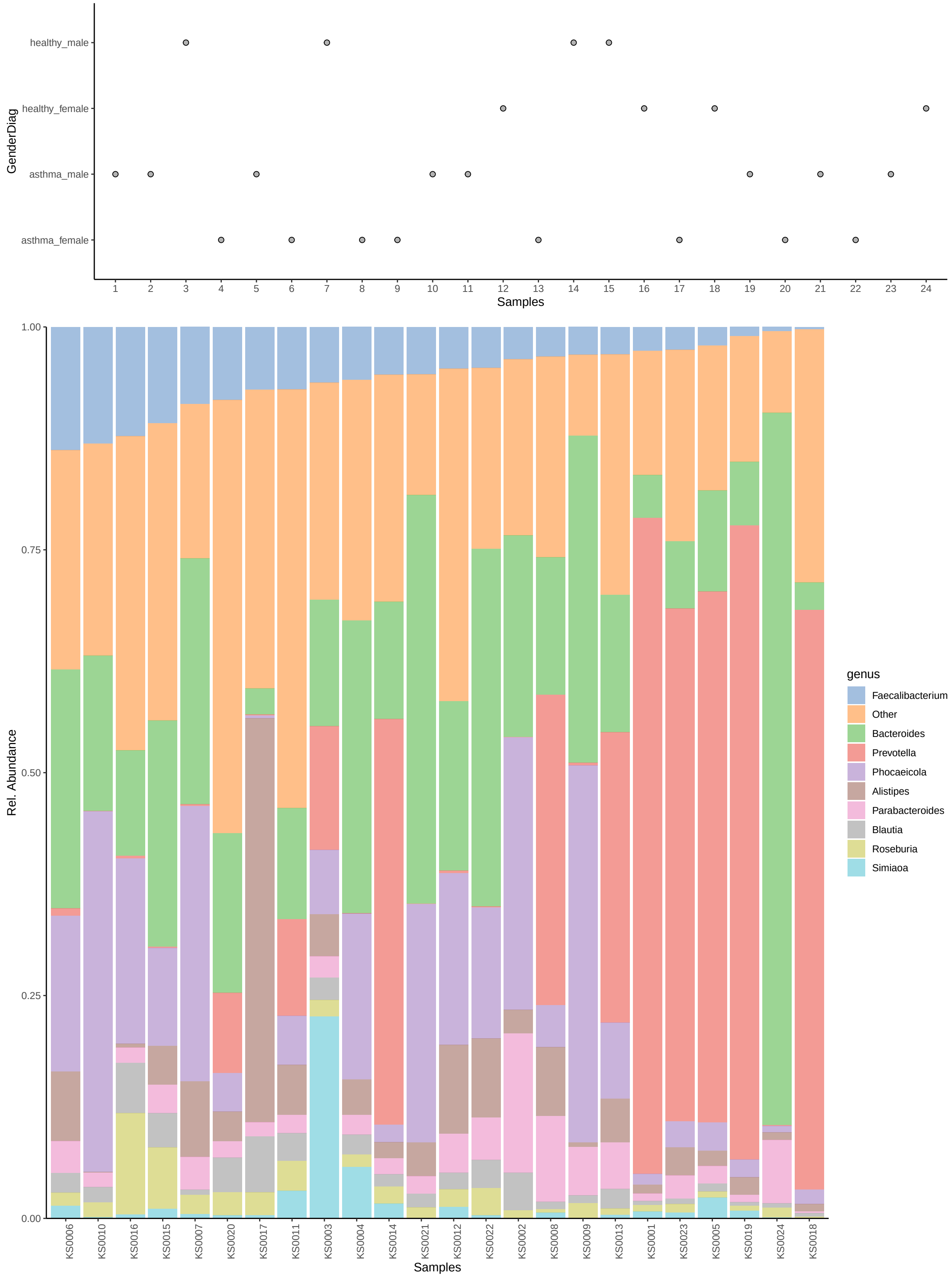

healthy_male
healthy_female
asthma_male
asthma_female
GenderDiag
1 2 3 4 5 6 7 8 9 10 11 12 13 14 15 16 17 18 19 20 21 22 23 24
Samples
1.00
0.75
0.50
0.25
0.00
Rel. Abundance
KS0006 KS0010 KS0016 KS0015 KS0007 KS0020 KS0017 KS0011 KS0003 KS0004 KS0014 KS0021 KS0012 KS0022 KS0002 KS0008 KS0009 KS0013 KS0001 KS0023 KS0005 KS0019 KS0024 KS0018
Samples
genus
Faecalibacterium
Other
Bacteroides
Prevotella
Phocaeicola
Alistipes
Parabacteroides
Blautia
Roseburia
Simiaoa

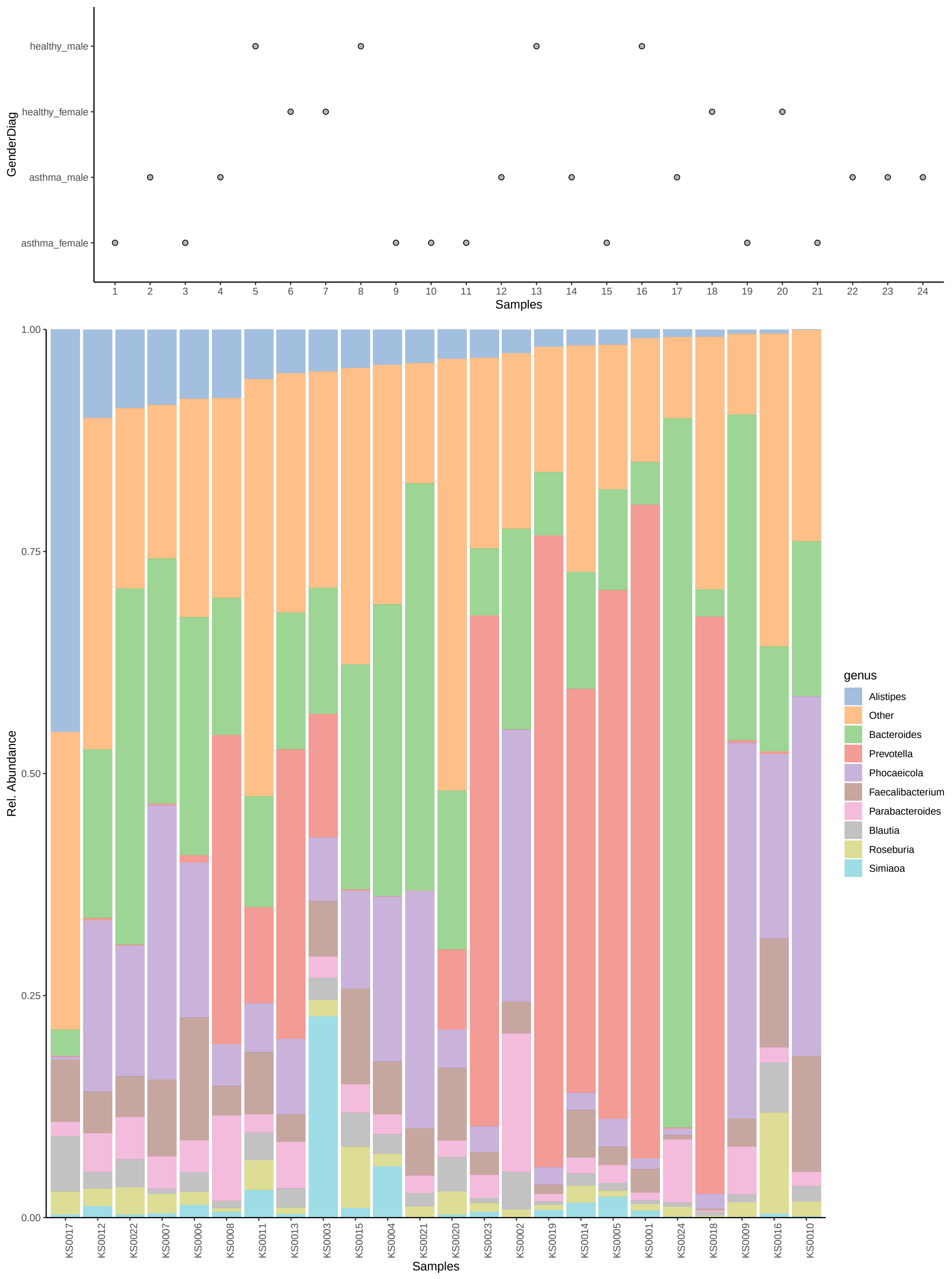
healthy_male
healthy_female
asthma_male
asthma_female
GenderDiag
1 2 3 4 5 6 7 8 9 10 11 12 13 14 15 16 17 18 19 20 21 22 23 24
Samples
1.00
0.75
0.50
0.25
0.00
Rel. Abundance
KS0017 KS0012 KS0022 KS0007 KS0006 KS0008 KS0011 KS0013 KS0003 KS0015 KS0004 KS0021 KS0020 KS0023 KS0002 KS0019 KS0014 KS0005 KS0001 KS0024 KS0018 KS0009 KS0016 KS0010
Samples
genus
Alistipes
Other
Bacteroides
Prevotella
Phocaeicola
Faecalibacterium
Parabacteroides
Blautia
Roseburia
Simiaoa

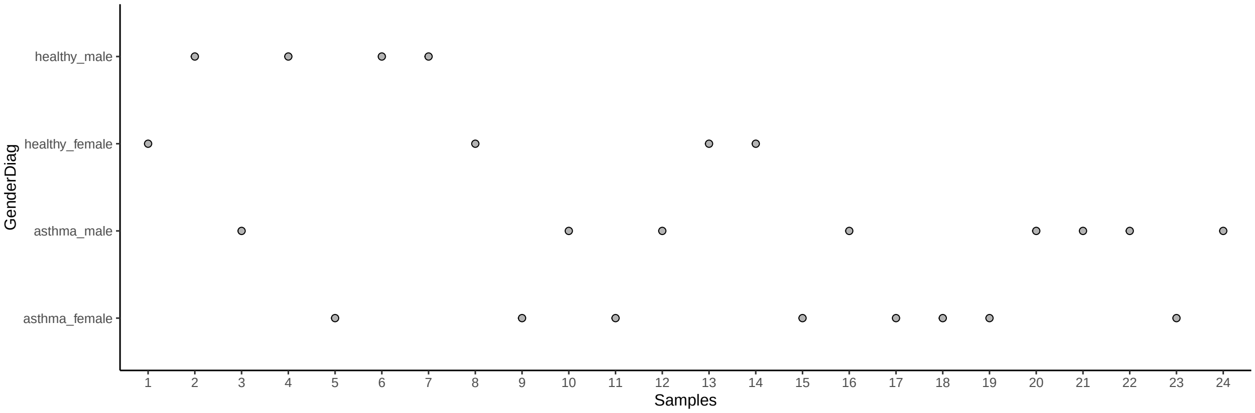
healthy_male
healthy_female
asthma_male
asthma_female
GenderDiag
1 2 3 4 5 6 7 8 9 10 11 12 13 14 15 16 17 18 19 20 21 22 23 24
Samples

7.2 Phylum

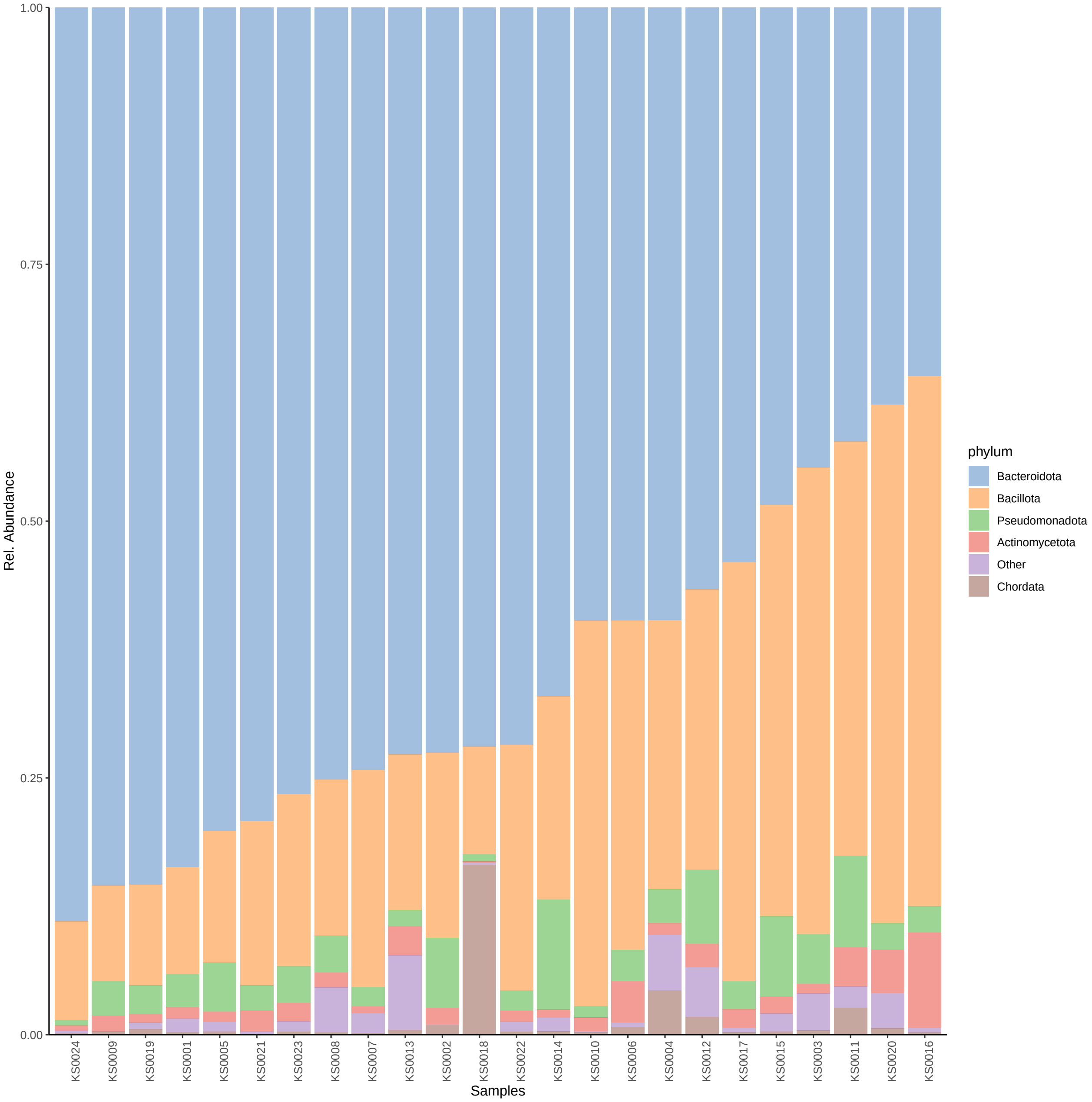
1.00
0.75
0.50
0.25
0.00
Rel. Abundance
KS0024
KS0009
KS0019
KS0001
KS0005
KS0021
KS0023
KS0008
KS0007
KS0013
KS0002
KS0018
KS0022
KS0014
KS0010
KS0006
KS0004
KS0012
KS0017
KS0015
KS0003
KS0011
KS0020
KS0016
Samples
phylum
Bacteroidota
Bacillota
Pseudomonadota
Actinomycetota
Other
Chordata

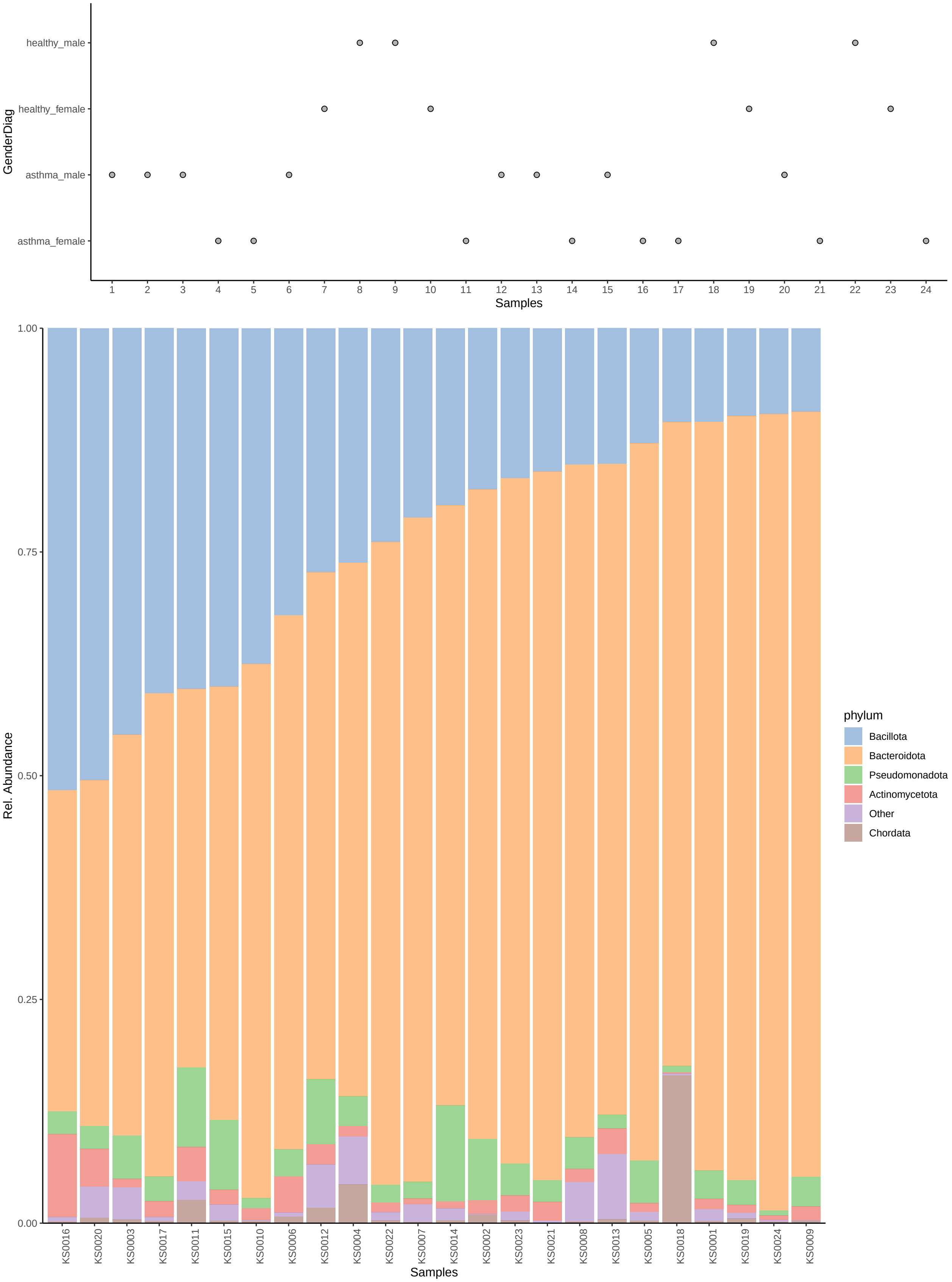

healthy_male
healthy_female
asthma_male
asthma_female
GenderDiag
1 2 3 4 5 6 7 8 9 10 11 12 13 14 15 16 17 18 19 20 21 22 23 24
Samples
1.00
0.75
0.50
0.25
0.00
Rel. Abundance
KS0016 KS0020 KS0003 KS0017 KS0011 KS0015 KS0010 KS0006 KS0012 KS0004 KS0022 KS0007 KS0014 KS0002 KS0023 KS0021 KS0008 KS0013 KS0005 KS0018 KS0001 KS0019 KS0024 KS0009
Samples
phylum
Bacillota
Bacteroidota
Pseudomonadota
Actinomycetota
Other
Chordata

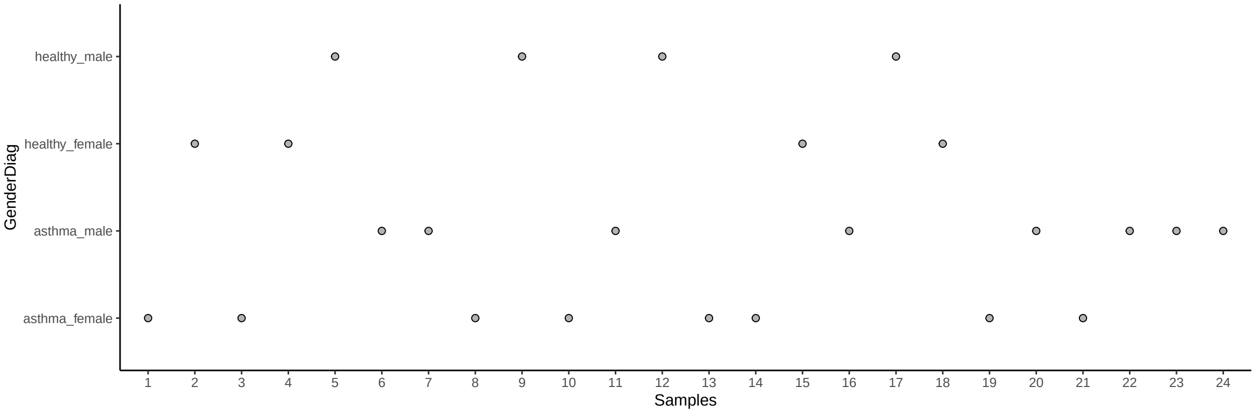

8 Appendix

8.1 Functions

8.2 Setup R

8.3 Versions

8.3.1 Document version

8.3.2 Session Info

Platform

- version: R version 4.3.0 (2023-04-21)
- os: macOS Monterey 12.5.1
- system: aarch64, darwin20
- ui: X11
- language: (EN)
- collate: en_US.UTF-8
- ctype: en_US.UTF-8
- tz: Asia/Tokyo
- date: 2023-10-31
- pandoc: 2.19.2 @ /Applications/RStudio.app/Contents/Resources/app/quarto/bin/tools/ (via rmarkdown)

Packages

	ondiskversion	loadedversion	date	source
abind	1.4.5	1.4-5	2016-07-21	CRAN (R 4.3.0)
ape	5.7.1	5.7-1	2023-03-13	CRAN (R 4.3.0)
aplot	0.2.1	0.2.1	2023-09-15	CRAN (R 4.3.0)
basilisk	1.13.3	1.13.3	2023-07-14	Bioconductor
basilisk.utils	1.13.3	1.13.3	2023-09-04	Bioconductor
beachmat	2.17.16	2.17.16	2023-09-03	Bioconductor

	ondiskversion	loadedversion	date	source
beeswarm	0.4.0	0.4.0	2021-06-01	CRAN (R 4.3.0)
Biobase	2.61.0	2.61.0	2023-06-02	Bioconductor
BiocGenerics	0.47.0	0.47.0	2023-06-02	Bioconductor
BiocManager	1.30.22	1.30.22	2023-08-08	CRAN (R 4.3.0)
BiocNeighbors	1.19.0	1.19.0	2023-04-25	Bioconductor
BiocParallel	1.35.4	1.35.4	2023-08-17	Bioconductor
BiocSingular	1.17.1	1.17.1	2023-07-09	Bioconductor
Biostrings	2.69.2	2.69.2	2023-07-05	Bioconductor
bit	4.0.5	4.0.5	2022-11-15	CRAN (R 4.3.0)
bit64	4.0.5	4.0.5	2020-08-30	CRAN (R 4.3.0)
bitops	1.0.7	1.0-7	2021-04-24	CRAN (R 4.3.0)
blob	1.2.4	1.2.4	2023-03-17	CRAN (R 4.3.0)
bluster	1.11.4	1.11.4	2023-08-02	Bioconductor
cachem	1.0.8	1.0.8	2023-05-01	CRAN (R 4.3.0)
callr	3.7.3	3.7.3	2022-11-02	CRAN (R 4.3.0)
cli	3.6.1	3.6.1	2023-03-23	CRAN (R 4.3.0)
cluster	2.1.4	2.1.4	2022-08-22	CRAN (R 4.3.0)
codetools	0.2.19	0.2-19	2023-02-01	CRAN (R 4.3.0)
colorspace	2.1.0	2.1-0	2023-01-23	CRAN (R 4.3.0)
corrplot	0.92	0.92	2021-11-18	CRAN (R 4.3.0)
cowplot	1.1.1	1.1.1	2020-12-30	CRAN (R 4.3.0)
crayon	1.5.2	1.5.2	2022-09-29	CRAN (R 4.3.0)
curl	5.0.2	5.0.2	2023-08-14	CRAN (R 4.3.0)
data.table	1.14.8	1.14.8	2023-02-17	CRAN (R 4.3.0)
DBI	1.1.3	1.1.3	2022-06-18	CRAN (R 4.3.0)
dbplyr	2.3.3	2.3.3	2023-07-07	CRAN (R 4.3.0)

	ondiskversion	loadedversion	date	source
DECIPHER	2.29.0	2.29.0	2023-07-07	Bioconductor
decontam	1.21.0	1.21.0	2023-05-20	Bioconductor
DelayedArray	0.27.10	0.27.10	2023-07-28	Bioconductor
DelayedMatrixStats	1.23.4	1.23.4	2023-08-09	Bioconductor
devtools	2.4.5	2.4.5	2022-10-11	CRAN (R 4.3.0)
digest	0.6.33	0.6.33	2023-07-07	CRAN (R 4.3.0)
dir.expiry	1.9.0	1.9.0	2023-05-11	Bioconductor
DirichletMultinomial	1.43.0	1.43.0	2023-06-02	Bioconductor
dplyr	1.1.3	1.1.3	2023-09-03	CRAN (R 4.3.0)
ellipsis	0.3.2	0.3.2	2021-04-29	CRAN (R 4.3.0)
evaluate	0.21	0.21	2023-05-05	CRAN (R 4.3.0)
fansi	1.0.4	1.0.4	2023-01-22	CRAN (R 4.3.0)
farver	2.1.1	2.1.1	2022-07-06	CRAN (R 4.3.0)
fastmap	1.1.1	1.1.1	2023-02-24	CRAN (R 4.3.0)
filelock	1.0.2	1.0.2	2018-10-05	CRAN (R 4.3.0)
forcats	1.0.0	1.0.0	2023-01-29	CRAN (R 4.3.0)
fs	1.6.3	1.6.3	2023-07-20	CRAN (R 4.3.0)
generics	0.1.3	0.1.3	2022-07-05	CRAN (R 4.3.0)
GenomeInfoDb	1.37.4	1.37.4	2023-09-07	Bioconductor
GenomeInfoDbData	1.2.10	1.2.10	2023-05-15	Bioconductor
GenomicRanges	1.53.1	1.53.1	2023-06-02	Bioconductor
ggbeeswarm	0.7.2	0.7.2	2023-04-29	CRAN (R 4.3.0)
ggforce	0.4.1	0.4.1	2022-10-04	CRAN (R 4.3.0)
ggfun	0.1.3	0.1.3	2023-09-15	CRAN (R 4.3.0)
ggnewscale	0.4.9	0.4.9	2023-05-25	CRAN (R 4.3.0)
ggplot2	3.4.3	3.4.3	2023-08-14	CRAN (R 4.3.0)

	ondiskversion	loadedversion	date	source
ggplotify	0.1.2	0.1.2	2023-08-09	CRAN (R 4.3.0)
ggraph	2.1.0	2.1.0	2022-10-09	CRAN (R 4.3.0)
ggrepel	0.9.3	0.9.3	2023-02-03	CRAN (R 4.3.0)
ggtree	3.9.1	3.9.1	2023-08-11	Bioconductor
glue	1.6.2	1.6.2	2022-02-24	CRAN (R 4.3.0)
graphlayouts	1.0.0	1.0.0	2023-05-01	CRAN (R 4.3.0)
gridExtra	2.3	2.3	2017-09-09	CRAN (R 4.3.0)
gridGraphics	0.5.1	0.5-1	2020-12-13	CRAN (R 4.3.0)
gtable	0.3.4	0.3.4	2023-08-21	CRAN (R 4.3.0)
hoardr	0.5.3	0.5.3	2023-01-26	CRAN (R 4.3.0)
htmltools	0.5.6	0.5.6	2023-08-10	CRAN (R 4.3.0)
htmlwidgets	1.6.2	1.6.2	2023-03-17	CRAN (R 4.3.0)
httpuv	1.6.11	1.6.11	2023-05-11	CRAN (R 4.3.0)
igraph	1.5.1	1.5.1	2023-08-10	CRAN (R 4.3.0)
IRanges	2.35.2	2.35.2	2023-06-23	Bioconductor
irlba	2.3.5.1	2.3.5.1	2022-10-03	CRAN (R 4.3.0)
jsonlite	1.8.7	1.8.7	2023-06-29	CRAN (R 4.3.0)
knitr	1.44	1.44	2023-09-11	CRAN (R 4.3.0)
labeling	0.4.3	0.4.3	2023-08-29	CRAN (R 4.3.0)
later	1.3.1	1.3.1	2023-05-02	CRAN (R 4.3.0)
lattice	0.21.8	0.21-8	2023-04-05	CRAN (R 4.3.0)
lazyeval	0.2.2	0.2.2	2019-03-15	CRAN (R 4.3.0)
lifecycle	1.0.3	1.0.3	2022-10-07	CRAN (R 4.3.0)
lubridate	1.9.2	1.9.2	2023-02-10	CRAN (R 4.3.0)
magrittr	2.0.3	2.0.3	2022-03-30	CRAN (R 4.3.0)
MASS	7.3.60	7.3-60	2023-05-04	CRAN (R 4.3.0)

	ondiskversion	loadedversion	date	source
Matrix	1.6.1.1	1.6-1.1	2023-09-18	CRAN (R 4.3.1)
MatrixGenerics	1.13.1	1.13.1	2023-07-26	Bioconductor
matrixStats	1.0.0	1.0.0	2023-06-02	CRAN (R 4.3.0)
memoise	2.0.1	2.0.1	2021-11-26	CRAN (R 4.3.0)
mgcv	1.9.0	1.9-0	2023-07-11	CRAN (R 4.3.0)
mia	1.9.16	1.9.16	2023-09-15	Github (microbiome/mia@210d90e38cc48c34f434c0db5d8a640fa0048f84)
miaViz	1.9.0	1.9.0	2023-05-19	Bioconductor
mime	0.12	0.12	2021-09-28	CRAN (R 4.3.0)
miniUI	0.1.1.1	0.1.1.1	2018-05-18	CRAN (R 4.3.0)
MOFA2	1.9.2	1.9.2	2023-09-20	Bioconductor
MultiAssayExperiment	1.27.5	1.27.5	2023-08-25	Bioconductor
munsell	0.5.0	0.5.0	2018-06-12	CRAN (R 4.3.0)
nlme	3.1.163	3.1-163	2023-08-09	CRAN (R 4.3.0)
openxlsx	4.2.5.2	4.2.5.2	2023-02-06	CRAN (R 4.3.0)
pander	0.6.5	0.6.5	2022-03-18	CRAN (R 4.3.0)
patchwork	1.1.3	1.1.3	2023-08-14	CRAN (R 4.3.0)
permute	0.9.7	0.9-7	2022-01-27	CRAN (R 4.3.0)
pheatmap	1.0.12	1.0.12	2019-01-04	CRAN (R 4.3.0)
pillar	1.9.0	1.9.0	2023-03-22	CRAN (R 4.3.0)
pkgbuild	1.4.2	1.4.2	2023-06-26	CRAN (R 4.3.0)
pkgconfig	2.0.3	2.0.3	2019-09-22	CRAN (R 4.3.0)
pkgload	1.3.2.1	1.3.2.1	2023-07-08	CRAN (R 4.3.0)
plyr	1.8.8	1.8.8	2022-11-11	CRAN (R 4.3.0)
png	0.1.8	0.1-8	2022-11-29	CRAN (R 4.3.0)
polyclip	1.10.4	1.10-4	2022-10-20	CRAN (R 4.3.0)
prettyunits	1.1.1	1.1.1	2020-01-24	CRAN (R 4.3.0)

	ondiskversion	loadedversion	date	source
processx	3.8.2	3.8.2	2023-06-30	CRAN (R 4.3.0)
profvis	0.3.8	0.3.8	2023-05-02	CRAN (R 4.3.0)
promises	1.2.1	1.2.1	2023-08-10	CRAN (R 4.3.0)
ps	1.7.5	1.7.5	2023-04-18	CRAN (R 4.3.0)
purrr	1.0.2	1.0.2	2023-08-10	CRAN (R 4.3.0)
R6	2.5.1	2.5.1	2021-08-19	CRAN (R 4.3.0)
rappdirs	0.3.3	0.3.3	2021-01-31	CRAN (R 4.3.0)
RColorBrewer	1.1.3	1.1-3	2022-04-03	CRAN (R 4.3.0)
Rcpp	1.0.11	1.0.11	2023-07-06	CRAN (R 4.3.0)
RCurl	1.98.1.12	1.98-1.12	2023-03-27	CRAN (R 4.3.0)
remotes	2.4.2.1	2.4.2.1	2023-07-18	CRAN (R 4.3.0)
reshape2	1.4.4	1.4.4	2020-04-09	CRAN (R 4.3.0)
reticulate	1.32.0	1.32.0	2023-09-11	CRAN (R 4.3.0)
rlang	1.1.1	1.1.1	2023-04-28	CRAN (R 4.3.0)
rmarkdown	2.25	2.25	2023-09-18	CRAN (R 4.3.1)
RSQLite	2.3.1	2.3.1	2023-04-03	CRAN (R 4.3.0)
rstudioapi	0.15.0	0.15.0	2023-07-07	CRAN (R 4.3.0)
rsvd	1.0.5	1.0.5	2021-04-16	CRAN (R 4.3.0)
Rtsne	0.16	0.16	2022-04-17	CRAN (R 4.3.0)
S4Arrays	1.1.6	1.1.6	2023-08-31	Bioconductor
S4Vectors	0.39.1	0.39.1	2023-06-02	Bioconductor
ScaledMatrix	1.9.1	1.9.1	2023-05-03	Bioconductor
scales	1.2.1	1.2.1	2022-08-20	CRAN (R 4.3.0)
scater	1.29.4	1.29.4	2023-08-24	Bioconductor
scuttle	1.11.2	1.11.2	2023-08-03	Bioconductor
sessioninfo	1.2.2	1.2.2	2021-12-06	CRAN (R 4.3.0)

	ondiskversion	loadedversion	date	source
shiny	1.7.5	1.7.5	2023-08-12	CRAN (R 4.3.0)
SingleCellExperiment	1.23.0	1.23.0	2023-04-25	Bioconductor
SparseArray	1.1.12	1.1.12	2023-08-31	Bioconductor
sparseMatrixStats	1.13.4	1.13.4	2023-08-14	Bioconductor
stringi	1.7.12	1.7.12	2023-01-11	CRAN (R 4.3.0)
stringr	1.5.0	1.5.0	2022-12-02	CRAN (R 4.3.0)
SummarizedExperiment	1.31.1	1.31.1	2023-05-01	Bioconductor
taxizedb	0.3.1	0.3.1	2023-04-03	CRAN (R 4.3.0)
tibble	3.2.1	3.2.1	2023-03-20	CRAN (R 4.3.0)
tidygraph	1.2.3	1.2.3	2023-02-01	CRAN (R 4.3.0)
tidyr	1.3.0	1.3.0	2023-01-24	CRAN (R 4.3.0)
tidyselect	1.2.0	1.2.0	2022-10-10	CRAN (R 4.3.0)
tidytree	0.4.5	0.4.5	2023-08-10	CRAN (R 4.3.0)
timechange	0.2.0	0.2.0	2023-01-11	CRAN (R 4.3.0)
treeio	1.25.4	1.25.4	2023-08-25	Bioconductor
TreeSummarizedExperiment	2.9.0	2.9.0	2023-07-07	Bioconductor
tweenr	2.0.2	2.0.2	2022-09-06	CRAN (R 4.3.0)
urlchecker	1.0.1	1.0.1	2021-11-30	CRAN (R 4.3.0)
usethis	2.2.2	2.2.2	2023-07-06	CRAN (R 4.3.0)
utf8	1.2.3	1.2.3	2023-01-31	CRAN (R 4.3.0)
uwot	0.1.16	0.1.16	2023-06-29	CRAN (R 4.3.0)
vctrs	0.6.3	0.6.3	2023-06-14	CRAN (R 4.3.0)
vegan	2.6.4	2.6-4	2022-10-11	CRAN (R 4.3.0)
vipor	0.4.5	0.4.5	2017-03-22	CRAN (R 4.3.0)
viridis	0.6.4	0.6.4	2023-07-22	CRAN (R 4.3.0)
viridisLite	0.4.2	0.4.2	2023-05-02	CRAN (R 4.3.0)

	ondiskversion	loadedversion	date	source
withr	2.5.0	2.5.0	2022-03-03	CRAN (R 4.3.0)
xfun	0.40	0.40	2023-08-09	CRAN (R 4.3.0)
xtable	1.8.4	1.8-4	2019-04-21	CRAN (R 4.3.0)
XVector	0.41.1	0.41.1	2023-06-02	Bioconductor
yaml	2.3.7	2.3.7	2023-01-23	CRAN (R 4.3.0)
yulab.utils	0.1.0	0.1.0	2023-09-20	CRAN (R 4.3.0)
zip	2.3.0	2.3.0	2023-04-17	CRAN (R 4.3.0)
zlibbioc	1.47.0	1.47.0	2023-05-20	Bioconductor
