# Supplementary material for: Metagenome and metabolome study on inhaled corticosteroids in asthma patients with side effects
Source: J Integr Bioinform. 2025 Jun 24;22(3):20240062. doi: 10.1515/jib-2024-0062 (PMC13066347; doi:10.1515/jib-2024-0062)
Supplement: Supplementary file 4 — Supplementary Material Details [file j_jib-2024-0062_suppl_004.pdf]

Выписка из протокола

Заседание №16

Комитета по Биоэтике (КБЭ) Национальный хирургический центр им. М.М. Мамакеева.

Дата заседания: 07 июня 2022г.

Состав КБЭ: заведующий отделением гнойной хирургии НХЦ д.м.н., профессор Эралиев Бейшенбек Ашимович

Секретарь КБЭ: Койуманов К.

Члены КБЭ: к.м.н. Ибраимов Б.А.

к.м.н. Элеманов Н.Ч.

адвокат Айткулов Ж.О.

Повестка дня

Рассмотрение материалов научного исследования «Исследования нового метода многомерного анализа данных метамикробиома, включая метаболомику у пациентов использующих ингаляционные глюкокортикостероиды» по специальности общественное здравоохранение. Главный исследователь аспирант кафедры общественного здоровья и здравоохранения Осмонов Б.Р. проект осуществляется при проф Горьянина И. в рамках сотрудничества с КГМА им И.К. Ахунбаева.

На рассмотрение представлены следующие документы:

1. Заявление на имя председателя КБЭ;
2. Аннотация исследования;
3. Информационный лист для пациента (на русском и кыргызском языках)
4. Лист информированного согласия (на русском и кыргызском языках)

Принятое решение:

Постановили: комитет по биоэтике (КБЭ) НХЦ им М.М. Мамакеева считает, что представленные на этическую экспертизу, материалы исследования могут быть одобрены.

Решение КБЭ: одобрить проведение исследования на тему: «Исследования нового метода многомерного анализа данных метамикробиома, включая метаболомику у пациентов использующих ингаляционные глюкокортикостероиды» главный исследователь: Осмонов Б.Р.

Председатель КБЭ,
Д.м.н., профессор — Эралиев Б.А.

Секретарь КБЭ — Койчуманов К.
